# Gut Microbiota Mediate the Metabolism of Colonic Prostaglandins

**DOI:** 10.21203/rs.3.rs-8856024/v1

**Published:** 2026-02-20

**Authors:** Nan Jing, Guodong Cao, Han Qiao, Matthew L. Edin, Katherine Z. Sanidad, Yige Wang, Eleanor Zhu, Jianzhong Ge, Jun Yang, Fred B. Lih, Emma Luu, Augustine Arredondo, Lauren E. Hutchinson, Quancai Sun, Jianan Zhang, Vladimir Yeliseyev, Jonathan C. P. Steele, Richard K. Phipps, Stephen K. Wrigley, Tetsuo Kokubun, Ravi N. Manohar, Emily Hopkins, Yi Wang, Ginger L. Milne, Sunny Hei Wong, Andreas J. Bäumler, Justin B. Siegel, Matthew R. Redinbo, Melody Y. Zeng, Darryl C. Zeldin, Zongwei Cai, Guodong Zhang

**Affiliations:** 1 Department of Nutrition, University of California, Davis, CA, USA.; 2 Department of Food Science and Technology, National University of Singapore, Singapore.; 3 State Key Laboratory of Environmental and Biological Analysis, Department of Chemistry, Hong Kong Baptist University, Hong Kong, SAR, China.; 4 Division of Intramural Research, National Institute of Environmental Health Sciences, National Institutes of Health, Research Triangle Park, Durham, NC, USA.; 5 Gale and Ira Drukier Institute for Children’s Health, Weill Cornell Medicine, New York, NY, USA.; 6 Department of Pediatrics, Weill Cornell Medicine, New York, NY, USA.; 7 Genome Center, University of California, Davis, CA, USA.; 8 Biological and Agricultural Engineering, University of California, Davis, CA, USA.; 9 Department of Entomology and Nematology, University of California, Davis, CA, USA.; 10 Departments of Chemistry, Biochemistry, Microbiology and Genomics, University of North Carolina at Chapel Hill, Chapel Hill, NC, USA.; 11 Department of Health, Nutrition, and Food Sciences, Florida State University, Tallahassee, FL, USA.; 12 Department of Food Science, University of Massachusetts, Amherst, MA, USA.; 13 Massachusetts Host-Microbiota Center, Department of Pathology, Brigham and Women’s Hospital, Boston, MA, USA.; 14 Hypha Discovery Limited, Abingdon, United Kingdom.; 15 Division of Clinical Pharmacology, Department of Medicine, Vanderbilt University Medical Center, Nashville, TN, USA.; 16 Lee Kong Chian School of Medicine, Nanyang Technological University, Singapore, Singapore.; 17 Department of Medical Microbiology and Immunology, University of California, Davis, CA, USA.; 18 Department of Chemistry, University of California, Davis, CA, USA.; 19 Department of Biochemistry and Molecular Medicine, University of California, Davis, CA, USA.; 20 Eastern Institute of Technology, Ningbo, Zhejiang, China.

## Abstract

Prostaglandins (PGs) are endogenous lipid signaling molecules that regulate diverse physiological and pathological processes, and their biosynthetic pathways are targets of widely used drugs such as aspirin and other nonsteroidal anti-inflammatory drugs (NSAIDs)^1–7^. However, previous research of PG biosynthesis has focused on host metabolic pathways; as such, the contribution of gut microbiota remains unknown^1^. Here, we demonstrate a colonic pathway in which gut microbes directly participate in PG metabolism, thereby regulating intestinal PG levels and their biological effects. Comparison of germ-free and conventionally raised mice reveals that the gut microbiota markedly increase gut levels of multiple PGs. This effect is driven by bacterial β-glucuronidases (GUS), which hydrolyze host-derived PG glucuronides – less active or inactive conjugates – to regenerate bioactive, free-form PGs. Administration of purified GUS enzyme or mono-colonization with wild-type or GUS-deficient bacteria in germ-free mice demonstrates the critical role for microbial GUS in regulating intestinal PG levels and downstream biological responses. Together, these findings reveal a previously unrecognized function of the gut microbiota in PG metabolism and highlight microbial pathways as potential targets for modulating colonic PG signaling to prevent or treat gastrointestinal disease.

Prostaglandins (PGs) are endogenous lipid-based signaling molecules that orchestrate a vast array of physiological and pathological processes, notably the regulation of inflammation and immune responses^1–7^. Given their central role, the PG biosynthetic pathways are targets of many widely used drugs^1,2^. For example, aspirin and other nonsteroidal anti-inflammatory drugs (NSAIDs), which inhibit cyclooxygenases (COXs), the rate-limiting enzymes in PG production, are the most widely used drugs to treat inflammation, pain, and related disorders^1,2^. First discovered in the 1930s^8–10^ and recognized with the Nobel Prize in Physiology or Medicine in 1982^11^, the PG biosynthetic pathways has been extensively studied^1^. The classical pathway for PG biosynthesis begins with the release of fatty acids, notably arachidonic acid (ARA), from membrane phospholipids by phospholipase A_2_ (PLA_2_) and related enzymes, generating intracellular free fatty acids^1,3^. These fatty acids are subsequently metabolized by COXs and downstream enzymes, including prostaglandin E synthase (PTGES), prostaglandin D synthase (PTGDS), and prostaglandin F synthase (PTGFS), to produce various PG species such as prostaglandin E_2_ (PGE_2_) (Fig. 1a)^1^. However, previous studies of PG biosynthesis have focused on host metabolic pathways, as both the substrates and enzymes involved are localized within host tissues^1^. The contribution of gut microbiota in this process remains unknown.

## Gut microbiota elevate colonic PGs

**Fig. 1.**
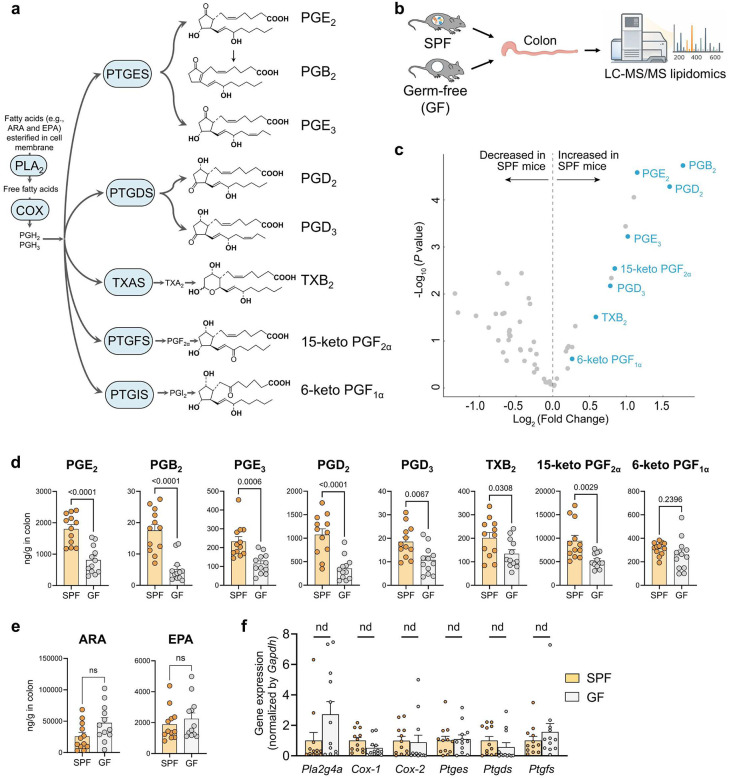
Gut microbiota elevate colonic concentrations of PGs. (**a**) Classical pathway for PG biosynthesis: Upon cellular stimulations, fatty acids (e.g., ARA and EPA) incorporated in membrane phospholipids are enzymatically released to form intracellular free fatty acids, which are then metabolized by a series of intracellular metabolic enzymes, including COXs and PG synthases, to generate various PGs. (**b**) The colon tissues of conventional (specific-pathogen free, SPF) and germ-free (GF) C57BL/6 mice were collected for LC-MS/MS-based targeted lipidomics analysis. (**c**) LC-MS/MS lipidomics analysis showed that PGs are among the most dramatically increased lipid metabolites in the colons of SPF mice compared to those of GF mice. Each plotted point in the volcano plot represents a metabolite. The blue points represent PGs, and the grey points represent other types of metabolites. Fold Change represents the level of the lipid metabolite in the SPF mice to that of the GF mice. (**d**) Colonic concentrations of numerous PGs are increased in SPF mice compared to GF mice. (**e**) Colonic levels of ARA and EPA, the fatty acid precursors of PG biosynthesis, are not significantly different between SPF and GF mice. “ns” means “not significant” (P > 0.05). (**f**) qRT-PCR analysis revealed no significant differences in the expression of genes involved in PG biosynthesis in colons of SPF mice *vs*. GF mice. Multiple-comparison correction was performed using the false discovery rate (FDR) method, and “nd” denotes genes that did not reach significance after FDR correction (q ≥ Q). Data are presented as mean ± SEM, n = 12 mice per group. Abbreviations: PGs, prostaglandins; ARA, arachidonic acid; EPA, eicosapentaenoic acid; COX, cyclooxygenase; PLA_2_, phospholipase A_2_; PTGES, prostaglandin E synthase; PTGDS, prostaglandin D synthase; PTGFS, prostaglandin F synthase; PTGIS, prostaglandin I synthase; TXAS, thromboxane synthase.

Since gut microbes predominantly reside in the colon^12^, where they may directly interact with colonic PGs, we hypothesize that the gut microbiota influence PG levels in the colon. To test this, we employed liquid chromatography-tandem mass spectrometry (LC-MS/MS)-based targeted lipidomics, which can analyze 56 lipid metabolites including COX-derived PGs, as well as lipoxygenase- and cytochrome P450-produced lipid metabolites, to compare profiles of lipid metabolites in the colons of germ-free (GF) versus conventionally raised (specific pathogen-free, SPF) C57BL/6 mice (see the scheme of the experiment in Fig. 1b).

The lipidomics analysis shows that among the detected lipid metabolites, PGs are the most dramatically altered metabolites in the colons of SPF mice compared to those of GF mice (Fig. 1c, see heatmap in Extended Data Fig. 1; complete results in Table S1; raw concentration data for each individual mouse are provided in the supplemental Excel file). A series of PGs, including PGE_2_, PGB_2_, PGE_3_, PGD_2_, PGD_3_, thromboxane B_2_ (TXB_2_, a stable metabolite of TXA_2_), and 15-keto PGF_2α_ (a stable metabolite of PGF_2α_), are significantly increased in the colons of SPF mice compared to GF mice (Fig. 1d). For instance, the colonic concentration of PGE_2_ was ~1800 ng/g in SPF mice and ~800 ng/g in GF mice, indicating that gut microbiota led to a >2-fold increase in colonic PGE_2_ levels (P < 0.0001) (Fig. 1d). In addition, 6-keto PGF_1α_, a stable metabolite of PGI_2_, also showed a similar trend, though the effect was not statistically significant (Fig. 1d). This finding demonstrates that gut microbiota has a major impact on colonic PG levels.

We further quantified PGE2, the most prominent PG involved in inflammation and cancer^1^, across multiple tissues from GF and SPF mice. Among the tissues analyzed, PGE_2_ was highly abundant in colon tissues of both GF and SPF mice. Consistent with the findings above, PGE_2_ levels were significantly increased in gut tissues, including the colon and cecum, of SPF mice compared with GF mice (P < 0.01). In contrast, PGE_2_ concentrations did not differ significantly in other tissues, including the lung, heart, spleen, small intestine, stomach, and kidney (Extended Data Fig. 2). Together, these results indicate that gut microbiota selectively modulate PG levels within the gut.

## Gut microbiota participate in PGE_2_ metabolism

We tested whether gut microbiota elevate colonic PG levels through modulating host pathways involved in the biosynthesis of PGs^1^. First, we found that compared to GF mice, colonic levels of ARA and eicosapentaenoic acid (EPA), the fatty acid precursors for the biosynthesis of PGs, were not significantly changed in SPF mice (Fig. 1e)^1^. Next, qRT-PCR showed that compared to SPF mice, the colonic expression of the genes encoding the enzymes involved in PG biosynthesis, including *Pla*_*2*_, *Cox-1*, *Cox-2*, *Ptges*, *Ptgds*, and *Ptgfs*, was not significantly different in GF mice (Fig. 1f)^1^. This finding suggests that the microbiota’s effects on colonic PGs are not due to altered host metabolic pathways.

We next tested whether gut microbiota elevates colonic PGs by directly participating in their metabolism. Given that gut microbiota increase colonic concentrations of numerous PGs, we hypothesize that gut microbes catalyze metabolic reactions targeting shared structural features of these PGs, thereby elevating their colonic concentrations. A common structural feature of various PGs is the presence of carboxylic acid (-COOH) and hydroxyl (-OH) groups (see chemical structures of PGs in Fig. 1a), moieties that could be metabolized by phase II enzymes such as uridine diphosphate-glucuronosyltransferases (UGT) or sulfotransferases (SULT), forming glucuronide or sulfate conjugates^13,14^. We hypothesize that gut microbes act on these PG conjugates, converting them to free PGs and therefore increasing colonic PG concentrations.

**Fig 2.**
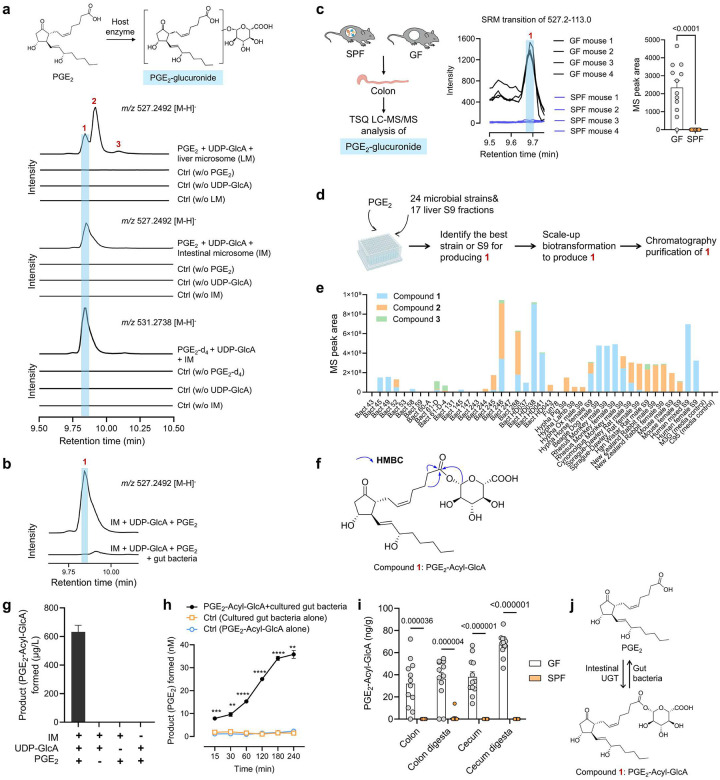
Gut microbiota mediate the metabolism of colonic PGE_2_. (**a**) LC-HRMS analysis showed that liver microsome converts PGE_2_ into three putative glucuronide conjugates (compounds 1, 2, and 3) via UGT-dependent manners (Upper); Intestinal microsome converts PGE_2_ into a single predominant glucuronide conjugate (compound 1) (Middle); Intestinal microsome converts isotope-labelled PGE_2_ (PGE_2_-d_4_) into a single predominant glucuronide conjugate at the same retention time as compound 1 (Bottom). (**b**) Compound 1 is degraded after incubation with mouse fecal bacteria. (**c**) TSQ LC–MS/MS analysis showed that compound 1 is detected in the colons of GF mice, but not in those of SPF mice (n = 12 mice per group). (**d**) We used a strategy of biotransformation to prepare the authentic standard of compound 1. (**e**) After screening 24 microbial strains and 17 mammalian S9 fractions, we found that HD038, a recombinant *Streptomyces* strain, is among the most effective biocatalysts to prepare compound 1. (**f**) NMR analyses, notably HMBC NMR, support that compound 1 is PGE_2_-Acyl-GlcA. (**g**) Intestinal microsome converts PGE_2_ into PGE_2_-Acyl-GlcA via UGT-dependent manners. (**h**) Mouse fecal bacteria catalyze the conversion of PGE_2_-Acyl-GlcA to PGE_2_. (**i**) PGE_2_-Acyl-GlcA is detected in the gut tissues of GF mice, but not in SPF mice (n = 12 mice per group). (**j**) Schematic illustration of PGE_2_ metabolism by host UGT enzymes and fecal bacteria. Data are presented as mean ± SEM. ** P < 0.01, *** P < 0.001, **** P < 0.0001.

To test this hypothesis, we first examined whether PGs could be metabolized by host UGT or SULT enzymes to generate the corresponding conjugates. Previous studies using radioactive substrate-based enzymatic assays suggest that PGE_2_ could be glucuronidated by UGT enzymes^15,16^; however, detailed characterization of reaction products has not been performed. Here, we studied the roles of host UGT or SULT enzymes in the metabolism of PGE_2_. Liquid chromatography-high resolution mass spectrometry (LC-HRMS) analysis of *m/z* 527.2492 [M-H]^−^, corresponding to the expected accurate mass of PGE_2_ mono-glucuronide conjugates, revealed that incubating PGE_2_ with liver microsomes and uridine diphosphate-glucuronic acid (UDP-GlcA, the cofactor for UGT enzymes^13^) resulted in the formation of three putative PGE_2_-glucuronides (**1**, **2** and **3**), which were absent in the negative control reactions (Fig. 2a). MS/MS fragmentation analysis further supported that the compounds **1**, **2** and **3** are PGE_2_-glucuronides (Extended Data Fig. 3). The formation of three isomers is consistent with the fact that PGE_2_ has three potential sites for glucuronidation: a COOH group and two OH groups. Surprisingly, when we conducted the same enzymatic reaction using intestinal microsomes, only compound **1** was formed as a single predominant product (Fig. 2a). To further validate this, we incubated isotope-labelled PGE_2_ (PGE_2_-d_4_), with or without intestinal microsomes or UDP-GlcA. LC-HRMS analysis of m/z 531.2737 [M-H]^−^, consistent with the accurate mass of PGE_2_-d_4_-glucuronide, showed the formation of a single peak at the same retention time as compound **1** (Fig. 2a). We also investigated whether PGE_2_ could be metabolized by host SULT enzymes to form sulfate conjugates. However, LC-HRMS analysis indicated that incubating liver S9 or intestinal microsome with PGE_2_ and 3’-phospho-adenosyl-5’-phosphosulfate (the cofactor for SULT enzymes^14^) did not result in the formation of any PGE_2_ sulfate conjugates (Extended Data Fig. 4). Overall, these results support that PGE_2_ is a substrate of host UGT enzymes, while it is not metabolized by SULT enzymes.

Having shown that intestinal enzymes convert PGE_2_ to a single dominant, putative glucuronide conjugate (compound **1**), we tested the interactions of gut microbes with compound **1**. Incubating mouse fecal bacteria with compound **1** led to the near-complete disappearance of **1** (Fig. 2b). This result supports the conclusion that gut microbes can process compound **1**. Next, we developed a triple quadrupole LC–MS/MS (TSQ LC–MS/MS) method that enables highly sensitive analysis of compounds **1–3** (Extended Data Fig. 5) and found that compound **1** was present in the colons of GF mice but absent in those of SPF mice (Fig. 2c), further suggesting that gut microbes could metabolize compound **1**. The other two PGE_2_-glucuronides, **2** and **3**, were not detected in the colons of SPF or GF mice (Fig. 2c, see raw LC-HRMS spectrum in supplemental information). This observation is consistent with our previous *in vitro* findings, which showed that **1** is the predominant glucuronide metabolite of PGE_2_ produced by intestinal microsome. Overall, these results support our hypothesis that PGE_2_ is metabolized by intestinal UGT enzymes to form its glucuronide conjugate (compound **1**), which is subsequently degraded by gut microbes.

To confirm that compound **1** is indeed a PGE_2_ glucuronide, we elucidated its chemical structure by preparing an authentic standard. The preparation of PGE_2_ glucuronides presents significant technical hurdles. PGE2 has multiple sites for glucuronidation and is known for its poor stability^17^, which complicates direct chemical derivatization to convert PGE_2_ into its glucuronide conjugates. Moreover, the total chemical synthesis of PGE_2_ is characterized by lengthy, multi-step synthetic routes with low overall yields, making the total synthesis of its glucuronide conjugates challenging^18,19^. To circumvent these limitations, we utilized a biotransformation strategy to produce compound **1** (see the scheme of the experiment in Fig. 2d). After screening 24 microbial strains and 17 mammalian liver S9 fractions with known glucuronidation ability, we identified HD038, a proprietary recombinant *Streptomyces* strain with endogenous production of UDP-GlcA and engineered to express a bacterial UGT enzyme originally cloned from a wildtype bacterial strain, as the most effective in converting PGE_2_ to **1** (Fig. 2e). Scale-up biotransformation feeding 100 mg PGE_2_ followed by chromatographic purifications yielded ~ 10 mg of **1**. NMR analysis, particularly heteronuclear multiple bond correlation (HMBC) 2D-NMR, confirmed that **1** is PGE_2_-Acyl-β-glucuronide (PGE_2_-Acyl-GlcA), with the β-glucuronide moiety linked to the -COOH group of PGE_2_ (see the chemical structure of PGE_2_-Acyl-GlcA and selected HMBC correlations in Fig. 2f, raw 1D and 2D NMR spectrum in Extended Data Fig. 6–11, and the summary of NMR data in Table S2). Stability testing showed that similar to PGE_2_^17^, PGE_2_-Acyl-GlcA is unstable under alkaline pH (Extended Data Fig. 12).

Using the authentic standard of PGE_2_-Acyl-GlcA, we investigated the roles of host and microbial enzymes in PGE_2_ metabolism. Incubation of PGE_2_ with intestinal or liver microsomes in the presence of UDP-GlcA resulted in the formation of PGE_2_-Acyl-GlcA, which was not observed in negative control reactions (see intestinal microsome reaction in Fig. 2g and liver microsome reaction in Extended Data Fig. 13). These results support a potential role of host UGT enzymes in catalyzing the glucuronidation of PGE_2_. Incubation of PGE_2_-Acyl-GlcA with cultured mouse fecal bacteria or fecal bacterial enzymes (prepared via lysis of mouse fecal bacteria) resulted in time-dependent production of free PGE_2_, indicating microbial deconjugation of PGE_2_-Acyl-GlcA (see the reactions using cultured fecal bacteria in Fig. 2h and fecal bacterial enzyme in Extended Data Fig. 14). In the absence of exogenous PGE_2_-Acyl-GlcA, LC-MS/MS analysis of isolated fecal bacteria detected neither PGE_2_-Acyl-GlcA nor PGE_2_ (Extended Data Fig. 15), suggesting that gut microbes do not endogenously produce PGs or their derivatives. Consistent with these *in vitro* findings, LC-MS/MS analysis revealed that PGE_2_-Acyl-GlcA was present in the gut tissues, including colon, cecum, colon digesta, and cecum digesta, of GF mice, but not in SPF mice (Fig. 2i). Collectively, these results support a model that after the formation of PGE_2_ in gut tissues, it is further metabolized by intestinal UGT enzymes to form PGE_2_-Acyl-GlcA. This conjugated metabolite can enter the gut lumen, as supported by its presence in colon and cecum digesta of GF mice (Fig. 2j), where it can directly interact with the gut microbiota and is hydrolyzed by gut microbes to regenerate free PGE_2_.

## Microbial enzymes in PGE_2_ metabolism

**Fig 3.**
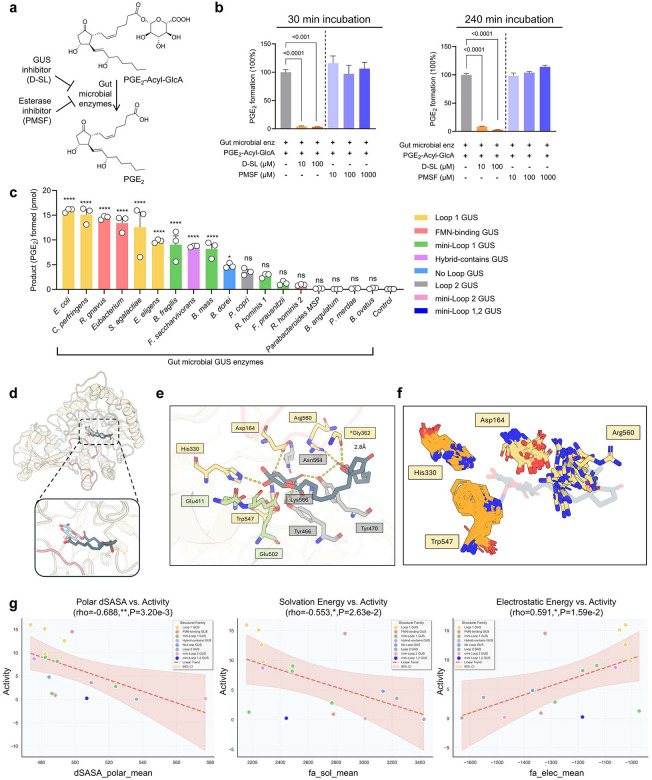
Specifical gut microbial enzymes mediate the metabolism of colonic PGE_2_. (**a**) We examined the effect of D-SL (a pan-GUS inhibitor) and PMSF (an esterase inhibitor) on fecal bacterial enzyme-catalyzed processing of PGE_2_-Acyl-GlcA. (**b**) Treatment with D-SL attenuated, while PMSF had no effect, on fecal bacterial enzyme-mediated processing of PGE_2_-Acyl-GlcA. (**c**) Specific microbial GUS enzymes process PGE_2_-Acyl-GlcA. (**d**) Overall binding mode of PGE2-Acyl-GlcA in the active site of *E. coli* GUS (EcGUS), a representative Loop-1 GUS protein, from Rosetta docking. The enzyme is shown in ribbon representation with Loop-1 highlighted in pink. The substrate is shown in stick representation with GlcA sugar moiety in light blue. (**e**) Detailed hydrogen bonding network between EcGUS and PGE2-Acyl-GlcA. Catalytic residues (Glu411 and Glu502) are shown in green and conserved binding residues are shown in gray, while computationally predicted interaction partners are shown in yellow. (**f**) Conservation analysis of predicted substrate-binding residues. Structural overlay of 18 GUS variants showing conserved residues (shown in orange) Trp547 and His330 versus variable residues Asp164 and Arg560 (shown in yellow). Variable residues are absent in multiple low-activity variants and represent priority mutagenesis targets. Substrate shown in dark blue. (**g**) Scatter plots of experimental GUS activity versus top three significant Rosetta-derived features: mean change in polar SASA upon product binding, electrostatic energy, and solvation energy. Each plot shows individual GUS isoforms (n = 18) colored by structural family, with linear trend lines and 95% confidence intervals. Spearman correlation coefficients (ρ) and p-values are indicated above each plot. ** P < 0.01, *** P < 0.001, **** P < 0.0001.

We investigated which microbial enzymes are involved in processing PGE_2_-Acyl-GlcA. Previous studies suggest that two classes of enzymes, β-glucuronidases (GUS) and esterases, may hydrolyze acyl glucuronide conjugates^20^. To determine which class of enzyme, GUS or esterases, converts PGE_2_-Acyl-GlcA to PGE_2_, we tested the extent to which co-administration of D-saccharic acid 1,4-lactone (D-SL, a pan-GUS inhibitor) or phenylmethylsulfonyl fluoride (PMSF, an esterase inhibitor) inhibits microbial processing of PGE_2_-Acyl-GlcA (Fig. 3a)^20^. D-SL at concentrations ranging from 10 to 100 μM inhibited 91–97% of mouse fecal bacterial enzyme-mediated conversion of PGE_2_-Acyl-GlcA to PGE_2_. In contrast, treatment with PMSF, at concentrations up to 1,000 μM, had no effect (see 30 min- and 240 min-assay data in Fig. 3b and complete time-course data in Extended Data Fig. 16). These results suggest that microbial GUS enzymes, rather than esterases, play a key role in processing PGE_2_-Acyl-GlcA.

The human and mouse gut microbiome have been shown to contain hundreds of microbial GUS isoforms that act on diverse glucuronidated substrates^21^. Based on active-site architecture and/or cofactor binding, these enzymes have been classified into eight major clades^21^. To broadly represent this diversity, we generated a panel of purified gut microbial GUS enzymes encompassing all major clades^22^. Screening this panel for PGE_2_-Acyl-GlcA metabolism revealed that Loop-1 and FMN-binding GUS enzymes, notably *E. coli* GUS (EcGUS) enzyme, efficiently catalyzed the conversion of PGE_2_-Acyl-GlcA to PGE_2_ (Fig. 3c). These results support the conclusion that specific microbial GUS isoforms are responsible for processing PGE_2_-Acyl-GlcA.

To understand the structural basis for microbial GUS-mediated PGE_2_ reactivation, we generated molecular models of EcGUS with PGE_2_-Acyl-GlcA using Rosetta Molecular Modeling Suite. The simulation was conducted such that PGE_2_-Acyl-GlcA would be positioned within the active site with the glucuronic acid moiety oriented in a catalytically competent position relative to the established catalytic residues (Fig. 3d). Detailed analysis revealed that PGE_2_-Acyl-GlcA forms an extensive hydrogen bonding network within the EcGUS active site, involving both the catalytic residues (Glu409 and Glu502), conserved binding residues (Tyr466, Tyr470, Asn564 and Lys566) and residues predicted to stabilize the PGE_2_ tail, including Tyr466, Tyr470, and Gly362 (Fig. 3e). Critically, we identified that Gly362 in Loop-1 forms a unique 2.8 Å hydrogen bond with the hydrophobic PGE_2_ tail carboxyl group, a stabilizing interaction absent in Loop-2 family enzymes (Extended Data Fig. 17). To extend our analysis across bacterial GUS variants, we compiled structures of 18 enzymes, utilizing existing crystal structures for 8 enzymes and generating AlphaFold3 models for the remaining 10 enzymes lacking structural data (see Table S3). Structural analysis of 18 GUS variants confirmed that while conserved residues like Trp547 and His330 maintain consistent positions, non-conserved residues such as Asp164 and Arg560 are absent in low-activity variants (Fig. 3f). Additionally, statistical comparative analysis across 18 bacterial GUS variants between Rosetta-calculated structural features and experimentally measured PGE_2_ production demonstrated that observed activity increases with enzyme-product interface interactions, particularly the change in polar solvent-accessible surface area (dSASA_polar; ρ = −0.69, P = 0.003) (Table S4). Loop-1 family enzymes, such as EcGUS, exhibit lower polar surface burial and weaker electrostatic interactions with the product compared to Loop-2 structural family indicating these are critical biophysical features for enzyme activity (13.36 ± 2.80 vs. 4.53 ± 4.63 pmol PGE_2_ formed, P = 0.004). These relationships are further illustrated by scatter plot analysis, where Loop-1 enzymes consistently occupy favorable regions of each structural parameter space (Fig. 3g). These computational insights provide a biophysical basis for how a subset of bacterial GUS enzymes convert inactive PGE2-Acyl-GlcA back to active PGE_2_. These features may be useful in future efforts in rapid identification of microbiome studies in which novel microbial GUS enzymes are identified and their potential to drive increased colonic PG levels.

## Multiple PGs metabolized by UGT and GUS

Beyond PGE_2_, we have shown that colonic concentrations of other PGs are also regulated by gut microbiota (Fig. 1). We investigated whether other PGs, such as PGB_2_, PGE_3_, TXB_2_, and 15-keto PGE_2_, are also subject to sequential metabolism by host UGT and microbial GUS enzymes. LC-HRMS analysis supports that intestinal microsome converts each of these PGs into a single predominant glucuronide conjugate via a UGT-dependent manner. Moreover, the glucuronide conjugate was almost completely degraded following incubation with mouse fecal bacteria or EcGUS enzyme (Extended Data Fig. 18). Together, these findings suggest that similar to PGE_2_, other PGs are also metabolized by intestinal UGT enzymes to form PG glucuronides, which are subsequently processed by gut microbial GUS enzymes.

## GUS regulates gut PG levels and effects

We hypothesize that microbial GUS metabolism plays a critical role in regulating colonic PG levels. To test this hypothesis, we first explored a pharmacological approach using inhibitors of microbial GUS enzymes. We examined several well-established microbial GUS inhibitors, including UNC10201652 and GUS-IN-1 (N-[(1,2-dihydro-6,8-dimethyl-2-oxo-3-quinolinyl)methyl]-N′-(4-ethoxyphenyl)-N-(2-hydroxyethyl)thiourea)^22–24^. Previous studies have demonstrated that these compounds selectively inhibit microbial GUS enzymes without affecting mammalian GUS activity; deficiency of human GUS results in Sly Syndrome, a potentially fatal lysosomal storage disease^24^. However, neither compound at a dose range of 10–100 μM affected fecal bacterial enzyme–mediated processing of PGE_2_-Acyl-GlcA (Extended Data Fig. 19). PGE_2_-Acyl-GlcA is considerably larger than the substrates examined previously for efficacy with these inhibitors^22–24^; thus, these inhibitors may not effectively compete for substrate binding and thereby can’t inhibit the processing of PGE_2_-Acyl-GlcA.

**Fig 4.**
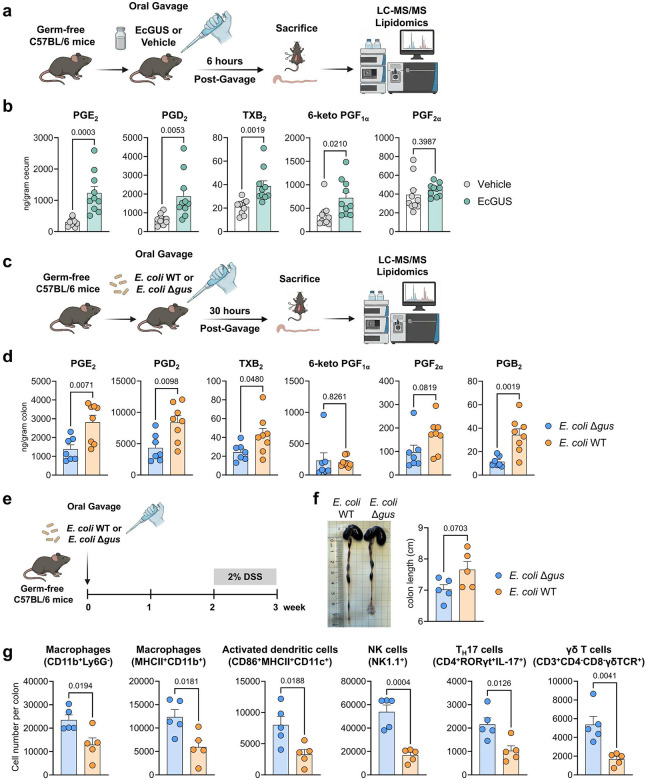
Microbial GUS metabolism plays a critical role in regulating gut PG levels and their effects. (**a**) GF C57BL/6 mice were orally treated with vehicle (PBS) or purified *E. coli* β-GUS (EcGUS) protein, then gut concentrations of PGs were measured. (**b**) Cecal PG concentrations were significantly increased in EcGUS-treated GF mice compared with vehicle–treated GF mice (n = 10 mice per group). (**c**) GF C57BL/6 mice were monocolonized with either an *E. coli* wild-type (WT) strain or an *E. coli* Δ*gus* strain via oral gavage, then gut concentrations of PGs were measured. (**d**) Compared with GF mice monocolonized with *E. coli* Δ*gus*, those monocolonized with *E. coli* WT exhibited significantly higher colonic PG levels (n = 7 mice for *E. coli* Δ*gus* and n = 8 mice for *E. coli* WT). (**e**) GF C57BL/6 mice were monocolonized with either *E. coli* WT or *E. coli* Δ*gus* via oral gavage, then stimulated with DSS to induce colitis. (**f**) The mice monocolonized with *E. coli* WT exhibited increased colon length, though the effects are not statistically significant (n = 5 mice per group). (**g**) The mice monocolonized with *E. coli* WT exhibited reduced infiltration of immune cells in the colon (n = 5 mice per group). Data are presented as mean ± SEM.

We next employed a strategy by orally administering purified GUS enzyme to GF mice, followed by measurement of PG levels in gut tissues (see the experimental scheme in Fig. 4a). We have shown that the EcGUS enzyme is highly effective to process PGE_2_-Acyl-GlcA and other PG glucuronides (Fig. 3 and Extended Data Fig. 18). In addition, previous animal studies have shown that oral administration of EcGUS enzyme modulated the pharmacokinetics of several drugs by enhancing their colonic de-glucuronidation, supporting the stability of EcGUS in the gastrointestinal tract and demonstrating the feasibility of using EcGUS administration in mice to study microbial GUS metabolism^25,26^. Therefore, we used EcGUS as an experimental probe to determine the roles of gut microbial GUS in regulating colonic PG levels. LC-MS/MS analysis showed that compared to vehicle (PBS)-treated GF mice, the cecal concentrations of PGs, including PGE_2_, PGD_2_, TXB_2_, and 6-keto PGF_1α_, were significantly increased in EcGUS-treated GF mice (Fig. 4b). Notably, the cecal concentration of PGE_2_ was 305.3 ± 39.5 (mean ± SEM) ng/g in PBS-treated GF mice versus 1234.0 ± 207.1 ng/g in EcGUS-treated GF mice (P = 0.0003), suggesting that EcGUS treatment causes ~4-fold increase of gut level of PGE_2_ (Fig. 4b). PGF_2α_ also showed a similar trend, though the effect was not statistically significant (P = 0.3987) (Fig. 4b). Other PGs, such as PGB_2_ or PGE_3_, were not detected, likely due to their low abundance in tissues (raw concentration data for each individual mouse are provided in the supplemental Excel file). Overall, this result supports a critical role of microbial GUS enzyme in regulating gut PG levels.

To further test our hypothesis, we used an alternative strategy of monocolonizing GF mice with either *E. coli* wild-type (WT) or a *gus* deletion (Δ*gus*) strain, then measured gut levels of PGs (see the scheme of the experiment in Fig. 4c). To validate this approach, we first characterized the two strains *in vitro*. Genome sequencing (Extended Data Fig. 20a) and colony PCR (Extended Data Fig. 20b) demonstrate successful deletion of the *gus* gene in the mutant strain. The WT and Δ*gus* strains exhibited comparable growth kinetics *in vitro* (Extended Data Fig. 20c). As expected, the *E. coli* Δ*gus* strain displayed a markedly reduced capacity to process PGE_2_-Acyl-GlcA compared with the WT strain *in vitro* (Extended Data Fig. 20d). Together, these results support that it is feasible to use *E. coli* WT *vs*. Δ*gus* to study the role of bacterial GUS in PG metabolism. Next, we found that compared to monocolonized mice with the WT strain, monocolonized mice with the Δ*gus* strain showed significantly lower levels of PGs, including PGE_2_, PGD_2_, PGB_2_, and TXB_2_ (P < 0.05), in gut tissues (Fig. 4d). For instance, the colonic concentration of PGE_2_ was 1381.7 ± 235.2 (mean ± SEM) ng/g in monocolonized mice with *E. coli* Δ*gus* versus 2819.3 ± 366.5 ng/g in monocolonized mice with *E. coli* WT (P = 0.0071) (Fig. 4d). In addition, PGF_2α_ also showed a similar trend, though the effect was not statistically significant (P = 0.0819) (Fig. 4b, raw concentration data for each individual mouse are provided in the supplemental Excel file). These findings further support a critical role for the microbial GUS pathway in regulating colonic PG levels.

Having shown that GF mice monocolonized with *E. coli* WT exhibit higher colonic PG levels than those monocolonized with *the* Δ*gus* strain (Fig. 4d), we next compared the development of PG-dependent disease in these two groups. Colonic PGs regulate a broad range of physiological and pathological processes^1,27^. Notably, they play a key role in maintaining gut barrier integrity, thereby protecting against dextran sodium sulfate (DSS)–induced epithelial injury and subsequent disease development^6,28^. Therefore, we compared the DSS-induced disorders in monocolonized mice with either *E. coli* WT or *E. coli* Δ*gus* (see the scheme of the experiment in Fig. 4e). Compared to DSS-treated monocolonized mice with the WT strain, mice colonized with the Δ*gus* strain exhibited shorter colon length (P = 0.0703; Fig. 4f) and increased colonic infiltration of immune cells including macrophages (CD11b^+^Ly6G^−^ MHCII^+^), activated dendritic cells (CD86^+^MHCII^+^CD11c^+^), NK cells (NK1.1^+^), T_H_17 cells (CD4^+^RORγt^+^IL-17^+^), and γδ T cells (CD3^+^CD4-CD8-γδTCR^+^) (P < 0.05; Fig. 4g). These results demonstrate that microbial GUS attenuated DSS-induced disease development, consistent with prior reports describing protective effects of colonic PGs in DSS colitis^6,28^. Together, these findings further support the microbial GUS pathway’s critical role in regulating gut PG levels and their biological effects.

## GUS drives PG reactivation

We compared the biological activity of PGE_2_-Acyl-GlcA with that of PGE_2_. PGE_2_ exerts its effects by binding to and activating its G protein-coupled receptors, PTGER1, PTGER2, PTGER3, and PTGER4^1,5^. We found that PGE_2_-Acyl-GlcA exhibited significantly reduced biological activity in activating all four receptors compared to PGE_2_. Notably, the potency of PGE_2_-Acyl-GlcA in activating PTGER4, the most abundantly expressed PGE_2_ receptor in the intestines of both mice and humans^4^, was approximately 60-fold lower than that of PGE_2_, with EC_50_ values of ~13 nM for PGE_2_ and ~840 nM for PGE_2_-Acyl-GlcA (Extended Data Fig. 21). These findings indicate that PGE_2_-Acyl-GlcA has markedly reduced biological activity compared to PGE_2_. Overall, our results suggest that intestinal UGT-catalyzed conversion of PGE_2_ to PGE_2_-Acyl-GlcA leads to metabolic inactivation of PGE_2_, while the microbial GUS metabolism reactivates PGE_2_.

We explored the potential implications of PGE_2_ to PGE_2_-Acyl-GlcA conversion in human health. First, we identified specific UGT isoforms that catalyze the conversion of PGE_2_ to PGE_2_-Acyl-GlcA. We screened a panel of purified human UGT enzymes and found that UGT2B isoforms, including UGT2B7 and UGT2B17, catalyzed the conversion of PGE_2_ to PGE_2_-Acyl-GlcA, whereas UGT1A isoforms exhibited weak or no activity (Extended Data Fig. 22a–b). Previous studies have shown that UGT2B17 is the most abundant UGT in human colon tissue, accounting for approximately 70% of total colonic UGT mRNA expression; in contrast, UGT2B7 expression in human colon tissue is low. Moreover, the UGT2B17 expression is low in other tissues, such as liver and kidney^29^. Together, these findings support an important role for UGT2B isoforms, particularly UGT2B17, in colonic glucuronidation of PGE_2_. Next, since PGs, notably PGE_2_, regulate various physiological and pathological processes in the gut, including colorectal cancer^1,27^, we analyzed the expression of UGT2B7 and UGT2B17 in colorectal cancer using The Cancer Genome Atlas (TCGA). UGT2B17 expression was significantly reduced in tumors from patients with colorectal cancer compared with matched adjacent non-tumor tissues (~95% reduction, P < 0.00001). UGT2B7 showed a similar downward trend, though the difference was not statistically significant (Extended Data Fig. 22c). Collectively, these data indicate that UGT2B17 plays a major role in the colonic conversion of PGE_2_ to PGE_2_-Acyl-GlcA, leading to metabolic inactivation of PGE_2_. Reduced UGT2B17 expression in human colorectal cancer may therefore impair PGE_2_ inactivation, leading to elevated PGE_2_ levels and more aggressive disease progression.

## Discussion

First discovered in the 1930s^8–10^ and recognized with the Nobel Prize in Physiology or Medicine in 1982^11^, the PG biosynthetic pathways have been extensively studied and are considered well defined^1^. Here, our findings reveal a previously unrecognized, microbiota-dependent metabolic pathway, expanding the conceptual framework of lipid mediator biology. Our data support a model in which, following its production in host gut tissues, PGE_2_ is further metabolized by host UGT enzymes—particularly UGT2B isoforms—to selectively form an acyl glucuronide conjugate, PGE_2_-Acyl-GlcA. This conjugate is secreted into the gut lumen (supported by the detection of PGE_2_-Acyl-GlcA in colon and cecum digesta of GF mice, see Fig. 2j), where it interacts with gut microbiota and is hydrolyzed by microbial enzymes to regenerate free PGE_2_, thereby elevating colonic PGE_2_ levels. Using a combination of approaches including enzyme inhibitors (pan inhibitors of GUS or esterase enzymes), screening of microbial GUS isoforms, and molecular modeling, our findings support that specific microbial GUS enzymes process PGE2-Acyl-GlcA through unique structural motifs. Many gut bacteria express GUS enzymes that catalyze the hydrolysis of glucuronide conjugates of xenobiotic compounds, leading to the release of glucuronic acid, which can be utilized by gut bacteria as a carbon source, as well as the generation of free aglycones that are often biologically active^21–24^. Here our data support that microbial GUS enzymes are also involved in the metabolism of glucuronides of endogenous lipids, such as PGE_2_. Beyond PGE_2_, other PGs undergo similar sequential metabolism by intestinal UGTs and microbial GUS enzymes (see the proposed model in Extended Data Fig. 23). Functional experiments, including administration of purified GUS enzyme and mono-colonization of GF mice with WT or *Δgus* bacteria, establish that microbial GUS play a critical role in regulating gut PG levels (Fig. 4). Collectively, these findings establish that gut microbes directly participate in PG metabolism and regulate gut PG levels.

Colonic PGs regulate a wide spectrum of physiological and pathological processes^1,4,6,27,28^. They are essential for maintaining epithelial barrier integrity by protecting against acute epithelial injury and promoting tissue repair following damage^1,6,27,28^. Accordingly, inhibition or genetic deletion of COXs, which lowers PG production, exacerbates DSS–induced colonic injury and disease progression^28^; whereas elevating tissue PG levels enhances mucosal regeneration and protects against DSS-induced pathology^6^. Beyond barrier maintenance, PGs also regulate intestinal inflammation, immune responses, and colorectal tumorigenesis^1,4,27^. Using mono-colonization of WT or Δ*gus* bacteria in GF mice, we demonstrate that microbial GUS regulate colonic PG levels and modulates downstream PG-dependent biological responses (Fig. 4). Together, these findings support a potential mechanism by which the gut microbiota influence intestinal physiology and pathology through modulation of PG signaling.

Given the central roles of PGs in gut homeostasis and disease, colonic PG pathways represent promising therapeutic targets^1,27^. For example, epidemiological studies and clinical trials have consistently shown that COX inhibitors, such as aspirin and other NSAIDs, significantly reduce colorectal cancer risk^27^. However, because COX enzymes are also expressed in various other tissues where they regulate essential physiological functions^1^, the broad suppression of COX activity by these inhibitors could lead to serious adverse effects, limiting their widespread use^7,30^. In contrast, PG-reactivating GUS enzymes are exclusively localized to the colon^21^, these microbial enzymes may represent promising therapeutic targets for selectively modulating colonic PG levels without affecting other tissues. Supporting this concept, our LC-MS/MS analyses of PGE_2_ across multiple tissues from GF and SPF mice demonstrate that the gut microbiota selectively modulate PGE_2_ concentrations in gut tissues (Extended Data Fig. 2). Targeting PG-reactivating GUS enzymes may therefore enable tissue-selective therapeutic modulation of PG signaling, offering potential strategies for preventing or treating colorectal cancer and inflammatory gastrointestinal diseases while minimizing systemic toxicity.

## Supplementary Material

1

This is a list of supplementary files associated with this preprint. Click to download.

- Rawdata.xlsx

## Data Availability

Source data are provided with this paper. All other data supporting the findings of this study are included in the Article and its Supplementary Information. Complete datasets underlying the figures are provided in a supplementary Excel file, and the raw mass spectrometry data are provided in a supplementary PDF document.

## References

[1] Funk C. D. Prostaglandins and leukotrienes: advances in eicosanoid biology. Science 294, 1871–1875 (2001). 10.1126/science.294.5548.1871 11729303

[2] Ricciotti E. & FitzGerald G. A. Prostaglandins and inflammation. Arterioscler Thromb Vasc Biol 31, 986–1000 (2011). 10.1161/atvbaha.110.207449 21508345 PMC3081099

[3] Nomura D. K. Endocannabinoid hydrolysis generates brain prostaglandins that promote neuroinflammation. Science 334, 809–813 (2011). 10.1126/science.1209200 22021672 PMC3249428

[4] Crittenden S. Prostaglandin E2 promotes intestinal inflammation via inhibiting microbiota-dependent regulatory T cells. Sci Adv 7 (2021). 10.1126/sciadv.abd7954 PMC7880593 33579710

[5] Yao C. Prostaglandin E2-EP4 signaling promotes immune inflammation through Th1 cell differentiation and Th17 cell expansion. Nat Med 15, 633–640 (2009). 10.1038/nm.1968 19465928

[6] Zhang Y. Inhibition of the prostaglandin-degrading enzyme 15-PGDH potentiates tissue regeneration. Science 348, aaa2340 (2015). 10.1126/science.aaa2340 26068857 PMC4481126

[7] Cheng Y. Role of prostacyclin in the cardiovascular response to thromboxane A2. Science 296, 539–541 (2002). 10.1126/science.1068711 11964481

[8] Kurzrok R. & Lieb C. C. Biochemical studies of human semen. II. The action of semen on the human uterus. Proceedings of the Society for Experimental Biology and Medicine 28, 268–272 (1930). 10.3181/00379727-28-5265

[9] Goldblatt M. W. Properties of human seminal plasma. J Physiol 84, 208–218 (1935). 10.1113/jphysiol.1935.sp003269 16994667 PMC1394818

[10] Euler U. S. Über die Spezifische Blutdrucksenkende Substanz des Menschlichen Prostata- und Samenblasensekretes. Klinische Wochenschrift 14, 1182–1183 (1935). 10.1007/bf01778029

[11] Oates J. A. The 1982 Nobel Prize in Physiology or Medicine. Science 218, 765–768 (1982). 10.1126/science.6753151 6753151

[12] Lynch S. V. & Pedersen O. The Human Intestinal Microbiome in Health and Disease. N Engl J Med 375, 2369–2379 (2016). 10.1056/NEJMra1600266 27974040

[13] Meech R. The UDP-Glycosyltransferase (UGT) Superfamily: New Members, New Functions, and Novel Paradigms. Physiol Rev 99, 1153–1222 (2019). 10.1152/physrev.00058.2017 30724669

[14] Gamage N. Human sulfotransferases and their role in chemical metabolism. Toxicol Sci 90, 5–22 (2006). 10.1093/toxsci/kfj061 16322073

[15] Little J. M. Glucuronidation of oxidized fatty acids and prostaglandins B1 and E2 by human hepatic and recombinant UDP-glucuronosyltransferases. J Lipid Res 45, 1694–1703 (2004). 10.1194/jlr.M400103-JLR200 15231852

[16] Hankin J. A., Wheelan P. & Murphy R. C. Identification of novel metabolites of prostaglandin E2 formed by isolated rat hepatocytes. Arch Biochem Biophys 340, 317–330 (1997). 10.1006/abbi.1997.9921 9143337

[17] Stehle R. G. & Oesterling T. O. Stability of prostaglandin E1 and dinoprostone (prostaglandin E2) under strongly acidic and basic conditions. J Pharm Sci 66, 1590–1595 (1977). 10.1002/jps.2600661123 21282

[18] Corey E. J., Schaaf T. K., Huber W., Koelliker U. & Weinshenker N. M. Total synthesis of prostaglandins F2-alpha and E2 as the naturally occurring forms. J Am Chem Soc 92, 397–398 (1970). 10.1021/ja00705a609 5411057

[19] Zhang F. Concise, scalable and enantioselective total synthesis of prostaglandins. Nat Chem 13, 692–697 (2021). 10.1038/s41557-021-00706-1 34045714

[20] Ikuta H. Species differences in liver microsomal hydrolysis of acyl glucuronide in humans and rats. Xenobiotica 52, 653–660 (2022). 10.1080/00498254.2022.2131484 36190839

[21] Pollet R. M. An Atlas of β-Glucuronidases in the Human Intestinal Microbiome. Structure 25, 967–977.e965 (2017). 10.1016/j.str.2017.05.003 28578872 PMC5533298

[22] Zhang J. Microbial enzymes induce colitis by reactivating triclosan in the mouse gastrointestinal tract. Nat Commun 13, 136 (2022). 10.1038/s41467-021-27762-y 35013263 PMC8748916

[23] Wallace B. D. Alleviating cancer drug toxicity by inhibiting a bacterial enzyme. Science 330, 831–835 (2010). 10.1126/science.1191175 21051639 PMC3110694

[24] Pellock S. J. Gut Microbial beta-Glucuronidase Inhibition via Catalytic Cycle Interception. ACS Cent Sci 4, 868–879 (2018). 10.1021/acscentsci.8b00239 30062115 PMC6062831

[25] Yip L. Y. The liver-gut microbiota axis modulates hepatotoxicity of tacrine in the rat. Hepatology 67, 282–295 (2018). 10.1002/hep.29327 28646502

[26] Eichenbaum G. Oral coadministration of β-glucuronidase to increase exposure of extensively glucuronidated drugs that undergo enterohepatic recirculation. J Pharm Sci 101, 2545–2556 (2012). 10.1002/jps.23113 22473491

[27] Gupta R. A. & Dubois R. N. Colorectal cancer prevention and treatment by inhibition of cyclooxygenase-2. Nat Rev Cancer 1, 11–21 (2001). 10.1038/35094017 11900248

[28] Morteau O. Impaired mucosal defense to acute colonic injury in mice lacking cyclooxygenase-1 or cyclooxygenase-2. J Clin Invest 105, 469–478 (2000). 10.1172/jci6899 10683376 PMC289156

[29] Zhou L., Montalvo A. D., Collins J. M. & Wang D. Quantitative analysis of the UDP-glucuronosyltransferase transcriptome in human tissues. Pharmacol Res Perspect 11, e01154 (2023). 10.1002/prp2.1154 37983911 PMC10659769

[30] Fitzgerald G. A. Coxibs and cardiovascular disease. N Engl J Med 351, 1709–1711 (2004). 10.1056/NEJMp048288 15470192

