# Supplementary material for: Gut Microbiota Mediate the Metabolism of Colonic Prostaglandins
Source: Res Sq. 2026 Feb 20:rs.3.rs-8856024. Preprint. [Version 1] doi: 10.21203/rs.3.rs-8856024/v1 (PMC12934979; doi:10.21203/rs.3.rs-8856024/v1)

List of Supplemental Information

1. Supplemental Materials and Methods
2. Supplemental Figures (Fig. S1-S23)
3. Supplemental Tables (Table S1-S9)
4. Raw spectrometry data from LC-HRMS and TSQ LC–MS/MS experiments

Note: Complete datasets underlying the figures are provided in a separate Excel file included as supplementary material.

Materials and Methods

Chemicals

PGs, including PGE_2, PGB_2, PGE_3, TXB_2, and 15-keto PGE_2 (≥98% purity), and the isotope-labeled PGE_2-d_4 (9-oxo-11α,15S-dihydroxy-prosta-5Z,13E-dien-1-oic-3,3,4,4-d_4 acid; ≥99% deuterated forms, d_1-d_4) were purchased from Cayman Chemical (Ann Arbor, MI). D-saccharic acid 1,4-lactone monohydrate (≥98.0%), UDP-glucuronic acid, 3'-phospho-adenosyl-5'-phosphosulfate, and phenylmethanesulfonyl fluoride were purchased from Sigma-Aldrich (Burlington, MA). β-Glucuronidase-IN-1(N-[(1,2-dihydro-6,8-dimethyl-2-oxo-3-quinolinyl)methyl]-N′-(4-ethoxyphenyl)-N-(2-hydroxyethyl)thiourea), >98% purity, was purchased from MedChemExpress (Monmouth Junction, NJ). UNC10201652 is kindly provided by Dr. Redinbo's laboratory at UNC-Chapel Hill. The authentic standard of PGE_2-Acyl-GlcA (≥96.7% purity) was generated by Hypha Discovery at Abingdon UK (see detailed preparation and purification methods below).

Animal experiments

The animal experiments were conducted following protocols approved by the Institutional Animal Care and Use Committees of Massachusetts Host-Microbiome Center at the Brigham and Women's Hospital (BWH) and Weill Cornell Medicine.

Animal experiment 1: Analysis of lipid metabolites in the tissues of SPF mice *vs.* GF mice

GF and SPF mouse experiments were conducted at the Massachusetts Host–Microbiome Center at Brigham and Women's Hospital (BWH). SPF C57BL/6 mice (4 weeks old) were obtained from Taconic Biosciences. Upon arrival, mice were housed in a standard SPF facility with access to autoclaved tap water and fed autoclaved LabDiet 5021 (LabDiet) for two weeks. GF C57BL/6 mice were obtained directly from the Massachusetts Host–Microbiome Center and maintained in GF isolators, where they received the same autoclaved tap water and autoclaved LabDiet 5021.

At 6 weeks of age, all mice were euthanized, and the entire gastrointestinal tract was excised and immediately frozen in liquid nitrogen. Frozen colon tissues were thawed on ice and gently flushed with ice-cold PBS to remove residual luminal contents. Tissues were then briefly blotted with a paper towel to remove excess moisture and debris, ensuring accurate tissue weight measurements for LC-MS/MS analysis.

In an independent cohort of GF and SPF mice, multiple tissues, including colon, cecum, lung, heart, spleen, small intestine, stomach, and kidney, were collected for LC-MS/MS quantification of PGE_2 concentrations.

Animal experiment 2: Oral gavage of *E. coli* GUS enzyme in GF mice

E. coli GUS (EcGUS) enzyme (Type IX-A, lyophilized powder, 1,000,000–5,000,000 units/g protein; Sigma-Aldrich, Cat # G7396) was reconstituted in sterile phosphate-buffered saline (PBS) to a concentration of 15,000 U/mL. The GUS enzyme solution was orally gavaged into 6-week-old C57BL/6 GF mice at a dose of 90 U per gram of body weight. After 6 h, the mice were euthanized to collect tissues for analysis.

Animal experiment 3: Mono-colonization of GF mice with *E. coli* WT and Δ*gus*

E. coli WT and Δ*gus* strains were kindly provided by Dr. Yi Wang's laboratory at UC Davis. Pure cultures of *E. coli* WT or Δ*gus* were grown overnight in Luria–Bertani (LB) broth, then 0.2 mL of overnight culture containing 5.5-7.5 × 10^8 CFU/mL was gavaged to 6-week-old C57BL/6 GF mice. After 30 h, the mice were euthanized for analysis.

To verify mono-colonization and exclude contamination, bacterial purity was assessed before and after gavage. An aliquot (100 µL) of overnight culture was serially diluted and plated on tryptic soy agar supplemented with 5% sheep blood for aerobic culture and on Brucella agar for anaerobic culture. Colonies recovered under aerobic conditions were morphologically consistent with *E. coli*. Gram staining of cultures and aerobic plates revealed a single Gram-negative, rod-shaped organism. In addition, bacterial identity was confirmed using VITEK MS MALDI–TOF mass spectrometry.

Animal experiment 4: Effects of mono-colonization of germ-free mice with *E. coli* WT or Δ*gus* in a DSS-induced colitis model

Pure cultures of *E. coli* WT or Δ*gus* were grown overnight in LB broth. Six-week-old GF C57BL/6 mice were monocolonized by oral gavage with 10^8 CFU in 100 μL PBS of the overnight culture. Following a 2-week colonization period, colitis was induced by administering 2% dextran sulfate sodium (DSS; MW 36–50 kDa; MP Biomedicals, Solon, OH) in the drinking water for 7 days. Mice were euthanized at the end of DSS treatment for analysis [1].

LC-MS/MS lipidomics analysis of lipid metabolites in the colon of SPF mice vs. GF mice
The LC-MS/MS lipidomics analysis of lipid metabolites in the colons of SPF and GF mice was performed at NIH/NIEHS, as described [2]. Colon samples were weighed and placed in tubes containing 9 μL of Hanks' Balanced Salt Solution (HBSS) per mg of colon tissue. Each tube was then supplemented with 1 μL of 1 mM 1-trifluoromethoxyphenyl-3-(1-propionylpiperidin-4-yl) urea (TPPU) in methanol. Tissues were homogenized using a Tissuelyzer II (Qiagen, Hilden, Germany) at 30 Hz for 10 minutes. To extract free lipid metabolites from the colon tissue, 10 μL of an internal standard mix (11,12-EET-d_{11}, 11,12-DHET-d_{11}, PGE2-d_9, ARA-d_9, and 15-HETE-d_8) was added to 100 μL of the homogenate (equivalent to 10 mg of colon tissue), followed by liquid–liquid extraction with 600 μL ethyl acetate. The organic phase was collected and dried under vacuum centrifugation. Extracted lipid metabolites were reconstituted in 50 μL of 30% ethanol and analyzed by LC-MS/MS using an Ultimate 3000 UHPLC system (Thermo Fisher Scientific) coupled to a TSQ Quantiva triple quadrupole mass spectrometer (Thermo Fisher Scientific, Waltham, MA). Chromatographic separation was performed on an XSelect CSH C18 column (2.1 × 50 mm, 3.5 μm particle size; Waters, Milford, MA) maintained at 50 °C.

Mobile phase A consisted of water/acetonitrile/acetic acid (85:15:0.1, v/v/v), and mobile phase B consisted of acetonitrile/methanol/acetic acid (70:30:0.1, v/v/v) [3]. The flow rate was 400 μL/min, and gradient elution was applied as follows: 20% B at 0 min; linear increase to 40% B from 0–5 min; to 55% B from 5–7 min; to 64% B from 7–13 min. From 13–19 min, the column was washed with 100% B at a flow rate of 550 μL/min, followed by re-equilibration. The injection volume was 10 μL.

Mass spectrometric detection was performed in negative ion electrospray ionization mode using multiple reaction monitoring (MRM). Data acquisition and quantification were performed using TraceFinder software (v4.1, Thermo Fisher Scientific). Quantification was achieved by comparison of analyte peak areas to calibration curves generated using authentic lipid standards (Cayman Chemical). Lipid standards were stored in 100% ethanol under argon and used within one year of purchase. Analyte retention times, monitored transitions, internal standards, and calibration curve ranges are provided in **Table S5**.

Liquid Chromatography-High Resolution Mass Spectrometry (LC-HRMS) analysis of PG glucuronides
We employed *in vitro* enzymatic reactions to generate PG glucuronides (as described below) and the formation of PG glucuronides was monitored using an ultra-high-performance liquid chromatography (UHPLC) system (Thermo Fisher Scientific) coupled with a Q Exactive™ Hybrid Quadrupole-Orbitrap™ Mass Spectrometer (Thermo Fisher Scientific). The mass spectrometer was operated in either selected ion monitoring (SIM) or parallel reaction monitoring (PRM) mode for target detection. The mass resolution was set to 35,000, with an isolation window of 1.6 m/z. Chromatographic separation was performed on an Acquity HSS T3 column (1.8 µm, 2.1 × 100 mm, Waters). The mobile phases consisted of (A) water with 0.1% formic acid (v/v) and (B) acetonitrile with 0.1% formic acid (v/v). The elution gradient was as follows: 2% B from 0 to 1 min, a linear increase to 100% B from 1 to 19 min, held at 100% B for 3 min (19-22 min), followed by re-equilibration at 2% B for 3 min.

UGT enzymatic assays using mouse microsomes
Mouse liver microsomes (pooled CD-1 male; Sigma-Aldrich) and mouse intestinal microsomes (pooled CD-1 male; IPHASE Biosciences, North Wales, PA) were resuspended in 100 mM HEPES (4-(2-hydroxyethyl)-1-piperazineethanesulfonic acid) buffer (Thermo Fisher Scientific) at a final protein concentration of 1 mg/mL and used for *in vitro* UGT activity assays. PGE_2 and other PGs were dissolved in 100% methanol to prepare 10 mM stock solutions. UDP-glucuronic acid (UDP-GlcA), $MgCl_2$ and D-saccharic acid 1,4-lactone monohydrate (D-SL) were dissolved in Milli-Q H_2O to generate 100× stock solutions. The final reaction mixture (500 μL) contained 5 mM $MgCl_2$, 5 mM D-SL, 4 mM UDP-GlcA, 100 μg microsomal protein, and 100 μM PG substrate. All components were diluted in assay buffer (100 mM HEPES, pH 7.4, containing 1mg/mL Brij 58). Reactions were incubated at 37 °C for 60 min. The reactions were quenched by adding three volumes of ice-cold

methanol, followed by centrifugation at 6,000 × g for 10 minutes at 4 °C. The resulting supernatant was concentrated using a vacuum centrifuge concentrator. The dried residue was then reconstituted in 100 µL methanol, followed by centrifugation at 10,000 × g for 10 minutes, and the final supernatant was subjected to LC-HRMS analysis.

Effects of gut bacteria or EcGUS enzyme on microsome-generated PG glucuronides
Fresh mouse feces (~200 mg) were collected and homogenized in 1.2 mL of PBS containing 0.05% L-cysteine. The homogenate was centrifuged at 900 × g for 5 minutes at 4 °C, and the supernatant containing viable gut bacteria was collected. The bacterial suspension (200 µL) was incubated with reconstituted PG glucuronide conjugates (10 µL), generated using microsome reactions (as described above), in a total volume of 400 µL PBS at 37 °C for 2 hours. To evaluate the effect of EcGUS, EcGUS (Sigma-Aldrich) was resuspended in PBS to a concentration of 40,000 U/mL. Five microliters (200 U) of EcGUS were used in place of the bacterial suspension. The reactions were quenched by the addition of three volumes of ice-cold methanol, and the sample was subsequently processed following the same procedure described above for LC-HRMS analysis.

SULT enzymatic assays using liver S9 or intestinal microsome
Mouse liver S9 fraction was prepared from mouse liver homogenate (0.5 g/mL) by centrifugation at 9000 × g for 20 min in 0.1 M Tris-HCl (pH 7.5). Mouse intestinal microsomes (pooled from CD-1 male mice) were purchased from IPHASE Biosciences. The reaction mixture (500 µL) contained 5 mM $MgCl_2$, 100 µM 3′-phosphoadenosine-5′-phosphosulfate (PAPS), 100 µg intestinal microsomal protein or 500 µg liver S9 protein, and 100 µM PGE_2. All components were diluted in assay buffer (100 mM HEPES, pH 7.4, containing 1 mg/mL Brij 58). Reactions were incubated at 37 °C for 60 min. To terminate the reaction, three volumes of ice-cold methanol were added to quench enzyme activity. The mixture was centrifuged at 6000 × g for 10 min at 4 °C, and the resulting supernatant was concentrated using a vacuum concentrator. The dried residue was reconstituted in methanol, centrifuged at 10,000 × g for 10 min, and the final supernatant was subjected to LC-HRMS.

Biotransformation screening for production of compound 1 (PGE_2-Acyl-GlcA)
Authentic standard of compound 1 (PGE_2-Acyl-GlcA, ≥96.7% purity) was generated by Hypha Discovery (Abingdon, UK) using a biotransformation approach. To identify an optimal production system, a panel of 24 microbial strains and 17 liver S9 fractions was screened for their ability to convert PGE_2 to compound 1 (PGE_2-Acyl-GlcA).

For microbial screening, bacterial strains were cultured in shake flasks containing proprietary M3G medium at 27 °C with agitation (200 rpm) for 48 h. A fungal strain was revived on malt extract agar plates and subsequently cultured in C95 medium under identical conditions. Aliquots (2.5 mL) of each culture were transferred to MicroBioreactor (EnzyScreen BV) wells pre-dosed with PGE_2 (final concentration, 0.1 mg/mL; stock solution, 25 mg/mL in DMSO). Reactions were incubated at 27 °C with shaking at 300 rpm. Samples were collected after 22 h, quenched with an equal volume of acetonitrile, and clarified by centrifugation. Supernatants were evaporated to dryness, reconstituted in methanol, and analyzed by LC-HRMS. Formation of PGE_2 glucuronide conjugates was assessed semi-quantitatively based on peak area comparisons.

For liver S9 screening, reactions were performed in 96-well plates (final volume, 100 µL) containing HEPES buffer (130 mM, pH 7.5), $MgCl_2$ (35 mM), liver S9 fraction (2–20 mg/mL protein), and UDP-glucuronic acid (40 mM). Reactions were initiated by addition of PGE_2 (final concentration, 0.1 mg/mL) and incubated at 37 °C with shaking (150 rpm) for 18 h. Samples were processed as described above.

From these screening experiments, a proprietary genetically engineered recombinant *Streptomyces* strain (HD038) was identified as the most efficient system for producing compound 1.

Scaled-up microbial fermentation to produce compound 1 (PGE_2-Acyl-GlcA)
For scale-up fermentation to prepare compound 1, seed cultures of strain HD038 were prepared from cryopreserved stock cultures inoculated into proprietary M3G medium and incubated at 27 °C with shaking (200 RPM). Production cultures were inoculated with 3-day old seed culture and further incubated under identical conditions. After 24 h, cultures (1 L total volume) were dosed with PGE_2 to a final concentration of 100

mg/L and incubated for an additional 48 h in the same manner. Fermentations were then harvested promptly after reaction was confirmed by daily analysis of time-course samples and extracted as described below.

Purification of compound 1 (PGE_2-Acyl-GlcA)
The combined fermentation broth was centrifuged to separate supernatant and biomass. Biomass pellets were extracted twice with acetonitrile, combined and concentrated under reduced pressure for combination with the broth supernatant. The combined supernatant and biomass extract were loaded onto Diaion HP20 resin preconditioned in water, washed with water, and eluted stepwise with increasing concentrations of acetonitrile (10–100%). Fractions containing compound 1 were pooled and further purified by reversed-phase chromatography using C18, RP8, and diphenyl stationary phases with aqueous acetonitrile gradients containing formic acid. The final product was isolated as an off-white resinous solid following lyophilization. The purity of compound 1 was assessed by LC-MS (Waters Acquity UPLC QDA), LC-UV (Waters Acquity UPLC PDA (UV-Vis detection)), and evaporative light-scattering detection (ELSD) using a Waters Acquity UPLC system equipped with a BEH Shield RP18 column (1.7 µm, 2.1 × 50 mm). Mobile phases consisted of water (A) and acetonitrile (B), both containing 0.1% formic acid. Full gradient conditions: Gradient (A%/B%): t=0 mins: 98/2 to 2/98 over 2.4 mins, held for 0.4 minute then returned to 98/2 over 0.05 min (t=2.85 mins) and equilibrated for a further 0.15 mins (t=3 mins) all at a flow-rate of 1.0 mL/min.

Structural elucidation of compound 1 (PGE_2-Acyl-GlcA) by NMR spectroscopy
The structure of compound 1 was confirmed by NMR spectroscopy and mass spectrometry. One- and two-dimensional NMR spectra (^1H, ^{13}C, COSY, HSQC, HMBC, and NOESY) were acquired on a 600 MHz spectrometer equipped with a cryo-enhanced probe. Spectra were processed using MestReNova software.

Quantification of PGE_2 and PGE_2-Acyl-GlcA by triple quadrupole LC–MS/MS (TSQ LC–MS/MS)
PGE_2 and PGE_2–Acyl-GlcA in mouse tissues were quantified using an TSQ LC-MS/MS method. Briefly, approximately 30 mg of tissue was homogenized in 400 µL of cold methanol at −4 °C for 5 min using a Servicebio KZ-1-Fp homogenizer. The homogenates were frozen at −20 °C for 2 h to precipitate proteins, then thawed and centrifuged at 10,000 × g for 10 min at −4 °C. The resulting supernatants were collected and subjected to TSQ LC–MS/MS analysis using a UHPLC system coupled to a TSQ Altis mass spectrometer (Thermo Fisher Scientific).

Chromatographic conditions were identical to those used for the LC-HRMS analysis. Briefly, analytes were separated on an Acquity HSS T3 column (1.8 µm, 2.1 × 100 mm; Waters). Mobile phase A consisted of water containing 0.1% (v/v) formic acid, and mobile phase B consisted of acetonitrile containing 0.1% (v/v) formic acid. The elution gradient was as follows: 2% B from 0–1 min; a linear increase to 100% B from 1–19 min; a hold at 100% B from 19–22 min; followed by re-equilibration at 2% B for 3 min.

The TSQ Altis mass spectrometer was operated in selected reaction monitoring (SRM) mode for targeted detection of PGE_2 and PGE_2-Acyl-GlcA. The monitored transitions and collision energies (CE) were as follows: PGE_2, m/z 351.2 → 189.1 (18 eV) and 351.2 → 271.2 (15 eV); PGE_2–Acyl-GlcA, m/z 527.2 → 113.0 (28 eV) and 527.2 → 271.2 (30 eV). Quantification was performed using authentic PGE_2 and PGE_2-Acyl-GlcA standards to generate calibration curves.

PGE_2-Acyl-GlcA stability
PGE_2-Acyl-GlcA (final concentration = 50 nM) was dissolved in phosphate buffer at different pH values (pH 5.0–8.0) and incubated at 37 °C to assess its stability. At t = 0-24 h, samples were analyzed by LC–MS/MS (Agilent 1200SL HPLC system coupled to a 4000 QTRAP mass spectrometer; Gemini 5 µm C18 column, 30 × 2 mm, Phenomenex, Torrance, CA) to quantify the remaining concentration of PGE_2-Acyl-GlcA.

Effects of cultured bacteria on processing PGE_2-Acyl-GlcA *in vitro*
The mouse fecal bacterial suspension was inoculated into MRS broth (Thermo Fisher Scientific) and cultured overnight at 37 °C in an anaerobic chamber (Vinyl Anaerobic Chamber Type A, Coy Labs, Grass Lake, MI) under an atmosphere of 85% N_2, 10% CO_2, and 5% H_2. When the OD_{600nm} reached ~0.5, 2 mL of cultures were centrifuged at 10,000 × g for 3 min, and the bacterial pellet was washed once with 500 µL sterile PBS. After a second centrifugation (10,000 × g, 3 min), the pellet was resuspended in 300 µL PBS to obtain the cultured bacterial suspension. PGE_2-Acyl-GlcA was resuspended in 100% methanol to a concentration of 10 µM. To

test the effects of cultured bacteria on processing PGE_2-Acyl-GlcA, 20 µL of cultured bacterial suspension, 1 µL PGE_2-Acyl-GlcA (50 nM final), and 179 µL PBS were mixed, then incubated at 37 °C. The reaction was quenched by addition of three-volume ice-cold methanol, centrifuged at 10,000 × g for 10 min, and the reaction was monitored by the formation of PGE_2 using LC–MS/MS. Formation of PGE_2 was quantified by LC-MS/MS using the same analytical method as that employed for PGE_2-Acyl-GlcA stability assays. PGE_2 concentrations were determined using calibration curves generated with authentic standards.

Effects of lysed bacterial enzymes on processing PGE_2-Acyl-GlcA *in vitro*
Bacterial cultures were prepared as described above. Lysozyme (10 µL, 50 mg/mL) was added to the resuspended bacterial suspension (300 µL) and incubated at 37 °C for 30 min. The suspension was then sonicated on ice (10 cycles of 10-s pulses at high intensity, with 30 s intervals on ice). Samples were centrifuged at 10,000 × g for 20 min at 4 °C, and the supernatant containing bacterial enzymes was collected and used in place of the intact bacterial suspension for enzymatic assays, as described above.

Screening of microbial GUS Enzymes for processing PGE_2-Acyl-GlcA *in vitro*
Purified microbial GUS enzymes were provided by Dr. Redinbo's laboratory at UNC-Chapel Hill. For each reaction, 5 µL of enzyme solution (1 nM final) and 35 µL of assay buffer (50 mM HEPES, 50 mM NaCl, pH = 6.5) were added to a 96-well PCR plate and incubated at 37 °C for 5 minutes. PGE_2-Acyl-GlcA was resuspended in 100% methanol to a concentration of 5 µM. The reaction was initiated by adding 10 µL of PGE_2-Acyl-GlcA (1 µM final) to each well. After a 2-hour incubation, 50 µL of methanol was added to quench the reaction. The mixture was then centrifuged at 13,000 × g for 10 minutes. The resulting supernatant was subjected to LC-MS/MS analysis to quantify PGE_2 formation.

Effects of GUS and esterase inhibitors on microbial enzymatic activity
Mouse fecal bacteria were cultured and lysed as described above. To assess the effects of GUS and esterase inhibitors, enzymatic assays were performed in the presence of 5 µL of the following inhibitors: the pan-GUS inhibitor D-saccharic acid 1,4-lactone monohydrate (D-SL; dissolved in Milli-Q H_2O; final concentration, 10–100 µM), the microbial GUS inhibitor UNC10201652 (dissolved in DMSO:H_2O, 1:9, v/v, final concentration, 10–100 µM), β-Glucuronidase-IN-1 (dissolved in DMSO; final concentration, 10–100 µM), or the esterase inhibitor phenylmethylsulfonyl fluoride (PMSF; dissolved in methanol; final concentration, 10–1000 µM). All other assay conditions remained as previously described.

Screening of human UGT enzymes that convert PGE_2 to PGE_2-Acyl-GlcA
Human UGT enzymes (UGT1A1, UGT1A3, UGT1A4, UGT1A6, UGT1A7, UGT1A8, UGT1A9, UGT1A10, UGT2B7, and UGT2B17) were purchased from BioIVT (Westbury, NY) and resuspended in 100 mM HEPES buffer at a final protein concentration of 1 mg/mL. PGE_2 was dissolved in methanol to prepare a 10 mM stock solution. The reaction mixture (500 µL) contained 5 mM $MgCl_2$, 5 mM D-SL, 4 mM UDP-GlcA, 100 µg recombinant UGT enzyme, and 100 µM PGE_2. All components were diluted in assay buffer (100 mM HEPES, pH 7.4, containing 1 mg/mL Brij 58). Reactions were incubated at 37 °C for 60 minutes. Reactions were terminated by adding three volumes of ice-cold methanol. Samples were centrifuged, dried by vacuum centrifugation, and reconstituted in methanol for TSQ LC–MS/MS analysis.

Quantitative real-time reverse transcription PCR (qRT-PCR)
Total RNA was isolated from the colon tissues using TRIzol (Thermo Fisher Scientific) according to the manufacturer's instructions. Complementary DNA (cDNA) was synthesized using the GoScript Reverse Transcriptase Kit (Promega, Madison, WI). qRT-PCR was performed using the CFX Duet Real-Time PCR System (Bio-Rad Laboratories, Hercules, CA) with iTaq Universal SYBR Green Supermix (Bio-Rad Laboratories). Primer sequences used for amplification are listed in **Table S6**. Glyceraldehyde-3-phosphate dehydrogenase (*Gapdh*) served as the internal control for normalization.

Structural analysis and molecular docking of GUS
We analyzed 18 bacterial GUS variants, and focused on structural differences between Loop-1 (E. coli, C. perfringens, S. agalactiae, E. eligens) and Loop-2 (P. copri, B. angulatum) enzyme families. Eight structures were obtained from the RCSB Protein Data Bank (E. coli: 3K46, C. perfringens: 4JKM, S. agalactiae: 4JKL, E. eligens: 6BJQ, B. fragilis: 3CMG, F. saccharivorans: 6NCY, B. dorei: 6ED1, F. prausnitzii: 6U7I). Ten structures were modeled using AlphaFold3 [4] (R. gnavus, Eubacterium sp., B. massiliensis, P. copri, R. hominis

1, R. hominis 2, B. angulatum, P. merdae, B. ovatus, Parabacteroides MSP). For AlphaFold3 structures, 10 models were generated per enzyme and the model with the highest confidence score was selected for docking studies.

Molecular docking was performed using Rosetta modeling software (version 2024.09, release 371) [5-7] with PGE_2-Acyl-GlcA as a substrate. Ligand conformer libraries were generated using Spartan to sample substrate flexibility. The binding site was defined using catalytic residues and conserved binding determinants identified from sequence conservation analysis [8]. The docking protocol proceeded through initial placement, rigid-body transformation using HighResDocker, and final minimization using FinalMinimizer. We generated 1000 poses per enzyme and selected the top 20 models based on lowest interface energy and constraint satisfaction filters. Convergence was assessed by calculating average pairwise heavy-atom RMSD of the glucuronic acid moiety among top 20 models using E. eligens GUS (6BJQ) as reference. Structural features including polar solvent-accessible surface area burial (dSASA_polar), electrostatic energy (fa_elec), and solvation energy (fa_sol) were calculated for each model using Rosetta's energy function.

Sequence Conservation and Statistical Analysis
Multiple sequence alignments were performed using Clustal Omega [9] to analyze Loop-1 conservation patterns. Sequence logos were generated using WebLogo [10] to visualize conservation at binding site segments and Loop-1 regions. Correlation analysis between Rosetta energy features and experimental activity (PGE_2 product formation in pmol) was performed using Python with Biopython [11]. Spearman rank correlation coefficients and p-values were calculated to assess statistical significance. Features with $p < 0.05$ were considered significant predictors of catalytic activity.

Construction of *E. coli* Δ*gus*
E. coli DH5α and MG1655 strains were used in this study and cultured in LB medium (1% (w/v) tryptone, 0.5% (w/v) yeast extract, and 1% (w/v) NaCl) at 30 °C or 37 °C. When required, LB medium was supplemented with antibiotics (50 μg/mL kanamycin and 50 μg/mL streptomycin) and inducers (1 mM isopropyl β-D-1-thiogalactopyranoside (IPTG) and 10 mM L-arabinose). All strains, plasmids, and primers used in this study are listed in **Table S7** and **Table S8**. *E. coli* DH5α was used for plasmid construction.

Deletion of the *gus* gene in *E. coli* MG1655 was performed using a CRISPR–Cas9/λ-Red recombination–based strategy as previously described [12,13]. Plasmid p15ALacCas9, derived from our previous work, harbors an IPTG-inducible Cas9 expression cassette and an L-arabinose–inducible λ-Red recombination system. The *gus*-targeting plasmid pTarget-*gus* was constructed using primers YW1020 and YW1021. Plasmid p15ALacCas9 was first electroporated into *E. coli* MG1655, followed by induction of Cas9 and λ-Red expression and preparation of competent cells. Plasmid pTarget-*gus*, together with a linear DNA fragment containing homologous arms flanking the *gus* gene, was then co-electroporated into the competent cells. Recombinant strains were selected on LB agar plates and incubated statically at 30 °C overnight.

Correct editing of the *gus* locus was screened by colony PCR using primers YW1014 and YW1019 annealing to regions outside the homologous arms and further confirmed by DNA sequencing. Positive clones were subcultured in antibiotic-free LB medium at 42 °C to facilitate plasmid curing. Genetically stable *E. coli* strains were subsequently cultured at 37 °C with shaking at 200 rpm. Bacterial growth was monitored by measuring OD_{600nm} at indicated time points, and growth curves were generated accordingly. Phanta Max Master Mix (2×) and Green Taq Mix (2×) (Vazyme Biotechnology, Nanjing, China) were used for high-fidelity DNA amplification and colony PCR, respectively. Plasmid extraction, DNA fragment purification, and plasmid assembly were performed using commercial kits (Omega and Vazyme Biotechnology).

To assess the ability of *E. coli* strains to process PGE_2-Acyl-GlcA in vitro, *E. coli* WT and Δ*gus* strains were cultured overnight in LB medium. Bacterial cultures were normalized based on OD_{600nm}, collected by centrifugation, and washed twice with PBS. Cell pellets were resuspended in PBS and lysed by sonication on ice (10 cycles of 10-s pulses at high intensity, with 30-s intervals between pulses). Lysates were centrifuged at 10,000 × g for 20 min at 4 °C, and the resulting supernatants containing bacterial enzymes were collected for subsequent enzymatic assays, as described above. PGE_2-Acyl-GlcA was added at a final concentration of 250 nM.

Flow cytometry analysis of lamina propria cells
Lamina propria cells were isolated from mouse intestines as previously described with minor modifications [1]. Briefly, intestines were excised, cleared of residual fat tissue, cut longitudinally, and washed in ice-cold PBS. Epithelial cells were removed by incubation in HBSS containing 5 mM ethylenediaminetetraacetic acid (EDTA; Thermo Fisher Scientific), 1 mM dithiothreitol (DTT; Sigma-Aldrich), and 2% heat-inactivated fetal bovine serum (FBS) at 37 °C for 20 min with shaking. Intestines were washed twice in PBS by vortex, chopped into <2 mm pieces, and placed into beakers before enzymatic digestion in digestion buffer consisting of dispase (0.4 U/mL; Thermo Fisher Scientific), collagenase III (1 mg/mL; Worthington Biochemical, Lakewood, NJ), and DNase I (20 μg/mL; Sigma-Aldrich) in RPMI-1640 medium (Gibco, Waltham, MA) supplemented with 10% FBS for 45-60 min at 37 °C with stirring (>600 rpm). Leukocytes were enriched by centrifugation in a 40% Percoll gradient with RPMI + 10% FBS (GE Healthcare, Waukesha, WI) at 2,000 rpm for 20 min. Isolated cells were washed with cold PBS and blocked with Fc block in FACS buffer (PBS with 1% BSA) for 15 min. Cells were washed with FACS buffer and incubated with surface markers and viability dye for 20 min at 4 °C (antibodies used in this project are listed in **Table S9**). For intracellular markers, cells were fix and permeabilized using the eBioscience Foxp3/Transcription Factor Staining Set according to the manufacturer's protocol (eBioscience, San Diego, CA). Stained cells were subsequently washed with FACS buffer (Fluorescence-Activated Cell Sorting buffer) and data was acquired using the Cytek Aurora (Cytek Biosciences, Fremont, CA) and analyzed using FlowJo software (Becton Dickinson, Franklin Lakes, NJ).

Statical Analysis
Data are expressed as mean ± standard error of the mean (SEM). Statistical comparison of two groups was performed using Student's t-test and a comparison of more than two groups was performed using two-way ANOVA. $P < 0.05$ was considered statistically significant. The statistical analyses were performed using GraphPad Prism 10.0 statistical software (GraphPad Software).

Data availability
Source data are provided with this paper. All other data supporting the findings of this study are included in the Article and its Supplementary Information. Complete datasets underlying the figures are provided in the supplementary Excel files, and the raw mass spectrometry data are provided in supplementary PDF documents.

References

1 Sanidad, K. Z. *et al.* Maternal gut microbiome-induced IgG regulates neonatal gut microbiome and immunity. *Sci Immunol* **7**, eabh3816, doi:10.1126/sciimmunol.abh3816 (2022).

2 Edin, M. L. *et al.* Effects of sEH inhibition on the eicosanoid and cytokine storms in SARS-CoV-2-infected mice. *FASEB journal : official publication of the Federation of American Societies for Experimental Biology* **38**, e23692, doi:10.1096/fj.202302202RR (2024).

3 Edin, M. L. *et al.* Epoxide hydrolase 1 (EPHX1) hydrolyzes epoxyeicosanoids and impairs cardiac recovery after ischemia. *The Journal of biological chemistry* **293**, 3281-3292, doi:10.1074/jbc.RA117.000298 (2018).

4 Abramson, J. *et al.* Accurate structure prediction of biomolecular interactions with AlphaFold 3. *Nature* **630**, 493-500, doi:10.1038/s41586-024-07487-w (2024).

5 Lemmon, G. & Meiler, J. Rosetta Ligand docking with flexible XML protocols. *Methods in molecular biology (Clifton, N.J.)* **819**, 143-155, doi:10.1007/978-1-61779-465-0_10 (2012).

6 Davis, I. W. & Baker, D. RosettaLigand docking with full ligand and receptor flexibility. *Journal of molecular biology* **385**, 381-392, doi:10.1016/j.jmb.2008.11.010 (2009).

7 Meiler, J. & Baker, D. ROSETTALIGAND: protein-small molecule docking with full side-chain flexibility. *Proteins* **65**, 538-548, doi:10.1002/prot.21086 (2006).

8 Wallace, B. D. *et al.* Structure and Inhibition of Microbiome β-Glucuronidases Essential to the Alleviation of Cancer Drug Toxicity. *Chemistry & biology* **22**, 1238-1249, doi:10.1016/j.chembiol.2015.08.005 (2015).

9 Sievers, F. *et al.* Fast, scalable generation of high-quality protein multiple sequence alignments using Clustal Omega. *Molecular systems biology* **7**, 539, doi:10.1038/msb.2011.75 (2011).

10 Crooks, G. E., Hon, G., Chandonia, J. M. & Brenner, S. E. WebLogo: a sequence logo generator. *Genome research* **14**, 1188-1190, doi:10.1101/gr.849004 (2004).

11 Cock, P. J. *et al.* Biopython: freely available Python tools for computational molecular biology and bioinformatics. *Bioinformatics (Oxford, England)* **25**, 1422-1423, doi:10.1093/bioinformatics/btp163 (2009).

12 Sharan, S. K., Thomason, L. C., Kuznetsov, S. G. & Court, D. L. Recombineering: a homologous recombination-based method of genetic engineering. *Nature protocols* **4**, 206-223, doi:10.1038/nprot.2008.227 (2009).

13 Li, Q. *et al.* Improving the Editing Efficiency of CRISPR-Cas9 by Reducing the Generation of Escapers Based on the Surviving Mechanism. *ACS synthetic biology* **12**, 672-680, doi:10.1021/acssynbio.2c00619 (2023).

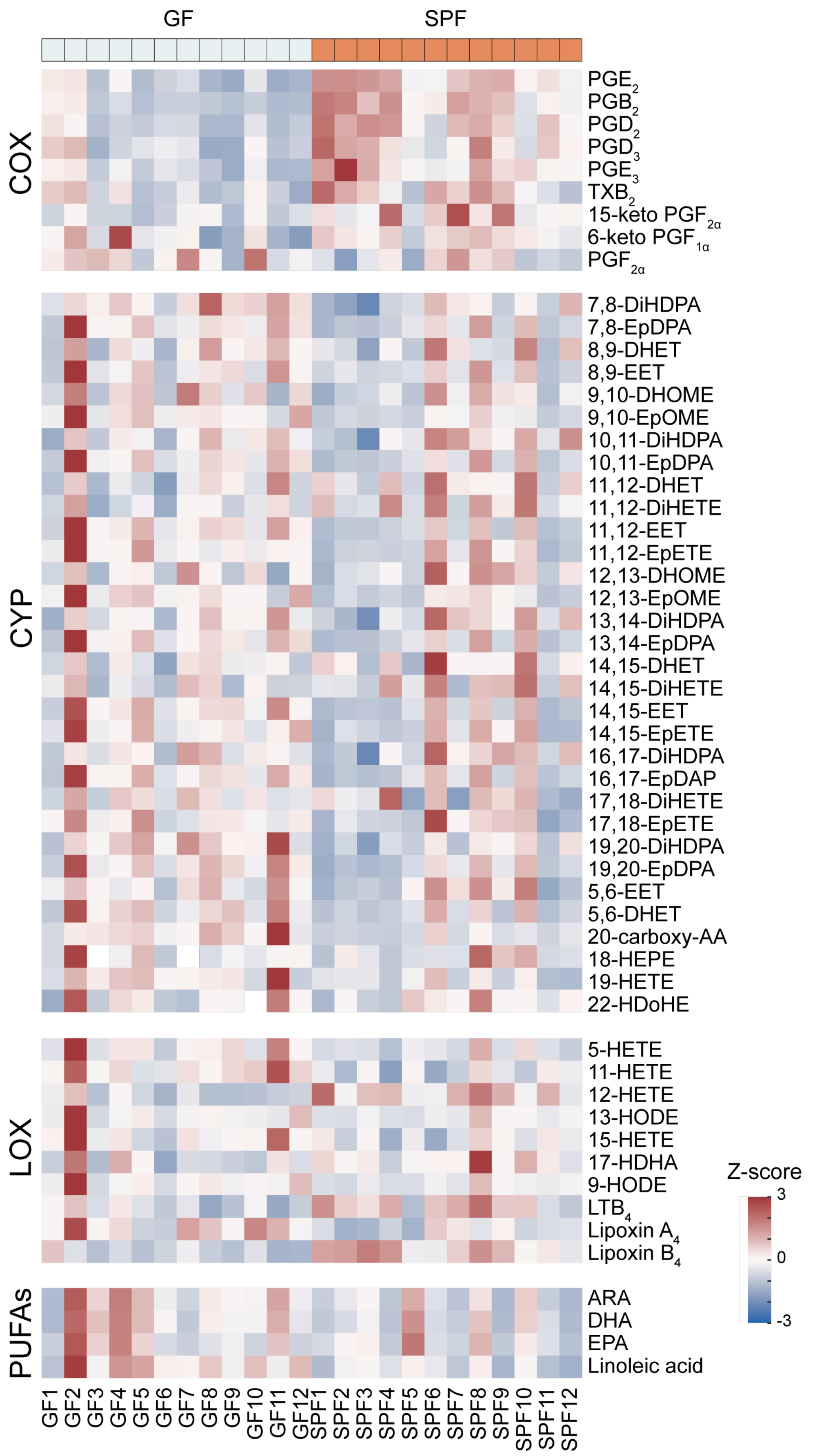

Extended Data Fig. 1 | Heatmap analysis of lipid metabolites in the colon tissues of conventional (specific pathogen-free or SPF) *vs.* germ-free (GF) mice. We used LC-MS/MS-based lipidomics to compare lipid metabolites in the colons of SPF mice and GF mice, and found that COX-derived prostaglandins (PGs)

are among the most markedly increased lipid metabolites in the colons of SPF mice compared to those of GF mice. **Note:** 5,6-EET (5,6-epoxyeicosatrienoic acid) is a chemically unstable metabolite that can undergo rapid cyclization; as a result, the measured concentration of 5,6-EET may not accurately reflect its true abundance in mouse tissues. n = 12 mice per group.

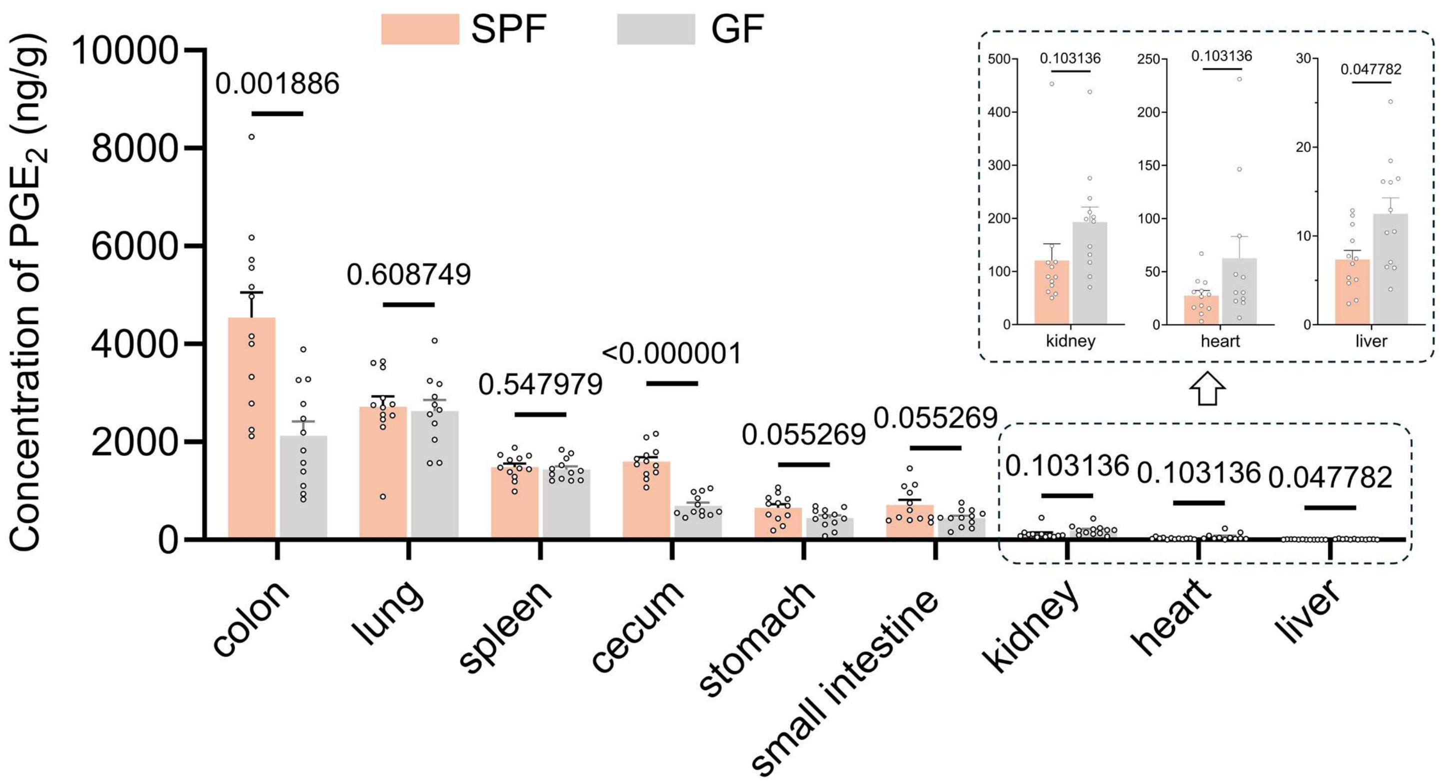

Extended Data Fig. 2 | Concentrations of PGE_2 in different organs from SPF and GF C57BL/6 mice. The results are expressed as mean ± standard error of the mean (SEM), n = 12 mice per group unless otherwise indicated in the figure. For lung and heart, n = 11 for GF mice due to sample unavailability. **Note:** The LC-MS/MS lipidomics analysis shown in Fig. 1d was performed using a UHPLC system coupled to a TSQ Quantiva tandem mass spectrometer at NIH/NIEHS, whereas the LC-MS/MS analysis in Extended Data Fig. 2 was conducted using a UHPLC system coupled to a TSQ Altis mass spectrometry system at HKBU. Because these analyses were performed in different laboratories using distinct protocols (e.g., sample preparation and standard curve construction) and two independent batches of GF and SPF C57BL/6 mice, the absolute PG concentrations measured in mouse tissues differed modestly between platforms (e.g., colonic PGE_2 concentrations in SPF mice were ~2,000 ng/g using the NIH/NIEHS method and ~4,000 ng/g using the HKBU method). Importantly, however, the direction and magnitude of change between GF and SPF mice were consistent across both analytical methods.

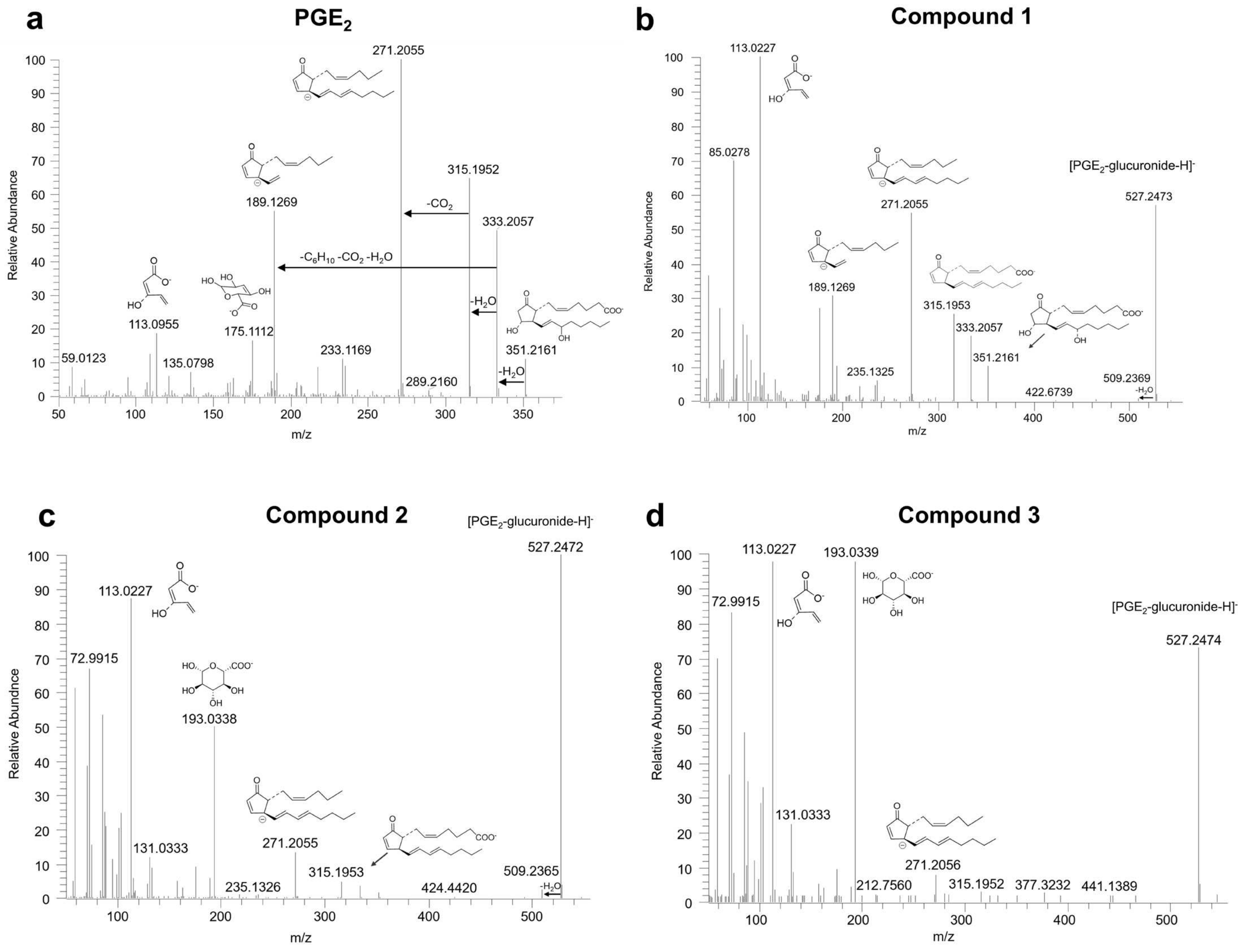

Extended Data Fig. 3 | Liver microsomes convert PGE_2 into three putative glucuronide conjugates. PGE_2 has three potential sites for glucuronidation (two -OH groups and a COOH- group), and therefore it could theoretically be converted into three glucuronide conjugates. MS/MS spectra and key ion fragments of PGE_2 (**a**), and its glucuronide conjugates including compound 1 (**b**), compound 2 (**c**), and compound 3 (**d**).

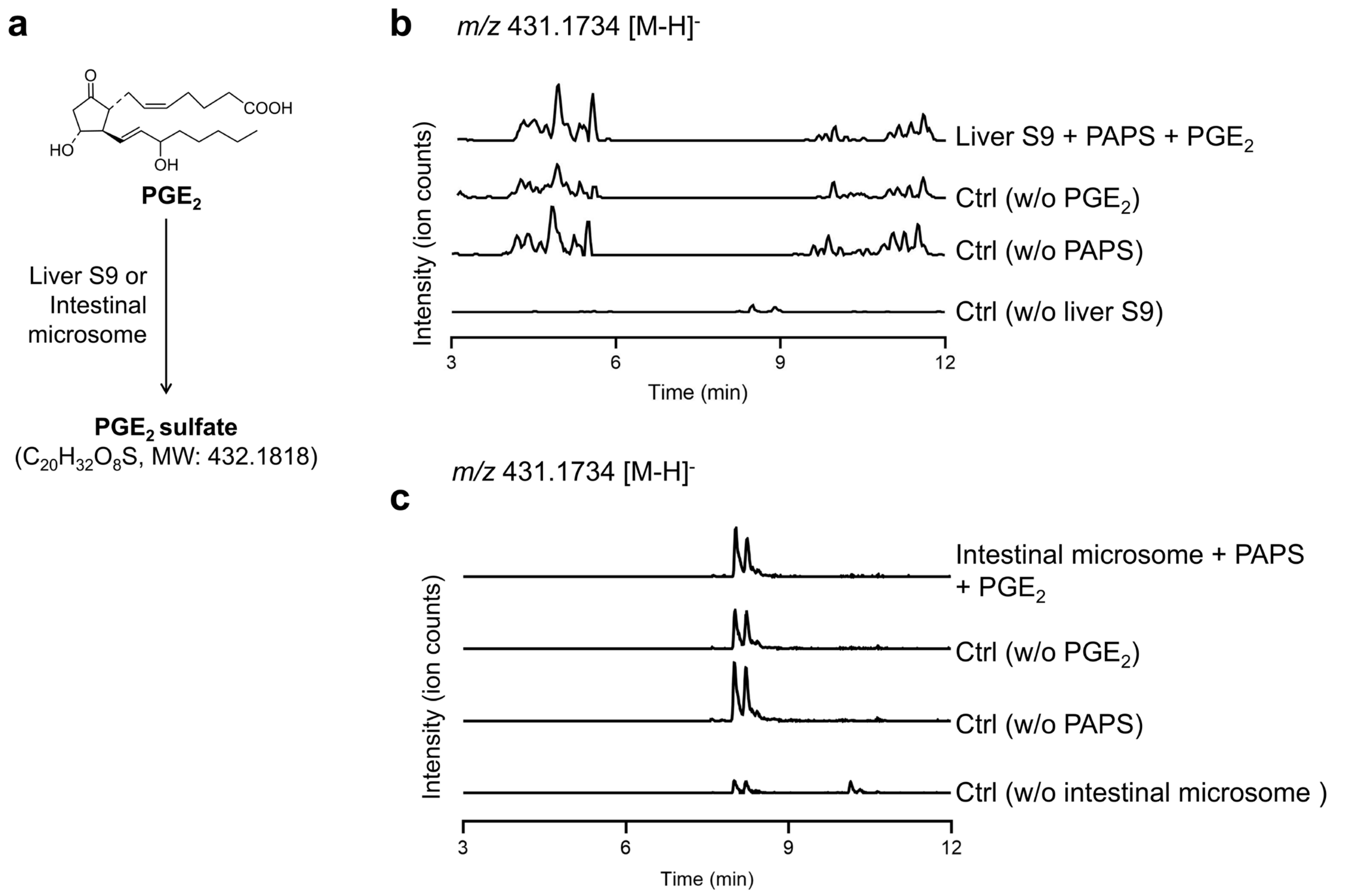

Extended Data Fig. 4 | PGE_2 is not metabolized by host SULT enzymes to form sulfate conjugates. (**a**) We incubated PGE_2 with liver or intestinal microsome, and 3′-phosphoadenosine-5′-phosphosulfate (PAPS, the cofactor for SULT enzymes), then used LC-HRMS to test the formation of potential PGE_2 sulfate conjugates. (**b-c**) LC-HRMS analysis suggests that liver S9 or intestinal microsome can't convert PGE_2 to its sulfate conjugates.

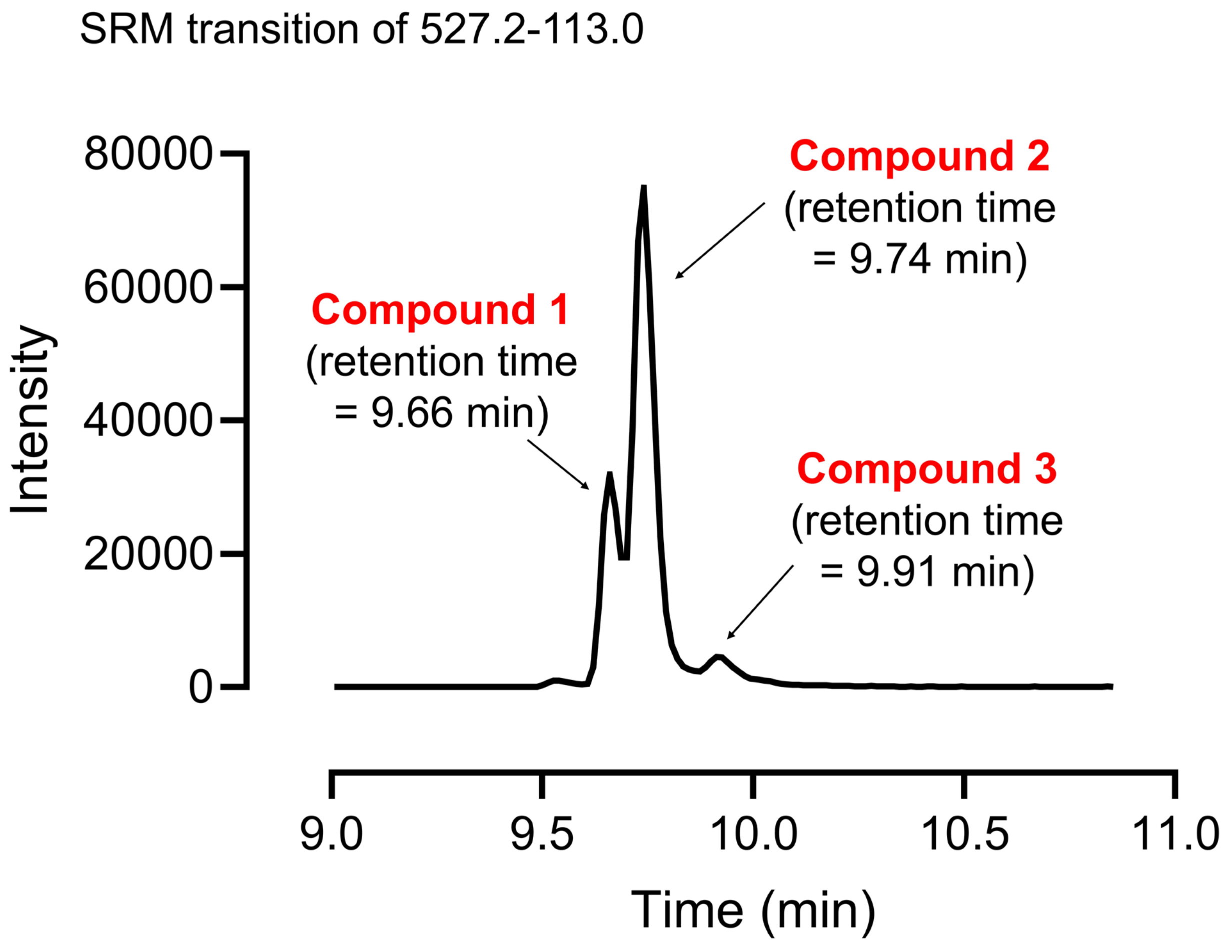

Extended Data Fig. 5 | TSQ LC–MS/MS method for the analysis of PGE_2-glucuronide conjugates. We established a TSQ LC–MS/MS method to analyze the three PGE_2-glucuronide conjugates (compounds 1, 2, and 3) that are generated by the liver microsomal reaction.

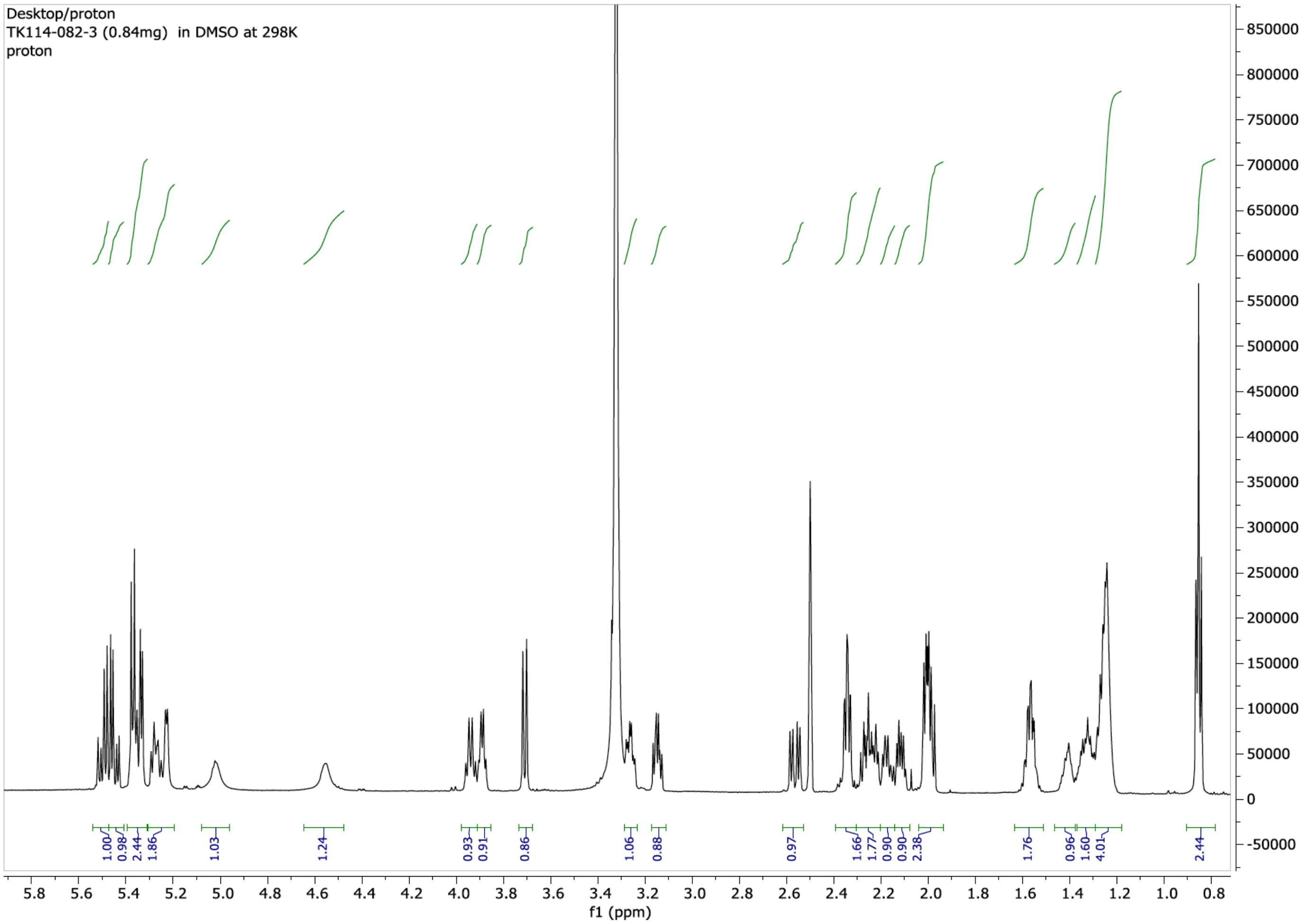

Extended Data Fig. 6 | ^{1}H NMR spectrum of PGE$_2$-glucuronide compound 1 (PGE$_2$-Acyl-GlcA) in DMSO-d_6.

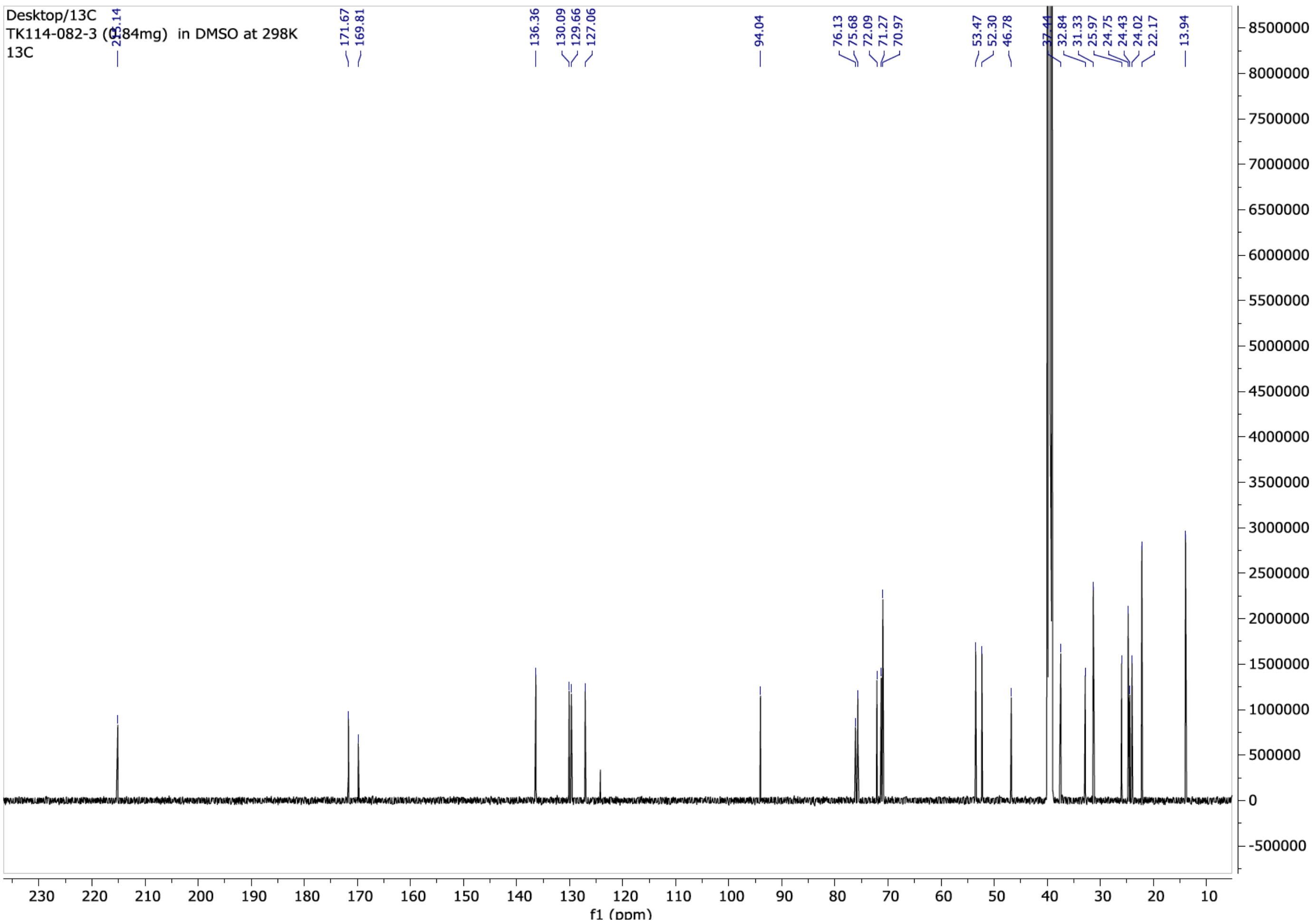

Extended Data Fig. 7 | ^{13}C NMR spectrum of compound 1 (PGE_2-Acyl-GlcA) in DMSO-d_6.

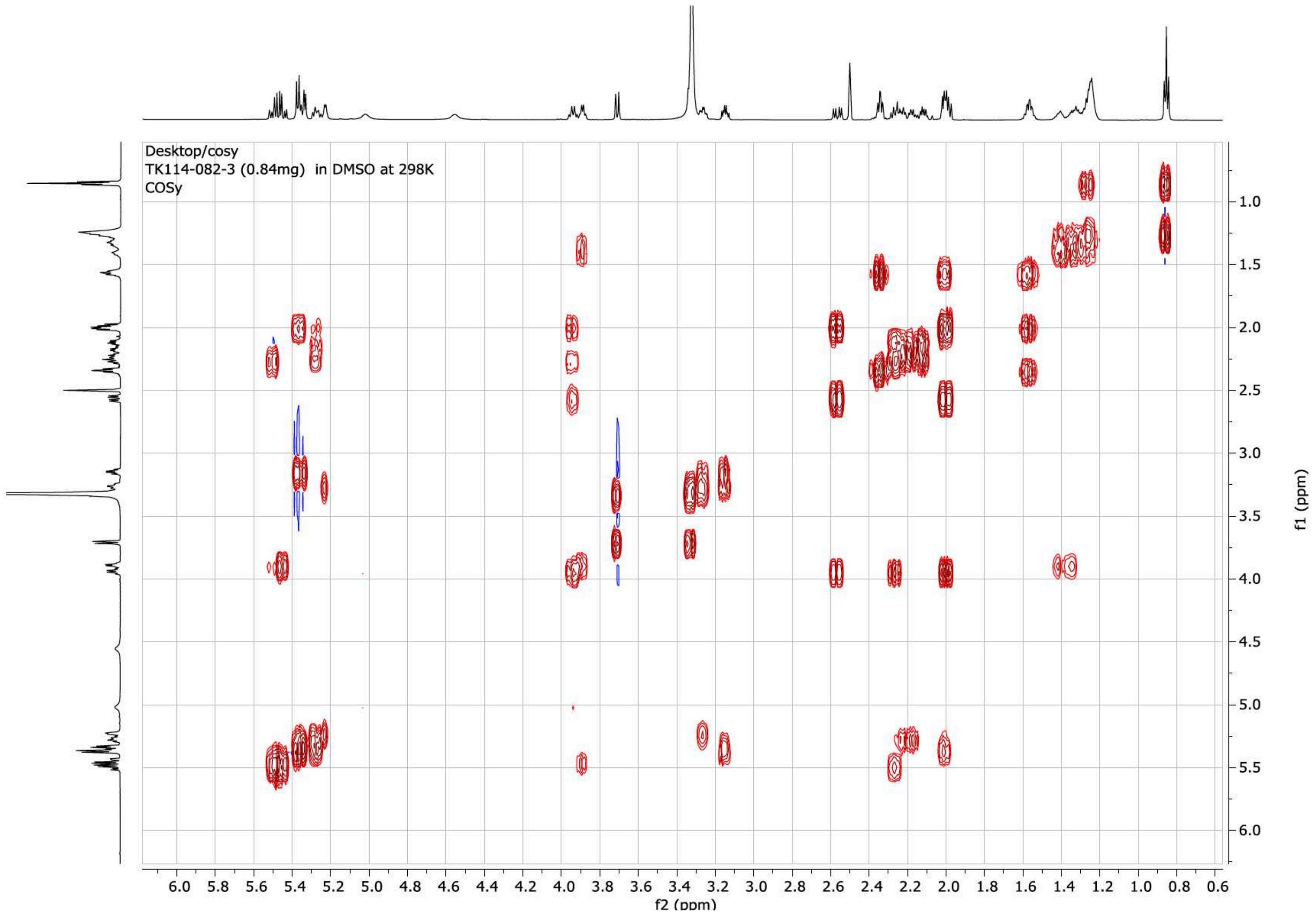

Extended Data Fig. 8 | COSY NMR spectrum of compound 1 (PGE_2-Acyl-GlcA) in DMSO-d_6.

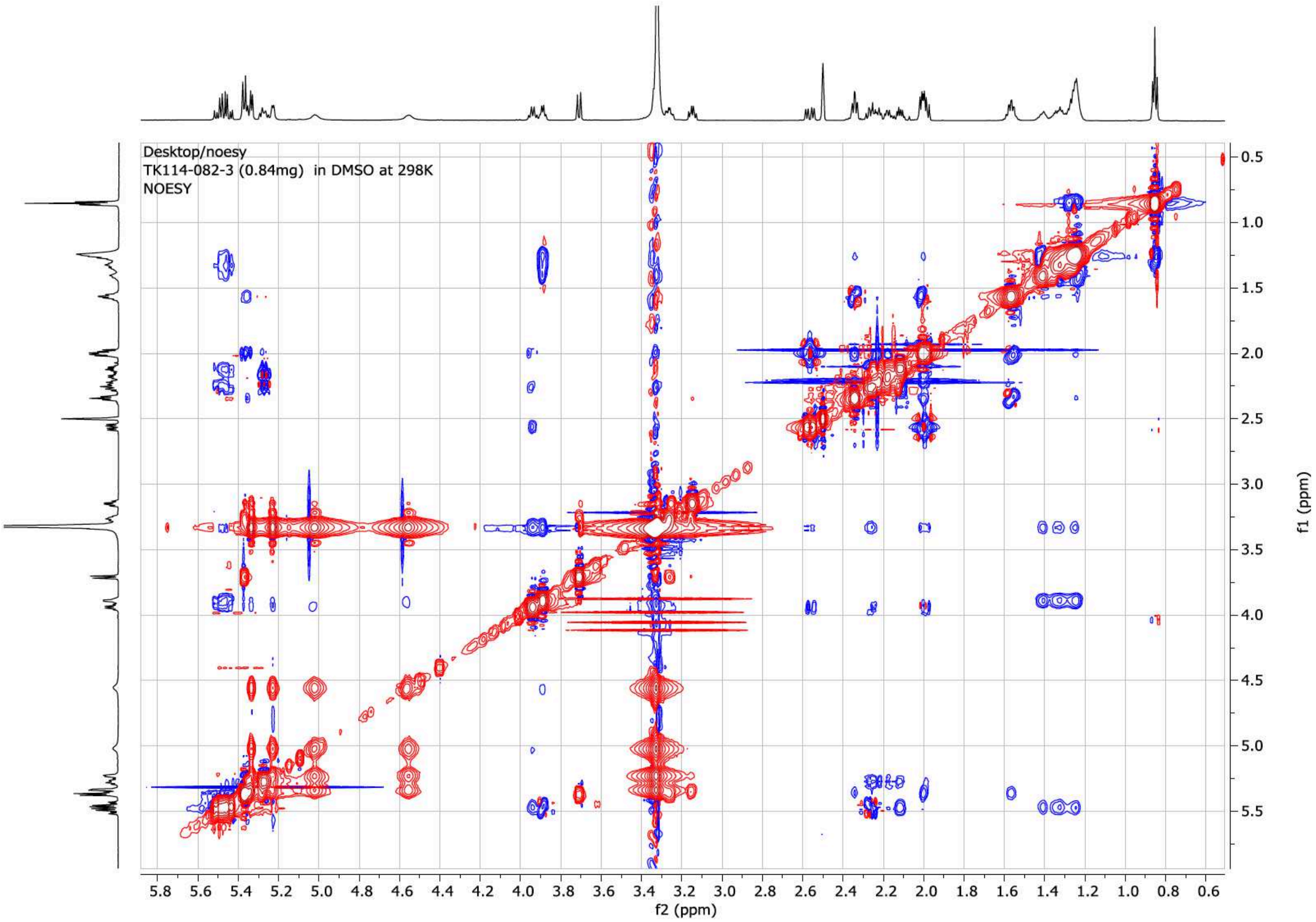

Extended Data Fig. 9 | NOESY NMR spectrum of compound 1 (PGE_2-Acyl-GlcA) in DMSO-d_6.

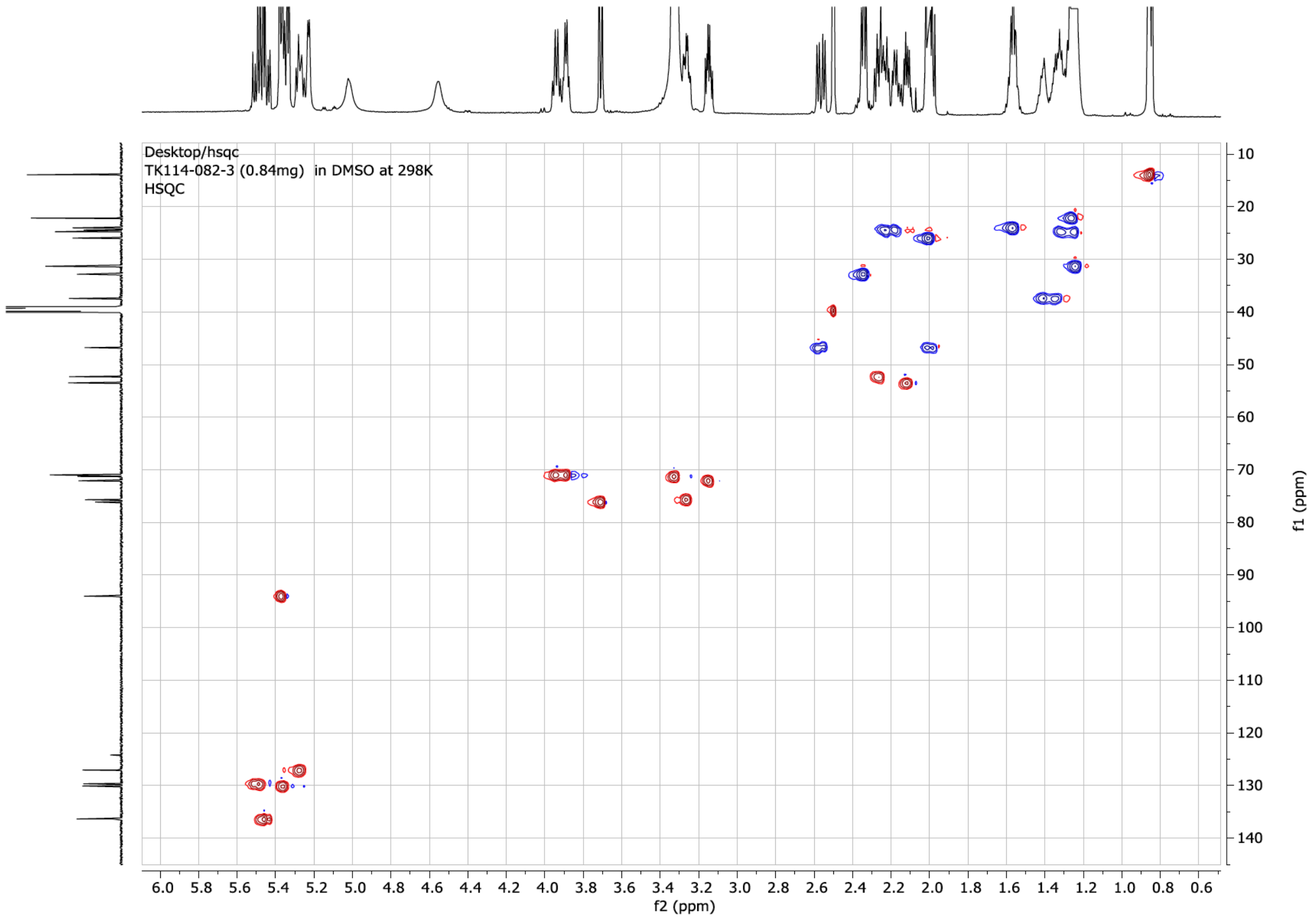

Extended Data Fig. 10 | HSQC NMR spectrum of compound 1 (PGE_2-Acyl-GlcA) in DMSO-d_6.

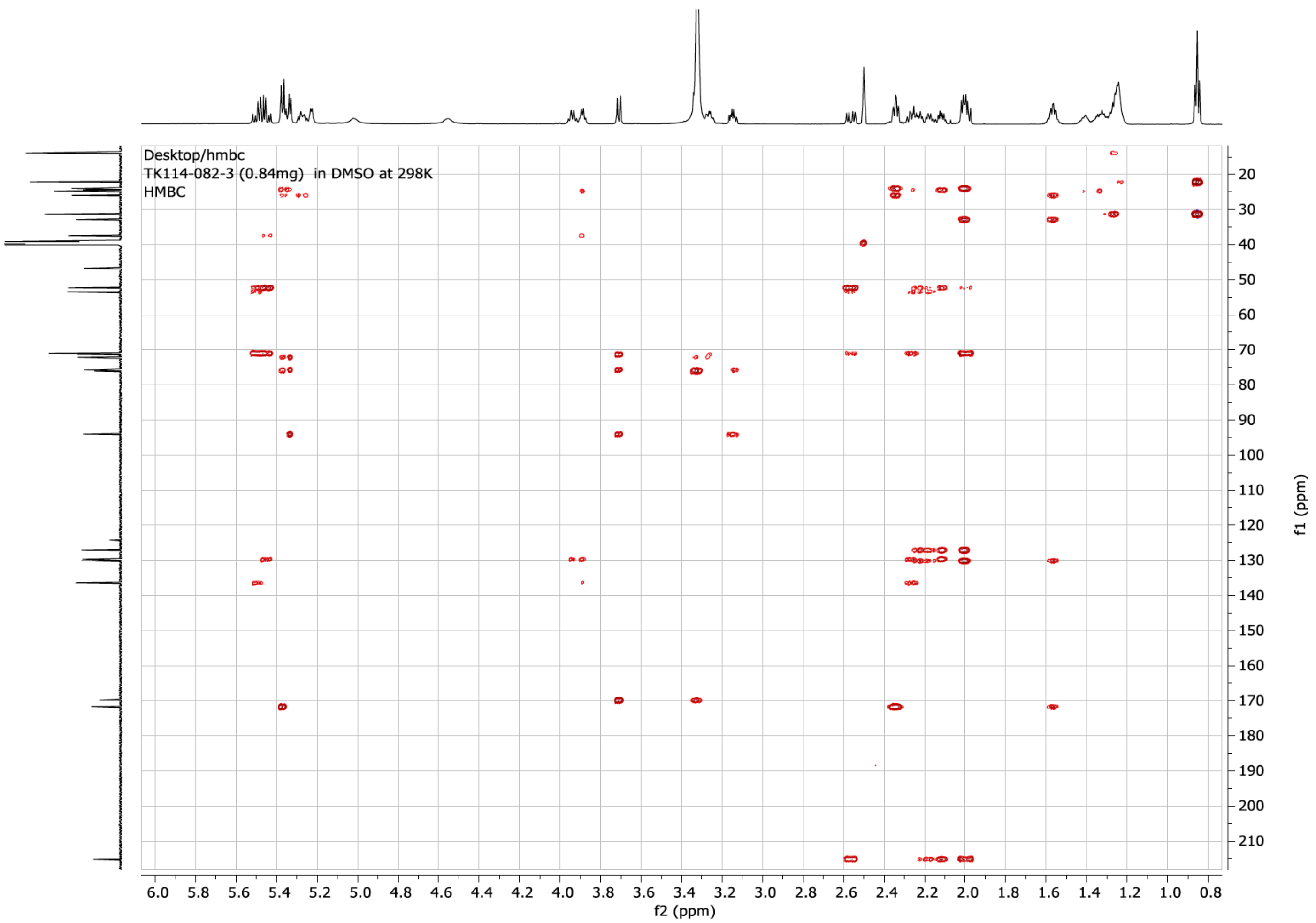

Extended Data Fig. 11 | HMBC NMR spectrum of compound 1 (PGE_2-Acyl-GlcA) in DMSO-d_6.

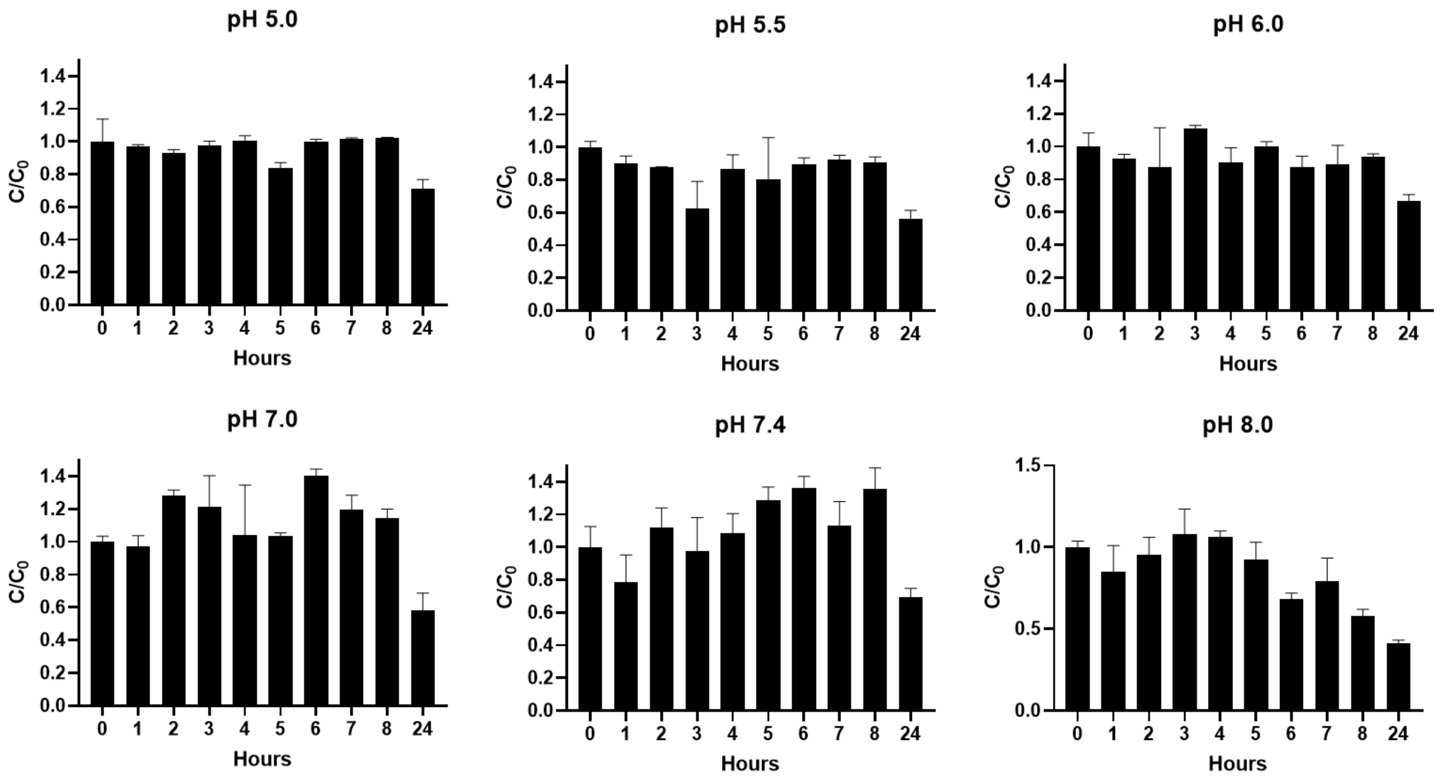

Extended Data Fig. 12 | PGE$_2$-Acyl-GlcA is unstable under alkaline conditions. The stability of PGE$_2$-Acyl-GlcA was assessed in phosphate buffers of varying pH at 37 °C. This compound showed reduced stability at pH values above 7.4.

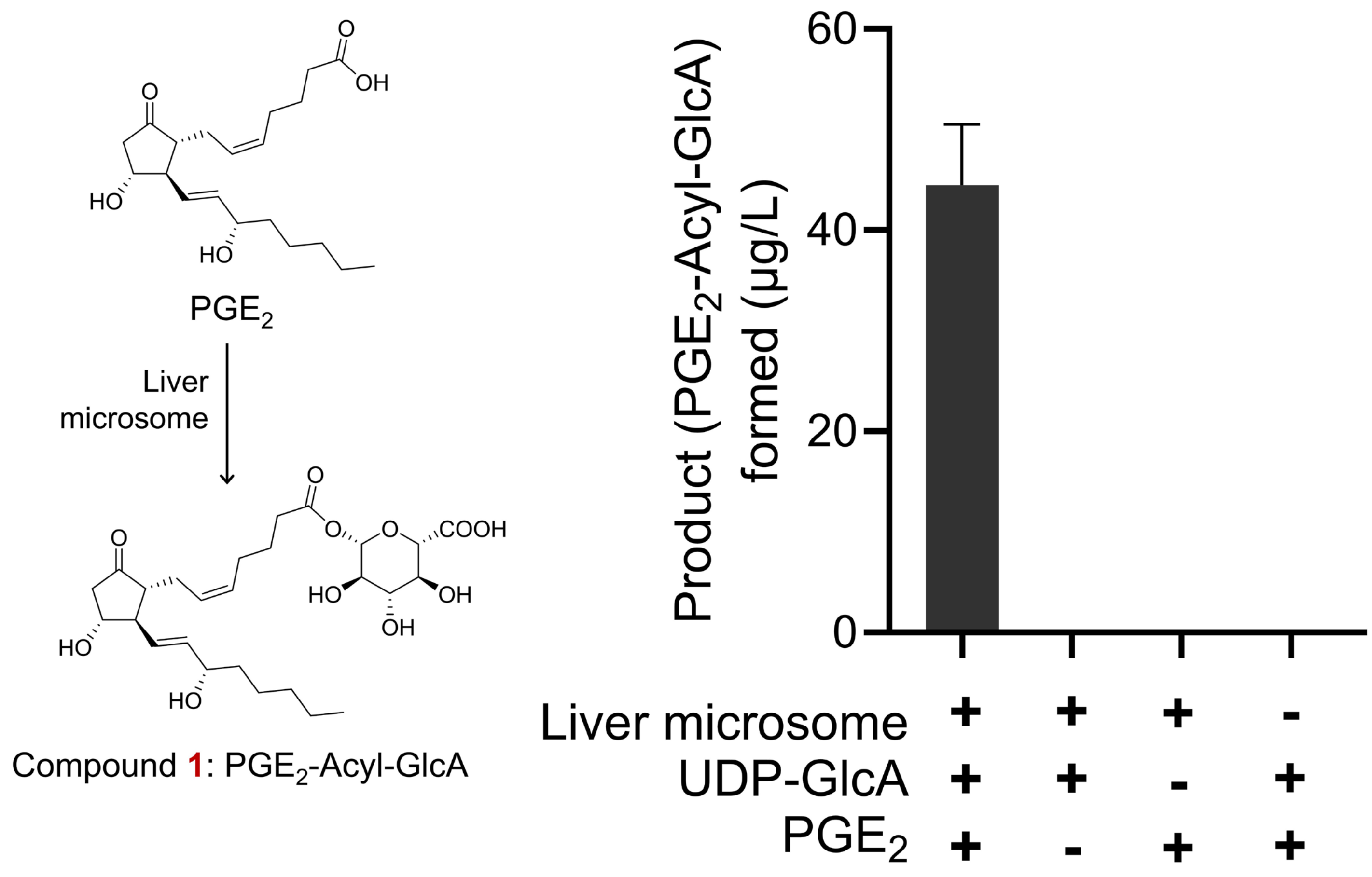

Extended Data Fig. 13 | Liver microsome converts PGE_2 into PGE_2-Acyl-GlcA via UGT-dependent manners.

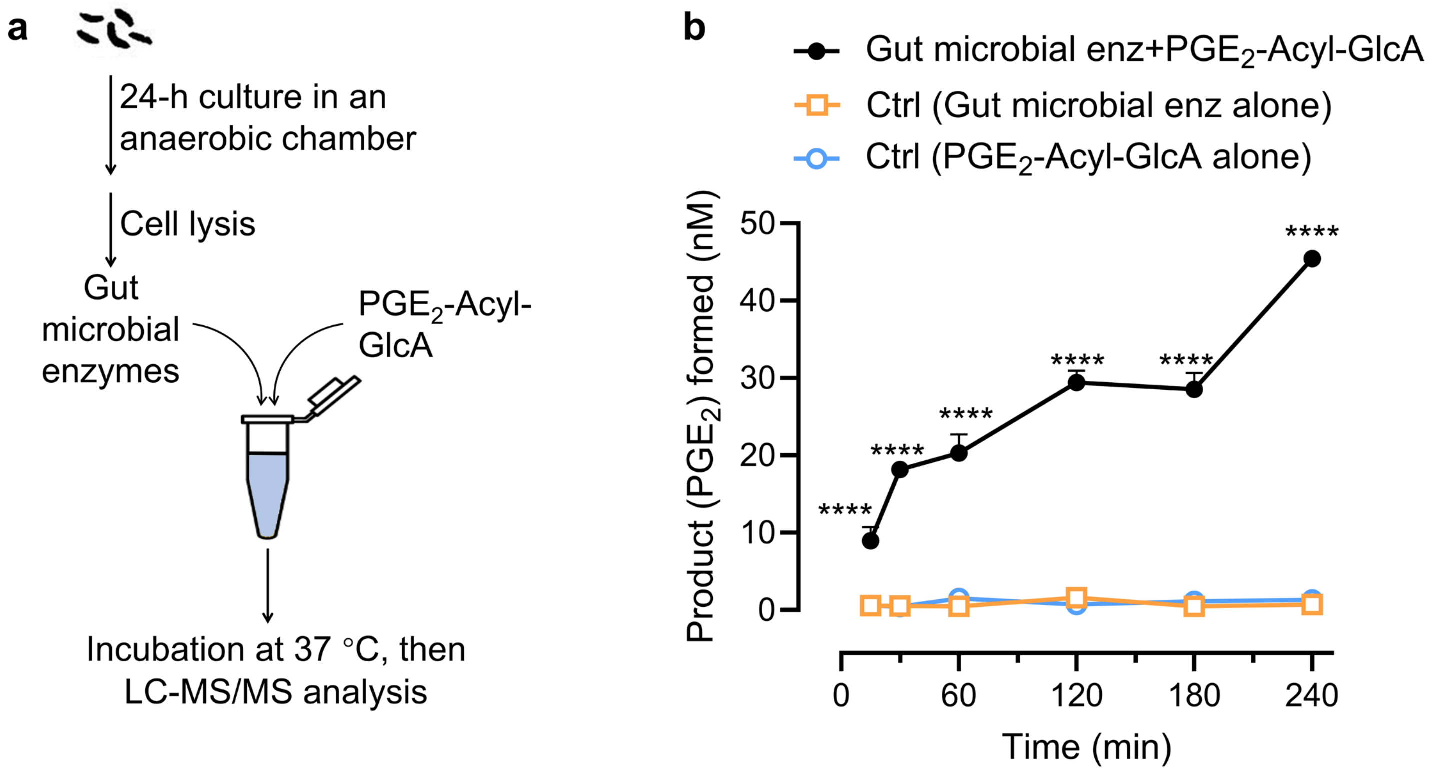

Extended Data Fig. 14 | Gut microbial enzymes catalyze the conversion of PGE_2-Acyl-GlcA to PGE_2. (**a**) Mouse fecal bacteria were cultured overnight in an anaerobic chamber, then the cells were lysed to liberate intracellular enzymes for enzymatic assays. (**b**) Gut microbial enzymes catalyze the conversion of PGE_2-Acyl-GlcA to PGE_2. **** $P < 0.0001$.

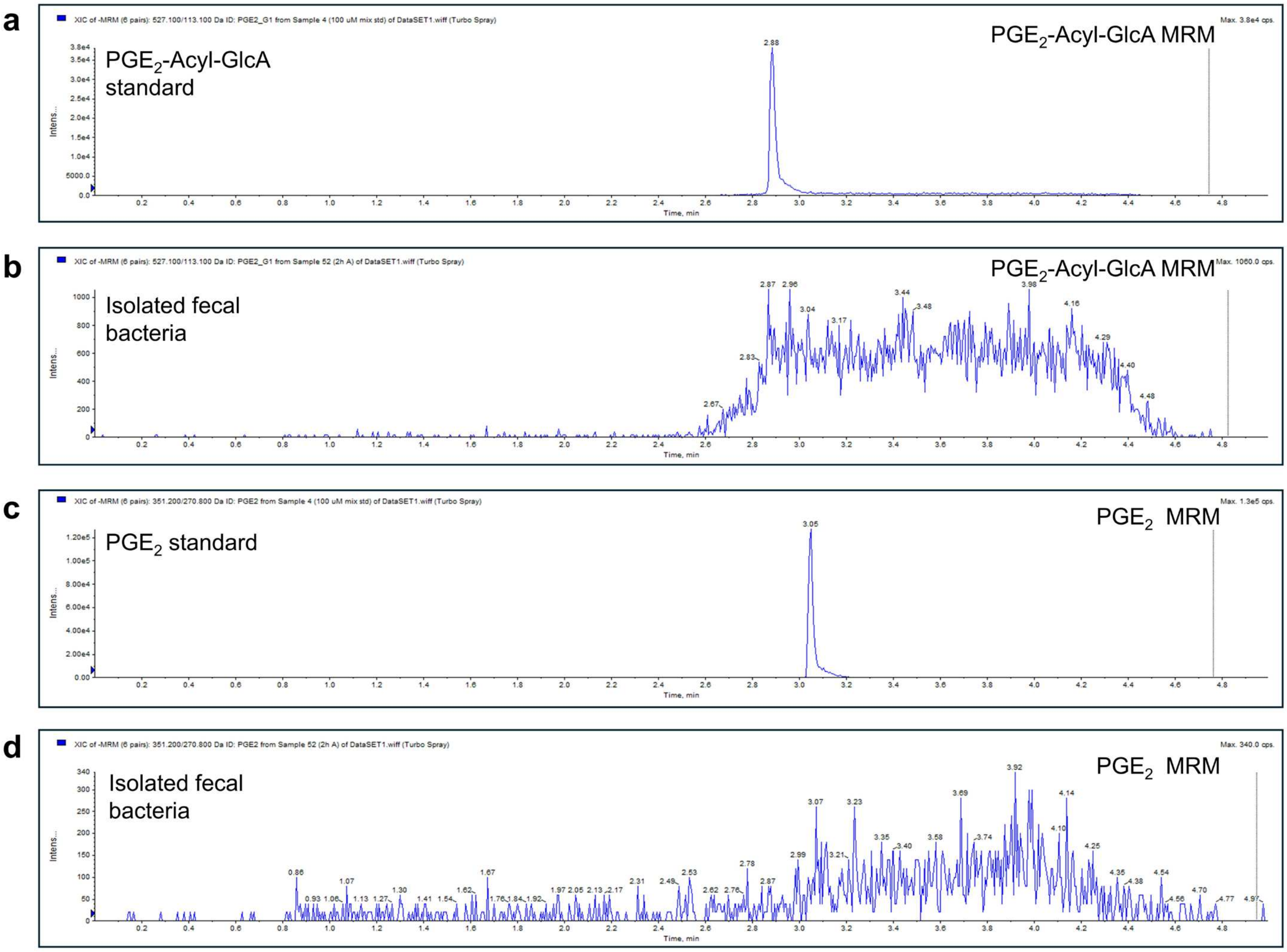

Extended Data Fig. 15 |. Isolated fecal bacteria do not endogenously produce PGE_2 or PGE_2-Acyl-GlcA. Mouse fecal samples were collected and suspended in sterile PBS containing 0.05% L-cysteine, centrifuged at 900 × g for 5 minutes to remove large debris. The resulting supernatant, which contained gut bacteria, was extracted with methanol and then analyzed by LC–MS/MS. (**a**) PGE_2-Acyl-GlcA standard (note that the maximum ion intensity of the Y-axis is 38,000 cps). (**b**) PGE_2-Acyl-GlcA was not detected in isolated fecal bacteria (the maximum ion intensity of the Y-axis is 1,080 cps). (**c**) PGE_2 standard (the maximum ion intensity of the Y-axis is 130,000 cps). (**d**) PGE_2 was not detected in isolated fecal bacteria (the maximum ion intensity of the Y-axis is 340 cps).

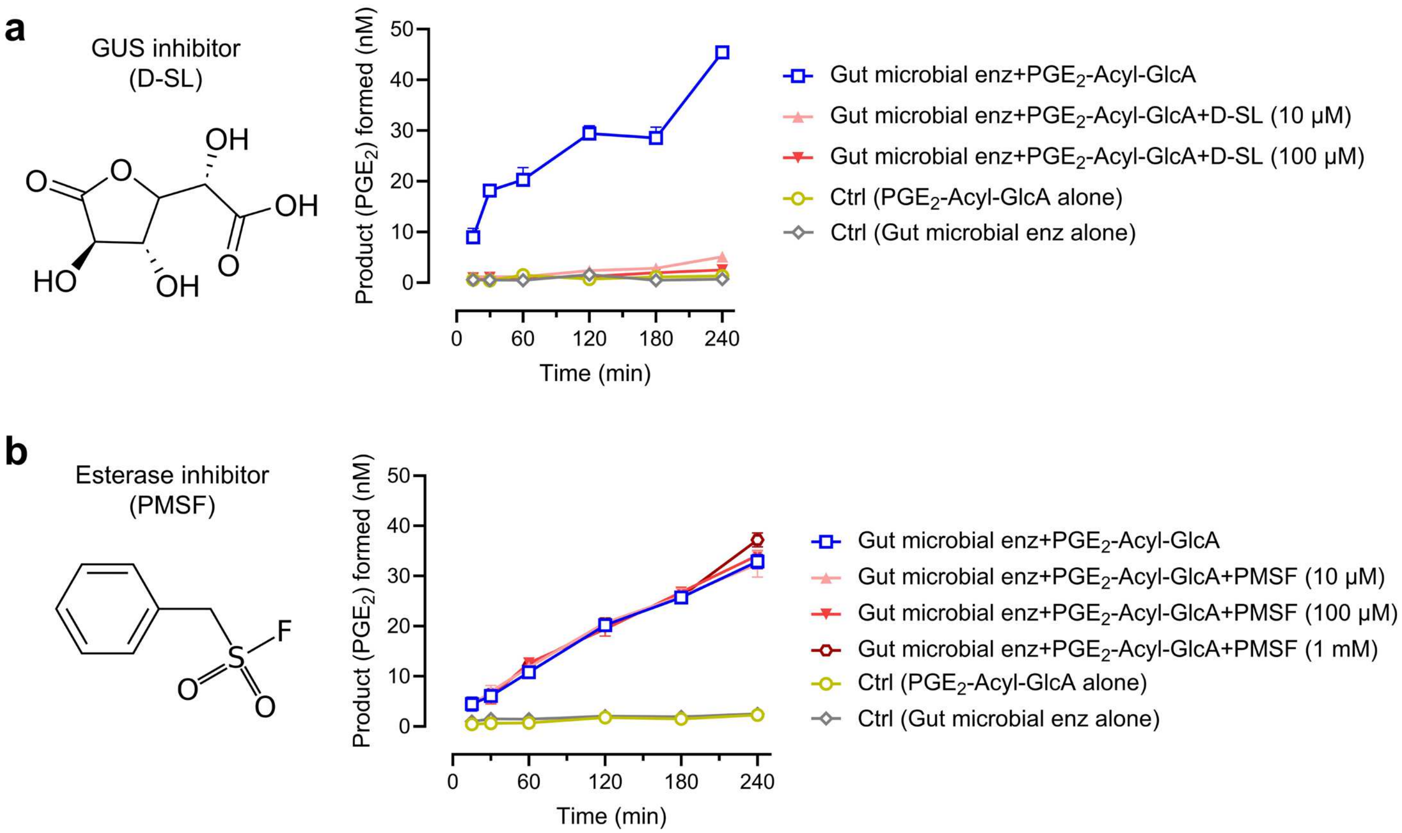

Extended Data Fig. 16 |. Inhibition of GUS enzymes blocks, while inhibition of esterase enzymes doesn't affect, the bacterial conversion of PGE_2-Acyl-GlcA to PGE_2. Mouse fecal bacteria were cultured overnight in an anaerobic chamber. Cells were then lysed to release intracellular enzymes for use in enzymatic assays. (**a**) The pan-GUS enzyme inhibitor (pan-GUSi), D-saccharic acid 1,4-lactone (D-SL), significantly inhibited the fecal bacterial enzymes-catalyzed conversion of PGE_2-Acyl-GlcA to PGE_2. (**b**) The esterase inhibitor phenylmethylsulfonyl fluoride (PMSF) had no effect on this bacterial conversion.

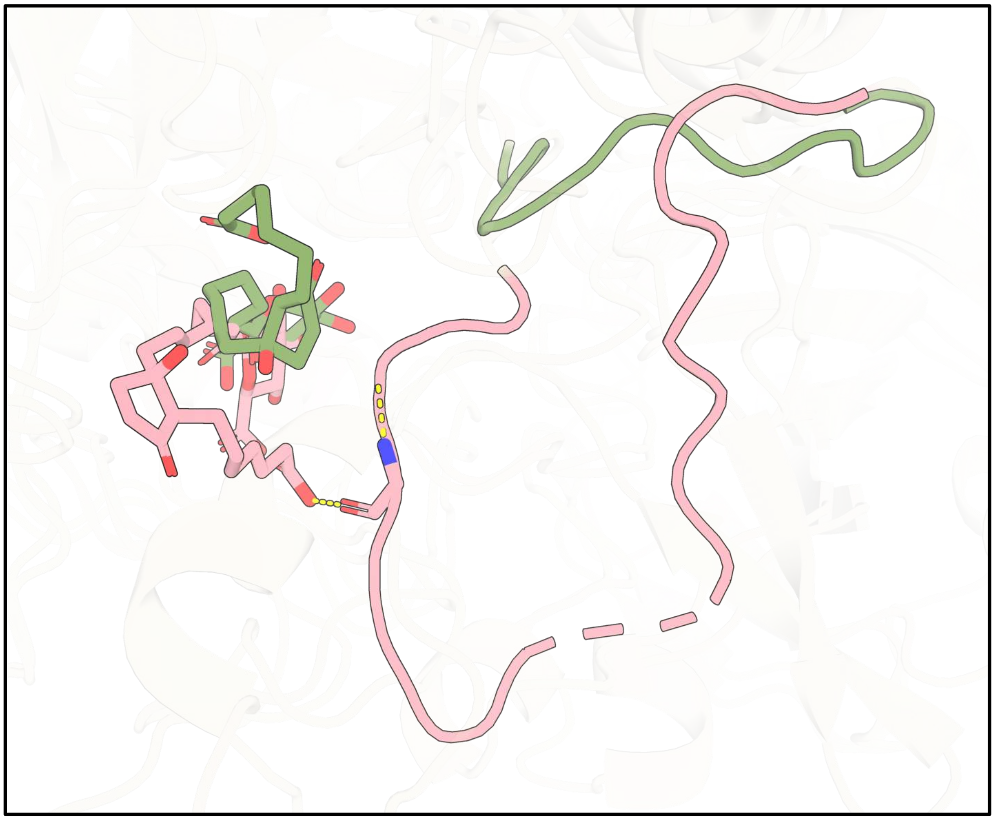

Extended Data Fig. 17 | Structural comparison between Loop-1 (in pink) representative, *E. coli* GUS, and Loop-2 (in green) representative. *P.copri* GUS, showing Loop-1-specific substrate tail anchoring via Gly362 that is absent in Loop-2 family enzymes

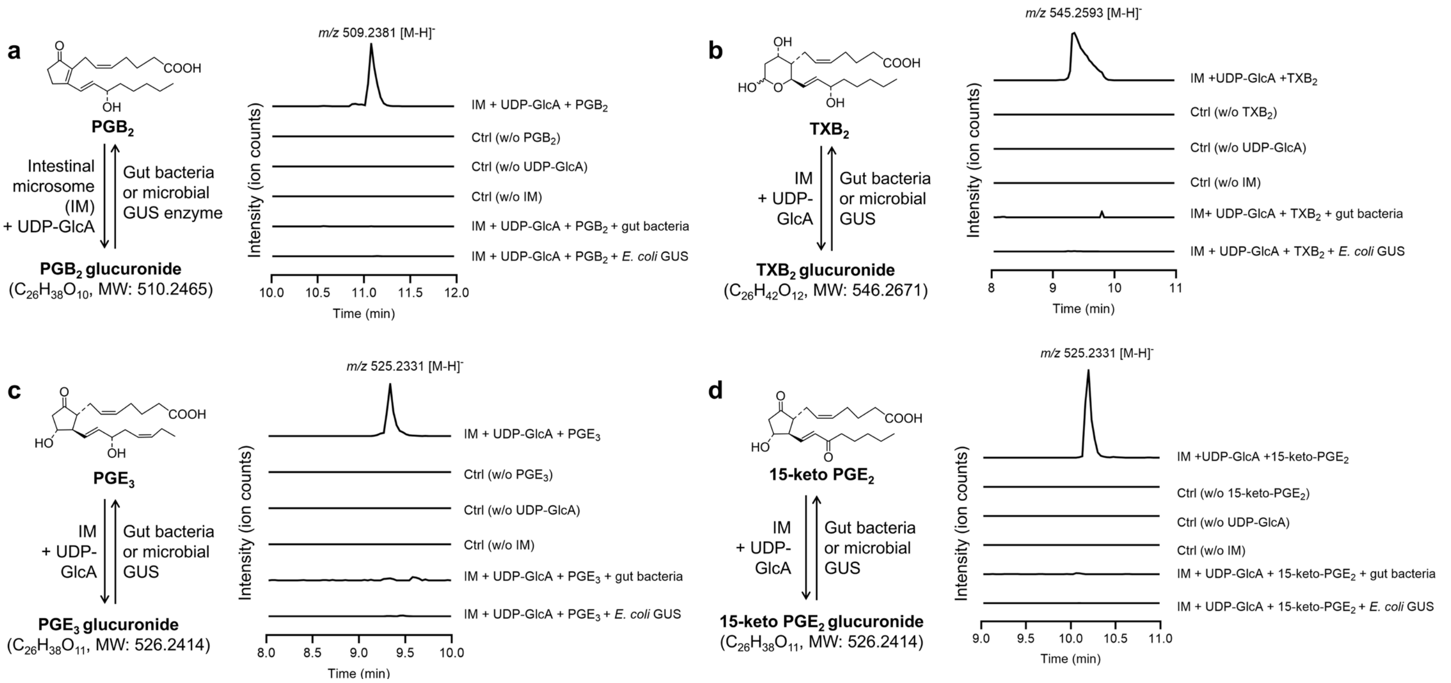

Extended Data Fig. 18 | In addition to PGE_2, other prostaglandins (PGs) also undergo sequential metabolism by host UGT enzymes and microbial GUS enzymes. LC-HRMS analysis supports that intestinal microsomes converted PGB_2 (**a**), TXB_2 (**b**), PGE_3 (**c**), or 15-keto PGE_2 (**d**) into a single predominant glucuronide conjugate via a UGT-dependent manner. The formed glucuronide conjugate was almost completely degraded following incubation with mouse gut bacteria or *E. coli* GUS enzyme.

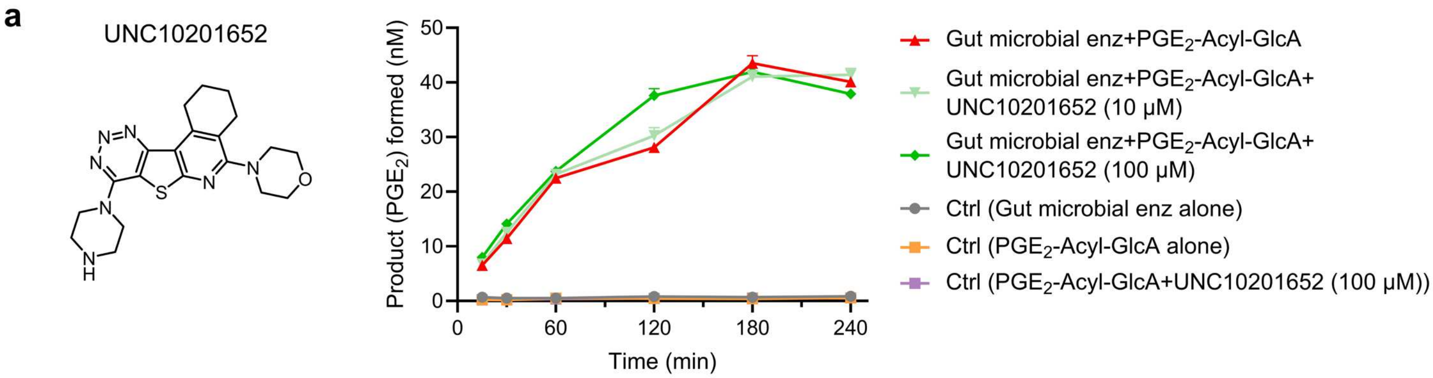

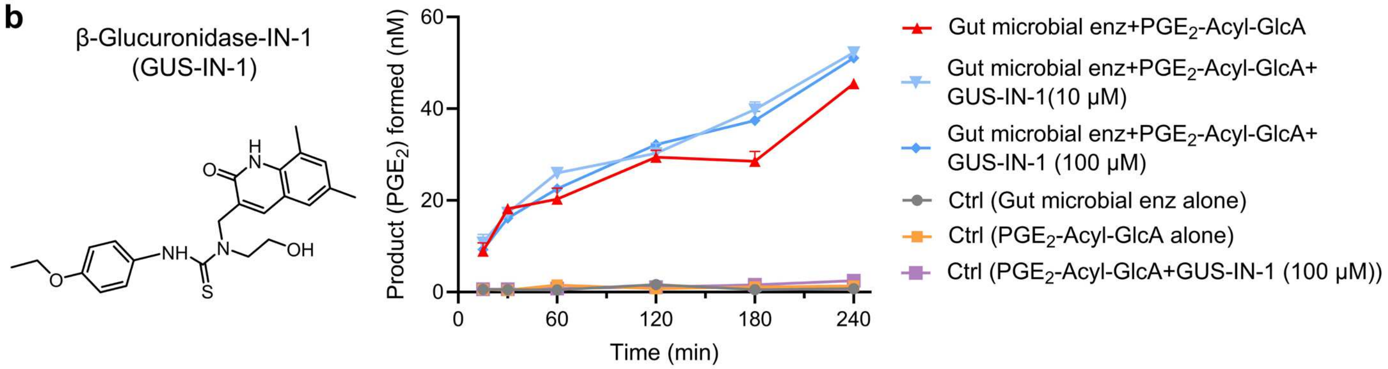

Extended Data Fig. 19 | Two well-established microbial GUS inhibitors, UNC10201652 (**a**) and β-Glucuronidase-IN-1 (**b**), at a dose range of 10 to 100 μM, did not affect fecal bacterial enzyme–mediated processing of PGE_2-Acyl-GlcA.

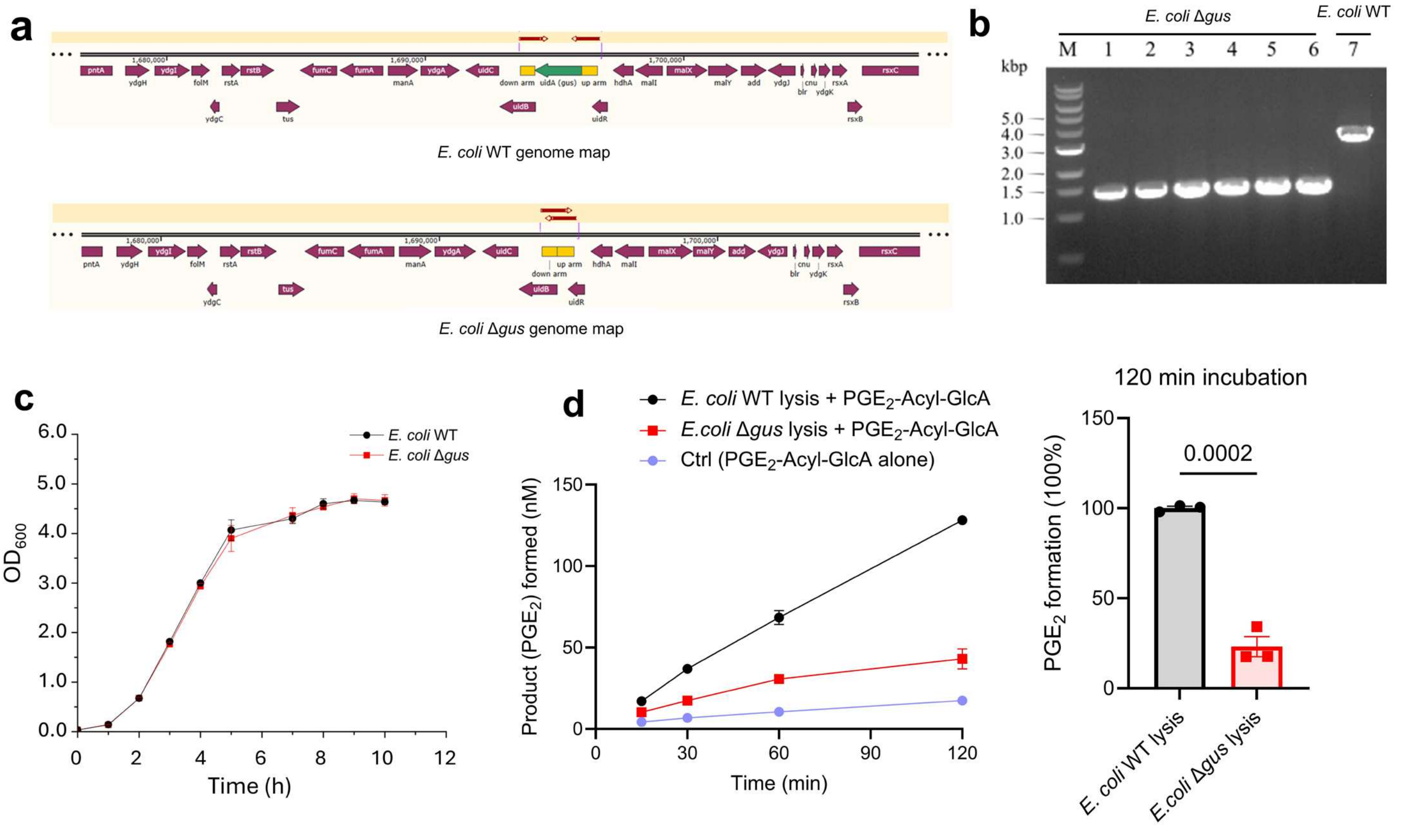

Extended Data Fig. 20 | Generation and validation of a *gus* deletion mutant (Δ*gus*) of *E. coli*. (**a**) Validation of *gus* gene deletion in the *E. coli* Δ*gus* strain by genome sequencing. (**b**) Confirmation of *gus* gene deletion by colony PCR. (**c**) Growth curves of *E. coli* WT and Δ*gus* strains cultured under identical conditions. (**d**) The Δ*gus* strain shows markedly reduced activity to process PGE_2-Acyl-GlcA compared to the WT strain *in vitro*. Left panel: time-course study; Right panel: product (PGE_2) formation after 120 min incubation.

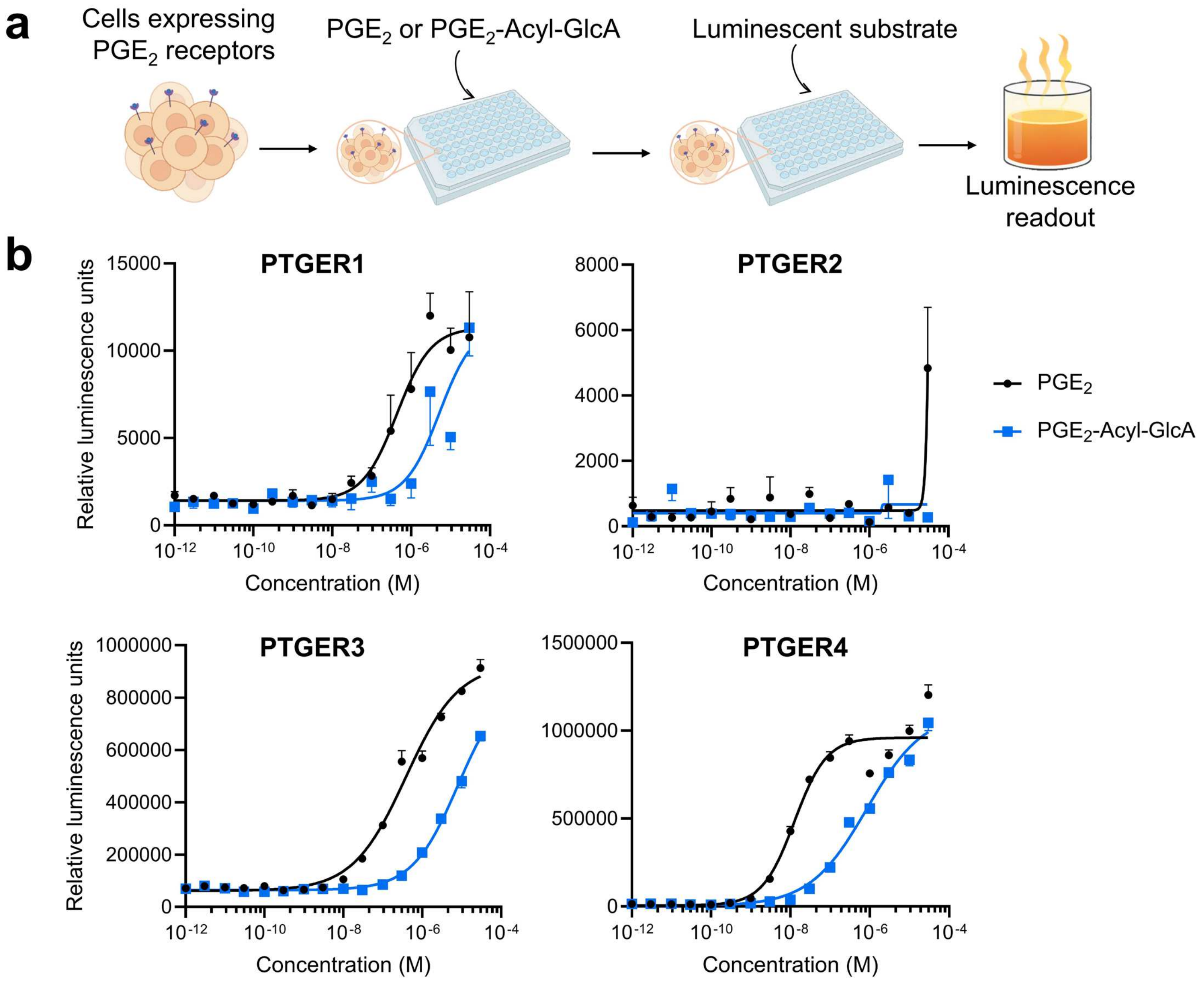

c

Receptor	EC_{50} (nM)	
	PGE_2	PGE_2-Acyl-GlcA
PTGER1	436	5280
PTGER2	37430	NA
PTGER3	385	7998
PTGER4	12.6	840.3

Extended Data Fig. 21 | PGE_2-Acyl-GlcA exhibits markedly reduced biological activity in activating PGE_2 receptors compared to PGE_2. (**a**) Scheme of the luminescence-based functional assay used to assess activation of PGE_2 receptors by PGE_2 or PGE_2-Acyl-GlcA. (**b**) Dose–response activation of the four receptors, PTGER1-4. (**c**) EC_{50} values of PGE_2 and PGE_2-Acyl-GlcA for each receptor calculated from the dose–response curves.

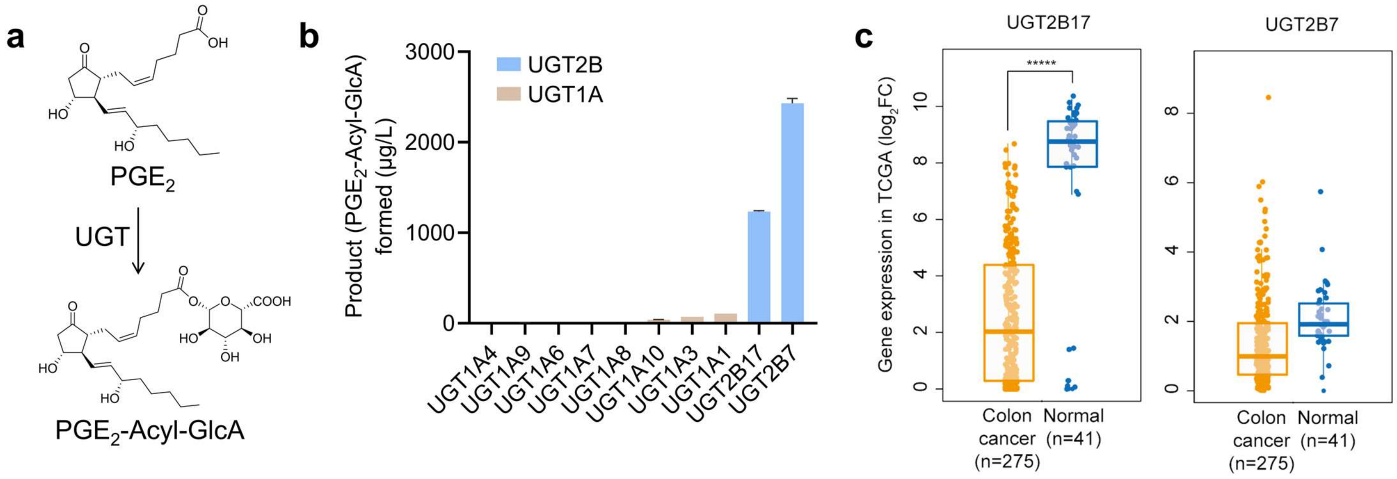

Extended Data Fig. 22 | UGT2B enzymes convert PGE$_2$ to PGE$_2$-Acyl-GlcA and these enzymes are downregulated in human colorectal cancer. (**a**) We tested the effects of purified human UGT enzymes on converting PGE$_2$ to PGE$_2$-Acyl-GlcA. (**b**) UGT2B isoforms, including UGT2B7 and UGT2B17, efficiently convert PGE$_2$ to PGE$_2$-Acyl-GlcA, while UGT1A enzymes have weak activities. (**c**) TCGA data show that UGT2B17 expression is significantly reduced in tumors from patients with colorectal cancer (TCGA-COAD cohort) compared with matched adjacent non-tumor tissues. UGT2B7 displays a similar trend, though not statistically significant. ***** $P < 0.00001$.

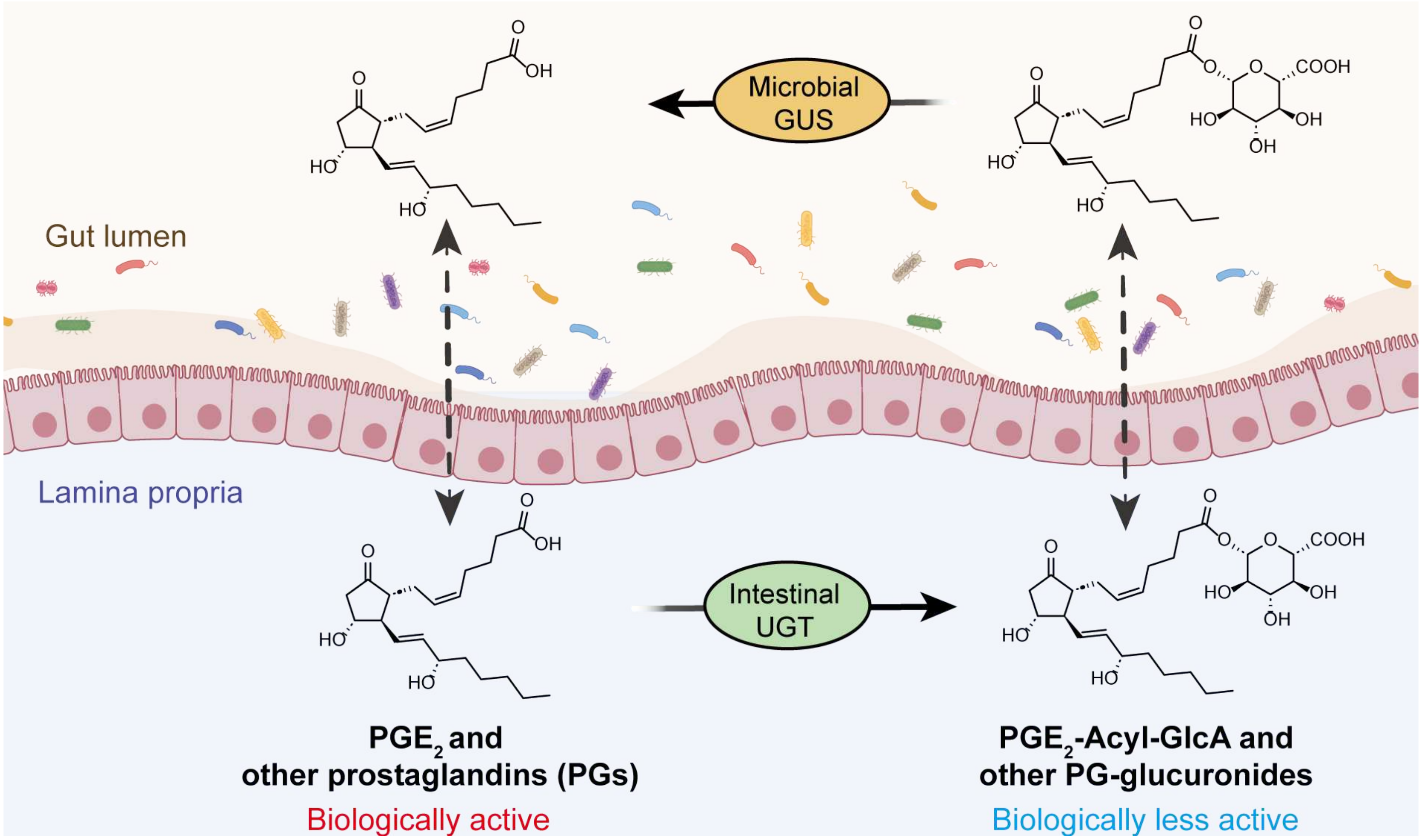

Extended Data Fig. 23 | Microbiota Mediate the Metabolism of Colonic Prostaglandins

Table S1: LC-MS/MS analysis of fatty acid metabolites in the colon of specific pathogen-free (SPF) mice and germ-free (GF) C57BL/6 mice

Metabolite	SPF mice (ng/g in tissue)		GF mice (ng/g in tissue)		P value	Log_2FC*
	Average	SEM	Average	SEM		
PGB_2	17.501	1.971	5.127	1.140	0.000	1.771
PGD_2	1075.680	124.961	357.303	70.638	0.000	1.590
PGD_3	18.564	1.979	10.791	1.683	0.007	0.783
PGE_2	1803.140	137.546	813.348	126.784	0.000	1.149
PGE_3	233.151	25.296	114.883	15.260	0.001	1.021
TXB_2	201.414	23.295	134.126	17.542	0.031	0.587
15-keto PGF_{2a}	9443.565	1162.201	5261.462	450.951	0.003	0.844
6-keto PGF_{1a}	313.054	16.248	260.780	40.076	0.240	0.264
PGF_{2a}	583.858	49.408	680.670	51.750	0.190	-0.221
10,11-DiHDPA	5.508	0.359	5.297	0.240	0.629	0.056
10,11-EpDPA	28.227	5.238	42.767	6.500	0.095	-0.599
11,12-DHET	22.790	1.396	19.841	1.331	0.141	0.200
11,12-DiHETE	0.840	0.062	0.679	0.045	0.048	0.307
11,12-EET	29.311	5.318	50.114	6.941	0.026	-0.774
11,12-EpETE	14.657	2.378	19.815	2.601	0.157	-0.435
11-HETE	407.920	24.742	547.397	34.950	0.004	-0.424
12,13-DHOME	786.307	89.170	683.123	74.051	0.383	0.203
12,13-EpOME	272.176	55.522	560.770	110.535	0.029	-1.043
12-HETE	728.177	71.115	366.607	48.266	0.000	0.990
13,14-DiHDPA	10.870	0.805	10.701	0.546	0.863	0.023
13,14-EpDPA	17.861	3.087	26.620	3.590	0.078	-0.576
13-HODE	3063.358	429.308	4730.724	1382.383	0.262	-0.627
14,15-DHET	19.813	1.388	17.491	0.837	0.166	0.180
14,15-DiHETE	60.093	4.340	51.757	3.028	0.129	0.215
14,15-EET	50.833	8.819	81.628	9.471	0.026	-0.683
14,15-EpETE	8.383	1.420	11.384	1.426	0.150	-0.442
15-HETE	279.337	15.639	333.764	28.077	0.104	-0.257
16,17-DiHDPA	14.158	1.018	14.000	0.618	0.896	0.016
16,17-EpDPA	16.478	2.844	24.853	3.196	0.063	-0.593
17,18-DiHETE	539.981	42.136	550.186	23.990	0.835	-0.027
17,18-EpETE	32.200	4.992	37.347	3.111	0.391	-0.214
17-HDHA	206.573	15.283	171.976	16.201	0.135	0.264
18-HEPE	175.490	24.224	156.423	32.177	0.641	0.166
19,20-DiHDPA	58.702	3.274	73.574	4.364	0.012	-0.326
19,20-EpDPA	121.485	18.805	191.673	22.430	0.025	-0.658
19-HETE	4.475	0.347	6.099	0.653	0.039	-0.447
20-carboxy-AA	8.289	1.494	20.843	4.178	0.010	-1.330
22-HDoHE	3.559	0.342	3.442	0.580	0.863	0.048
5,6-DHET	17.488	2.716	26.253	3.454	0.059	-0.586
5,6-EET	24577.037	3735.281	26668.658	2304.934	0.638	-0.118
5-HETE	33.988	4.055	51.178	8.169	0.073	-0.590
7,8-DiHDPA	9.487	0.673	11.756	0.553	0.016	-0.309
7,8-EpDPA	52.031	8.863	77.968	10.932	0.079	-0.584

8,9-DHET	24.478	1.534	25.189	1.245	0.723	-0.041
8,9-EET	84.535	12.562	110.319	18.682	0.264	-0.384
8-iso PGF_{2a}	13.215	1.012	16.313	1.841	0.155	-0.304
9,10-DHOME	259.717	30.192	307.838	36.050	0.317	-0.245
9,10-EpOME	504.826	109.413	1233.348	282.115	0.025	-1.289
9-HODE	3369.929	428.192	5376.873	1364.437	0.174	-0.674
ARA	25863.716	6210.528	47288.204	8370.224	0.052	-0.871
DHA	23063.806	5687.099	38509.794	6849.750	0.097	-0.740
EPA	1892.046	304.539	2253.641	375.797	0.463	-0.252
Linoleic acid	93932.088	9374.863	156158.667	16601.491	0.004	-0.733
Lipoxin_A4	29.073	2.732	44.703	4.358	0.006	-0.621
Lipoxin_B4	3.322	0.306	1.546	0.209	0.000	1.104
LTB4	8.458	0.928	4.866	0.659	0.005	0.797

*FC: fold of change, representing the level of the lipid metabolite in the SPF mice to that of the GF mice.

Table S2: NMR Signal assignments (ppm), m (J/Hz) for PGE_2 and PGE_2-Acyl-GlcA

Position	PGE_2		PGE_2-Acyl- GlcA	
	δC*/ppm	δH*/ppm, m (J/Hz)	δC*/ppm	δH*/ppm, m (J/Hz)
1	174.3	-	171.7	-
2	33.2	2.17, t (7.4)	32.8	2.35, td (7.5, 1.8)
3	24.4	1.52, p (7.3)	24.0	1.57, m
4	26.2	1.98, m	26.0	2.01, m
5	130.4	5.36, m	130.1	5.36, m
6	126.8	5.26, m	127.1	5.27, m
7	24.4	2.23, m; 2.17, m	24.4	2.23, m; 2.18, m
8	53.5	2.12, dt (11.3, 5.6)	53.5	2.13, m
9	215.1	-	215.1	-
10	46.8	2.57, dd (18.1, 7.2); 2.00, m	46.8	2.57, dd (18.1, 8.0); 2.00, m
11	71.0	3.94, q (8.4)	71.0	3.95, q (7.9)
12	52.3	2.26, m	52.3	2.27, dd (11.3, 8.0)
13	129.7	5.49, dd (15.4, 7.7)	129.7	5.51, dd (15.5, 7.6)
14	136.4	5.44, dd (15.4, 6.1)	136.4	5.45, dd (15.4, 6.1)
15	71.0	3.89, q (6.2)	71.0	3.90, q (6.1)
16	37.5	1.41, m; 1.32, m	37.4	1.41, m; 1.35, m
17	24.4	1.32, m; 1.25, m	24.8	1.32, m; 1.24, m
18	31.3	1.25, m	31.3	1.25, m
19	22.2	1.26, m	22.2	1.27, m
20	13.9	0.85, t (6.9)	13.9	0.86, t (6.9)
1’			94.0	5.38, d (8.2)
2’			72.1	3.16, td (8.5, 5.2)
2’OH			-	5.34, d (5.5)
3’			75.7	3.27, td (8.9, 4.3)
3’-OH			-	5.24, d (4.9)
4’			71.3	3.33, m
5’			76.1	3.72, d (9.6)
6’			169.8	-

*Referenced to residual DMSO at 39.520, 2.500 ppm; s = singlet, d = doublet, t = triplet, q = quartet,p = pentet, m = multiplet, dd = doublet of doublets, dt = doublet of triplets, td = triplet of doublets.

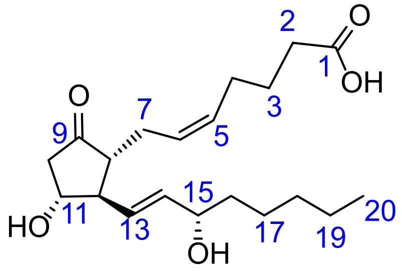

PGE_2

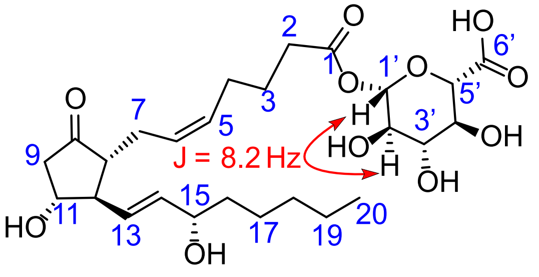

PGE_2-Acyl-GlcA

Chemical structure and numbering of PGE_2 and PGE_2-Acyl-GlcA. The coupling constant of H-1’ and H-2’ supports that the glucuronide moiety is β configuration.

Table S3: Structural parameters used in modeling and analysis of bacterial GUS proteins

GUS isoforms *	Clade	Structures used for modeling	Uniprot code	NCBI Accession	Catalytic residues	Total models
E. coli GUS protein	L1	6LEL	P05804	NP_416134.1	E409<br>E511<br>N564	1000 models
C. perfringens GUS protein	L1	4JKM	Q8XP19	WP_003467686.1	E412<br>E505<br>N567	1000 models
R. gnavus GUS protein	FMN	AlphaFold3 generated	R5TSA0	6MVG_A	E402<br>E490<br>N555	1000 models
Eubacterium (UNC 361-28) GUS protein	FMN	AlphaFold3 generated	UniParc UPI00033F5701	WP_022501306	E387<br>E475<br>N540	1000 models
S. agalactiae GUS protein	L1	4JKL	Q8E0N2	WP_000966715	E399<br>E492<br>N554	1000 models
E.eligens GUS protein	L1	6BJQ	C4Z6Z2	WP_012740861	E413<br>E504<br>N566	1000 models
B.fragilis GUS protein	mL1	3CMG	Q5LIC7	3CMG_A	E429<br>E510	1000 models
F.saccharvivorans GUS protein	hybrid	6NCY	A0A174EHD1	6NCY_A	E407<br>E491<br>N555	1000 models
B.mass GUS protein	mL1	AlphaFold3 structure available on: https://alphafold.e	U6RLV2	WP_270534885	E418<br>E499<br>N570	1000 models

		bi.ac.uk/entry/U6<br>RLV2				
B.dorei GUS protein	NL	6ED1	A0ABF7PHL9<br>I9FNR0<br><br>remarks:<br>unreviewed	WP_007841259	E421<br>E512<br>N577	1000 models
P.copri GUS protein	L2	AlphaFold3<br>generated	N/A	WP_217761416	E392<br>E489<br>N554	1000 models
R. hominis 1 GUS protein	mL1	AlphaFold3<br>generated	A0A395V874	7KGZ_A	E369<br>E457<br>N523	1000 models
F. prausnitzii GUS protein	mL1	6U7I	UPI000BED8C8D	WP_097774993.1	E411<br>E503<br>N565	1000 models
R. hominis 2 GUS protein	FMN	AlphaFold3<br>structure<br>available on:<br>https://alphafold.e<br>bi.ac.uk/entry/A0<br>A395V8I7	A0A395V8I7	6MVH_A	E379<br>E467<br>N532	1000 models
Bifidobacterium angulatum GUS protein	L2	AlphaFold3<br>generated	C4FCU9	WP_418218672	E462<br>E574<br>N650	1000 models
P.merdae GUS protein	mL2	AlphaFold3<br>structure<br>available on:<br>https://alphafold.e<br>bi.ac.uk/entry/K5<br>ZWV5	K5ZWV5	WP_005639106	E426<br>E510<br>N606	1000 models

B.ovatus GUS protein	m1,2	AlphaFold3 structure available on: https://alphafold.ebi.ac.uk/entry/A7LXZ4	A7LXZ4	WP_004298526	E415<br>E504<br>N576	1000 models
Parabacteroides MSP GUS protein	mL2	AlphaFold3 generated	N/A	WP_010800018.1	E420<br>E574<br>N646	1000 models

* Amino acid sequences of the GUS isoforms are shown below:

***E.coli* GUS protein**
MGSSHHHHHHSSGLVPRGSHMMLRPVETPTREIKKLDGLWAFSLDRENCGIDQRWWESALQESRAIAVPGSFNDQFADADIRNYAGNVWYQR
EVFIPKGWAGQRIVLRFDAVTHYGKVWVNNQEVMEHQGGYTPFEADVTPYVIAGKSVRITVCVNNELNWQTIPPGMVITDENGKKKQSYFHDFF
NYAGIHRSVMLYTTPNTWVDDITVVTHVAQDCNHASVDWQVVANGDVSVELRDADQQVVATGQGTSGTLQVVNPHLWQPGEGYLYELCVTAK
SQTECDIYPLRVGIRSVAVKGEQFLINHKPFYFTGFGRHEDADLRGKGFDNVLMVHDHALMDWIGANSYRTSHYPYAEEMLDWADEHGIVVIDET
AAVGFNLSLGIGFEAGNKPKELYSEEAVNGETQQAHLQAIKELIARDKNHPSVVMWSIANEPDTRPQGAREYFAPLAEATRKLDPTRPITCVNVM
FCDAHTDTISDLFDVLCLNRYYGWYVQSGDLETAEKVLEKELLAWQEKLHQPIIITEYGVDTLAGLHSMYTDMWSEEYQCAWLDMYHRVFDRVS
AVVGEQVWNFADFATSQGILRVGGNKKGIFTRDRKPKSAAFLLQKRWTGMNFGEKPQQGGKQ

***C.perfringens* GUS protein**
MLYPIITESRQLIDLSGIWKFKLNEGNGLTEELSKAPLEDTIEMAVPSSYNDLVESQEVRDHVGWVWYERNFTIPKTLLNERIVLRFGSATHEAKVY
LNGELLVEHKGGFTPFEAEINDLLVSGDNRLTVAVNNIIDETTLPVGLVKEVEVDGKKVIKNSVNFDFFNYAGIHRPVKIYTTPKSYIEDITIVTDFKEN
NGYVNYEVQAVGKCNIKVTIIDEENNIVAEGEGKEGKLTINNVHLWEPMNAYLYKLKVELLDDEEIIDTYFEEFGVRTVEVKDGKFLINNKPFYFKGF
GKHEDSYVNGRGINEAINIKDFNLMKWIGANSFRTSHYPYSEEIMRLADREGIVVIDETPAVGLHLNFMATGFGGDAPKRDTWKEIGTKEAHERIL
RELVSRDKNHPCVVMWSVANEPDSDSEGAKEYFEPLIKLTKELDPQKRPVTVVTYLMSTPDRCKVGDIVDVLCLNRYYGWYVAGGDLEEAKRM
LEDELKGWEERCPKTPIMFTEYGADTVAGLHDTVPVMFTEEYQVEYYKANHEVMDKCKNFVGEQVWNFADFATSQGIIRVQGNKKGIFTRERKP
KMIAHSLRERWTNIPEFGYKK

***R.gnavus* GUS protein**

HHHHHHSSGVDLGTENLYFQSNAMREIFNLNEKWAFSKEAIKPPVAMPQNWYWVNLPHTWNAIDGQDGGNDYYRGTCFYAKEVQKEELPEGE
QYYLEFLGVNASADVYVNGNHLDHHDGGYSTWRVNMTDALTDGKNLIVVAVDNSANDRVYPQKADFTFYGGMYRDVNIIAVNKSHFDLDYYGG
NGLKVTPEVADKNAKIAVEVFLSGEKAGQQLVYQITDAEGVLAAETKTGISDKQVNLEITDVHLWNGRKDPYLYTATVRLMEDGVCIDSVSTRFGC
RTFTIDPDKGFFLNGNSYPLRGVSRHQDRAGNGNALLPEHHREDIDLICEMGATTIRLAHYQHAQYFYDLCDEKGLVLWAEIPYISQHMKNGREN
TISQMKELIVQNYNHPSIVVWGLSNEITMSGEADEDLMENHHILNDLAHEMDPTRLTTMAVVSMCDMHNPYIQIPDVVSYNHYFGWYGGDTSMN
GPWMDEFHKEFPKIPIGMSEYGCEALNWHTSDPKQGDYTEEYQAHYHEELIKQLYTRPYIWATHVWNMFDFAADARNEGGENGMNHKGLVTF
DRKYKKDAFYAYKAWLSDEPFVHICGKRYVDRVENVTKVTVYSNQKTVELFANGESLGKKEASDHFFYFEVPNHGETKLLAVAGECRDESFIKKV
EKFNEAYRLTEKGAVLNWFDITAPDGFFSLNDTVGDIMSNEDGNKVMQELFQSFGQAKAQGGGMNSGMMKMLSGFTVLRMINMMGLTSQMTS
QPGEAEKTNEVSKEQLLAINDRLNQIRK

***Eubacterium* GUS protein**
MNKKEVKMATKNINASWQFTKDAITVAEGYSVLTQGNWEALDLPHTWNGKDGQDGGNDYHRGTCYYVKNMKREEFGSEPITYIEFNGANSSA
WLYVNGKEAGHHDGGYSTWRVNVTDFLADENEIVVAVDNAPNDHVYPQMADFTFYGGLYRDVNVISVPETHFDLDYYGTHGIAVTPIVEGANAS
VEVEVFVTDATEEDTLEYVIKNGDEVIATKSVSANETKVTFEIENVHLWNGRKDPHLYTAEVTLKRNDTVLDERSTRFGCRTFVIDPEKGFILNGER
YPLHGVSRHQDRPHIGNALTHKEHKEDIELILEMGANTIRLAHYQHDQYIYDLCDETGLVLWAEIPYISKHLPNGRENTISQMKELIIQNYNHPSIVV
WGLSNEITMGGGEDDDLIENHNILNDLCHQMDPTRLTTMAVVSMCSIDAKYIQIPDVISYNHYYGWYGGTTDMNGEFFDDFHKKYPNIPIGLSEYG
CEALNWHTSNPVQGDYTEEYQAYYHEELIKQLFTRDYIWATHVWNMYDFAADARAEGGENGMNHKGLVTFDRKYKKDAFYAYKAWLSDDPFV
HICGKRYVDRVEDTTKVTVYSNQPEVELFVNGESLGKQTSDVHFFYFDVPNAGESTLVAKAGDCSDESKIRKVDTFNEEYRLKEEGEVLNWFDID
MPEGYFNINDKIGDIMKANEGKMFIDELMLKIMKGADGGAAEAAEAAAGAGSEGLMKMLSGFTVKRMLNLMGTAGGGKQFTKEELLELNSKLNKI
KR

***S.agalactiae* GUS protein**
MLYPLLTKTRNTYDLGGIWNFKLGEHNPNELLPSDEVMVIPTSFNDLMVSKEKRDYIGDFWYEKVIEVPKVSEDEEMVLRFGSVTHQAKIYVDGVL
VGEHKGGFTPFEVLVPECKYNNEKIKVSICANNVLDYTTLPVGNYSEIIQEDGSIKKKVRENFDFFNYAGVHRPLKLMIRPKNHIFDITITSRLSDDL
QSADLHFLVETNQKVDEVRISVFDEDNKLVGETKDSRLFLSDVHLWEVLNAYLYTARVEIFVDNQLQDVYEENFGLREIEVTNGQFLLNRKPIYFK
GFGKHEDTFINGRGLNEAANLMDLNLLKDMGANSFRTSHYPYSEEMMRLADRMGVLVIDEVPAVGLFQNFNASLDLSPKDNGTWNLMQTKAAH
EQAIQELVKRDKNHPSVVMWVVANEPASHEAGAHDYFEPLVKLYKDLDPQKRPVTLVNILMATPDRDQVMDLVDVVCLNRYYGWYVDHGDLTN
AEVGIRKELLEWQDKFPDKPIIITEYGADTLPGLHSTWNIPYTEEFQCDFYEMSHRVFDGIPNLVGEQVWNFADFETNLMILRVQGNHKGLFSRNR
QPKQVVKEFKKRWMTIPHYHNKKNSVK

***E.eligens* GUS protein**

MLYPVLTQSRLLSDLSGVWDFKLDNGKGFEEKWYEKPLKDADTMPVPASYNDLKEGTDFRDHYGWVFYQRNISVPEYVKSQRIVLRCAAVTHY
AMIYLNGKLICEHKGGFLPFEVELNDDLQDGDNLLTIAVNNVIDYTTLPVGGKANMMSGMMGGMGAGASDKPQNNPNFDFFNYCGITRPVKIYTT
PETYINDITVTADIDFTKEEPSAVLNYNVEIKGKDYNNITCKVELFDEEGTKLSETEGSEGTFEISNVRLWQPLNAYLYKIKVTAGQDVYTLPYGVRS
VRVDGTKFLINEKPFYFKGYGKHEDTFPNGRGINLPMNTKDISIMKWQHANSFRTSHYPYSEEMMRLCDEEGIVVIDETTAVGVNLQFGGGANFG
GERIGTFDKEHGVQTQEHHKDVIRDLISRDKNHACVVMWSIANEPDSAAEGAYDYFKPLYDLARELDPQKRPCTLVSVQGTTADTDCSSQLSDVI
CLNRYYGWYFGGPDLEVSEIGLRKELSDWGKLGKPVMFTEYGADTVSGLHDTTSVMYTEEYQVEYYEMNNKVFDEFDFVVGEQAWNFADFAT
SQSLLRVQGNKKGLFTRDRKPKMVAHYFRNRWSTIPEFGYKTK

***B.fragilis* GUS protein**
MSLRQDILLNNNWNFRFSHQVQGDTRRVDLPHTWNAQDALAGKIDYKRGIGNYEKALYIRPEWKGKRLFLRFDGVNSIADVFINRKHIGEHRGGY
GAFIFEITDLVKYGEKNSVLVRANNGEQLDIMPLVGDFNFYGGIYRDVHLLITDETCISPLDYASPGVYLVQEVVSPQEAKVCAKVNLSNRAADGTA
ELQVLVTDGTKVICKESRNVSLKQGADILEQLPLLIQKPRLWNGCEDPFMYQVSISLHKDGKQIDSVTQPLGLRYYHTDPDKGFFLNGKHLPLHGV
CRHQDRAEVGNALRPQHHEEDVALMREMGVNAIRLAHYPQATYMYDLMDKHGIVTWAEIPFVGPGGYADKGFVDQASFRENGKQQLIELIRQH
YNHPSICFWGLFNELKEVGDNPVEYVKELNALAKQEDPTRPTTSASNQDGNLNFITENIAWNRYDGWYGSTPKTLATFLDRTHKKHPELRIGISE
YGAGASIYHQQDSLKQPSASGWWHPENWQTYYHMENWKIIAERPFVWGTFVWNMFDFGAAHRTEGDRPGINDKGLVTFDRKVRKDAFYFYKA
NWNKQEPMIYLAEKRCRLRYQPEQTFMAFTTAPEAELFVNGVSCGKQKADTYSTVVWKNVKLTSGENIIRVTTPGKKPLTDEVTVEYKEDREGH
HHHHH

***F.saccharvivorans* GUS protein**
MEAKKEKKYMSDIHLEDYTEQYETGFATVDTMIFEGGRREELLNGGWHYAVDQYDTCLRQKWYKERYRDEKGFTVPIDYSFDEWPVMQLPCS
WNTIDPMYLLYEGSMVFTRKFSYIAEREETVFLKVGAANYLCRVFLNGKYVGMHRGGSTPAFWNITEYLKAENRIVLAVDGTRRPEQVPTENTD
WFNYCGVYRDIALIRVPKCHIKTFKIALVPDGTFGHVMAKVTLSEKITAKAELVIEELGVSRKIQLENGAGEVVFDAKPELWTPEKPKLYDVKVTCG
TDTVSDRVGFREIRVNGRDILLNGEPVFLRGISCHEDSVENGKGLTREERIENIRIAKELGCNFMRLAHYPHNEEMAKLADELGLLLWEEIPVYWAI
RFEREKTYEDAQNQLRELINRDWNRASVIIWSVGNENADTDERLKFMSVLAECAHREDETRMVSAACLVNAAKNKIEDRLMEYLDIIGINEYCGW
YTPDFAMLPALMENSQPDKPVIVTEFGADALPHHHGTISDKGTEECQADVYEKQIATLRNIDYIKGMTPWILYDFRCPRRTSLIQKYYNRKGLLSE
DKKYRKPAFYVLQKFYEELKRKEQENLYFQSGSHHHHHH

***B.mass* GUS protein**
QRENILINQDWNFRFSHQVDKNSSRRVDLPHTWNAQDALSGKPDYKRGIGNYDKKLFIRSEWKGKRLFLRFEGANCVSNVFINGKQIGEHRGGY
GAFIFEITDKVNYGKDNTVLIRVNNGEQLDVMPLVGDFNFYGGIYRDVHLLVTEDICISPLDYASPGVYLFQQHVGEKQAAVLARINLSNGTEHPRQ
ATLRLQVKEGDKVVYQADKKVTVAPHTSVQPEEMSFTLLNPRLWNGREDPFMYQTVITLVKDGKEIDKVEQPLGLRYYTTDADRGFFLNGKHLP
LHGVCRHQEWAEVGNALRPMHHEEDTRLMLEMGVNAIRLAHYPQATYMYDLMDRNGIVTWAEIPFVGPGGYADKGFVDQPSFRENGKEQLKE

MIRQHFNHPSICFWGLFNELKENGDNPLEYIKELNVLAHQEDPTRPTTSASNQGGAINFITDNIAWNRYDGWYGATPATLASWLDKTHQAHPEIKI
AISEYGAGASIYHQQDSLVQTSPGSWWHPENWQTEYHIQNWKIISERPYVWGSFVWNMFDFGAAHRTEGDRPGINDKGLVTHDRKVKKDAFYF
YKANWNPEPMVYIAGRRSVNRVKPVTEVQIFSNCAEVTLKVNGQIIKK

***B.dorei* GUS protein**
MKRFAGWLLFFWGCICCICASEISITDSWKYKAENDERFSSMDWNDSDWVTVDLPHTWNAGDVIDEQRGYRRGISWYRKKLFIPSEARDKKITL
RFDGVASKADVYLNGKLLKTHLGAYTAFGVDITDICEVGKENLLAVKVDNSSSLGEILPPVSGDFSIFGGIYRRVFLQWTEKVHFVTEPYAAVPVRI
QTPEVSVSEASMQIVAFLKNDFTDTKHVHVNVFLCDEMNRIVKEKQLKLKLIPGRKYPISTSVGRIENPHLWSPELPYLYTVKVQVCDAKNGEMYQ
EVISPVGFRWFSVDKTGFYLNGKYLKLRGAARHQDYAGLGTAIPVEMNRRDMRLLKEMGANFVRISHYPQDPEIYRACDELGLIVWSEICVVNEV
RKNTAFAHNCKEMLKEMILQNYNHPSVVLWGAMNELWDYHKQAIALARELEALKKELDPYRLSCVAFHAFTWEKPYTQSSKEMFSISDVNGVNV
YESWYQGDSATIAPMFDKFCSYSTAKPRFLSEFGAGSDERIHSYTPRTFDFTPEFQLDFNRRYINEMEKRPDYIGYSIWNLVDFQVDGRGDSKPN
LNQKGMLTEDRRKKEIYYYCQARWSDIPMIHIAGADWTKRVEICDDSINVRKISVFSNQKTVELIHNGKSLGVREVVNGEAVFAVPFINGENLLDAR
SGALSDRLKIQMKLLSSRLTDSDVLLDGLCINLGQEHCYFIDPQLQEIWIPDKPYTKGSWGYMDGKPFNSWPGSSHDGVRYGVGADIKNTFLEPL
FQTFLIGTTCYRLDVPDGVYEIGFYFTEPFSKDERKNIVRTGVSAEGQRVFDVSVNGEKLIDSLNLADSYGEQTAVVKTLVVNVRNHEGLEILLSPQ
KGQGVISGLKVKKIR

***P.copri* GUS protein**
QRKAATINDNWEFKLPTSQKWTSVNIPHTYTLDAYQGRNYYKGKAEYRRILTLPEINPDRRYFLKIDAANKAAEVKVNGKEVGCHAGGYSSFTFDI
TDFLNTNPARQGEKSENTIEITVDNSRPDVTPIMADFTFWGGIYRDVWLVSTPDIHFNMLNMGSDGIFVSTPIVNEKQSAVKVVSEVSNDGQKAS
MVELRNEIFSPDGKLLQTIKKRITLKAGETQRTALQSKPIANPLLWTPERPTLYKVKTSIIDTKSGKVIDEKNHKVGFRWFSFDGEKGFCLNGKTYKL
RGFNRHQDQAPVGVALPDEAHRRDIKLMKELGSNYIRISHYPQDDALLDACDELGLLAWEEIPIIDLVPDTPHYADNCERNLREMIRQHYNHPSIIN
WGYMNEILLCTPWPGTKEWPAFKERTLALAHRLEKVLKDEDPTRKSVMAFNMTNTYNEIGLNLVDVVGWNLYHGWYQGELNGFNHWCEDQHQ
RYPKKPMIISEWGAGSDQRLRSNSAHAFDFSIEYQQTYIEHYLPFIEEKPWISGCTYWNFIDFNVAERQESMPRVNNKGIAYNDRTLKDVAYYFKS
MWRKDIPVVHIASRDWSIRTGHINEPQRIKVYSNMPEVELIVNGRSYGKKSVQNCFAVFDVVLPFGSSTLEAKGFNEVLTDYKNKVDGNTGDVMK
IQYNPLPNLAKGEELAINVGSNCYFISSLSQLTWLPDQAYKPGAWGYVGAESKSTTSEIENTIDGPIYQTWREGDLEYRIDAPCGEYEVELLMADV
TKPAVQLPNLLAKSNTESSSKDVRFDVSINDERKESDFTPTDGRHYRTAFKRKYIVENNRGSINIQLKSLQGKAFLNGIKIRKLN

***R. hominis 1* GUS protein**
MRNTIVLEKDWTFYKNPQSESGEAVTLPHTWNAVDGQDGGNDYYRGTCKYVRHFAKPELEKGGRAYLEFNGAAMTADVVVNGTKLFHHEGGF
STFRVDVTEQLTEDNLLEVYVDNSDNTKVYPQKADFTFYGGLYRMVKLVTVPKVHFVMDYAGGNGMKVTPEVTILDAAEKQADADVTVELWMT
GEATDVTVAVAGETQTVPVENGYARAVFALKNVHLWDGVDDPYLYTAKAELPGGDVVERTFGCRSFKVDSREGFFLNGRSYPLRGVSRHQDR
AGAGNALTYEMHREDMAIVRELGANTIRLAHYQHAQEFYDLCDENGIIVWAEIPYITMHMADGTENTLSQMKELIVQNYHHPSIVCWGLSNEITAA

SAVNEELLENHRRLNDLCHELDKTRPTVMADVFMLETDSPMLEIPDMNSYNLYFGWYIGELDQNDSFFDEYHSTYPDRVIGLSEYGADANPAYH
SANPERGDYTEEYQCVYHEHMAKMIEERPYLWATHVWNLFDFAADGRDEGGRHGENQKGLVTMDRRIKKDAFYVYKAYWSKAPFVHLCGSRY
TDRAEDVTEIKVYSNQKKVSLFVDGAEKETKEGARIFRFRVPITGTHTIRAVSGDCTDEITVRKVDEPNPDYIFNKQGDVVNWFDKEDFKADHYSI
SDTLGELAKNEMANAIVQSLMAQASASRGDVAESVKDNPALQRMMQRMTLASLLKQAGDAVSEEQMKALNDALQKIPKN

***F. prausnitzii* GUS protein**
MLYPEQNEARLKLSLDGTWAFALGSCAETQFDPAKPLPDAQPIAVPASYNDQNDQTTALRRHYGWVWYQRKVTLPAFCAGQRVVLRFGSVTHT
AKVWLNGQLIAQHKGGFTPFEADVTALLQPGETALLTVACDNRVNHSTLPVGNEDGQLAFFGSDNAGIPSVEAAKRAAAPQNRPNFDFFNYAGI
HRPVVLYTTPKEYIEDVTIVPAVDGTVQYAVKTTGSAPVRVTVLDADGNAVASAESAEGTITIPEVHLWEPRPGTPYLYTLHATCGADVYDQTFGV
RSIEVRGTQVLLNGKPLYFKGFCKHEDFTAHGRGFDPVLNVKDVNLIHWANANAVRTSHYPYAEEFYDLCDREGILVMDETPAVGIGGGAAVNP
YKEYPLAEHHRQVLAEMIHRDKNHPCVVLWSLGNEPNLEHFPQDAYDYWHPLYELAHQLDPQDRPVTLVCCQNDYTKDITTRTMDIVCINRYYG
WYNLSGDMDAACYGLNQELDFWAEQHKPVMMSEYGADTVAGLHTAGAEMFSEEFQVEFYRRLDAEFDKRPWFVGEFVWNFADYDTVQGPM
RVDGNKKGLFTRDRRPKLGMHFLRQRWAEIPTFGFK

***R. hominis 2* GUS protein**
HHHHHHSSGVDLGTENLYFQSNAMREVINFNTKWAFTKEATEVPKEMPEKWYWVTLPHSWNEIDGQDGGNDYYRGTCYYAKQLKKSELPEAD
CYYLELRGANASADVYVNGKAVAHHDGGYSTWRVDITKELTEEENLIVIAVENGVNDRVYPQNADFTFYGGLYRDVNIIAVNKSHFDLDYYGGPG
IKVTPEIKGADASVEVEVFLTNAAADQKLVYTVKDAEGKEVAKTETAAGETKAVLSIPAVHLWNGKKDPYLYTAEVALVSGEEAVDAVSTRFGCRT
FEIDPERGFILNGEEYPLRGVSRHQDRWGIGNALLPEHHREDIDLICELGATTIRLAHYQHDQYFYDLCDERGLVIWAEIPYISSHMPNGRENTISQ
MKELVVQNYNHPSIVVWGLSNEITMAGSSDEDLLENHRILNDMVHEMDHTRLTTIAVVSMCDIHDPYIQIPDVISYNHYFGWYGGDVSMNGPWM
DNFHKEFPNIPLGMSEYGCEALNWHTSDPKQGDYTEEYQAYYHEEMIKQLFTRKYIWATHVWNMFDFGADARNEGGENGQNHKGLVTFDRKY
KKDSFYAYKAWLSDEPFVHLCGKRYVDRVEDTTKVTVYSNLPEVELFVNGKSAGKLQAEDHFFHFEVPNVGESTLVAVAGEYKDESHIRKVDTF
NEEYSLKESGAILNWFDITEPEGYYSLNDRLSDIMKSEEGKALFMGLMSKVAAGMSQGNEKNDGNPAAGAMANPKMLEMLGGFTVIRMINLMGA
AGPKVEWKKEDLLGLNAQLNKIKRVD

***Bifidobacterium angulatum* GUS protein**
MRRKYFASIVAVAMLAAGVPCAQAEELDANTAQTTDSQIVQNETQNNGSSNEAAESALNASADNQIAGRTVENIDKGWTFSKNDASMEGWTFP
TGASEGTIDLPHSWDYAHPTMSYIPENNRKTVTYSKQLDVAKYHGKNLFIKFYGSNKNTTVKVDGQEVGTHVGGYSAFIFDLTKYVQDKDSVALT
VDVTNVDTVSIPINVDYTQFSGIYRDVELIALPNQYISTENKGSSGVFVDYKLNGNNASVNTRVDVTNKATEAANLVLKTTISDNAGNVVSEQSSDI
QVSAGTESAEQKLDQQLTNVHRWNGRTDPYLYTMNVSLQDAAGHVLDTESTKIGFRTFKVSNGKAYLNGKQIEIHGVGYHQDREGVGNAVSRD
QMAQDIDTMLDMGVNAVRTSHYPHDPAFYEMADEKGLLVYCEIPYYLIYSKADSYKNSITNQLTEMIRQGYNYPSIVMWGVQNEVRYSEQFASY
GPDFKVTEDELVTFNSALVDLAHQEDPNRLIVQANIDGADAVNTSAKWSSKIDLTGMNLYVGFKSPVRNADAAGHKKLVESLTNKMNNYQQVLG

ADSMMLSEYGAGANIDQHTEVDGSFSWNGASDANGDKHYEEYQSYLLEAYWDYIQHSTNVAASFVWNMFDFSSYRNAGGKERLNTKGLLCYD
HVTKKDAYYFFKANWNKSDKFVYLTSKRFTQRNKPTQQIKAYSNCDNAELFLNGKSLGAGTKQQDGVFVWDNVKLAGQVENSIKVVAHDGSKT
YEDAVDGVTYGMQFEDVNANTPHVEDIQWLADNGVTEGWVDSTGKRTFRGMDTVKRQDMAAFLYRLAGSPDYTPSASDKSRFTDVTEDTPHA
KEIWWLGTNGIAEGWDDGSFRGMDTVKRQDMAAFLKRLATKNLGVKDSSYNRNPFADVNKRTPHYKEILWMAGTGISEGWTEANGTKTYRGM
SDVVRQDMAAFLHRLGNYANTGSVES

***P.merdae* GUS protein**
MKYLFVACLLCLSVLSAVAKVPAMNKIRLTNNWEYLKGDLGGIWEAVRPAAPGSSEAVPIWQPVTLPHCFNAEDAVDPDVNYYEGPGWYKTLLAI
DNPYRNGRIVLDFDGAGQKTDVYVYTTHVGSHVGGYDSWNVDITDAVKAFLGSKDAERFKGKVPLSIRCDNSRDLEMIPSDLADFNIYGGLYRYL
NLVYLPEVSFEQIHLESSLSSNLKEGILKVKTSFYNPEDIRKADVTVSVYDVDRKPVFSKTLEGILPLGDQLLAKMKIKNPVLWDVDVPQLYTCELTV
KTPDQTFTTEERFGFRHTEFKDKGPFFLNGKRLLLRGTHRHEDHAGVAQAMTEDMMRREMRMMKDMGVNFIRLGHYQQSEIILDLCDELGILV
WEEIPWCRGGLGGDVYKKQARRMLANMIVQHHNHPAVIIWGLGNENDWPNDFNTFDKSAIRAFMKELHDMAHRLDDTRMTAIRRCEFCNDIVD
VYSPSIWAGWYRGVFTDYKSISEQEMQKVKHFLHVEWGGDSHARRHSEDAFYNLKNIEAGKGGDERAGDASLYGGVPRASRDGDWSESYVV
RLIDWHLKEQETMPWLTGTAYWPFKDFSTPVRPDNPVPYVNQKGVVERDFTPKESYYVFQSYWTEKPMIHIYGHTWPVRWGGKDDRKEILVYS
NCDEVELFVNGVSQGVKRRNSQDYPAAGLRWNCVYQEGMNEIRAVGVKKKEKKEVSDVIRQEYQTAKWDKEAACQVSLLSEEGDTALVQVQLI
DKNGIRCLSSKKQITFEIAGDGSLICNLGTSTGSRKVQAYNGRALIRIKRNEGNSVVAVKSEGLPTAFLELKSPK

***B.ovatus* GUS protein**
MKNRIIILCLVCLCLVNIGLFAQETSPRTIFSLNEGWECRPITTVNRKAPFTPVTIPHTWNTSYIEGTTLYERKMMVYQRPLVVTKAMKNKRLFLYFE
GVNSAAQVFMNRRTVGEHLGGYTAFCIEITDEVKEGENLLEVWASNAYRTDILPVSGDFNVNGGIHRPCHLIVTGQDCISPLFYASPGVFIHQENI
SKTVADVNVETHLSLKNKKQGLRLKTTVADADNKTVASNEVEVSDVIVKQPMKIHRPILWDGKKNPYLYTVTVELYDGNLLKDRMVQRTGFRYFS
VDHEKGFFLNGEYLNLYGFCRHEDAVGRASALLPEDYRMDMELIKESGATAMRLAHYPHAEPMYDLSDENGIILWTEIPMCGPGGQAFTGFVDT
EGYKDNARLAVKELVYQKFNHPSICFWGICNEILVSDGKRFVEYDNPIPFIKELNGIYKSIDSSRLTALATCVDQSYYLGCSDLIAWNKYFGWYKDA
APSASKFFDDCRDSSKGIPVGVSEYGGGASINHHQWPLAMEDRSDSHFHPEEAQTFCHEGNWESFAKRPYLWAKFIWVFADFPSYMRQEGEK
DGYNDKGLVTHDRKTKKDAFYFYKANWNPEPMIYITSRRFTKRDNPKTDIKVFTNLKEATLYINNRKIGTMKPDEMNRVIWKDIRLNDGRNIICVEG
KNGKGLLSDTCEWYCIK

***Parabacteroides MSP* GUS protein**
MNKRVLSLTLLLACLGLMTMQAEGRKVESFNSGWSFKKAPAEKELAINAPKWDKGWSEVEIPHTWNAKDMQVQANSFYEGAAYYKKQYFFPAE
LKDKRVFLRFEGVGSCAEVFVNGMLATSHKGGYSAFACEISPLLKAGEENEIIVKADNKSRPDVIPVNHNLFGVYGGIYRPVWLVVTEPCNISVTD
CASPGVYVTQKNVSKKQADVKVKVKLDNGTLQPVPVTLQNTIYDQEGKQVATHSQSFELSAQGEQAYESSFTIKKPTLWQGRENPYLYKVVSRL
IKDGQVIDEMVQPLGLRKYEIVAGKGFYLNGEKYPMYGVTRHQDWWGLGSALKNENHDFDLATIMDVGATTVRFAHYQQSDYLYSRCDSLGLII

WAEIPFVNRVTGQEAENCRNQLREMIRQSFNHPSIYVWGLHNEGATTVRFAHYQQSDYLYSRCDSLGLIIWAEIPFVNRVTGQEAENCRNQLRE
MIRQSFNHPSIYVWGLHNEVYQPHQYTKELTQSLHDLAKTEDPDRYTVSVNGYGHMEHPVNLVADIQGMNRYFGWYEKKIQDIKPWVENLEKEY
PHQKLMLTEYGADANLNHQTEYLGDALNWTKEFYPETFATKTHEYQWSVIAAHPYIIASYLWNTFDFCAPMWVRGGVPARNMKGLVTFDRKIKK
DSYFWYKANWSKEPVLYLTQRRNWDREKKETSVTVYSNIGTPKVYLNGKELTGIREGYTPVHYIIDNITLDMGKNIVKTVVVKDGKTYEDEIEWVY
NGEKKRDSDQSVNKEEHAGF

Table S4: Correlation analysis of Rosetta-calculated structural features and experimental GUS activity

Rosetta Metrics	Spearman ρ	P value
dSASA polar mean	-0.688	0.003
fa elec mean	0.591	0.016
fa sol mean	-0.553	0.026

Note: Table shows Spearman correlation coefficients (ρ) and P-values for the top three features that significantly correlate with PGE_2 production from PGE_2-Acyl-GlcA substrate across 18 GUS isoforms. dSASA_polar_mean represents the mean change in polar solvent-accessible surface area upon product binding; fa_elec_mean represents electrostatic energy; fa_sol_mean represents solvation energy. Negative correlations indicate that lower values of the feature are associated with higher enzymatic activity.

Table S5: Analyte retention times, ionizations, internal standards and the range of the standard curves of the LC-MS/MS lipidomics method

Compound	Precursor (m/z)	Product (m/z)	RT (min)	ISTD	Low Standard (pg/µl)	High Standard (pg/µl)
6-keto-$PGF_{1\alpha}$	369.2	163.1	2.39	PGE2-d9	0.1	120
TXB_2	369.2	169.2	3.21	PGE2-d9	0.025	30
PGB_2	333.2	175.2	5.21	PGE2-d9	0.1	120
PGD_2	351.2	271.1	3.96	PGE2-d9	0.025	30
PGE_2	351.2	271.1	3.75	PGE2-d9	0.025	30
8-iso-$PGF_{2\alpha}$	353.2	193.1	3.21	PGE2-d9	0.05	60
$PGF_{2\alpha}$	353.2	193.1	3.65	PGE2-d9	0.05	60
PGD_3	349.2	269.2	3.31	PGE2-d9	0.025	30
PGE_3	349.2	269.2	3.16	PGE2-d9	0.025	30
11-dehydro-TXB_2	367.2	161.3	3.73	PGE2-d9	0.025	30
15-ketoPGE_2	349.2	113.1	4.8	PGE2-d9	0.025	30
15-keto-$PGF_{2\alpha}$	351.2	219.2	3.85	PGE2-d9	0.025	30
19,20-DiHDPA	361.2	273.2	6.77	11,12-DHET-d11	0.1	120
16,17-DiHDPA	361.2	233.1	7.06	11,12-DHET-d11	0.1	120
13,14-DiHDPA	361.2	193.1	7.18	11,12-DHET-d11	0.1	120
10,11-DiHDPA	361.2	153.1	7.35	11,12-DHET-d11	0.1	120
7,8-DiHDPA	361.2	189.1	7.75	11,12-DHET-d11	0.1	120
17,18-DHET	335.2	247.1	5.89	11,12-DHET-d11	0.1	120
14,15-DHET	335.2	207.1	6.13	11,12-DHET-d11	0.1	120
11,12-DHET	335.2	167.1	6.23	11,12-DHET-d11	0.1	120
12,13-DHOME	313.2	183.2	6.29	11,12-DHET-d11	1	1200
9,10-DHOME	313.2	201.1	6.5	11,12-DHET-d11	1	1200
14,15-DHET	337.2	207.2	6.75	11,12-DHET-d11	0.1	120
11,12-DHET	337.2	167.2	7.09	11,12-DHET-d11	0.1	120
8,9-DHET	337.2	127.3	7.4	11,12-DHET-d11	0.1	120
5,6-DHET	337.2	145.1	7.84	11,12-DHET-d11	0.1	120
19,20-EpDPE	343.2	281.1	10.34	11,12-EET-d11	0.2	240
16,17-EpDPE	343.2	233.1	10.78	11,12-EET-d11	0.2	240
13,14-EpDPE	343.2	193.1	10.89	11,12-EET-d11	0.2	240
10,11-EpDPE	343.2	153	10.98	11,12-EET-d11	0.2	240
7,8-EpDPE	343.2	189.1	11.17	11,12-EET-d11	0.2	240
17,18-EpETE	317.2	259.1	8.94	11,12-EET-d11	0.1	120
14,15-EpETE	317.2	207.1	9.42	11,12-EET-d11	0.2	240
11,12-EpETE	317.2	167.1	9.5	11,12-EET-d11	0.2	240
12,13-EpOME	295.2	195.2	10.34	11,12-EET-d11	0.5	600
9,10-EpOME	295.2	277.3	10.54	11,12-EET-d11	0.5	600
14,15-EET	319.2	219.3	10.52	11,12-EET-d11	0.2	240
11,12-EET	319.2	179.2	10.9	11,12-EET-d11	0.2	240
8,9-EET	319.2	151.1	11	11,12-EET-d11	0.2	240
5,6-EET	319.2	191.3	11.17	11,12-EET-d11	0.2	240
22-HDoHE	343.2	269.1	8.07	d8-15-HETE	0.025	30

17-HDHA	343.2	281.2	9.04	d8-15-HETE	0.025	30
20-HEPE	317.2	243.2	7.08	d8-15-HETE	0.025	30
18-HEPE	317.2	161.4	7.27	d8-15-HETE	0.025	30
13-HODE	295.2	195.2	8.47	d8-15-HETE	2.5	3000
9-HODE	295.2	171.2	8.59	d8-15-HETE	2.5	3000
20-HETE	319.2	245.1	7.8	d8-15-HETE	0.5	600
19-HETE	319.2	231.1	7.68	d8-15-HETE	0.5	600
15-HETE	319.2	219.1	8.87	d8-15-HETE	0.1	120
12-HETE	319.2	179.2	9.47	d8-15-HETE	1.25	1500
11-HETE	319.2	167.2	9.22	d8-15-HETE	0.1	120
8-HETE	319.2	163.3	9.47	d8-15-HETE	0.1	120
5-HETE	319.2	257.3	9.47	d8-15-HETE	0.1	120
Lipoxin_A_4	351.2	115.1	4.43	d8-15-HETE	0.025	30
Lipoxin_B_4	351.2	221.1	3.96	d8-15-HETE	0.025	30
LTB_4	335.2	195.3	6.11	d4-LTB4	0.025	30
20-carboxy-AA	333.2	271.2	7.31	d4-LTB4	0.025	30
DHA	327.2	283.2	12.69	ARA-d8	15	18000
EPA	301.2	257.2	12.28	ARA-d8	15	18000
LA	279.2	261.2	12.86	ARA-d8	15	18000
ARA	303.2	259.2	12.8	ARA-d8	15	18000
PGE_2-d9	360.2	280.1	3.72			
11,12-EET-d11	330.2	179.2	10.9			
11,12-DHET-d11	348.3	167.1	7.02			
d8-15-HETE	327.2	226.1	8.77			
d4-LTB4	339.2	197.1	6.08			
AA-d8	311.2	267.2	12.79			

Note: Details of reference standards and internal standards used to assess lipid mediators' concentrations. Ionization masses (Precursor and Product) in mass/charge ratio (m/z), retention time (RT), internal standard (ISTD) used, and the minimum and maximum concentrations used in the standard curve (pg/µl injected). Analyte quantification was determined using standard curves determined based on peak area analyte/peak area of indicated ISTDs.

Table S6: Sequences of primers used in qRT-PCR

Gene name	Forward	Reverse
Gapdh	AGGTCGGTGTGAACGGATTTG	TGTAGACCATGTAGTTGAGGTCA
Pla2g4a	CAGCACATTATAGTGGAACACCA	AGTGTCCAGCATATCGCCAAA
Cox-1	CGATCTGGCTTCGTGAAC	GAGCTGCAGGAAATAGCC
Cox-2	TTCAACACACTCTATCACTGGC	AGAAGCGTTTGCGGTACTCAT
Ptges	TTAGAGGTGGGCAGGTCAGA	CCACTCGGGCTAAGTGAGAC
Ptgds	GTCAGTCAGAGGGCTGGTCAC	GGACTCTTATCCTTCTCCTCACG
Ptgfs	TGATGAGAGCAAGCAAATCT	ACAGTACCTTGTCGCCAC

Table S7: Strains and plasmids for construction of *E. coli* Δ*gus*

Strains or plasmids	Description	Source or reference
E. coli DH5α	Plasmid construction	Lab storage
E. coli MG1655	The host used in this study	Lab storage
p15ALacCas9	Inducible expression of cas9 and λ-Red recombination systems	Lab storage
pTarget	Constitutive expression cassette of gRNA	Lab storage
pTarget-*gus*	Derived from pTarget, target *gus* gene in *E. coli* MG1655	This study

Table S8: Primer's sequence for construction of *E. coli* Δ*gus*

Name	Sequence (5'→3')
YW1014	CTGCGCAACGATACGTACCACATTCTCACG
YW1015	CGCCAGCACAATTGTCCAGAAATGG
YW1016	GGAGTCCCTTATCAACAACTCTCCTGGCGCA
YW1017	AGTTGTTGATAAGGGACTCCTCATTAAGATAATAATACTGGTCAACC
YW1018	CATATGACGATCACGGAATTTGTTGCCCA
YW1019	CGATTGAAGGGATTCATTTCGTTGACTATATGGTCGAGT
YW1020	CCAAAGCCAGTAAAGTAGAAGTTTTAGAGCTAGAAATAGC
YW1021	TTCTACTTTACTGGCTTTGGACTAGTATTATACCTAGGACTGA
YW1022	GCGGTATTTTCTCCTTACGC
YW1023	GCAGGTCGACTCTAGAGAAT

Table S9: Antibodies used in Flow Cytometry

Antibodies	Source	Identifier
Anti-mouse CD3 – BV711 (clone 145-2C11)	BioLegend	100349
Anti-mouse CD4 – BV510 (clone RM4-4)	BioLegend	116025
Anti-mouse CD8a – PerCP/eFluor710 (clone 53-6.7)	Invitrogen	46-0081-82
Anti-mouse CD11b – APC (clone M1/70)	BioLegend	101212
Anti-mouse CD11c – FITC (clone N418)	BioLegend	117305
Anti-mouse CD25 – PE (clone PC61)	BioLegend	102007
Anti-mouse CD45 – Alexa Fluor 700 (clone 30-F11)	BioLegend	103128
Anti-mouse CD45R/B220 – Pacific Blue (clone RA3-6B2)	BioLegend	103227
Anti-mouse CD86 – BUV395 (clone GL1)	BD Biosciences	564199
Anti-mouse Foxp3 – PE/Cy5.5 (clone FJK-16s)	Invitrogen	35-5773-82
Anti-mouse γδTCR – eFluor 450 (clone eBioGL3)	eBioscience	48-5711-80
Anti-mouse Ly-6G – PE/Cy7 (clone RB6-8C5)	eBioscience	25-5931-82
Anti-mouse MHC-II (I-A/I-E) – BV650 (clone M5/114.15.2)	BioLegend	107641
Anti-mouse Rorγt – PE-CF594 (clone Q31-378)	BD Biosciences	562684

Raw spectrometry data from liquid chromatography–high-resolution mass spectrometry (LC-HRMS) and triple-quadrupole liquid chromatography–tandem mass spectrometry (TSQ LC–MS/MS) experiments

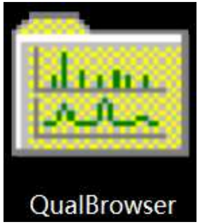

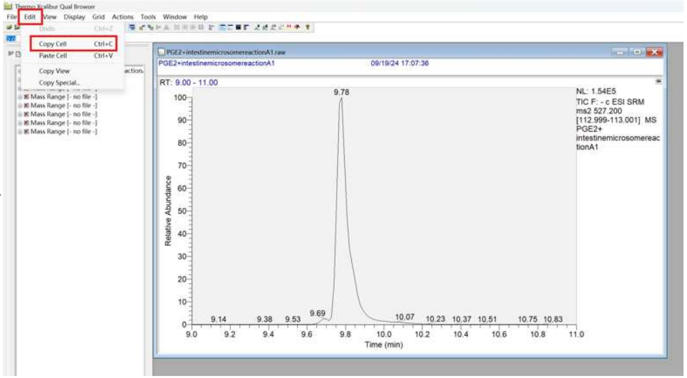

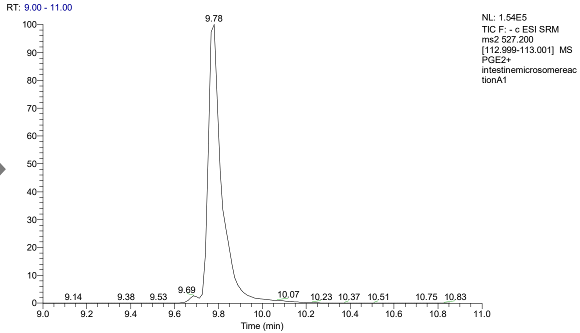

Raw LC–MS/MS data were opened in Thermo Xcalibur Qual Browser. After setting the appropriate visualization parameters, peak intensity values were exported by selecting "Edit" → "Copy Cell" and pasted into slides.

Fig. 2a
LC-HRMS spectrometry of liver microsome and intestinal microsome reaction

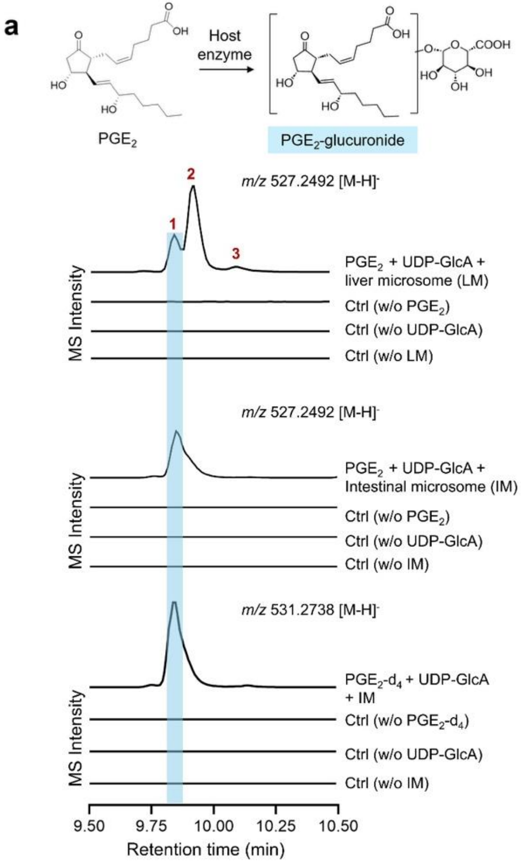

Fig. 2a Upper

PGE_2 + UDP-GlcA+liver microsome (LM) Duplicate 1

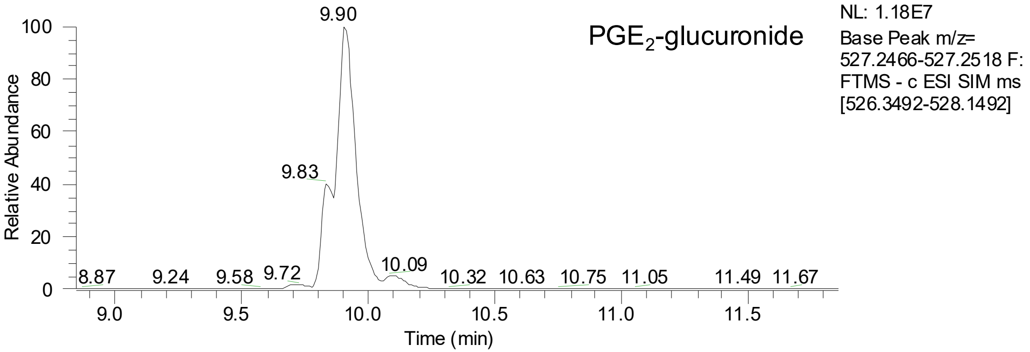

PGE_2 + UDP-GlcA+ liver microsome (LM) Duplicate 2

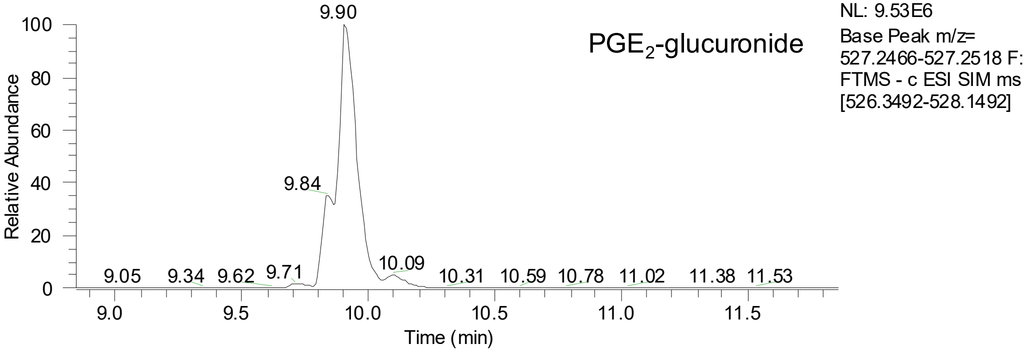

Fig. 2a Upper

Ctrl (w/o PGE_2) Duplicate 1

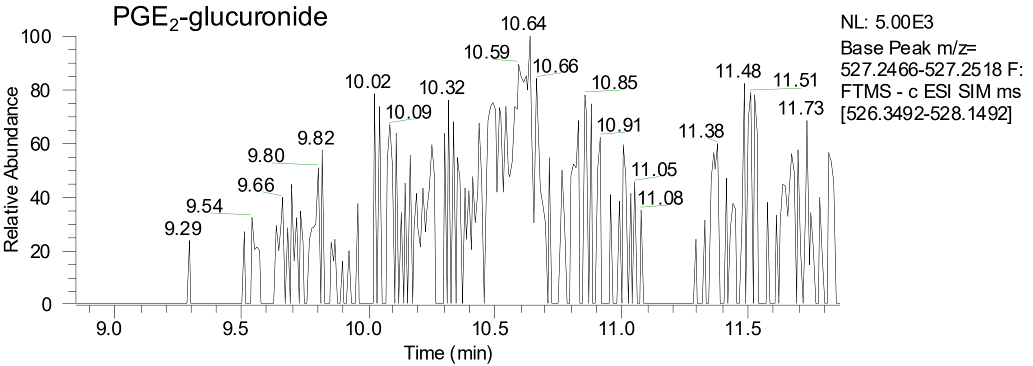

Ctrl (w/o PGE_2) Duplicate 2

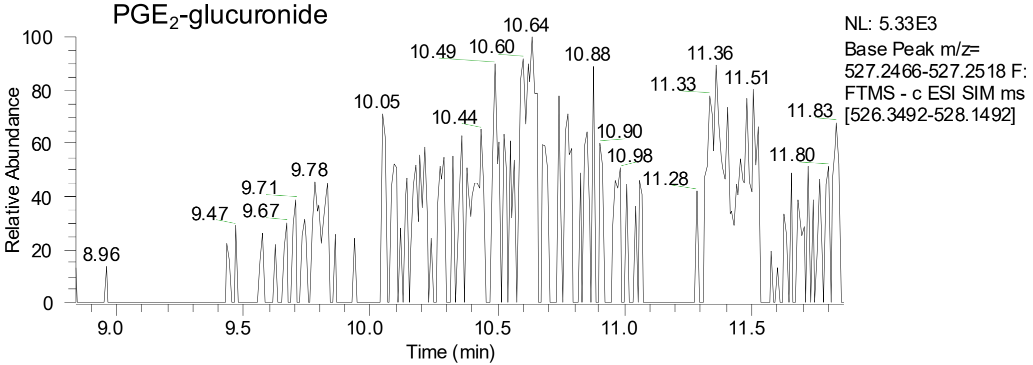

Fig. 2a Upper

Ctrl(w/o UDP-GlcA)
Duplicate 1

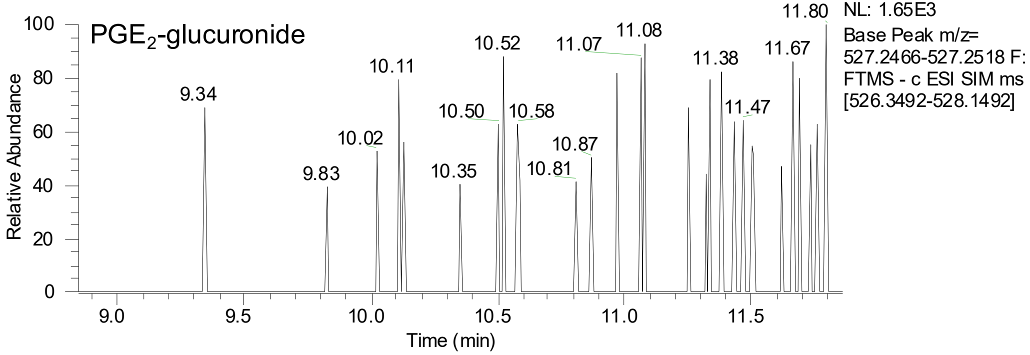

Ctrl(w/o UDP-GlcA)
Duplicate 2

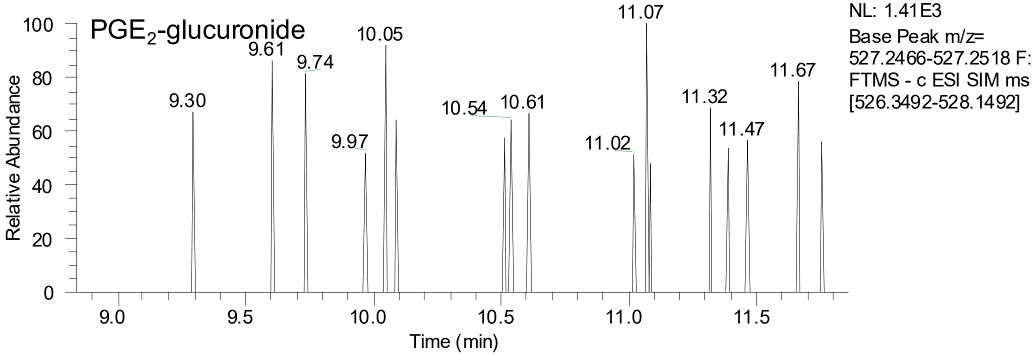

Fig. 2a Upper

Ctrl (w/o LM)
Duplicate 1

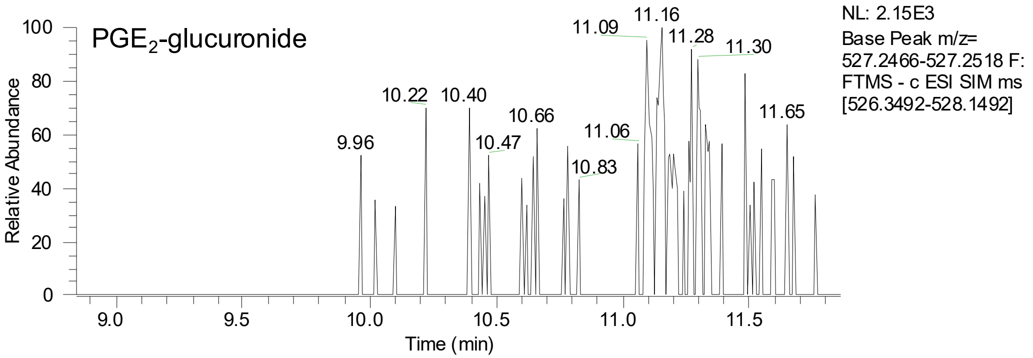

Ctrl (w/o LM)
Duplicate 2

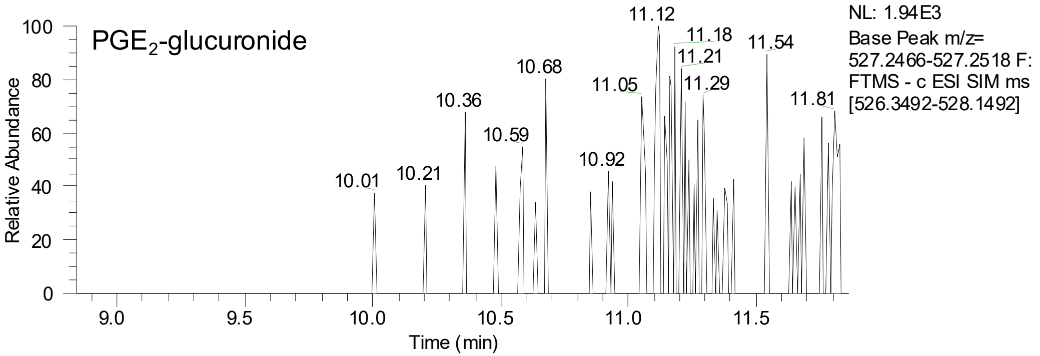

Fig. 2a Middle

PGE_2 + UDP-GlcA + (Intestinal microsome) IM

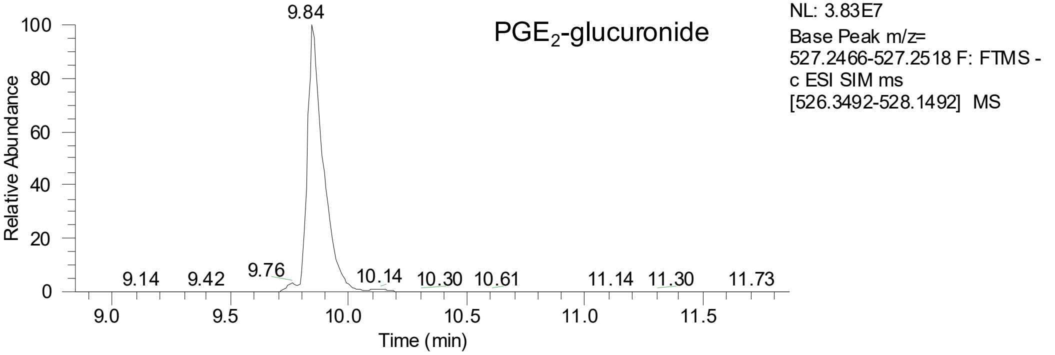

Ctrl (w/o PGE_2)

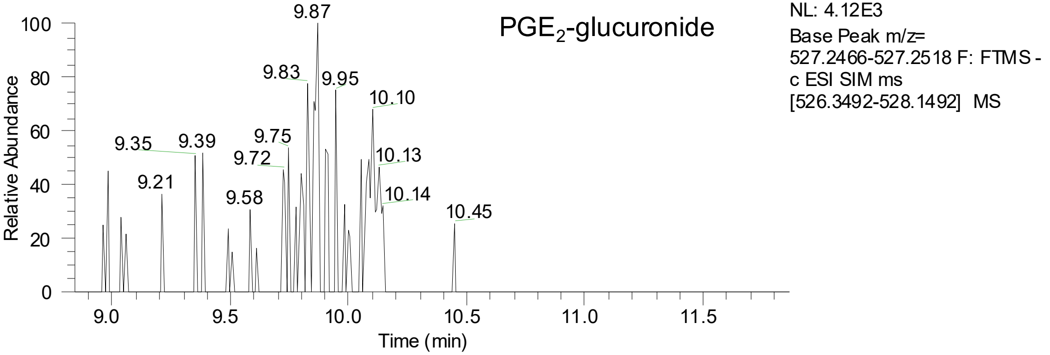

Fig. 2a Middle

Ctrl (w/o UDP-GlcA)

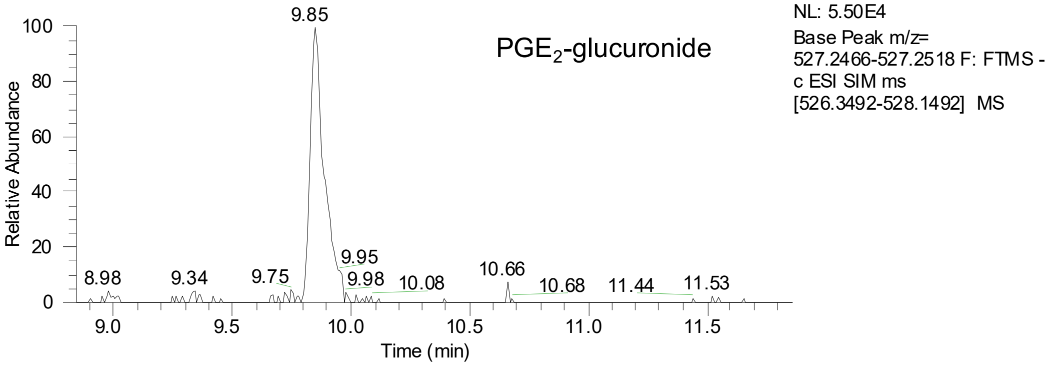

Ctrl(w/o IM)

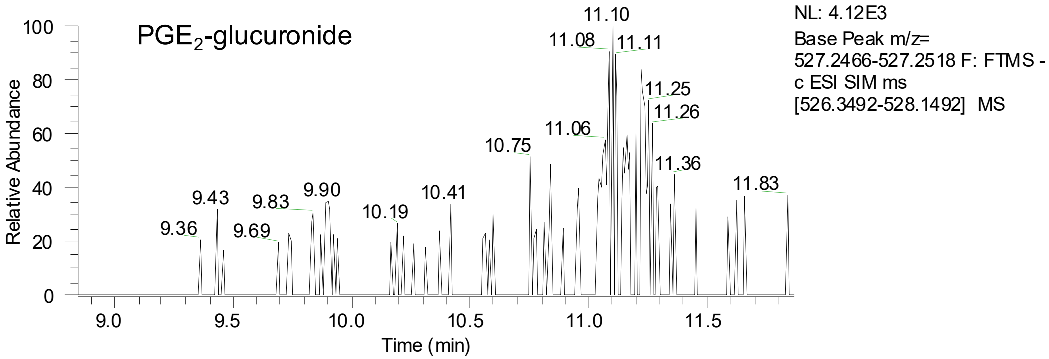

Fig. 2a Bottom

PGE$_2$-d$_4$ + UDP-GlcA + IM

Ctrl(w/o PGE$_2$-d$_4$)

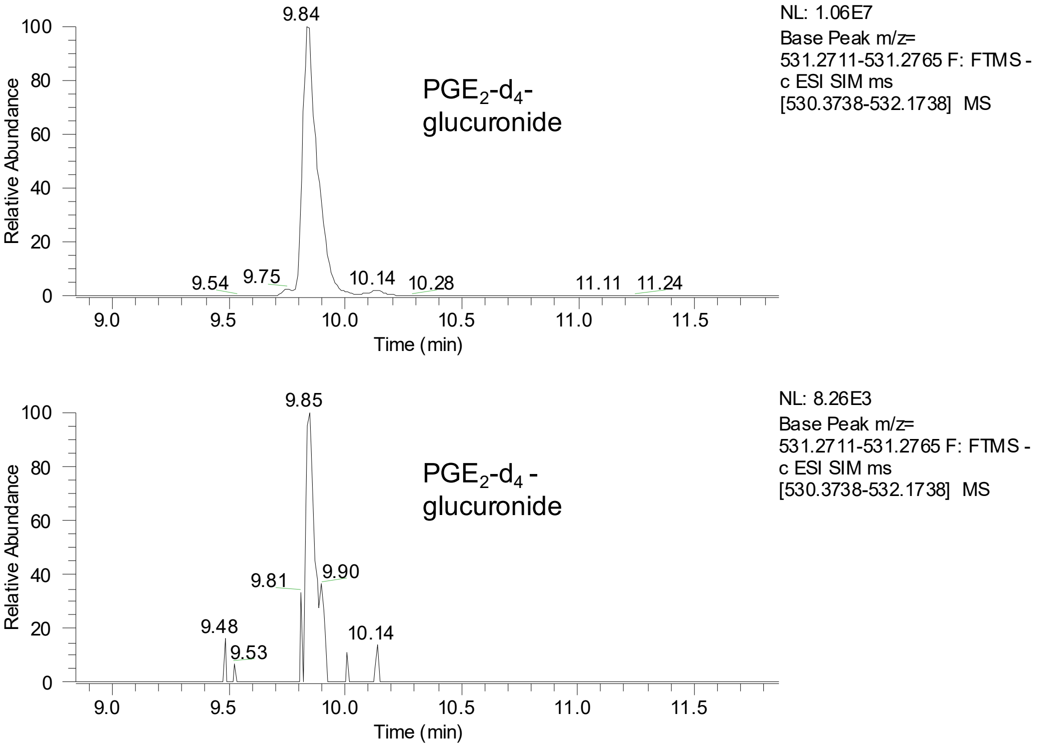

Fig. 2a Bottom

Ctrl(w/o UDP-GlcA)

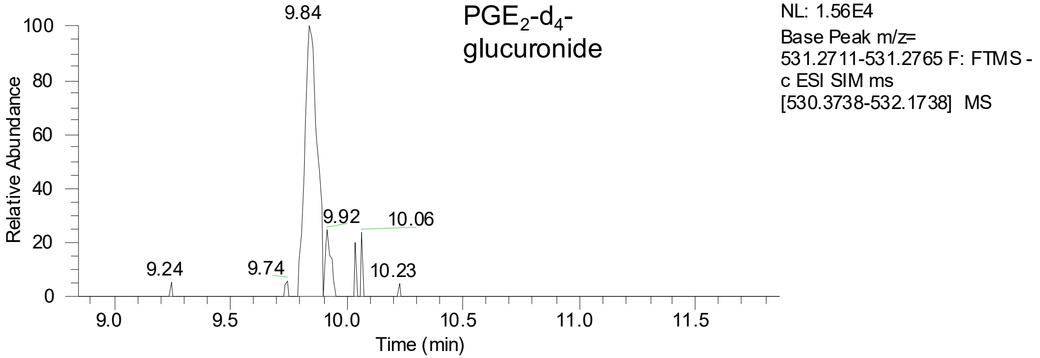

Ctrl(w/o IM)

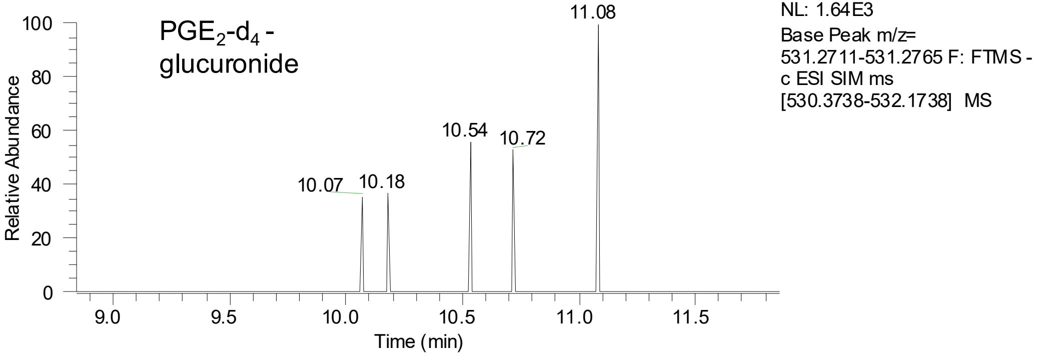

Fig. 2b
LC-HRMS spectrometry of incubation of intestinal microsome produced PGE_2-glucuronide and mouse gut bacteria

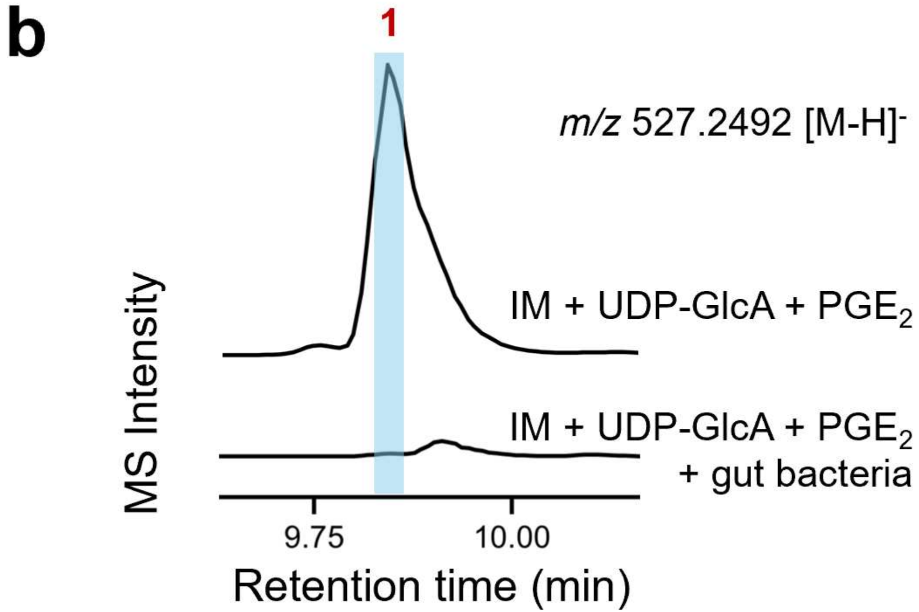

Fig. 2b

IM + UDP-GlcA+ PGE_2

IM + UDP-GlcA + PGE_2+ gut bacteria

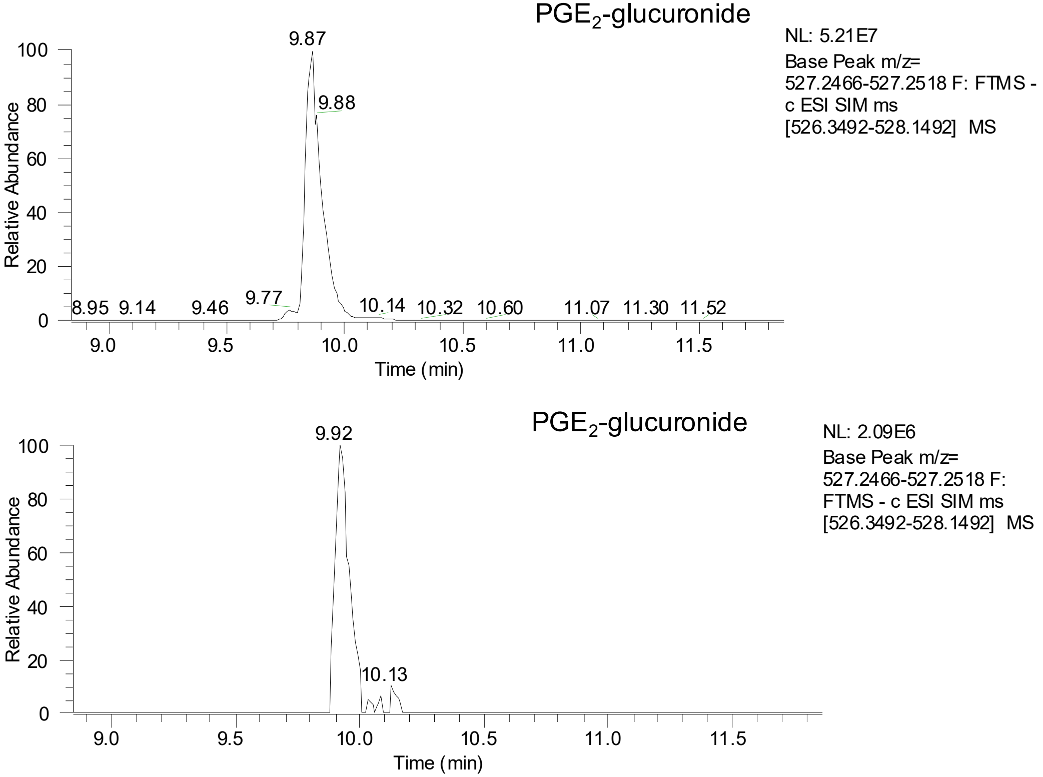

Fig. 2c
TSQ LC–MS/MS spectrometry of PGE_2-glucuronide in the colon tissues of GF and SPF mice

c

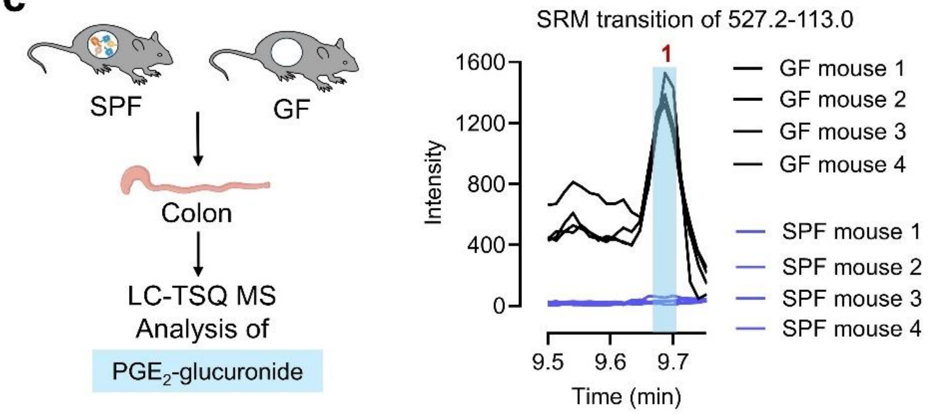

Fig. 2c

Colon-GF mouse 1

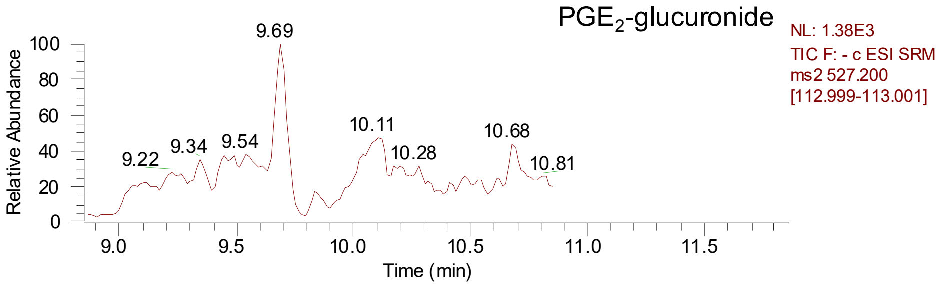

Colon-GF mouse 2

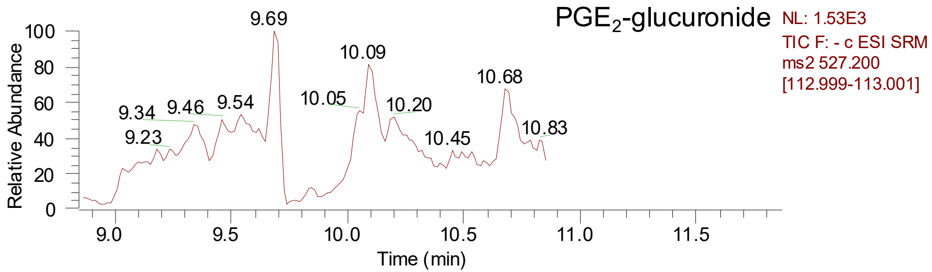

Fig. 2c

Colon-GF mouse 3

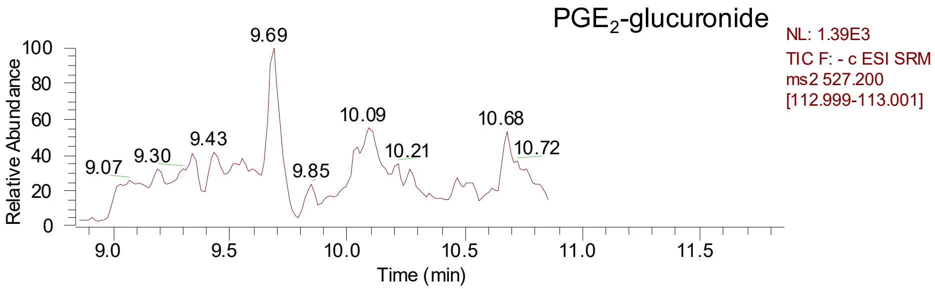

Colon-GF mouse 4

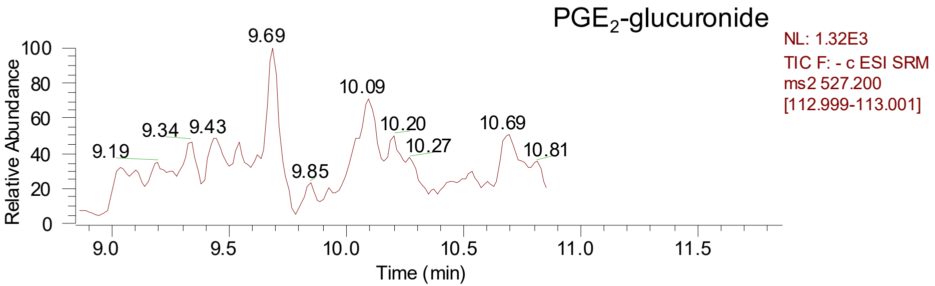

Fig. 2c

Colon-SPF mouse1

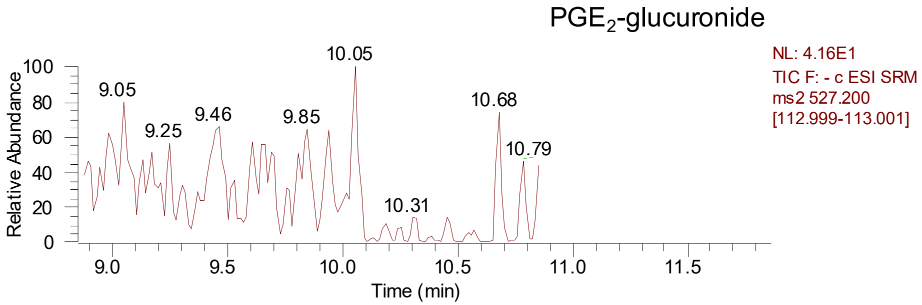

Colon-SPF mouse 2

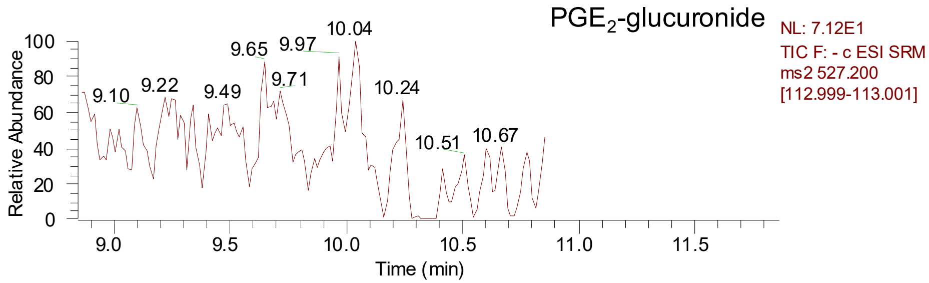

Fig. 2c

Colon-SPF mouse 3

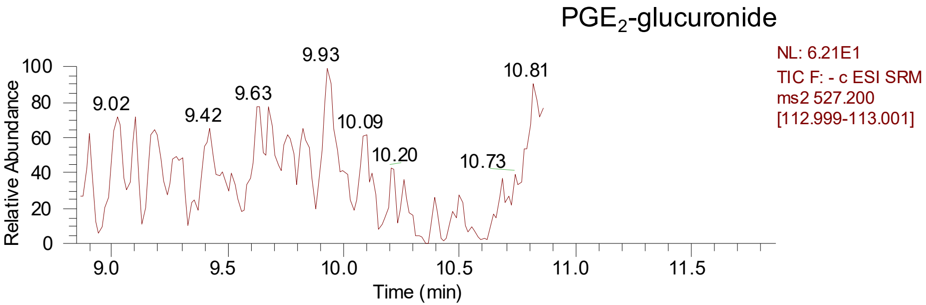

Colon-SPF mouse 4

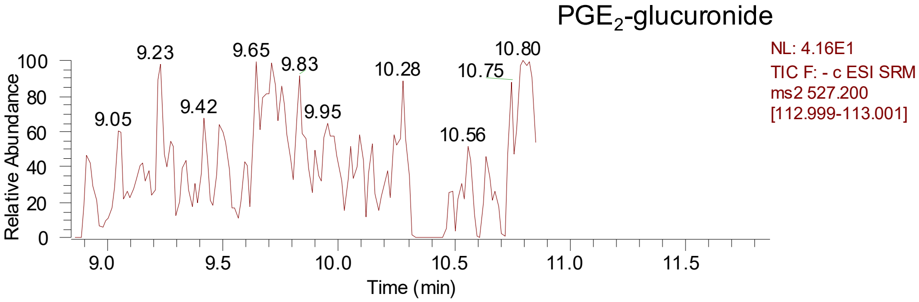

Fig. 2i
TSQ LC–MS/MS spectrometry of PGE_2-Acyl-GlcA in the gut tissues of GF and SPF mice

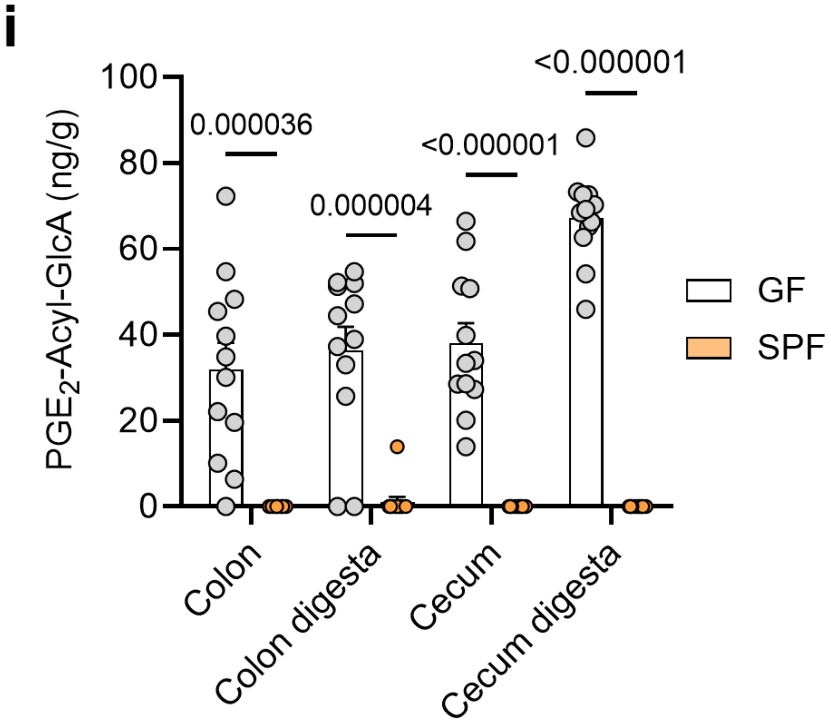

Fig. 2i

Colon-GF mouse 1

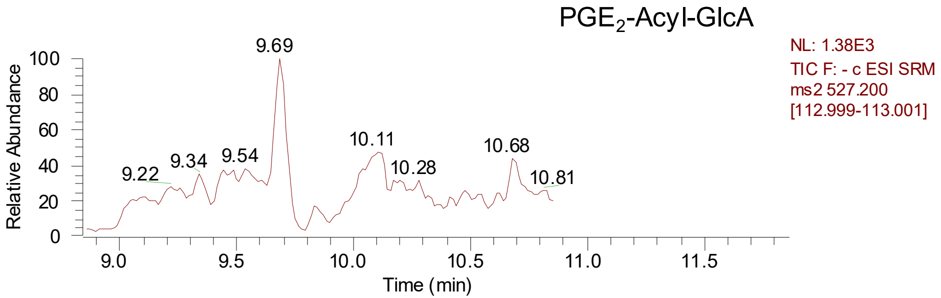

Colon-GF mouse 2

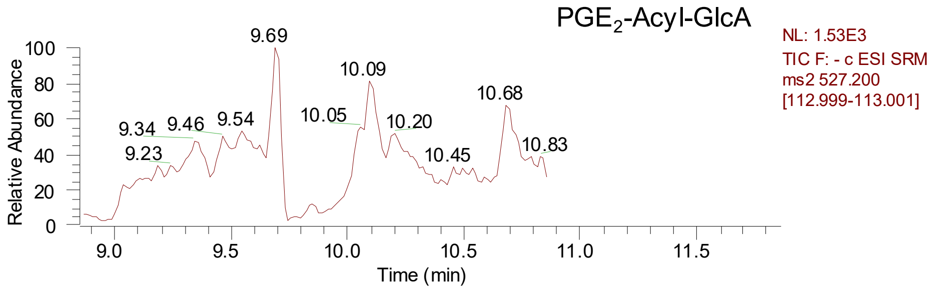

Fig. 2i

Colon-GF mouse 3

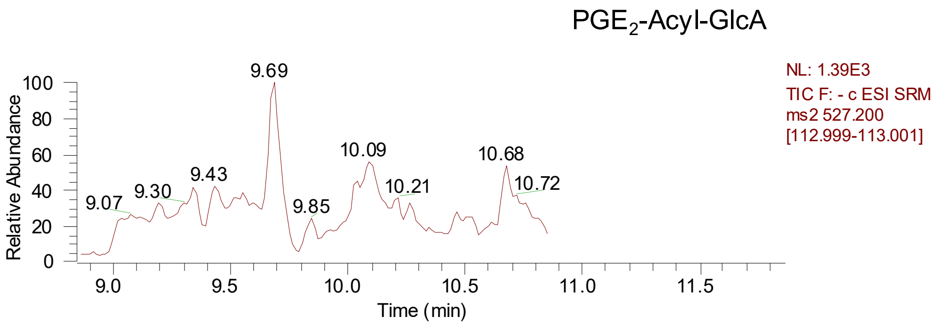

Colon-GF mouse 4

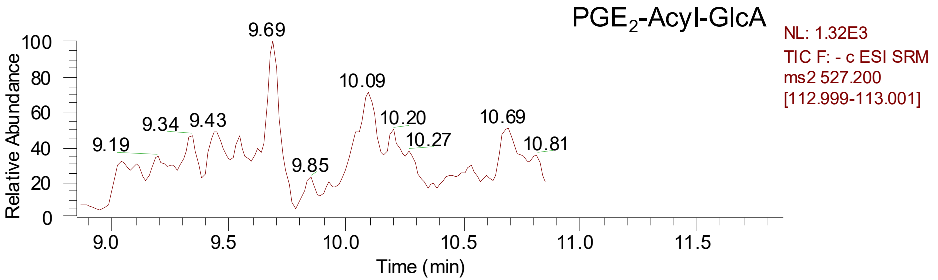

Fig. 2i

Colon-SPF mouse1

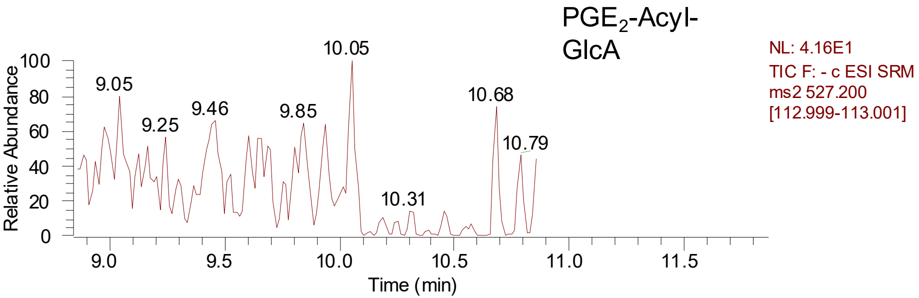

Colon-SPF mouse 2

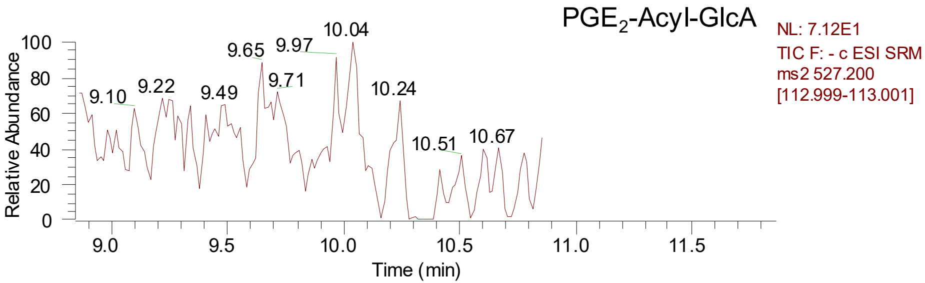

Fig. 2i

Colon-SPF mouse 3

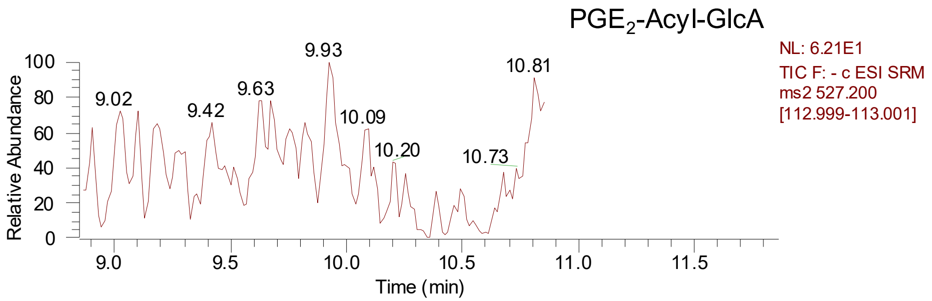

Colon-SPF mouse 4

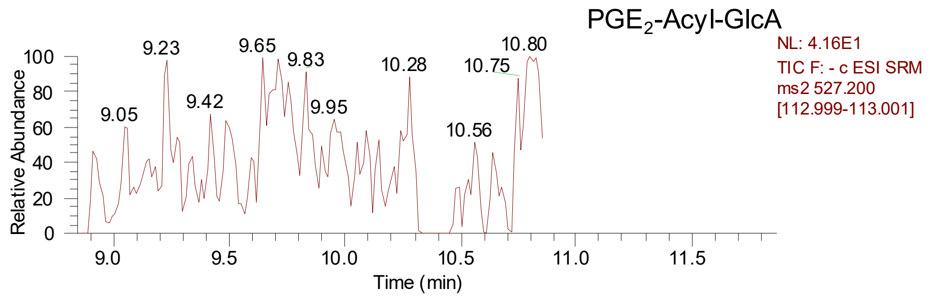

Fig. 2i

Colon digesta-GF mouse 1

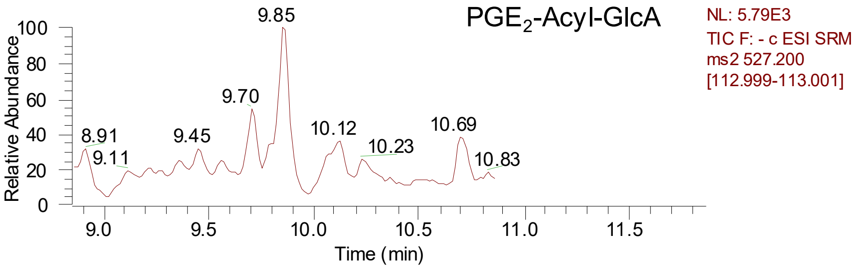

Colon digesta-GF mouse 2

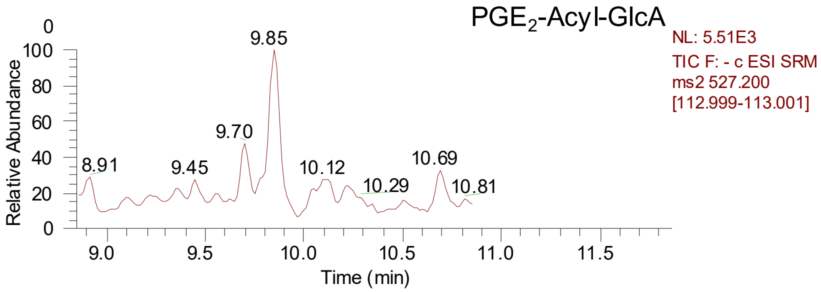

Fig. 2i

Colon digesta-GF mouse 3

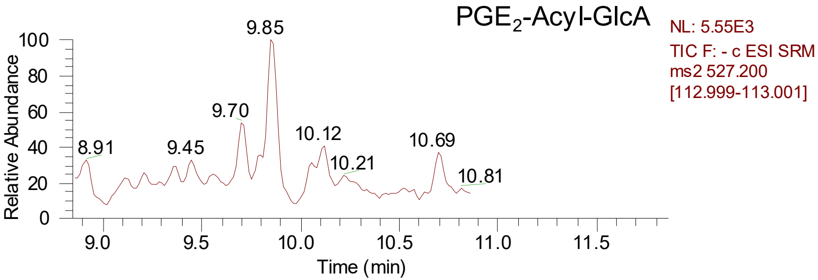

Colon digesta-GF mouse 4

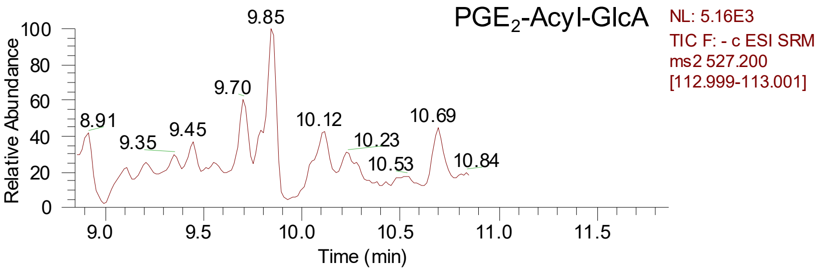

Fig. 2i

Colon digesta-SPF mouse 1

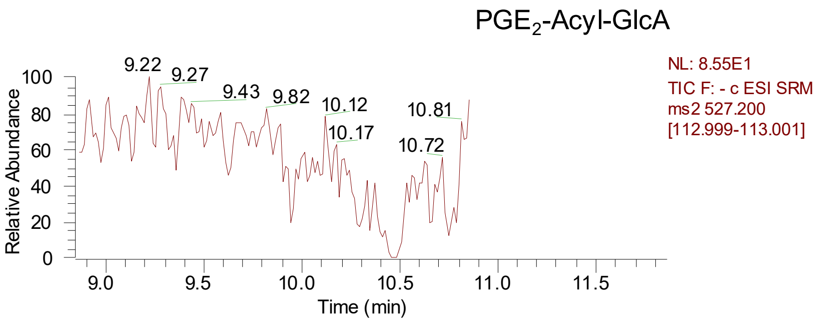

Colon digesta-SPF mouse 2

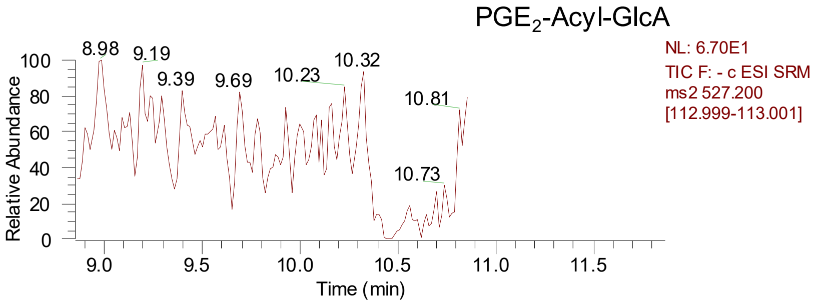

Fig. 2i

Colon digesta-SPF mouse 3

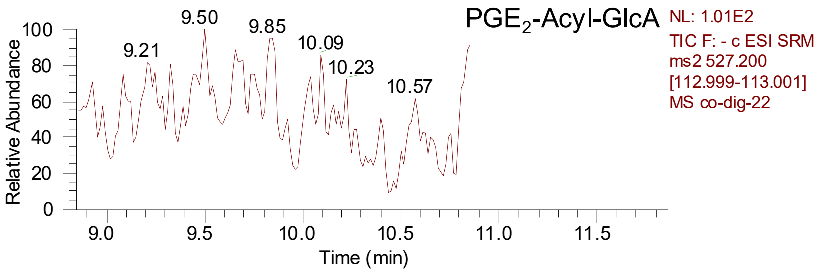

Colon digesta-SPF mouse 4

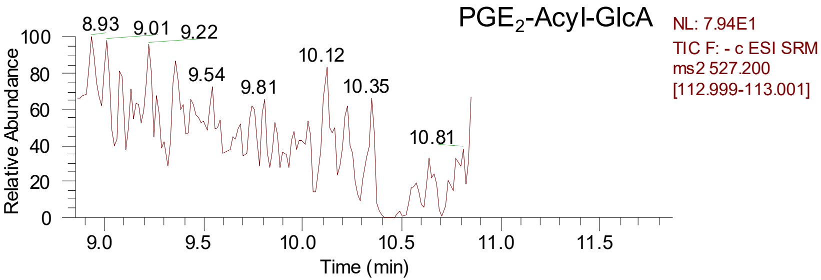

Fig. 2i

Cecum-GF mouse 1

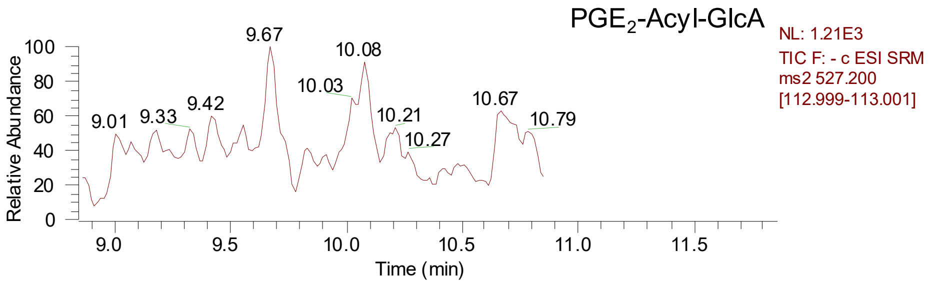

Cecum-GF mouse 2

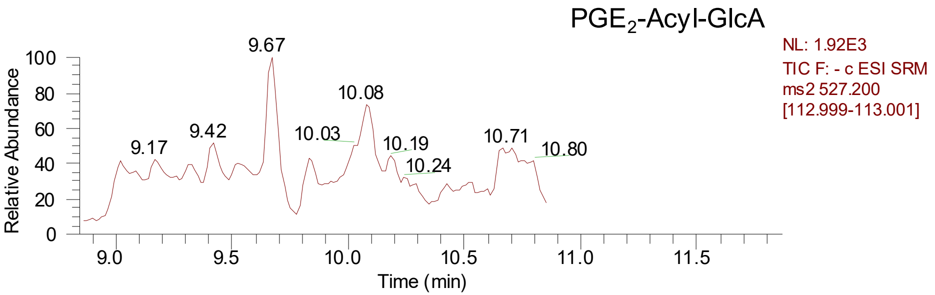

Fig. 2i

Cecum-GF mouse 3

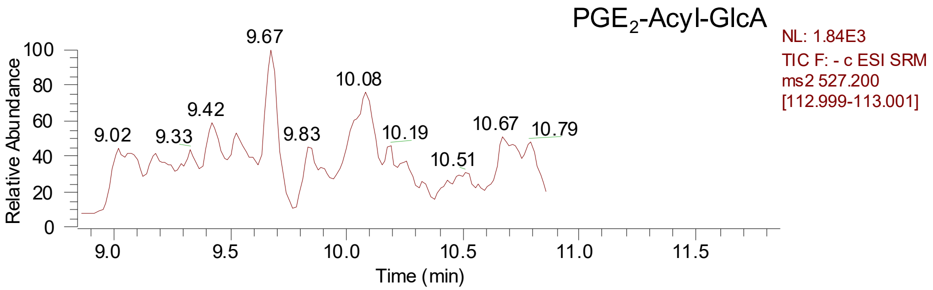

Cecum-GF mouse 4

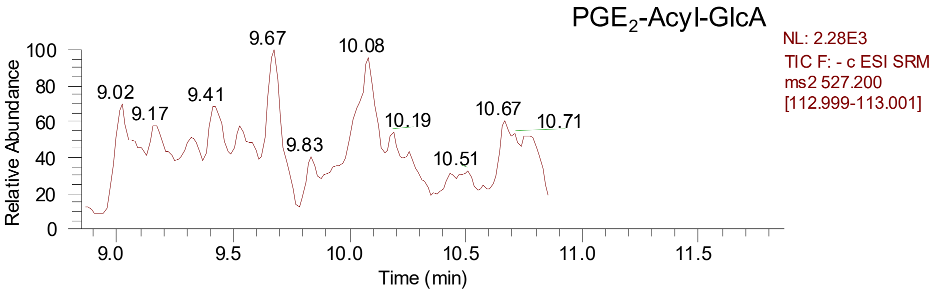

Fig. 2i

Cecum-SPF mouse 1

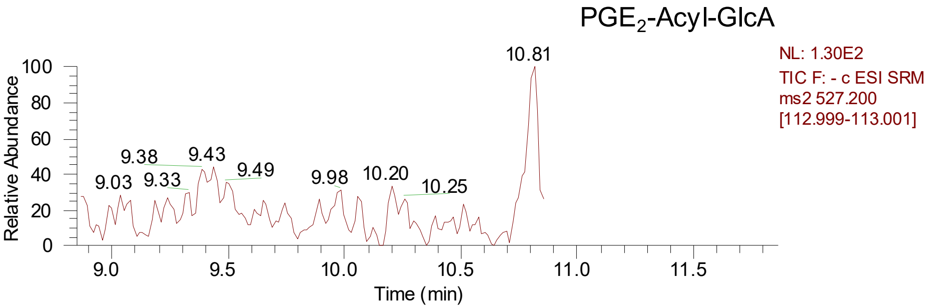

Cecum-SPF mouse 2

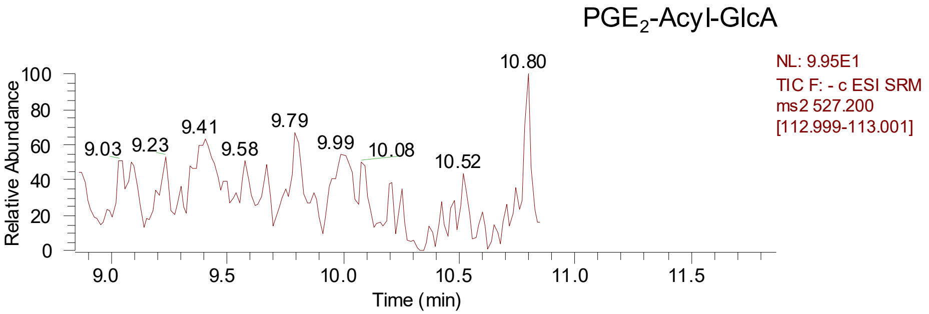

Fig. 2i

Cecum-SPF mouse 3

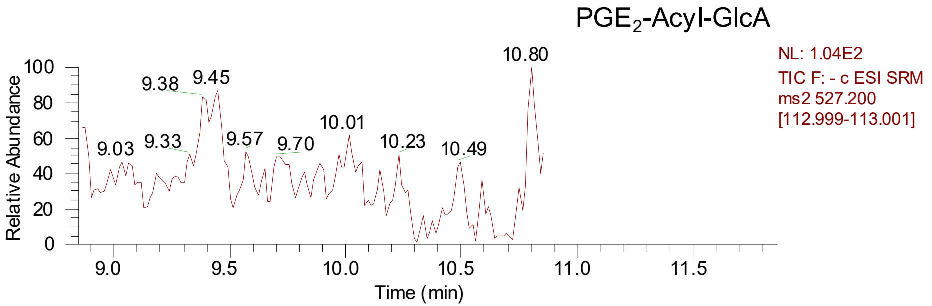

Cecum-SPF mouse 4

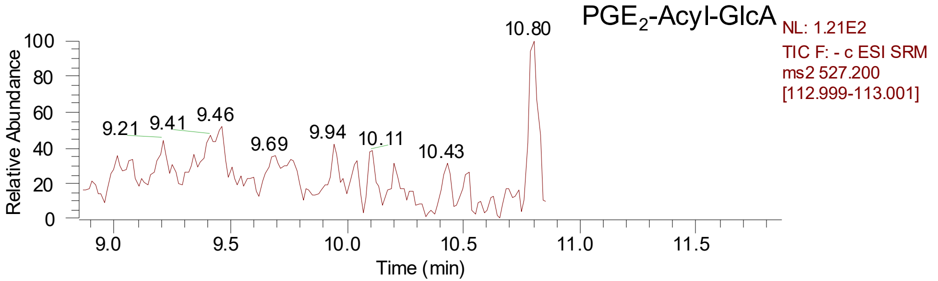

Fig. 2i

Cecum digesta-GF mouse 1

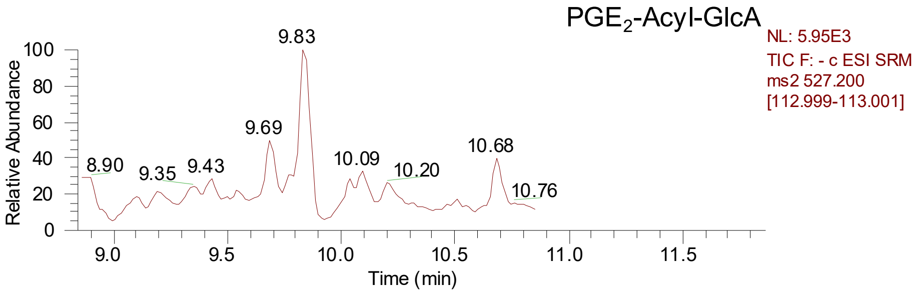

Cecum digesta-GF mouse 2

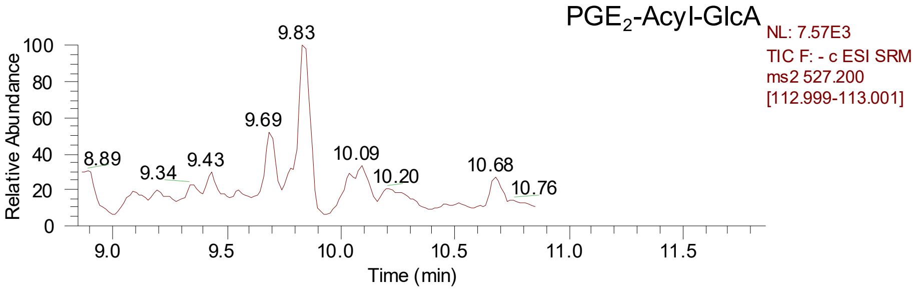

Fig. 2i

Cecum digesta-GF mouse 3

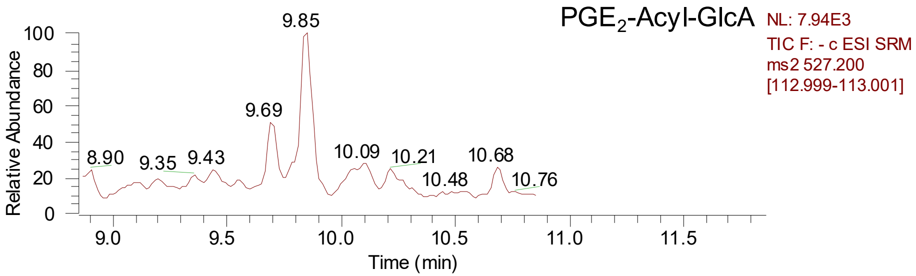

Cecum digesta-GF mouse 4

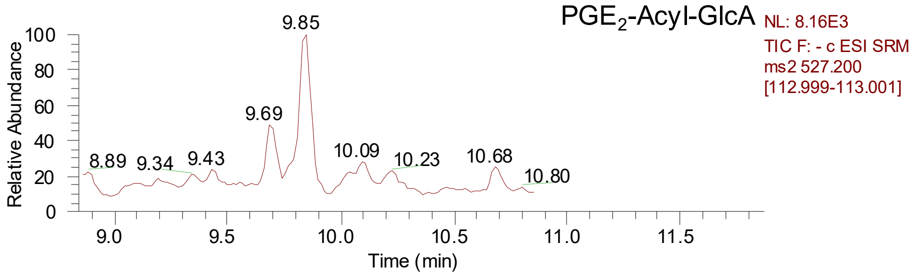

Fig. 2i

Cecum digesta-SPF mouse 1

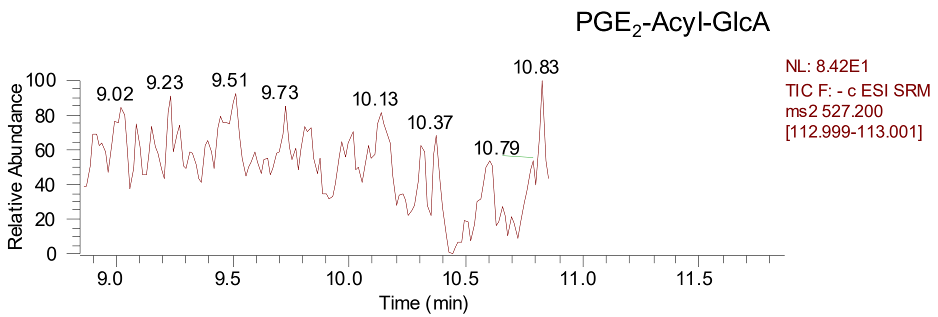

Cecum digesta-SPF mouse 2

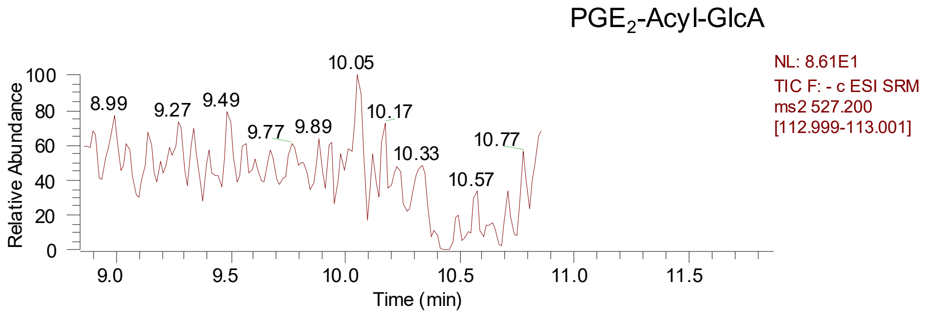

Fig. 2i

Cecum digesta-SPF mouse 3

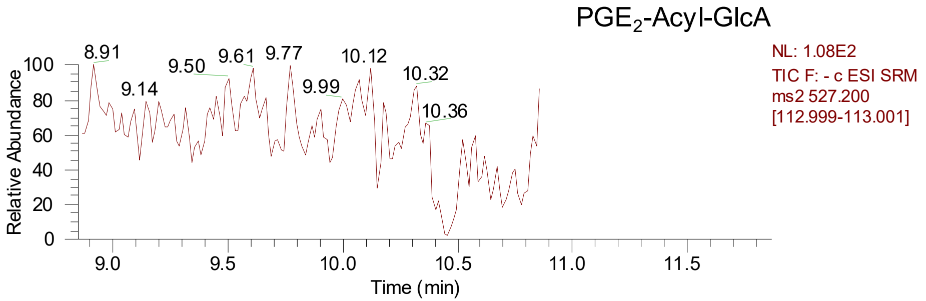

Cecum digesta-SPF mouse 4

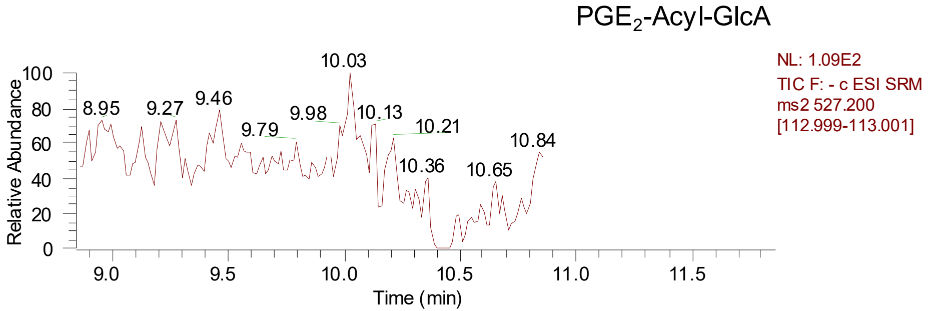

Fig. 2j
TSQ LC–MS/MS spectrometry of PGE_2-Acyl-GlcA in intestinal microsome reaction

g

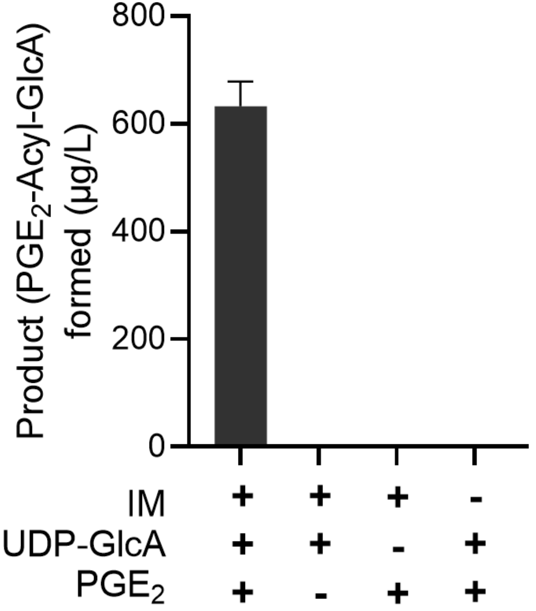

Fig. 2j

IM + UDP-GlcA + PGE$_2$
Duplicate 1

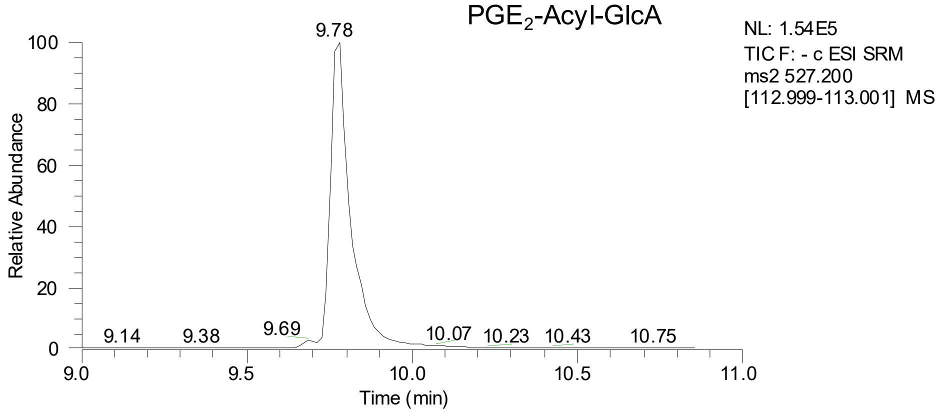

IM + UDP-GlcA + PGE$_2$
Duplicate 2

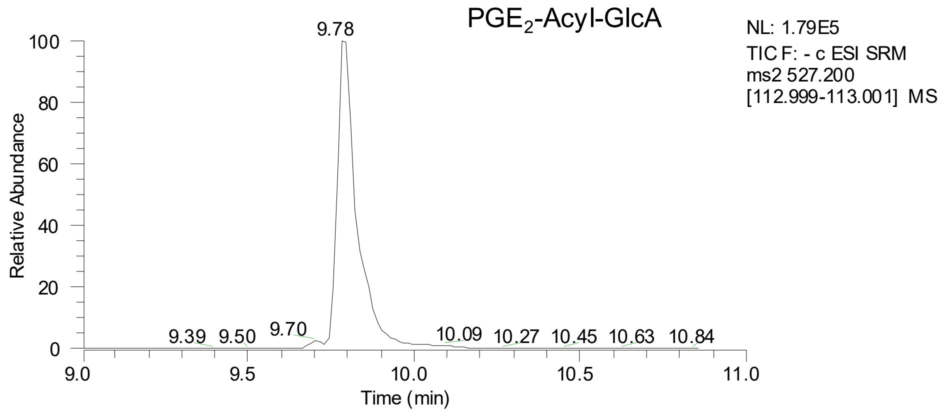

Fig. 2j

Ctrl (IM + UDP-GlcA)
Duplicate 1

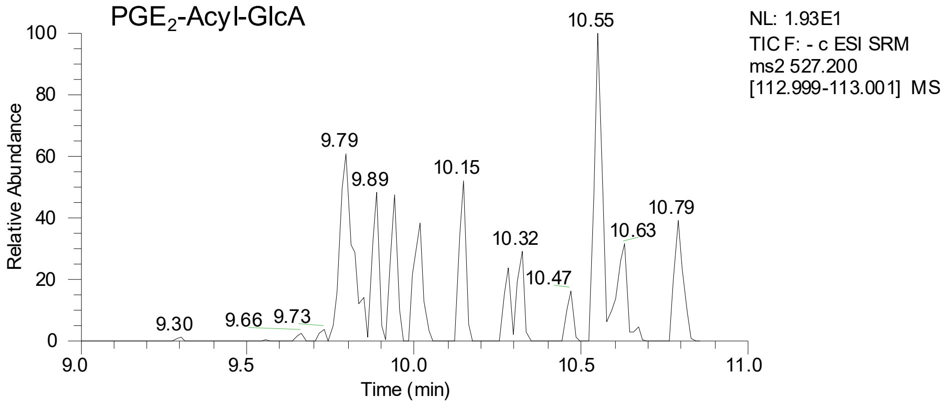

Ctrl (IM + UDP-GlcA)
Duplicate 2

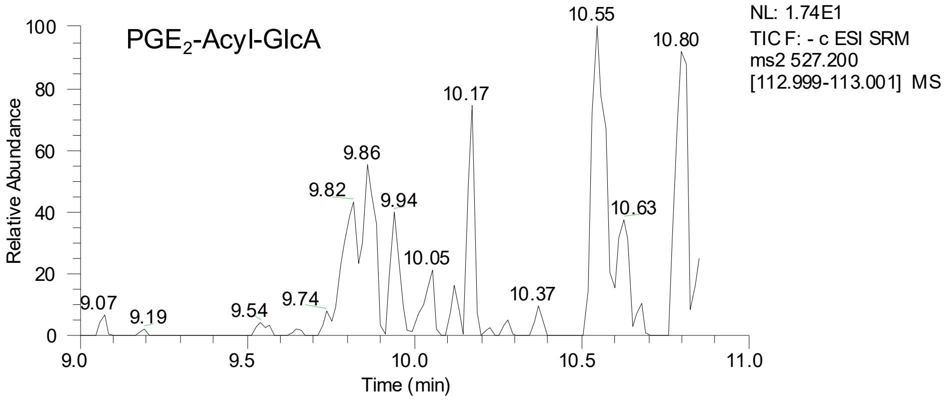

Fig. 2j

Ctrl (IM + PGE_2)
Duplicate 1

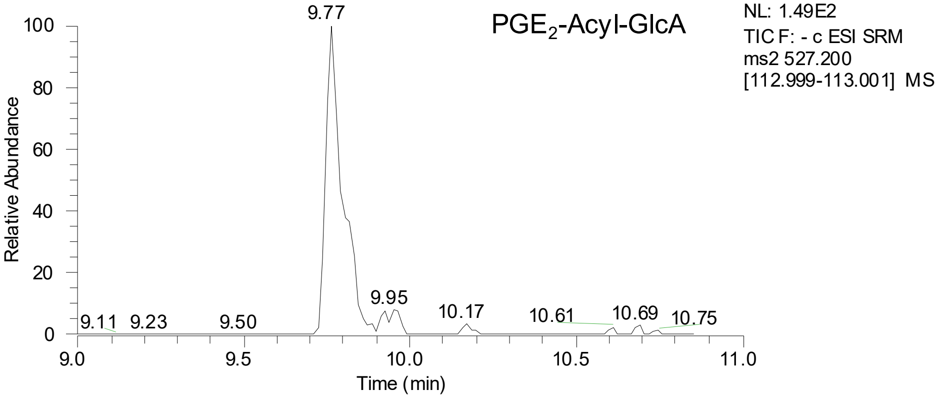

Ctrl (IM + PGE_2)
Duplicate 2

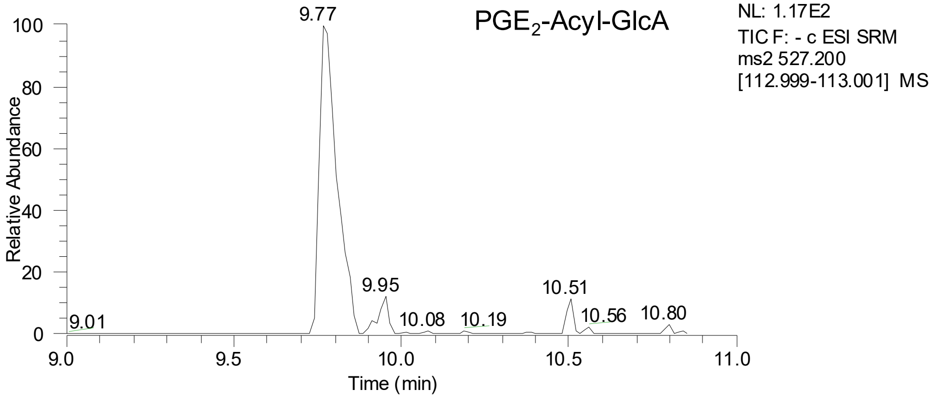

Fig. 2j

Ctrl (UDP-GlcA + PGE$_2$)
Duplicate 1

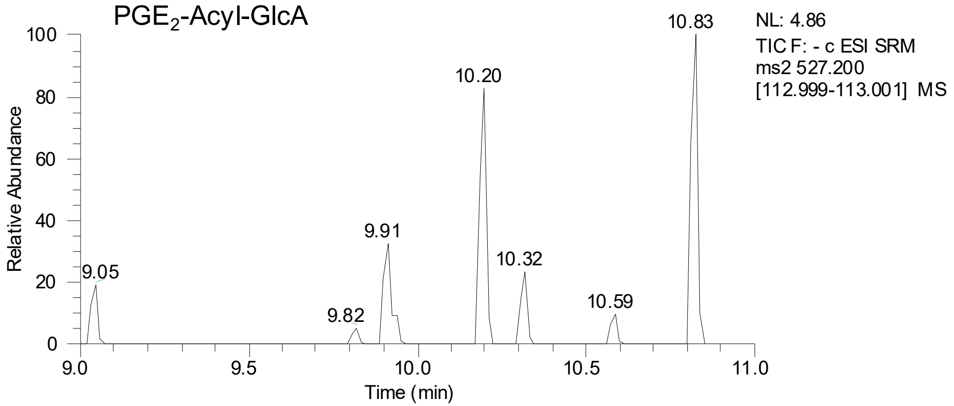

Ctrl (UDP-GlcA + PGE$_2$)
Duplicate 2

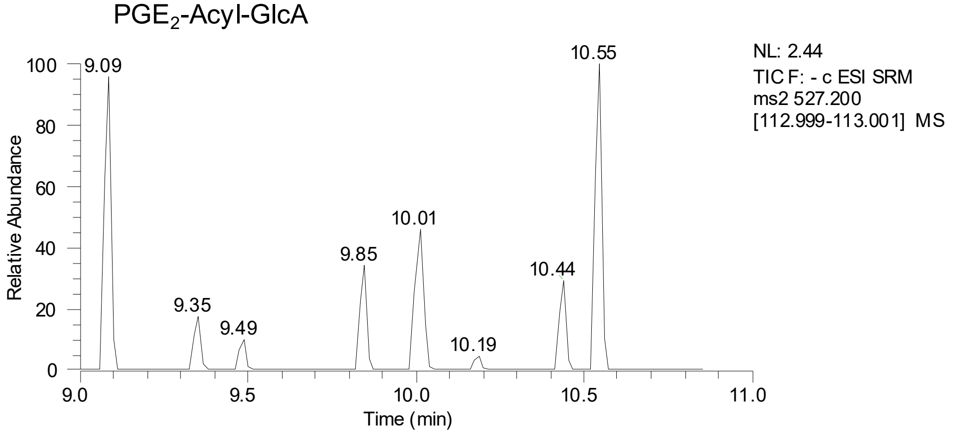

Extended Data Fig. 2
TSQ LC–MS/MS spectrometry of PGE_2 in mouse tissues

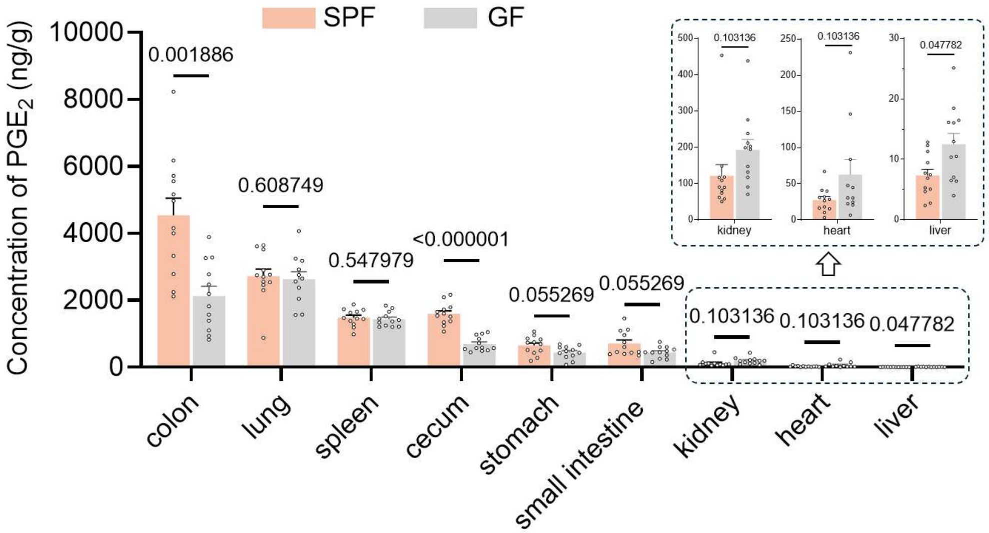

Extended Data Fig. 2

Colon-GF mouse 1

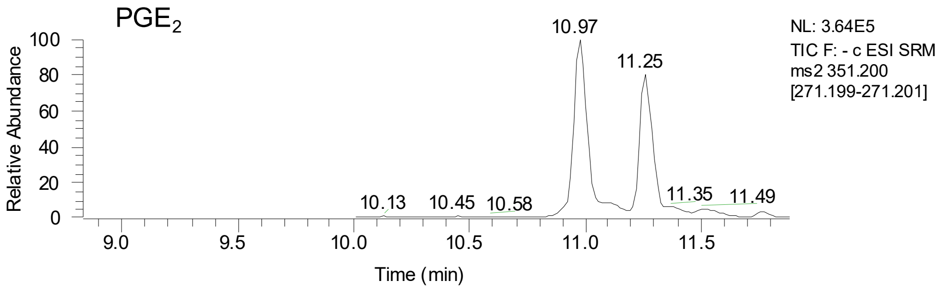

Colon-GF mouse 2

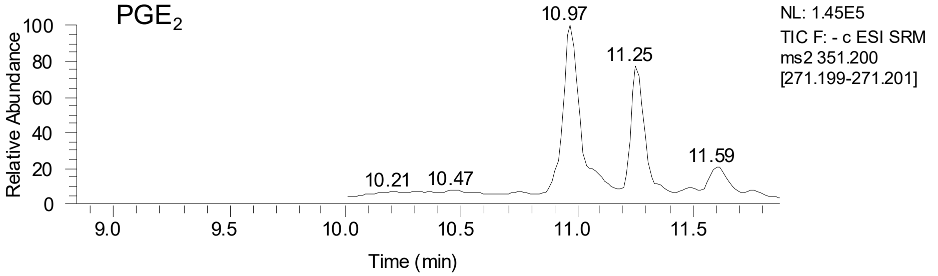

Extended Data Fig. 2

Colon-GF mouse 3

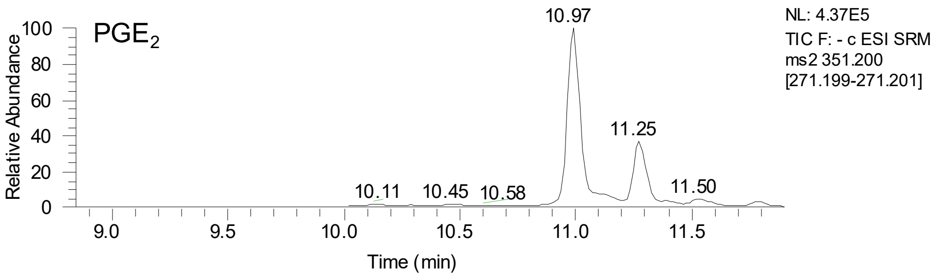

Colon-GF mouse 4

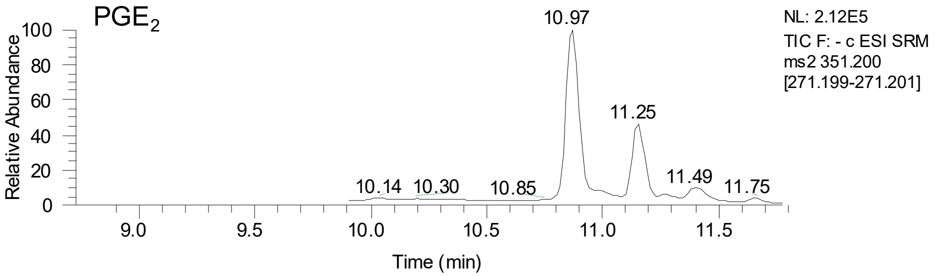

Extended Data Fig. 2

Colon-SPF mouse1

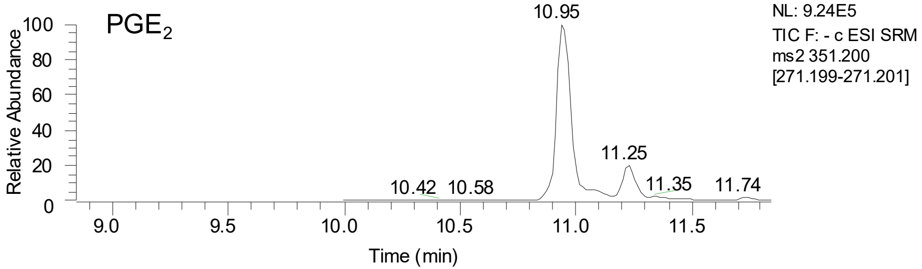

Colon-SPF mouse 2

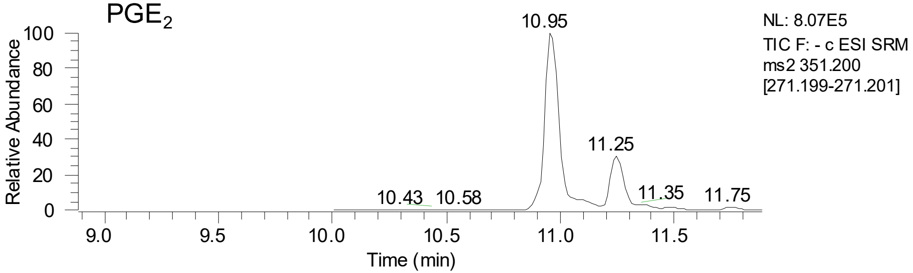

Extended Data Fig. 2

Colon-SPF mouse 3

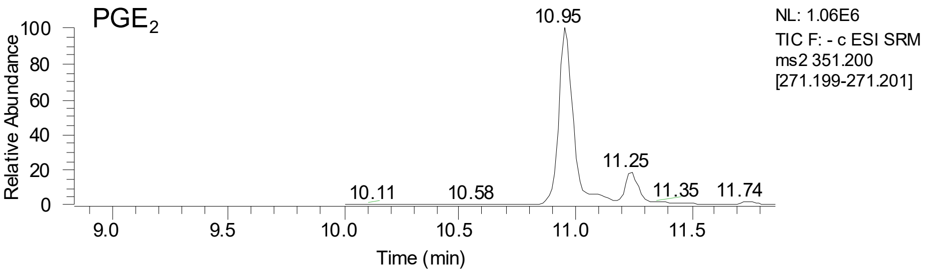

Colon-SPF mouse 4

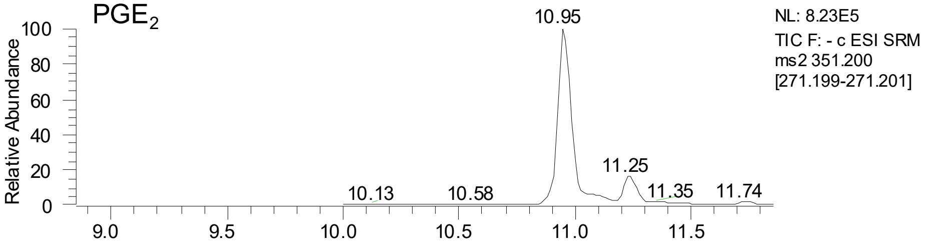

Extended Data Fig. 2

Lung-GF mouse 1

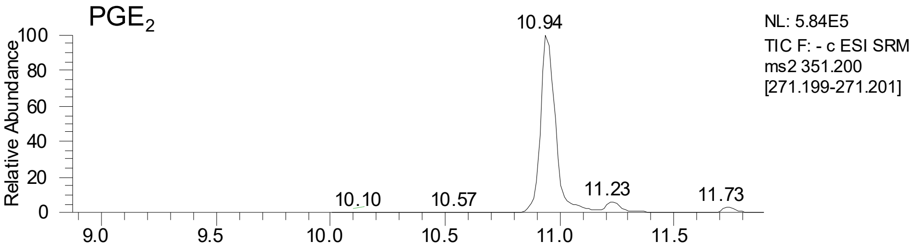

Lung-GF mouse 2

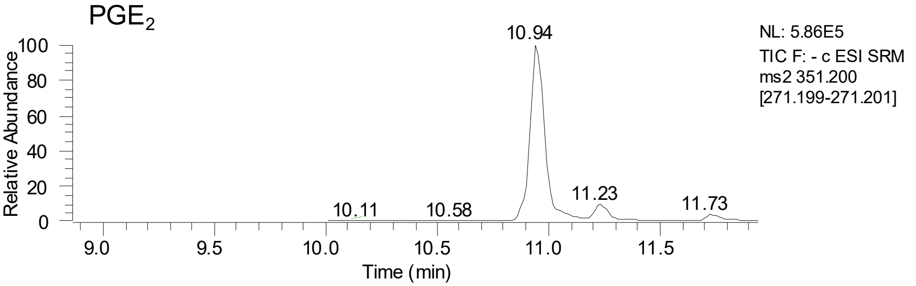

Extended Data Fig. 2

Lung-GF mouse 3

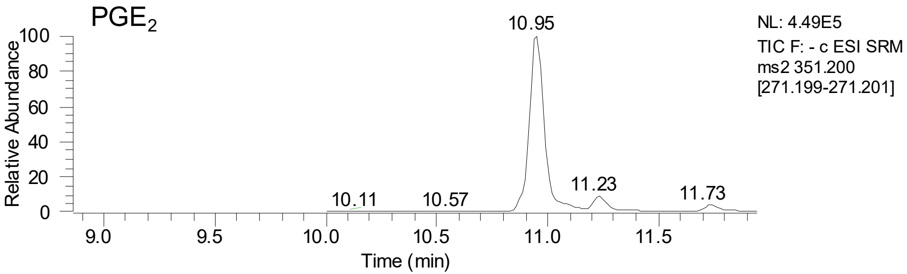

Lung-GF mouse 4

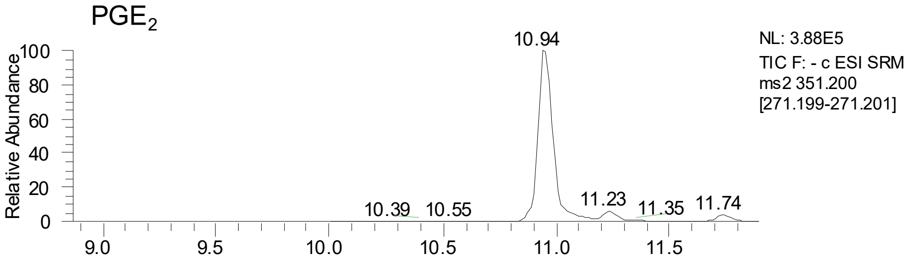

Extended Data Fig. 2

Lung-SPF mouse 1

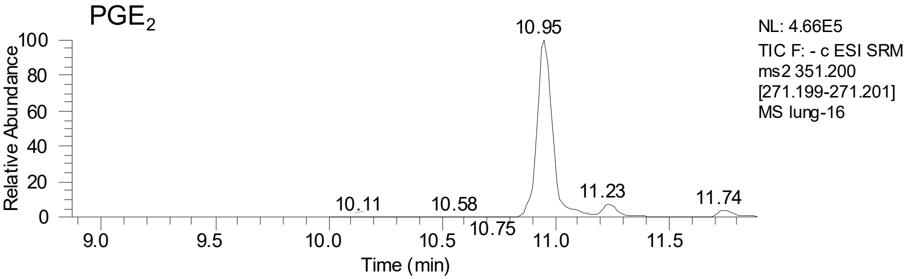

Lung-SPF mouse 2

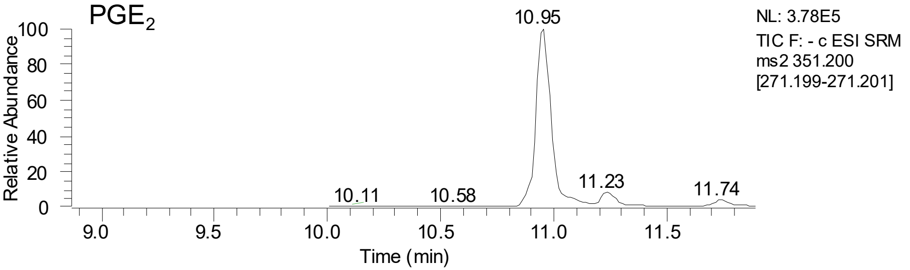

Extended Data Fig. 2

Lung-SPF mouse 3

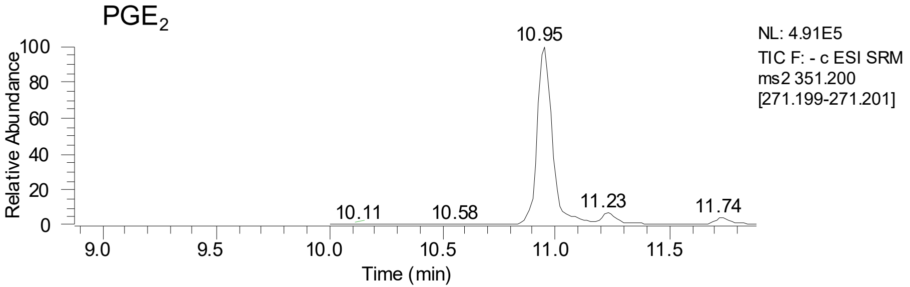

Lung-SPF mouse 4

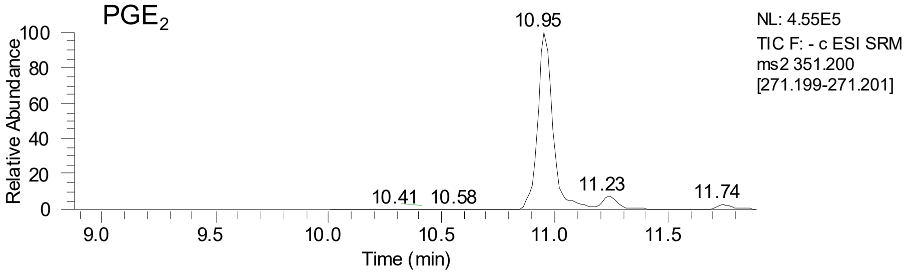

Extended Data Fig. 2

Spleen-GF mouse 1

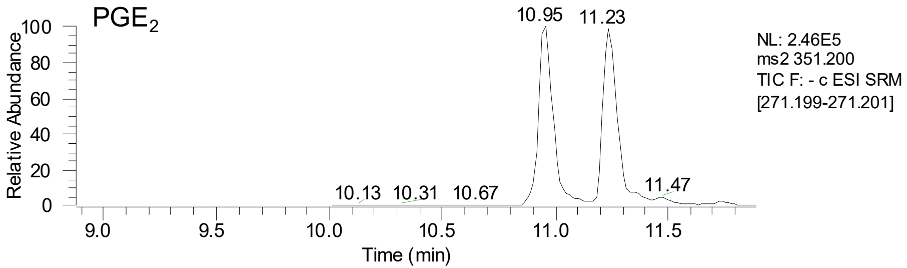

Spleen-GF mouse 2

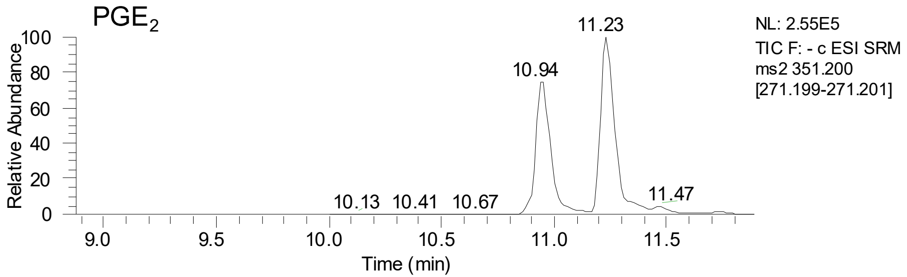

Extended Data Fig. 2

Spleen-GF mouse 3

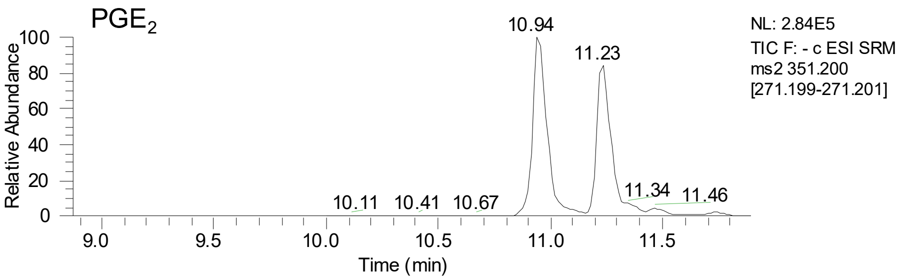

Spleen-GF mouse 4

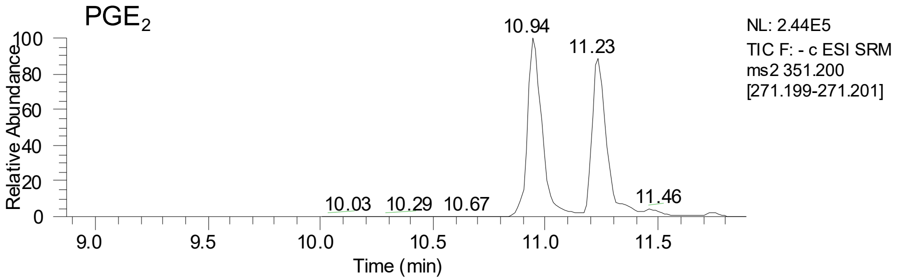

Extended Data Fig. 2

Spleen-SPF mouse 1

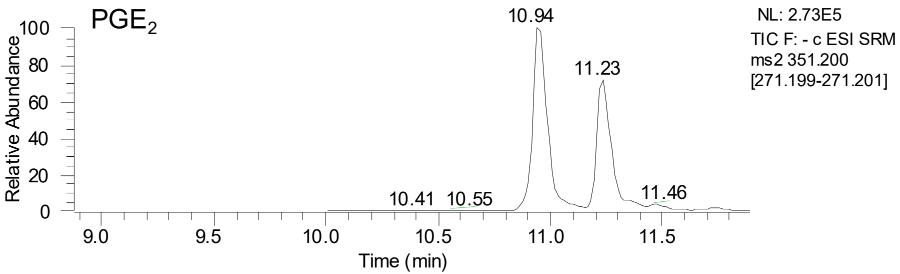

Spleen-SPF mouse 2

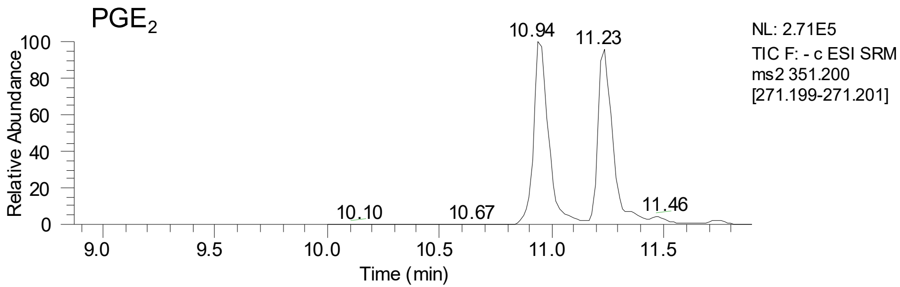

Extended Data Fig. 2

Spleen-SPF mouse 3

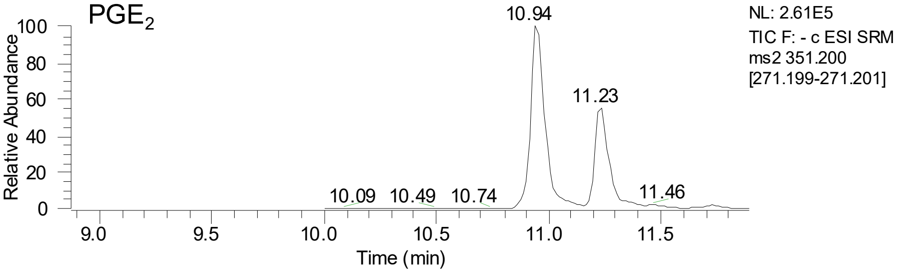

Spleen-SPF mouse 4

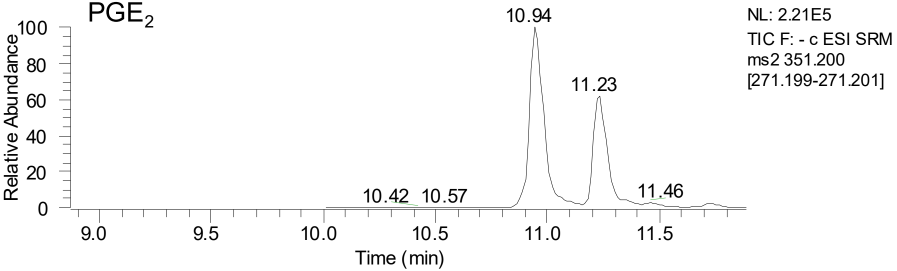

Extended Data Fig. 2

Cecum-GF mouse 1

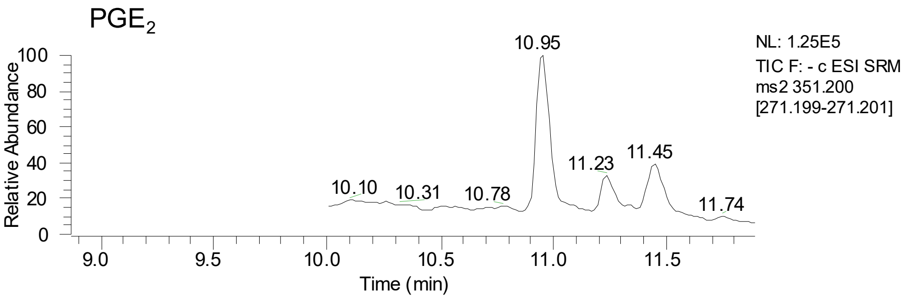

Cecum-GF mouse 2

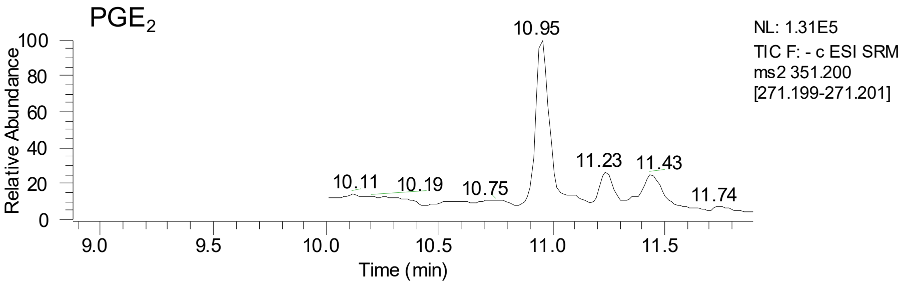

Extended Data Fig. 2

Cecum-GF mouse 3

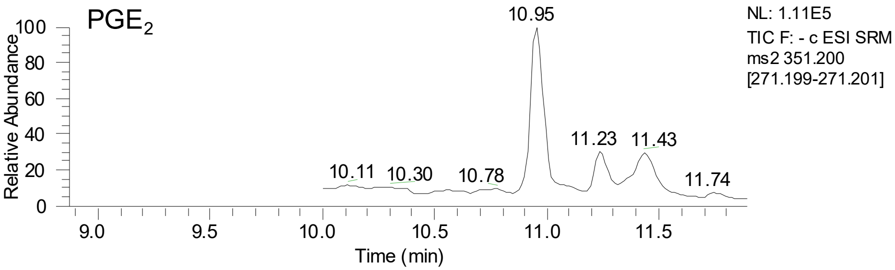

Cecum-GF mouse 4

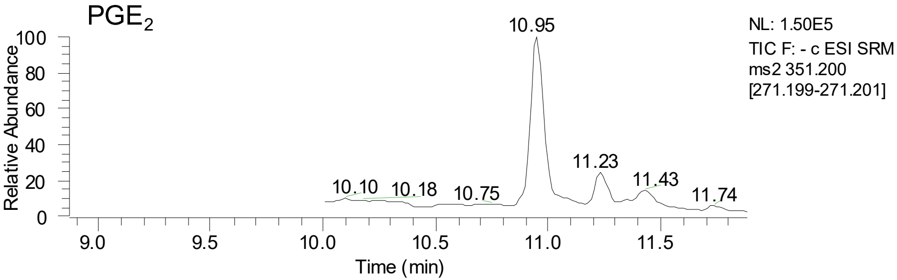

Extended Data Fig. 2

Cecum-SPF mouse 1

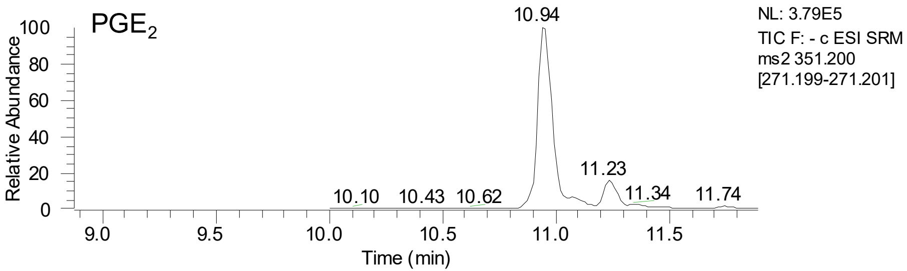

Cecum-SPF mouse 2

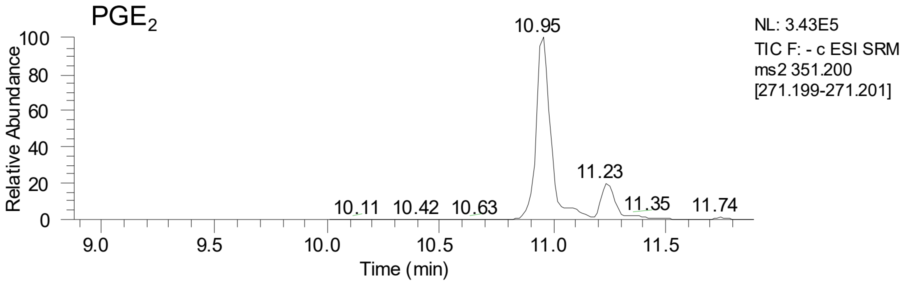

Extended Data Fig. 2

Cecum-SPF mouse 3

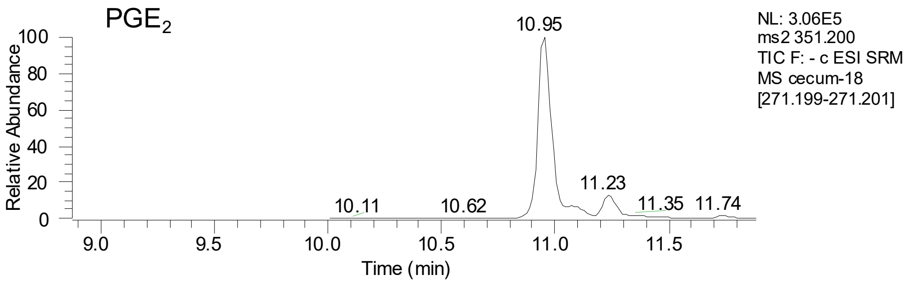

Cecum-SPF mouse 4

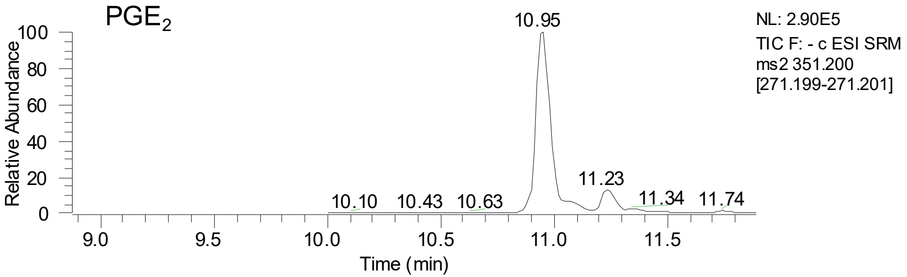

Extended Data Fig. 2

Stomach-GF mouse 1

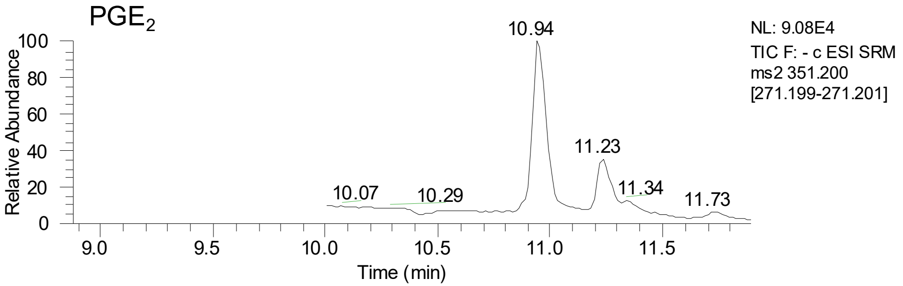

Stomach-GF mouse 2

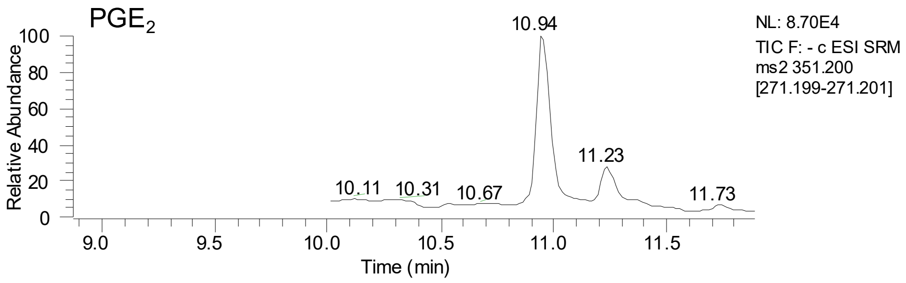

Extended Data Fig. 2

Stomach-GF mouse 3

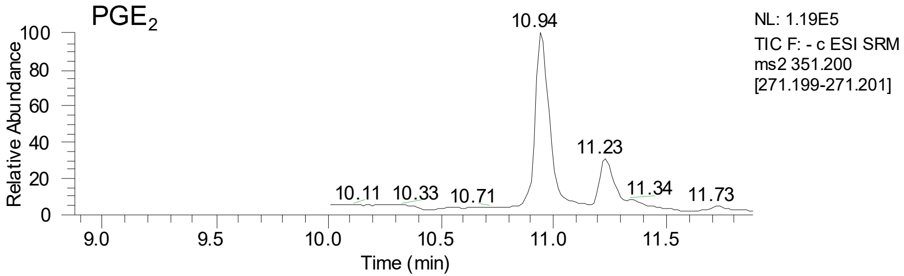

Stomach-GF mouse 4

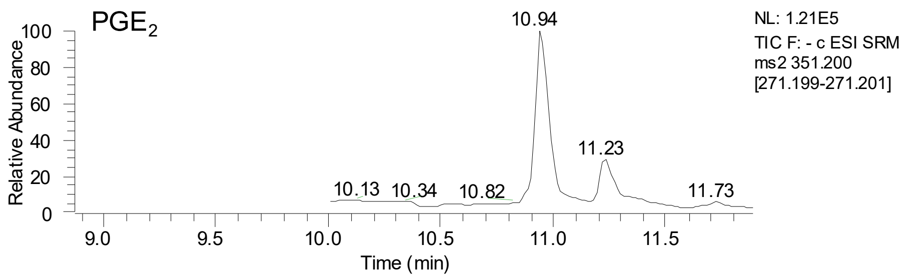

Extended Data Fig. 2

Stomach-SPF mouse 1

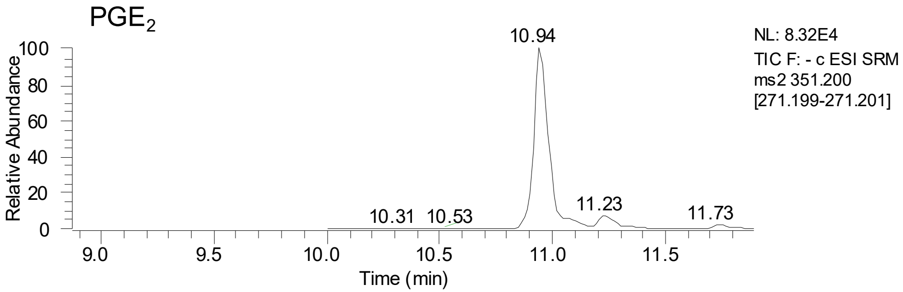

Stomach-SPF mouse 2

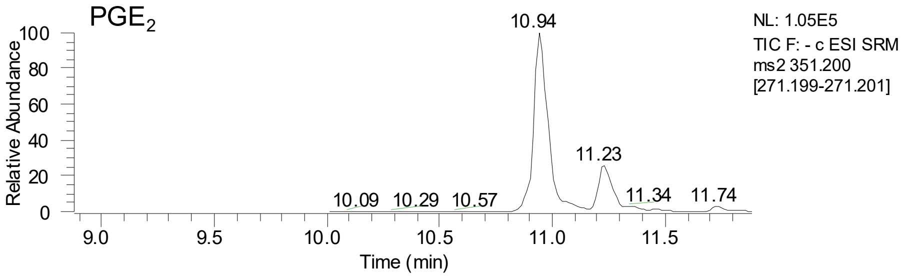

Extended Data Fig. 2

Stomach-SPF mouse 3

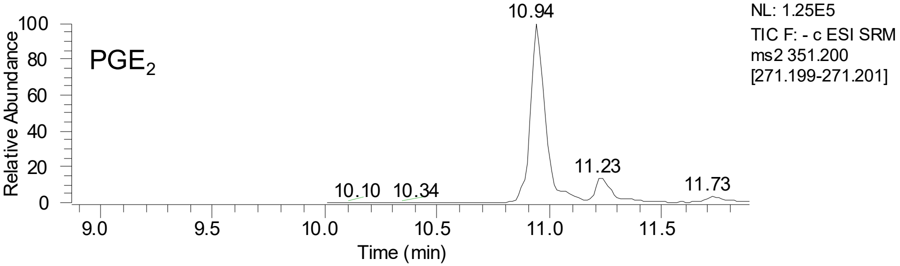

Stomach-SPF mouse 4

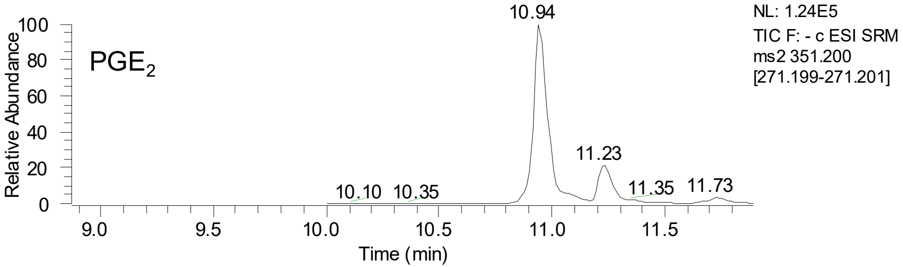

Extended Data Fig. 2

Small intestine-GF mouse 1

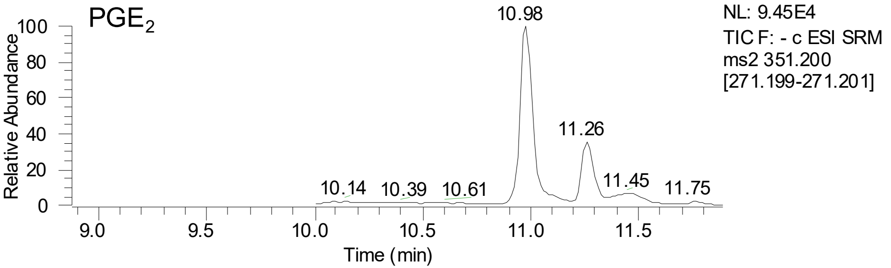

Small intestine-GF mouse 2

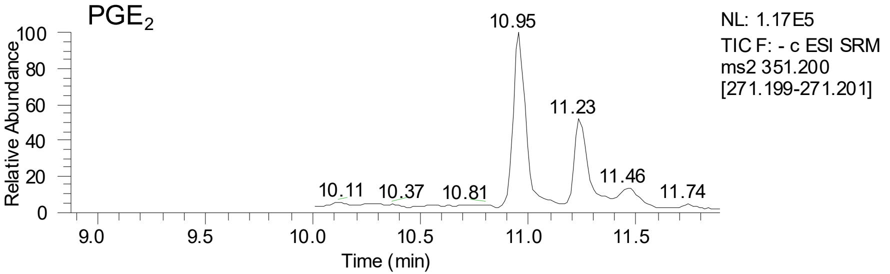

Extended Data Fig. 2

Small intestine-GF mouse 3

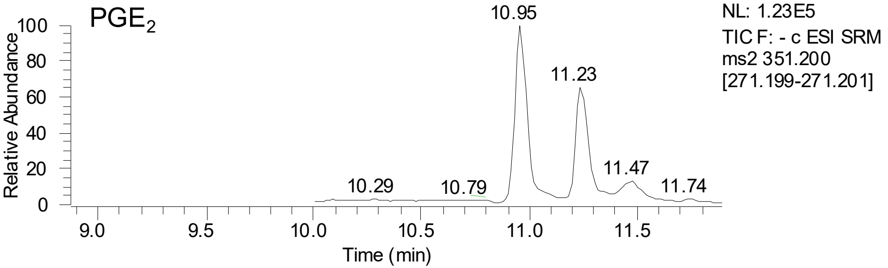

Small intestine-GF mouse 4

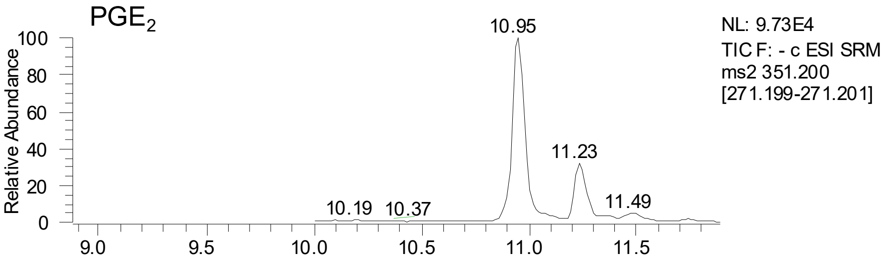

Extended Data Fig. 2

Small intestine-SPF mouse 1

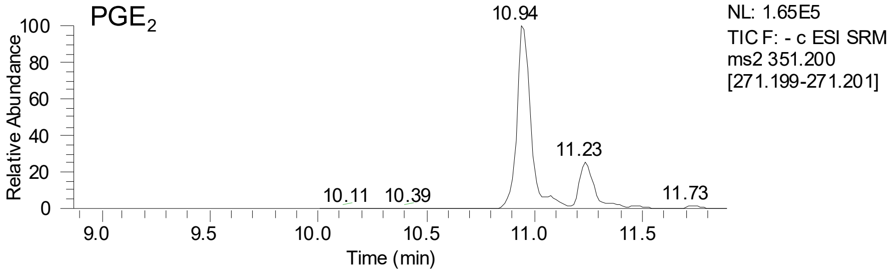

Small intestine-SPF mouse 2

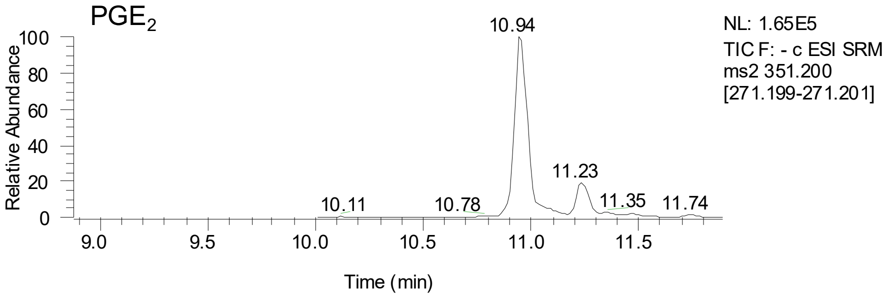

Extended Data Fig. 2

Small intestine-SPF mouse 3

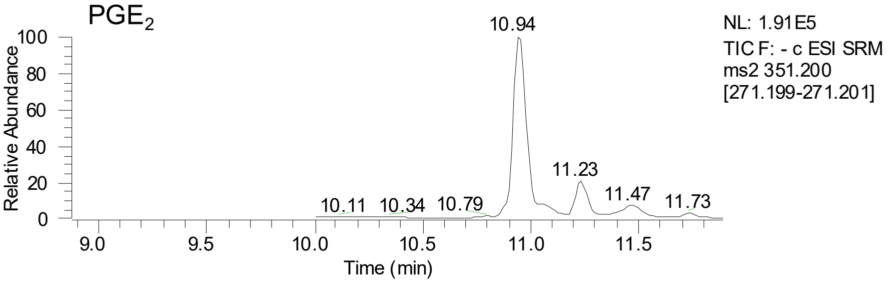

Small intestine-SPF mouse 4

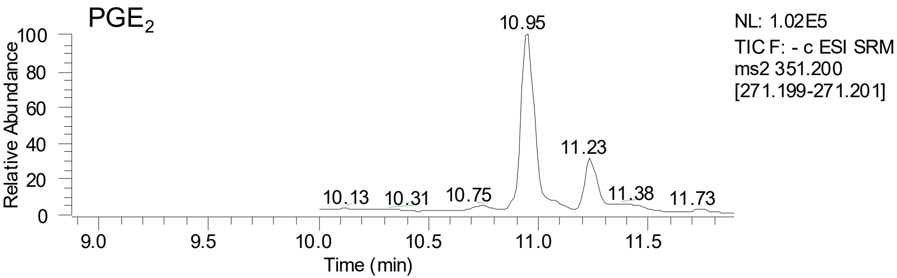

Extended Data Fig. 2

Kidney-GF mouse 1

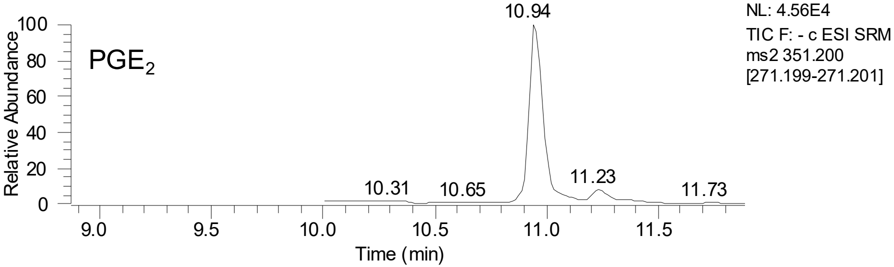

Kidney-GF mouse 2

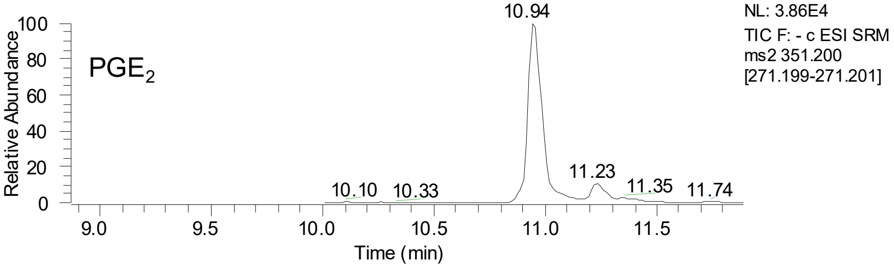

Extended Data Fig. 2

Kidney-GF mouse 3

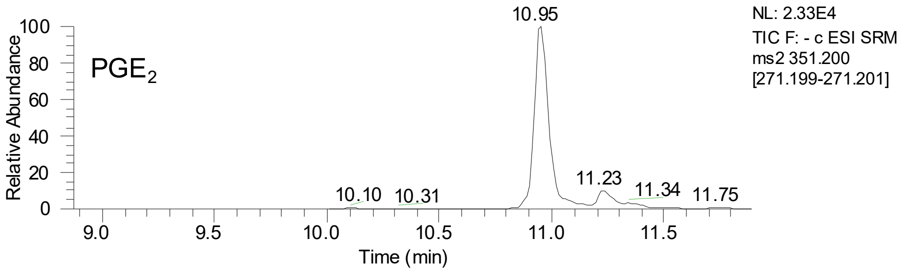

Kidney-GF mouse 4

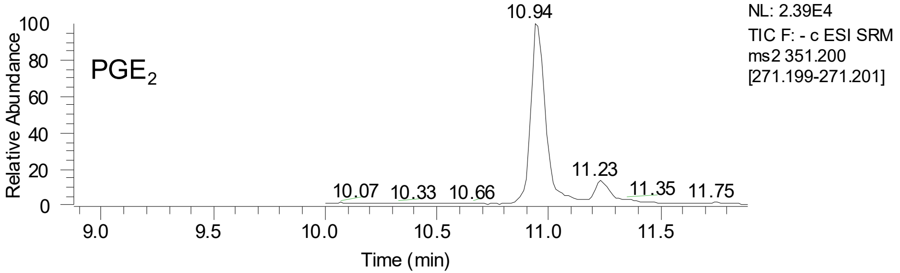

Extended Data Fig. 2

Kidney-SPF mouse 1

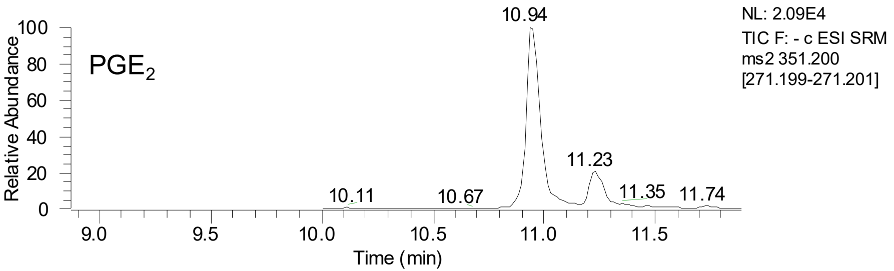

Kidney-SPF mouse 2

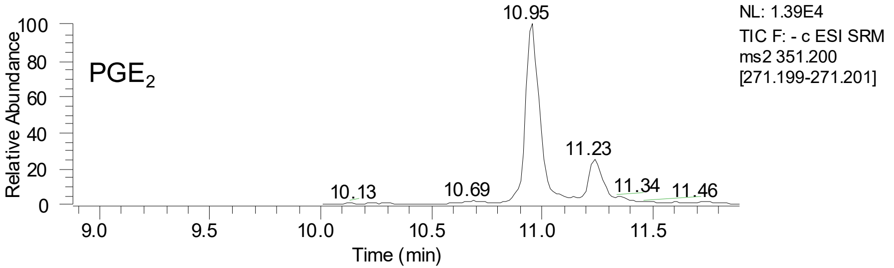

Extended Data Fig. 2

Kidney-SPF mouse 3

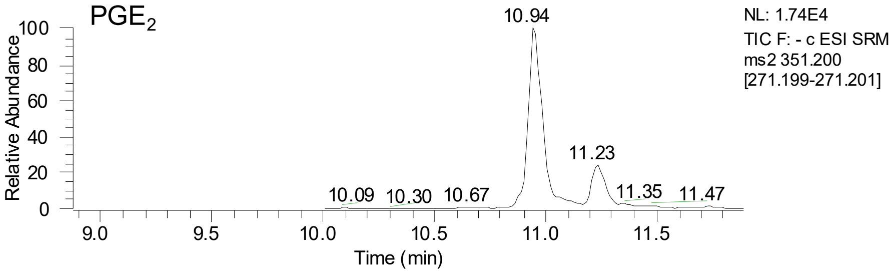

Kidney-SPF mouse 4

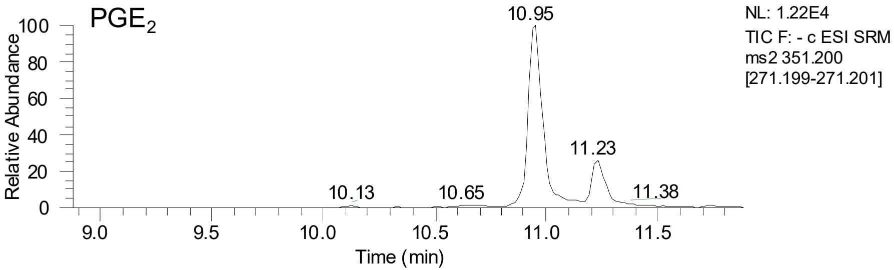

Extended Data Fig. 2

Heart-GF mouse 1

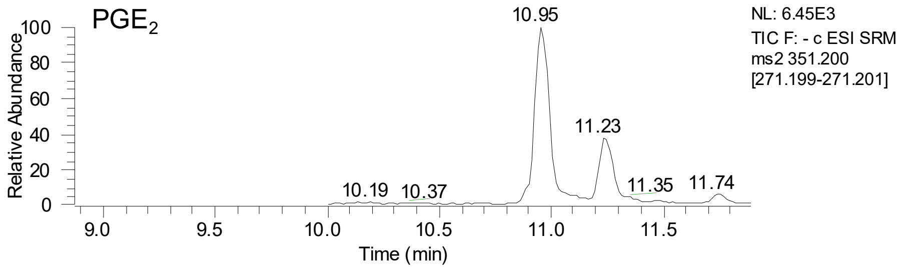

Heart-GF mouse 2

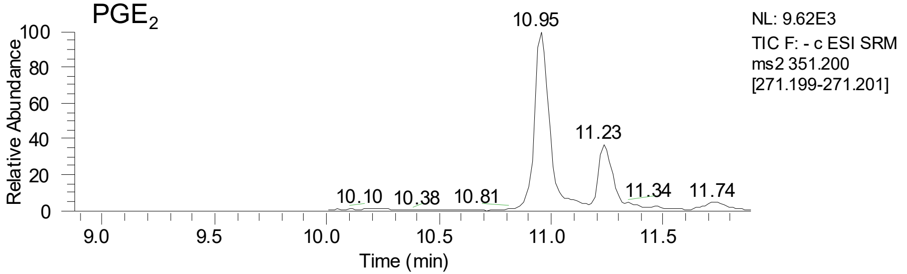

Extended Data Fig. 2

Heart-GF mouse 3

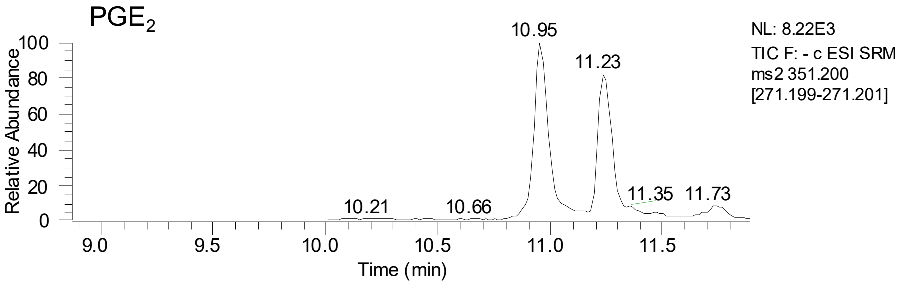

Heart-GF mouse 4

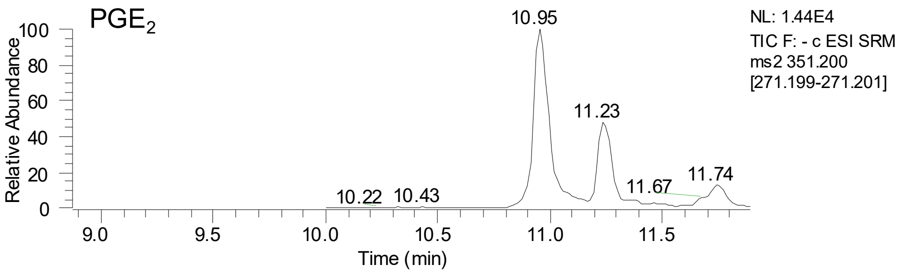

Extended Data Fig. 2

Heart-SPF mouse 1

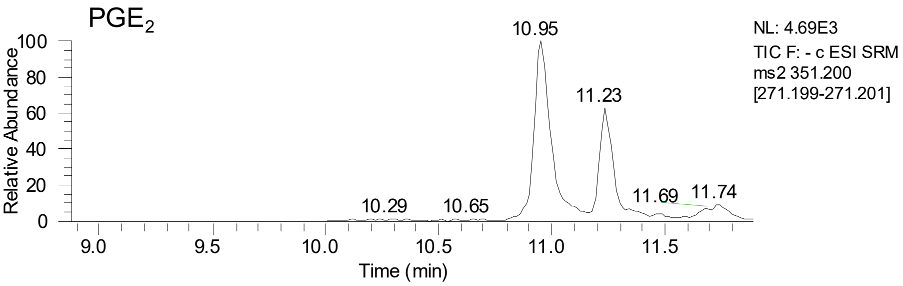

Heart-SPF mouse 2

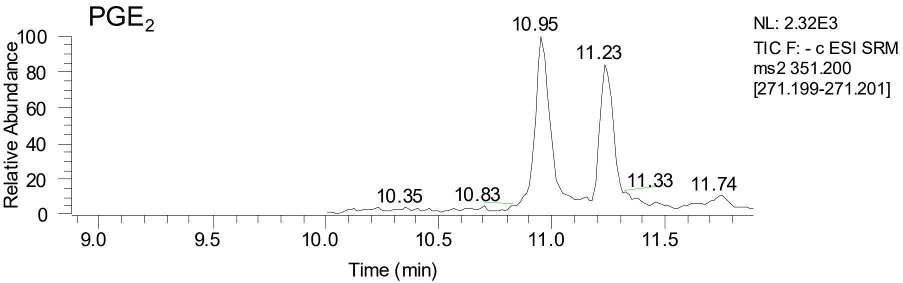

Extended Data Fig. 2

Heart-SPF mouse 3

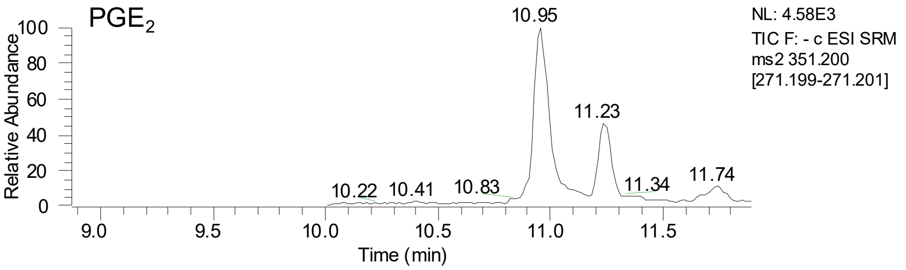

Heart-SPF mouse 4

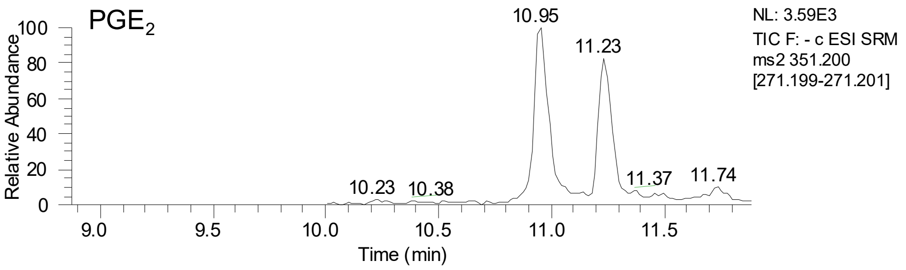

Extended Data Fig. 2

Liver-GF mouse 1

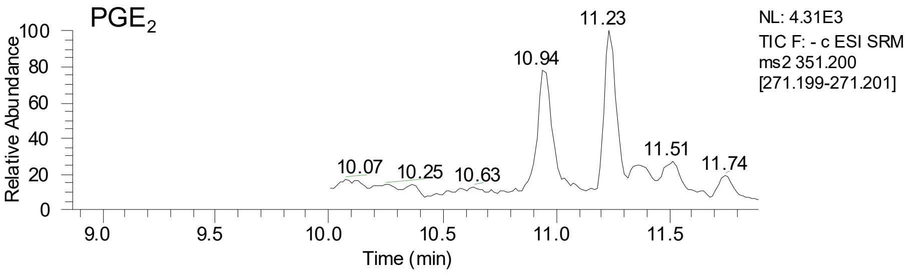

Liver-GF mouse 2

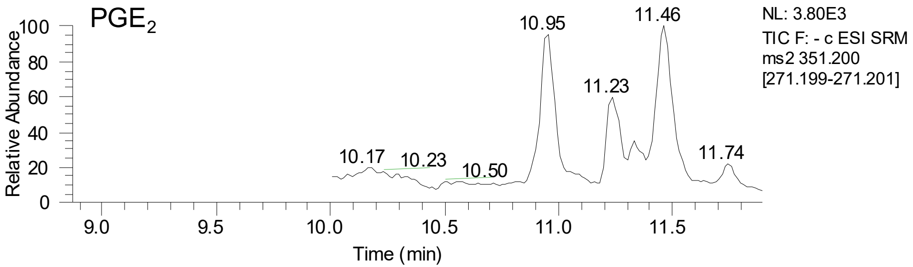

Extended Data Fig. 2

Liver-GF mouse 3

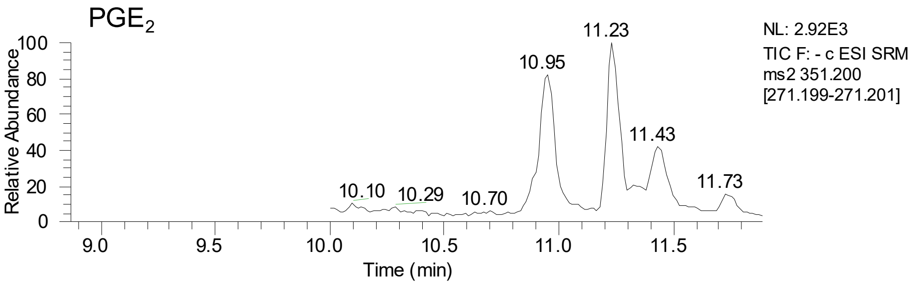

Liver-GF mouse 4

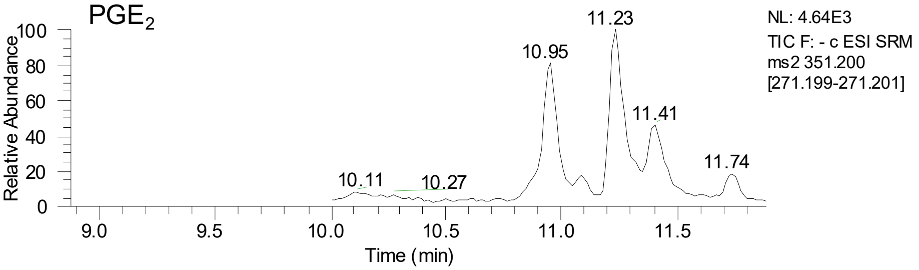

Extended Data Fig. 2

Liver-SPF mouse 1

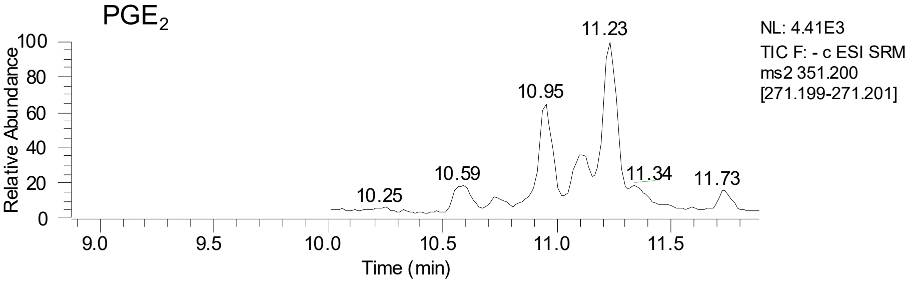

Liver-SPF mouse 2

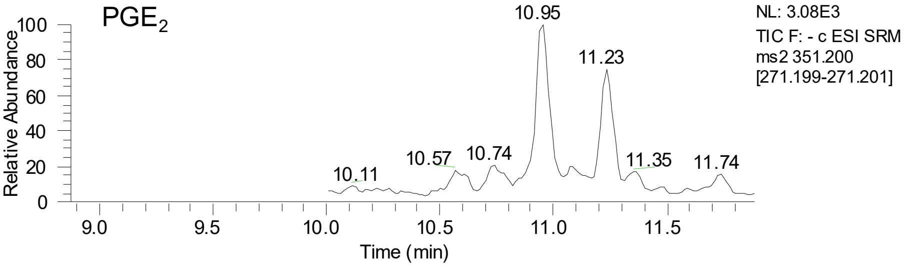

Extended Data Fig. 2

Liver-SPF mouse 3

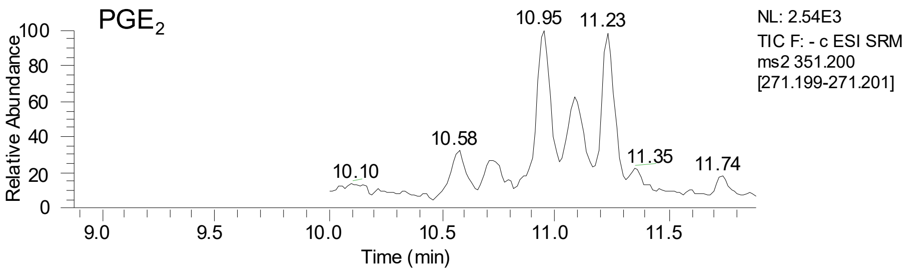

Liver-SPF mouse 4

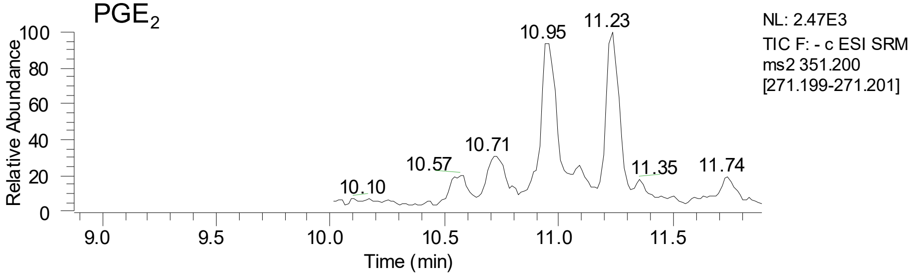

Extended Data Fig. 4
LC-HRMS spectrometry of PGE_2-sulfate

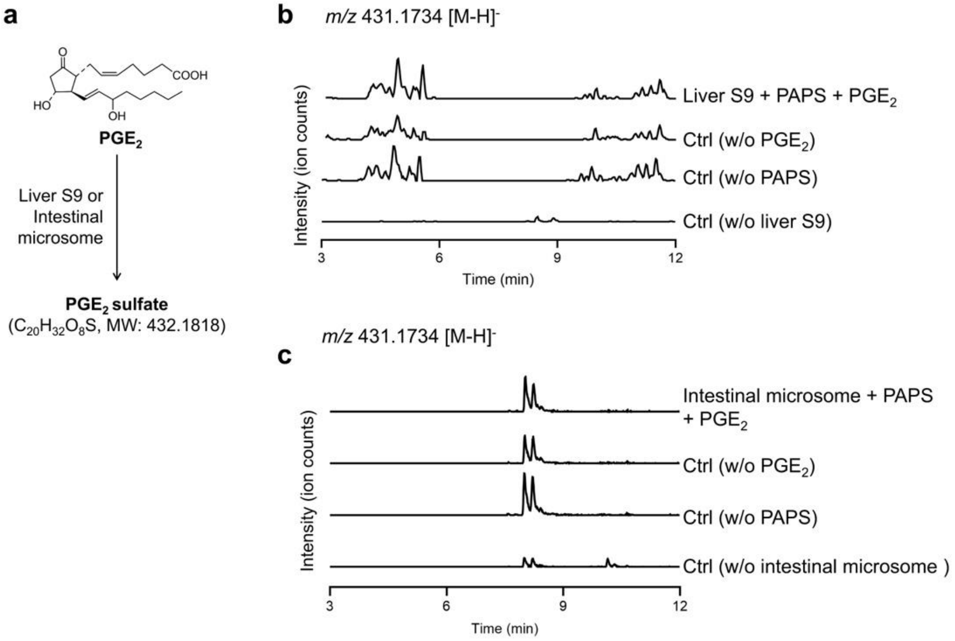

Extended Data Fig. 4

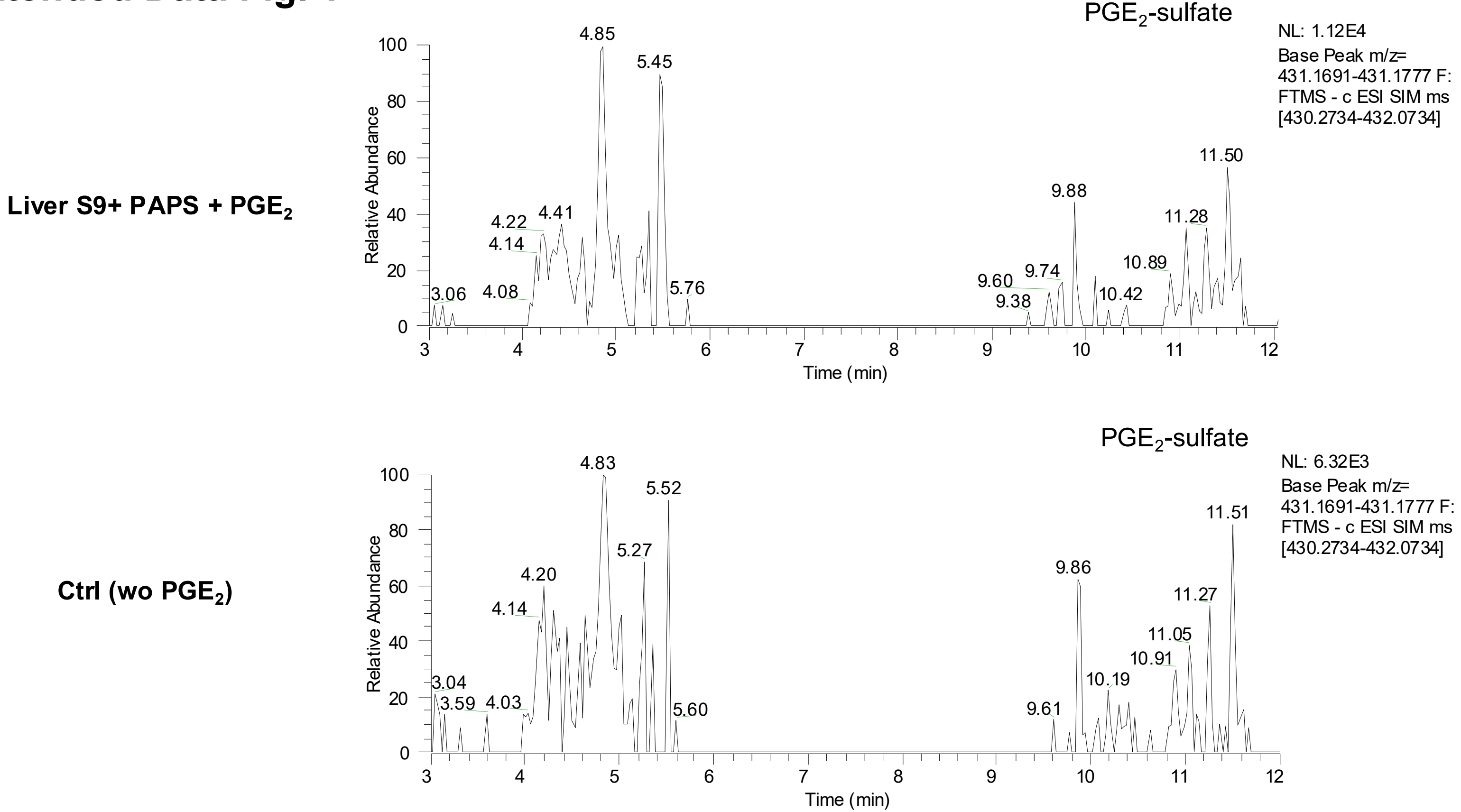

Extended Data Fig. 4

Ctrl (w/o PAPS)

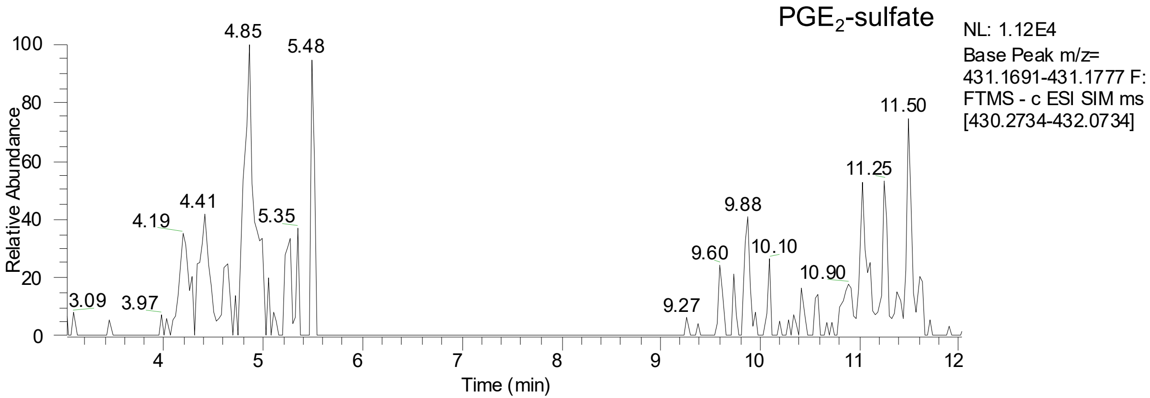

Ctrl(w/o liver S9)

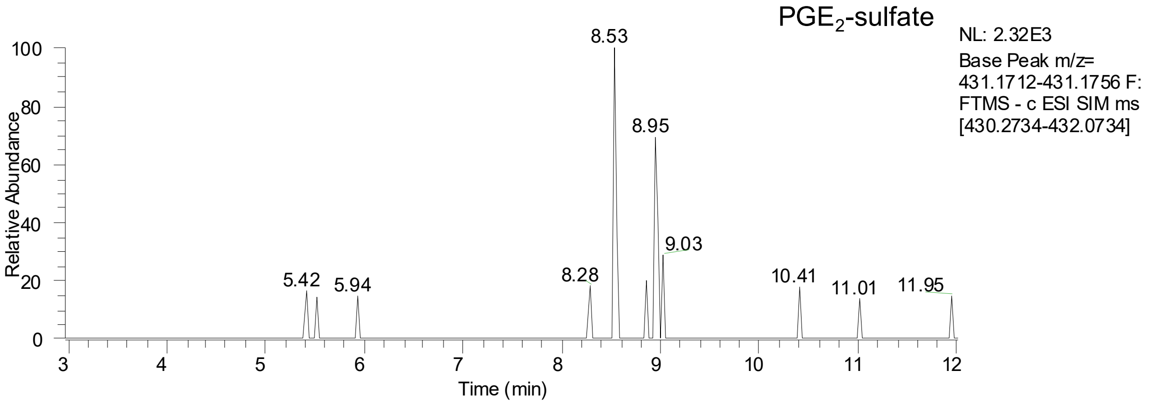

Extended Data Fig. 4

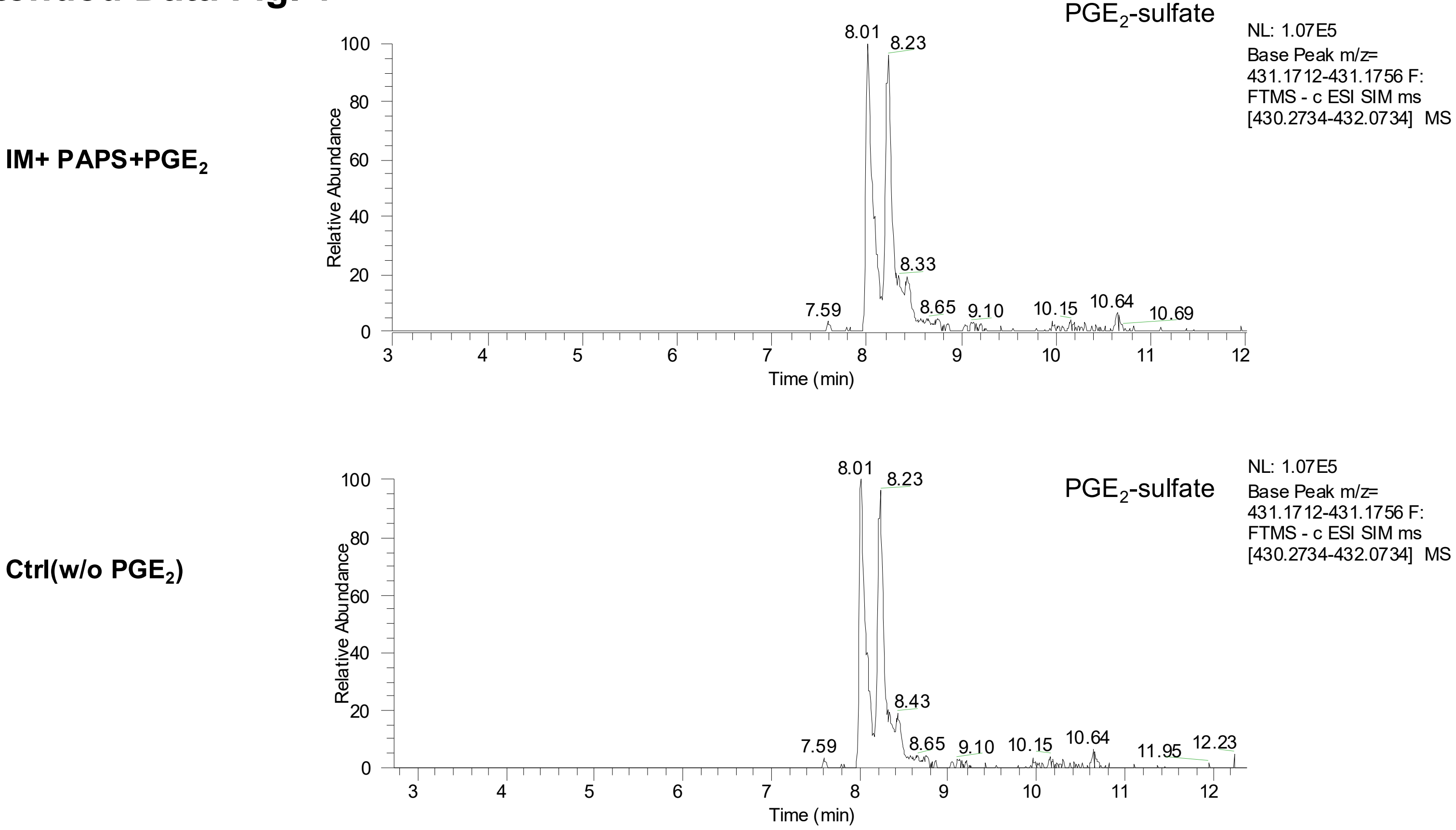

Extended Data Fig. 4

Ctrl(w/o PAPS)

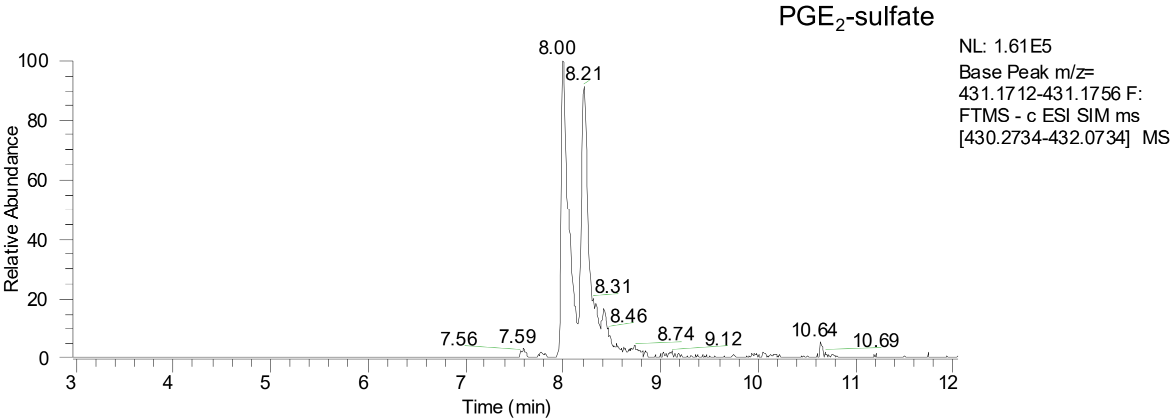

Ctrl (w/o IM)

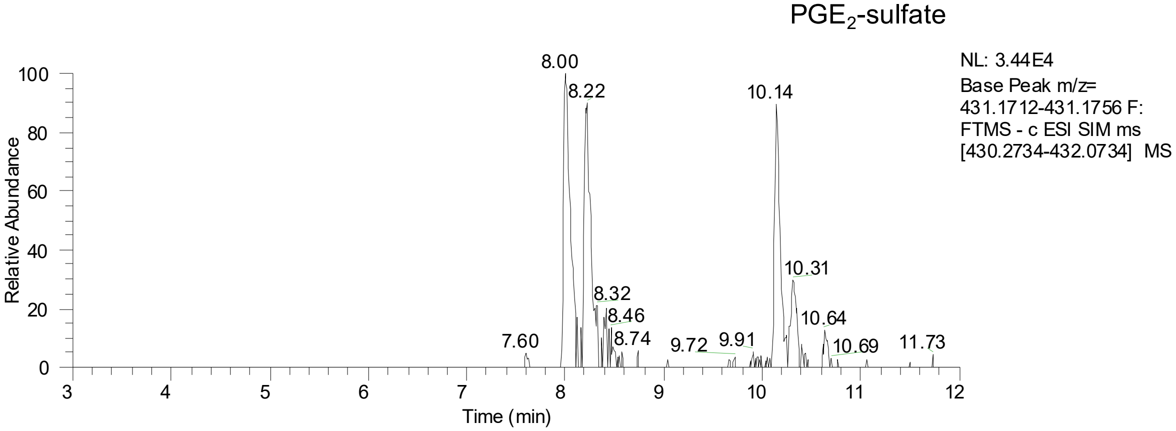

Extended Data Fig. 4
TSQ LC–MS/MS spectrometry of PGE_2-glucuronide

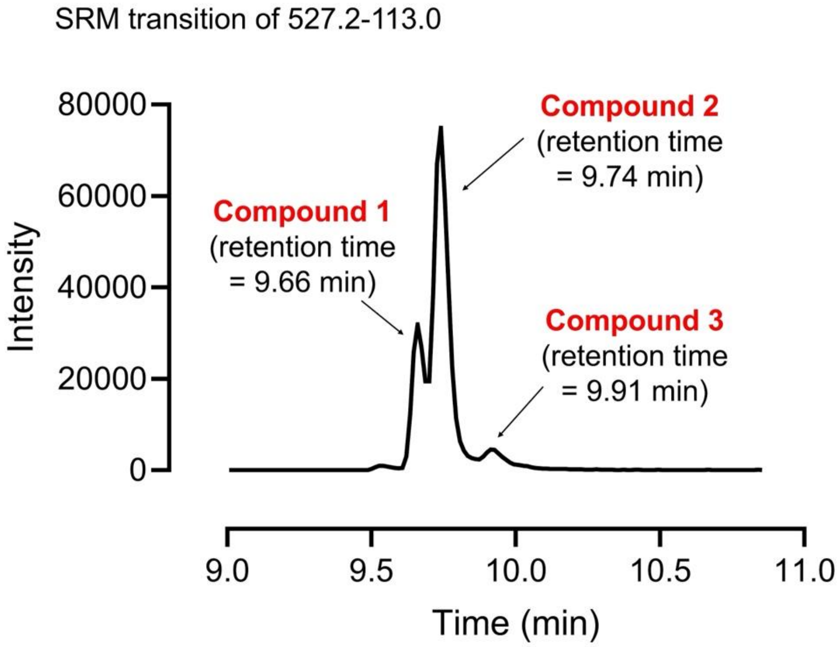

Extended Data Fig. 4

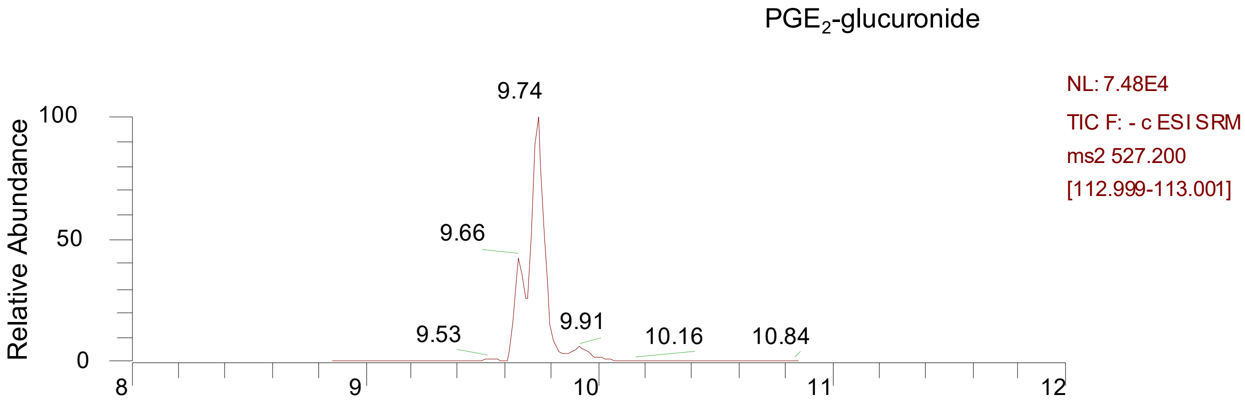

Extended Data Fig. 13
TSQ LC–MS/MS spectrometry of PGE_2-Acyl-GlcA in liver microsome reaction

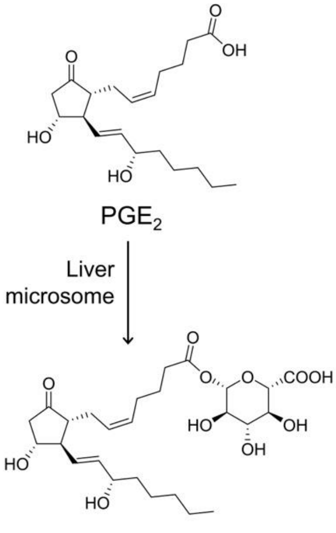

Compound 1: PGE_2-Acyl-GlcA

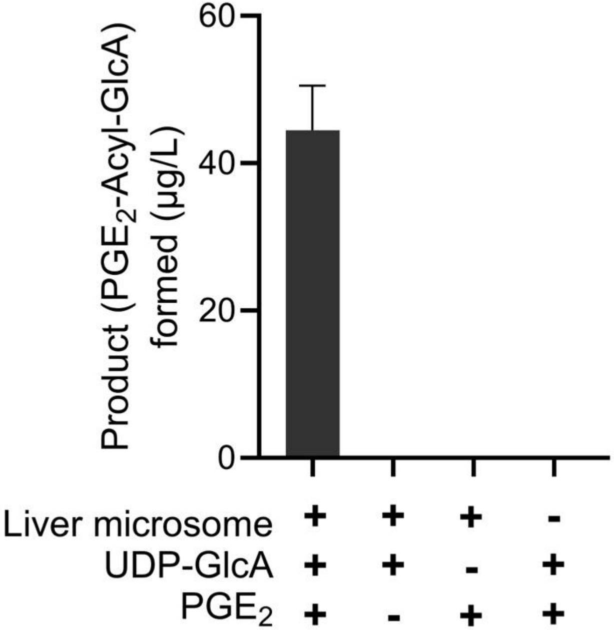

Extended Data Fig. 13

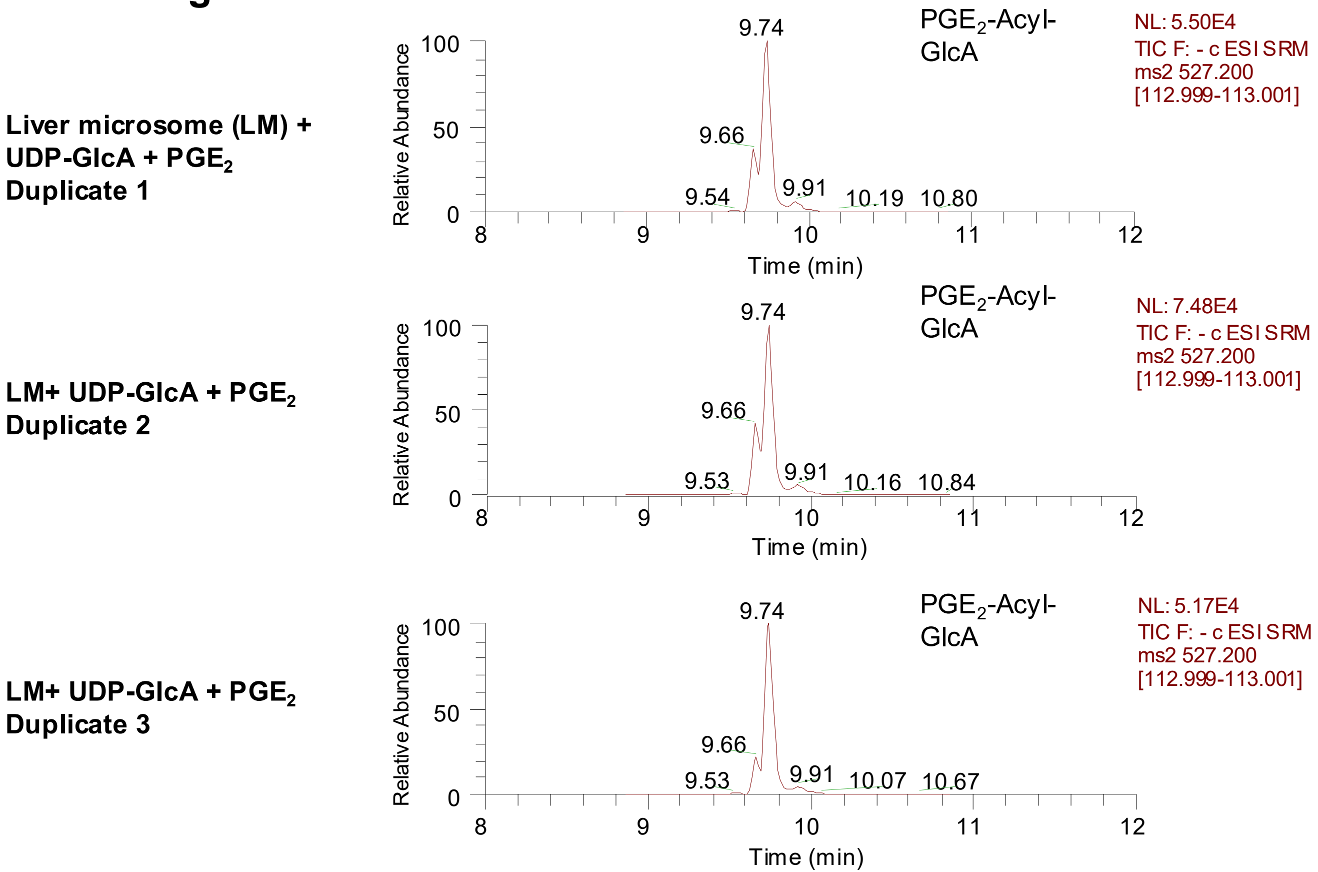

Extended Data Fig. 13

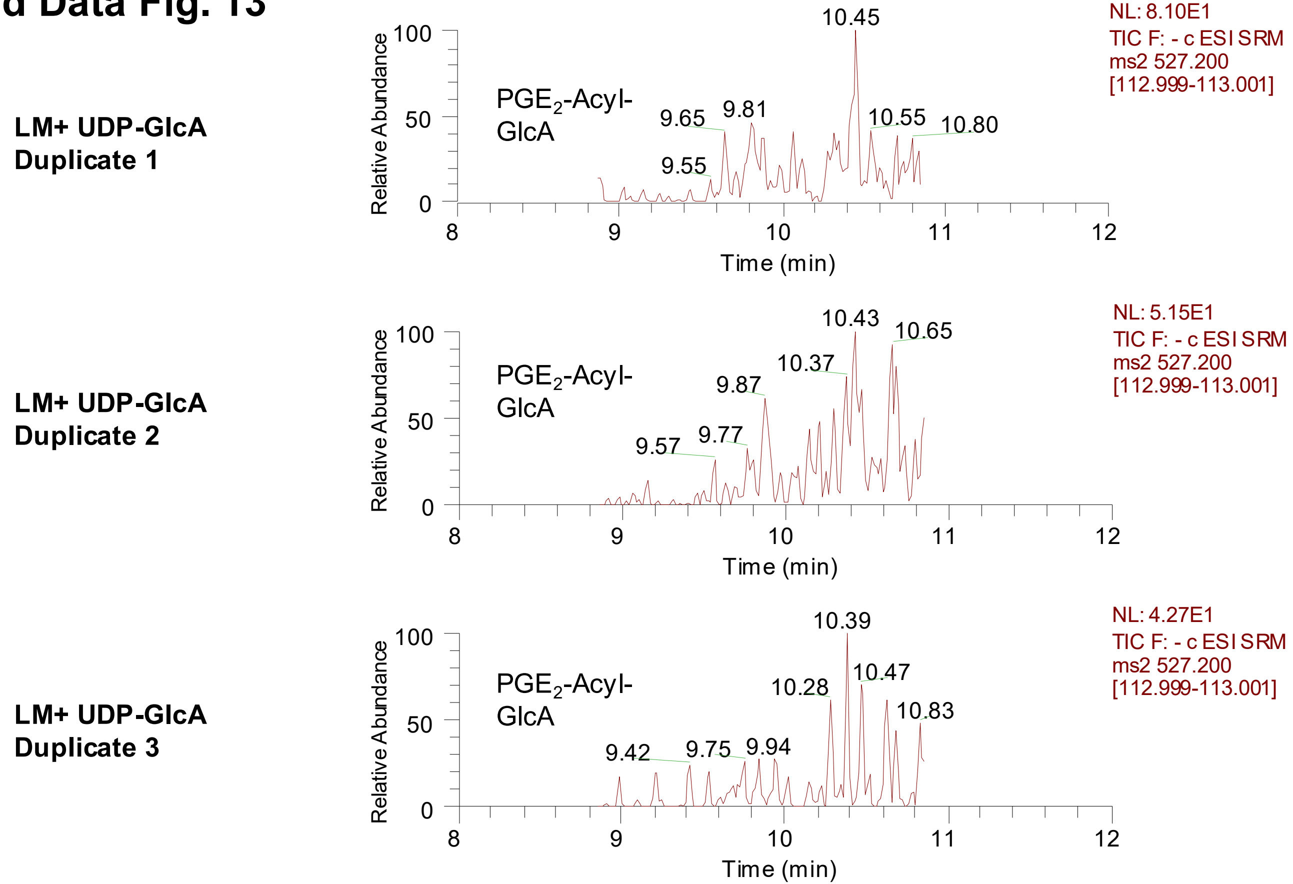

Extended Data Fig. 13

LM+ PGE_2 Duplicate 1

LM+ PGE_2 Duplicate 2

LM+ PGE_2 Duplicate 3

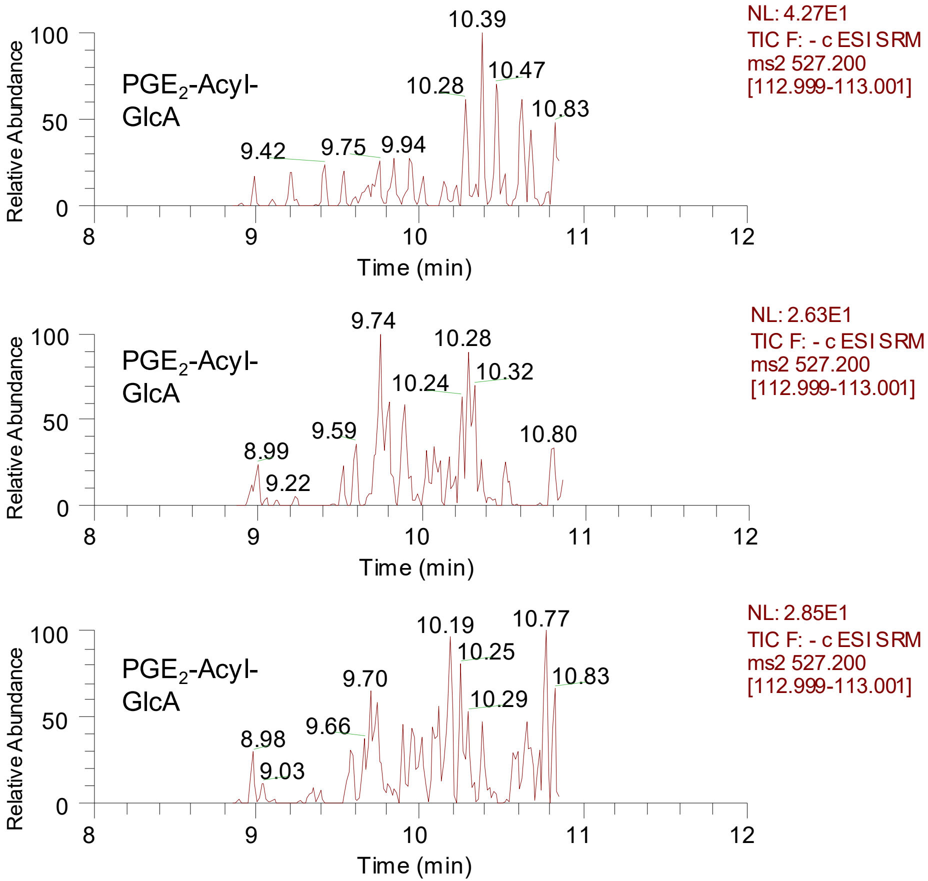

Extended Data Fig. 13

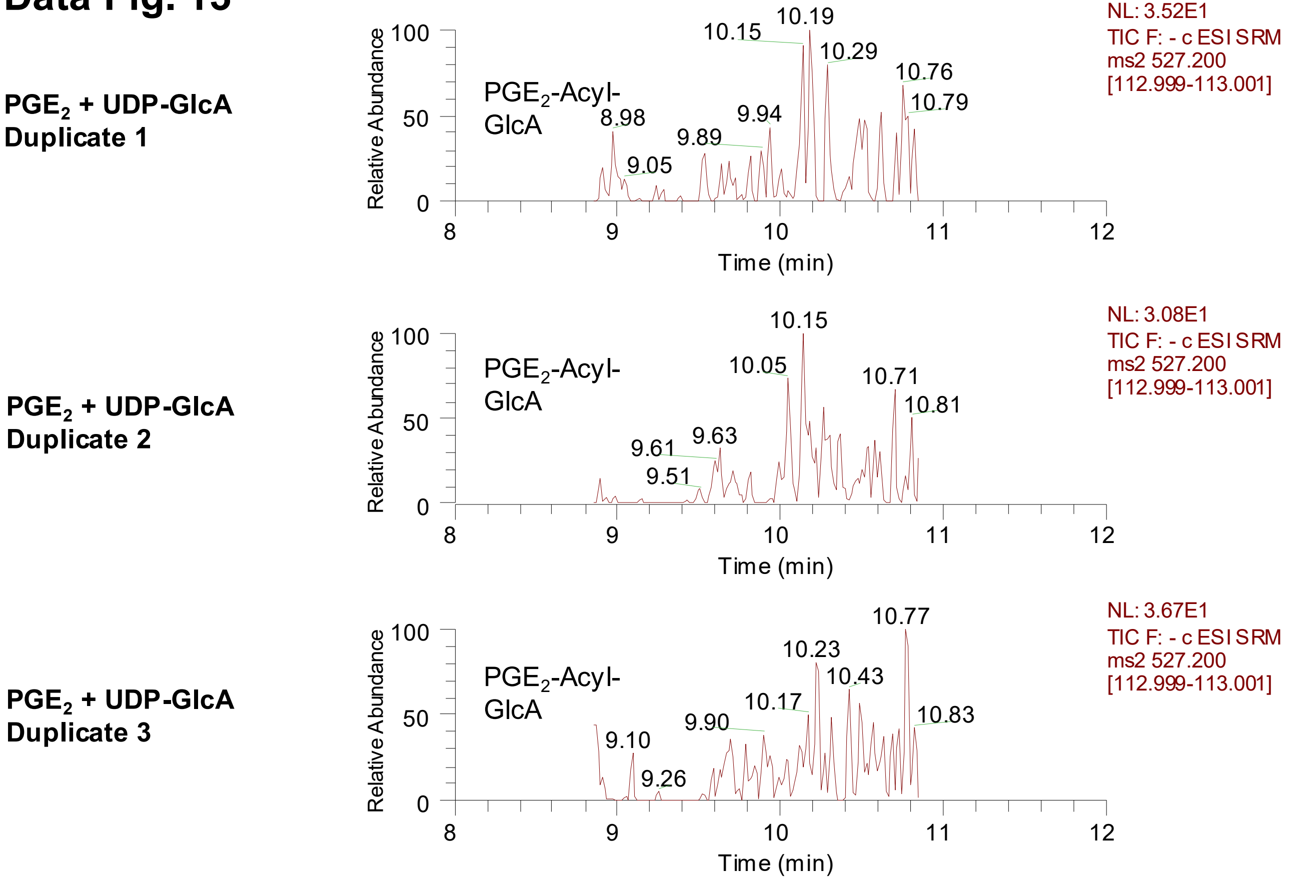

Extended Data Fig. 18 LC-HRMS spectrometry of other PG-glucuronides in intestinal microsome reaction

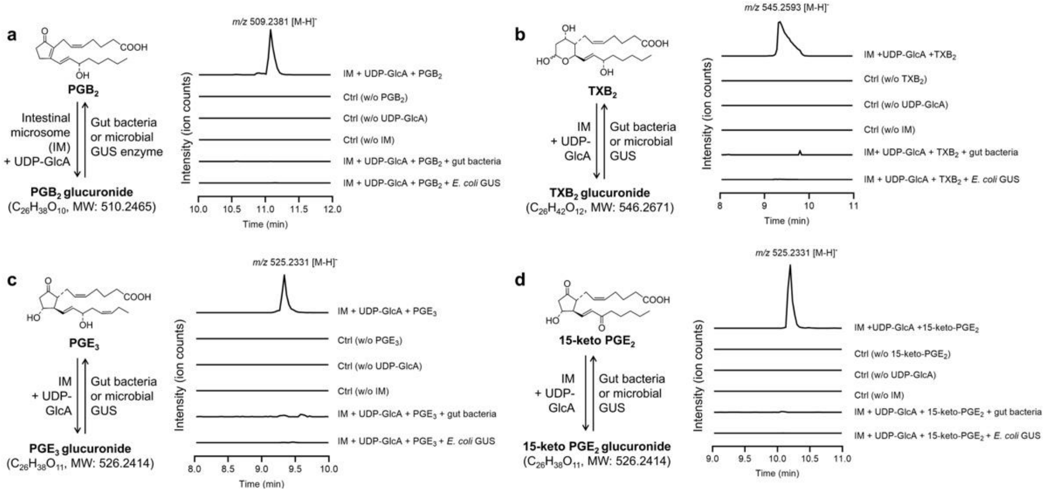

Extended Data Fig. 18

IM + UDP-GlcA + PGB_2

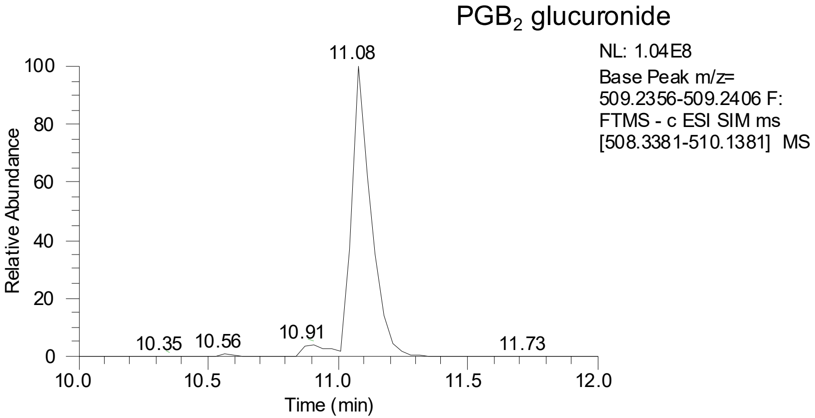

Ctrl （w/o PGB_2)

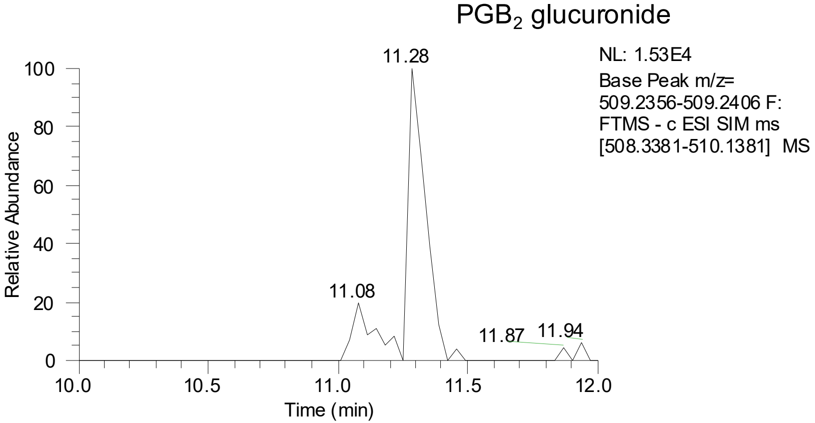

Extended Data Fig. 18

PGB$_2$ glucuronide

Ctrl（w/o UDP-GlcA)

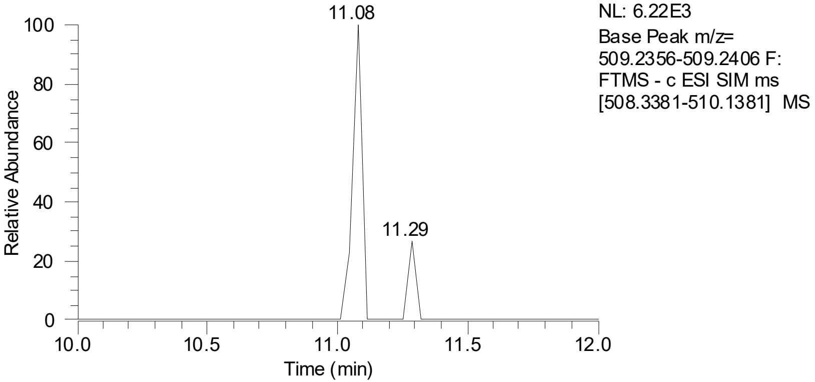

PGB$_2$ glucuronide

Ctrl（w/o IM)

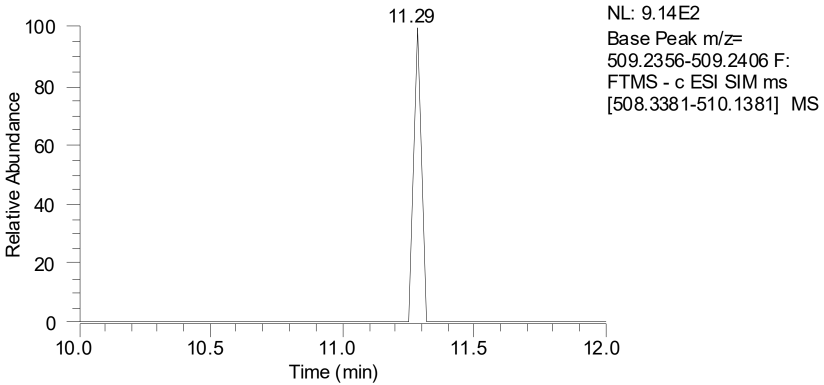

Extended Data Fig. 18

IM + UDP-GlcA + PGB_2+ gut bacteria

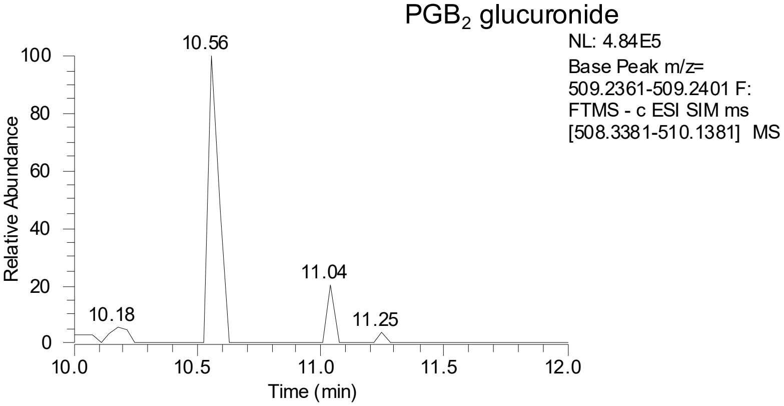

IM + UDP-GlcA + PGB_2 + *E. coli* GUS

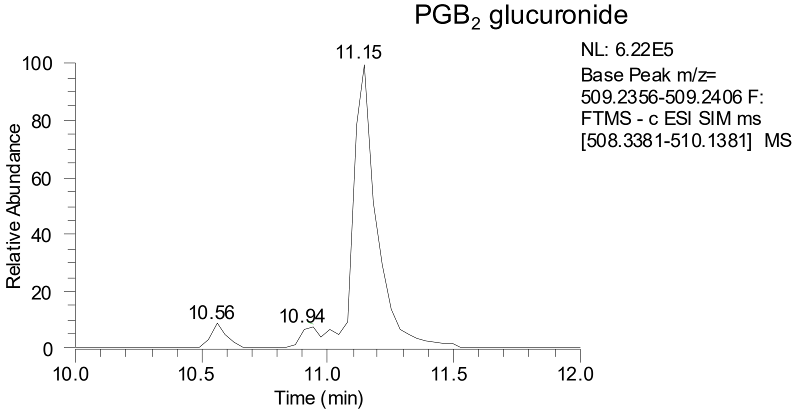

Extended Data Fig. 18

IM + UDP-GlcA + TXB$_2$

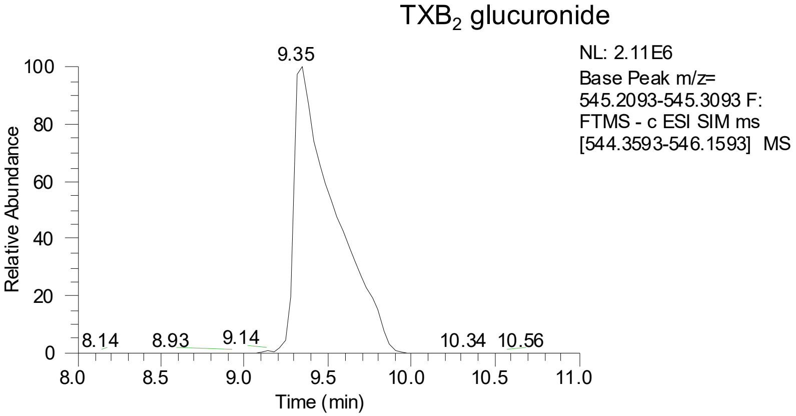

Ctrl (w/o TXB$_2$)

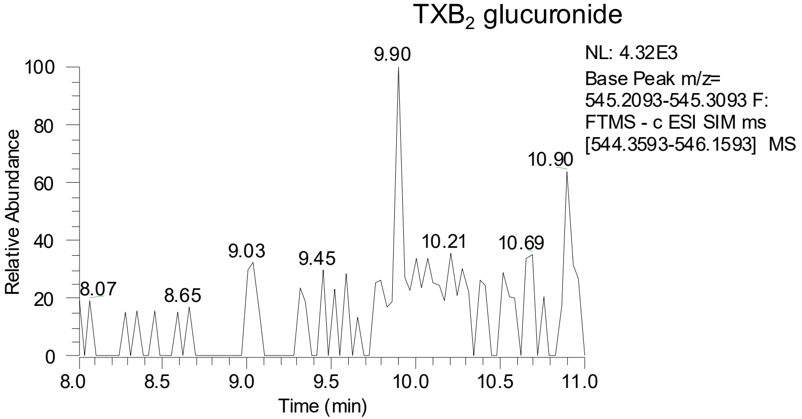

Extended Data Fig. 18

Ctrl（w/o UDP-GlcA)

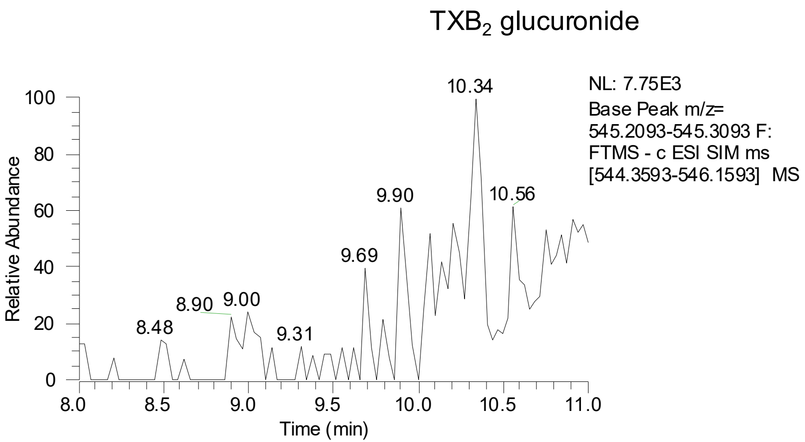

Ctrl（w/o IM)

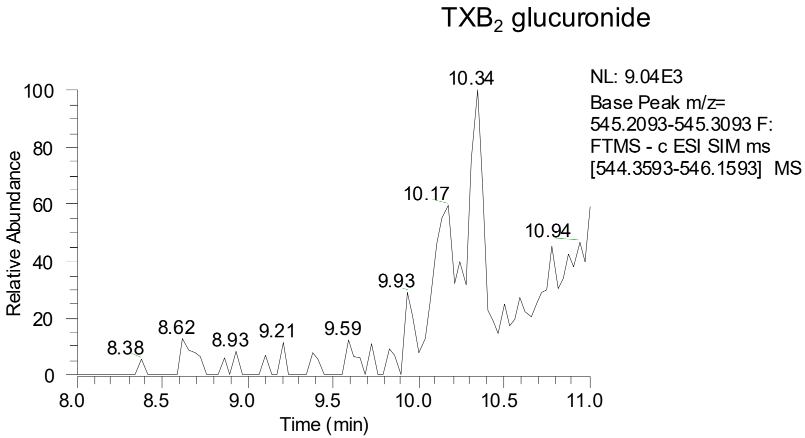

Extended Data Fig. 18

IM + UDP-GlcA + TXB_2+ gut bacteria

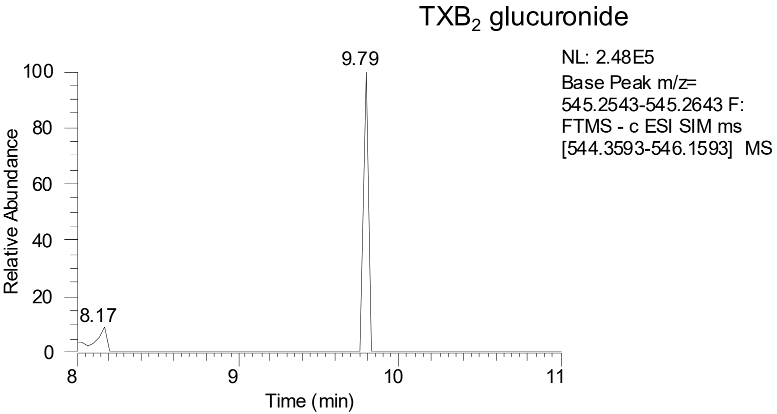

IM + UDP-GlcA + TXB_2 +
***E. coli* GUS**

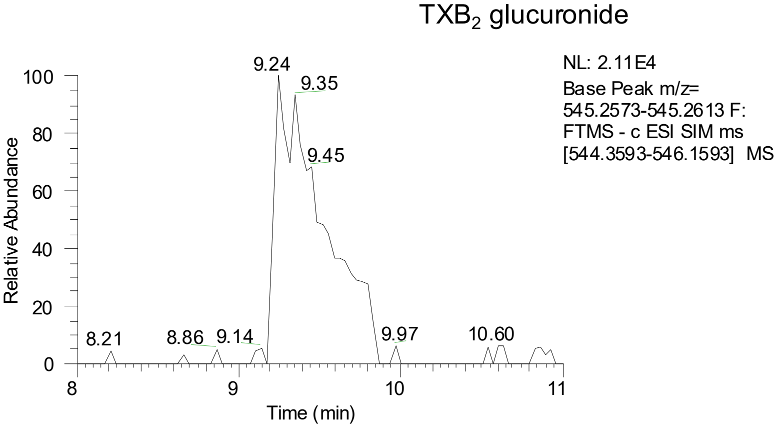

Extended Data Fig. 18

IM + UDP-GlcA + PGE$_3$

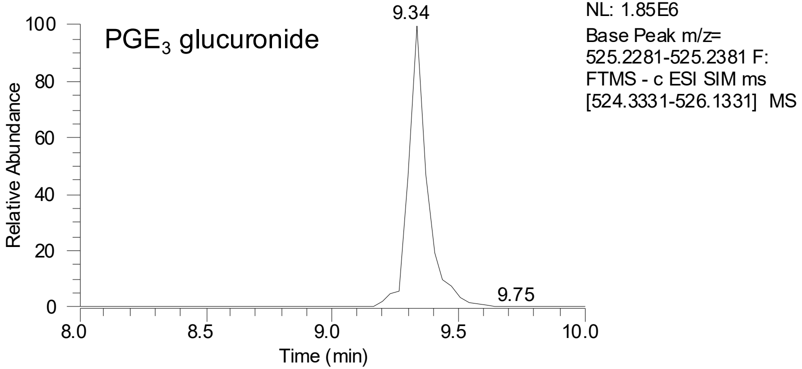

Ctrl（w/o PGE$_3$)

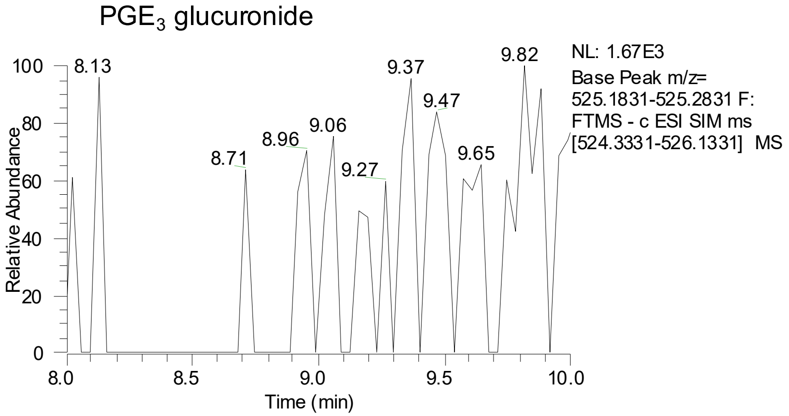

Extended Data Fig. 18

Ctrl（w/o UDP-GlcA)

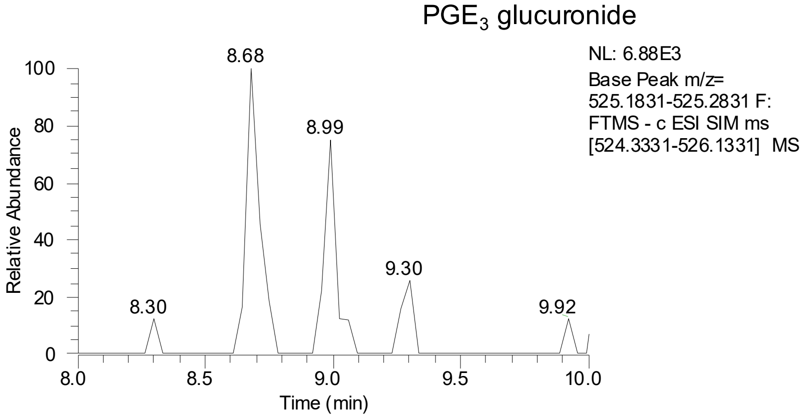

Ctrl（w/o IM)

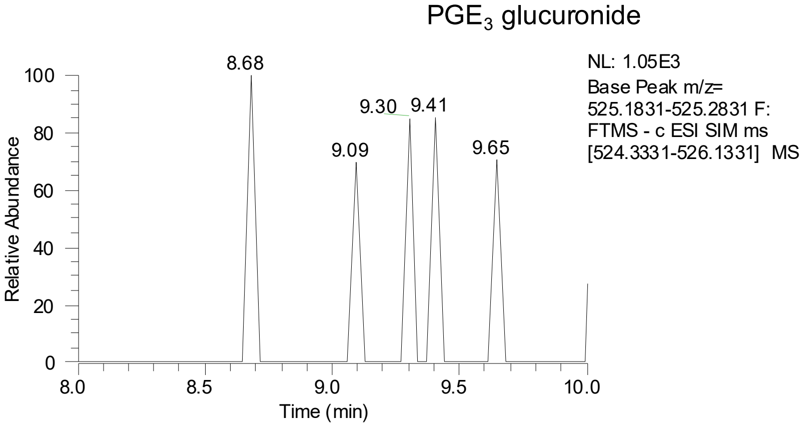

Extended Data Fig. 18

IM + UDP-GlcA + PGE$_3$+ gut bacteria

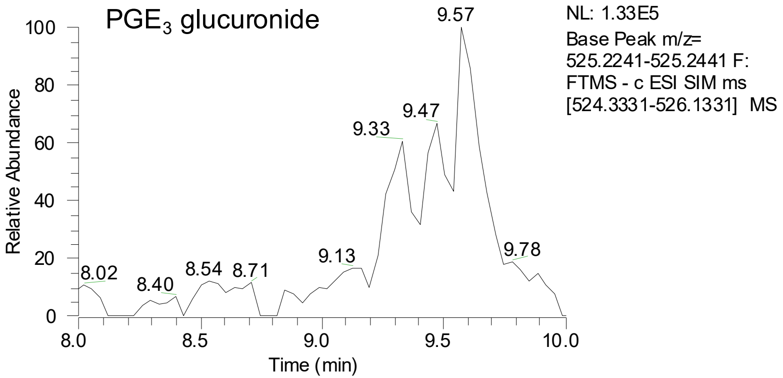

IM + UDP-GlcA + PGE$_3$ +
***E. coli* GUS**

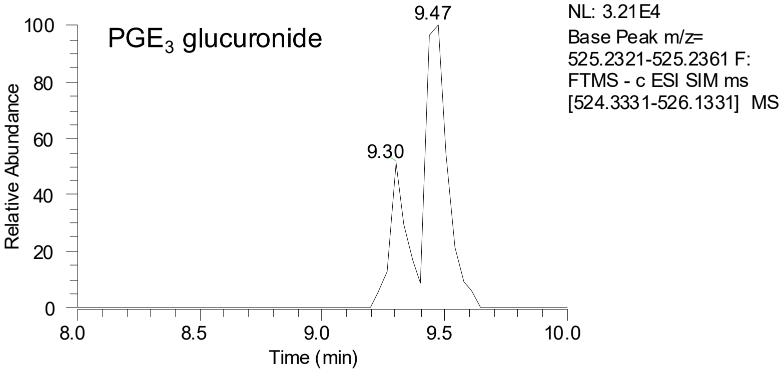

Extended Data Fig. 18

15-keto PGE_2 glucuronide

IM + UDP-GlcA + 15-keto PGE_2

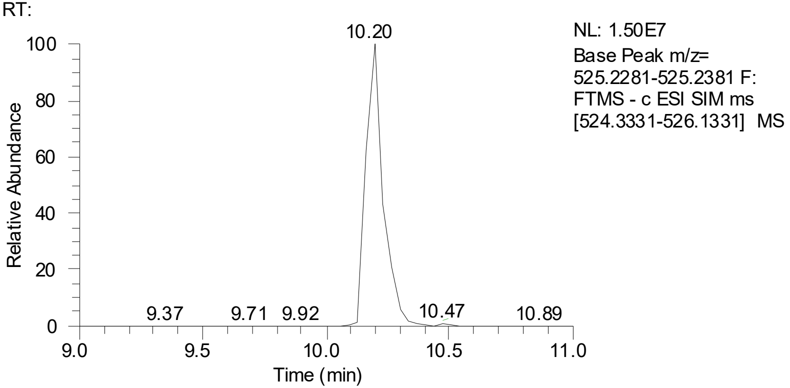

15-keto PGE_2 glucuronide

Ctrl (w/o 15-keto PGE_2)

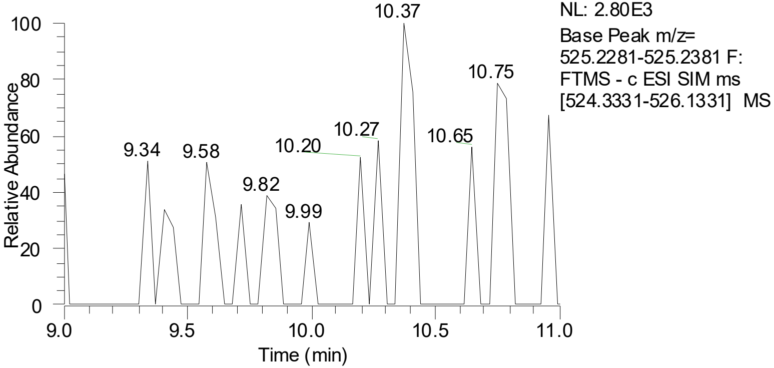

Extended Data Fig. 18

Ctrl（w/o UDP-GlcA)

15-keto PGE_2 glucuronide

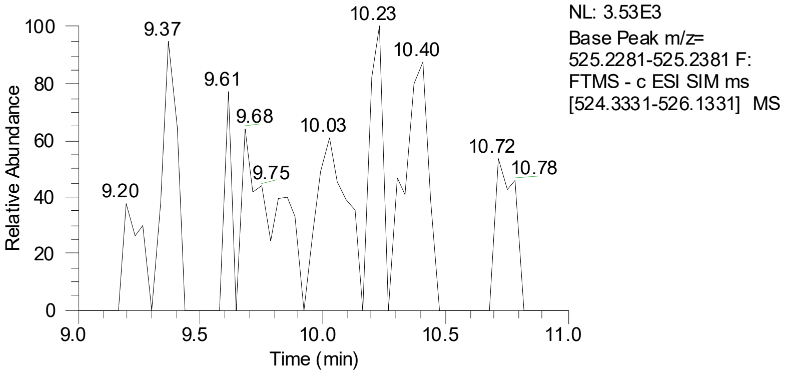

Ctrl（w/o IM)

15-keto PGE_2 glucuronide

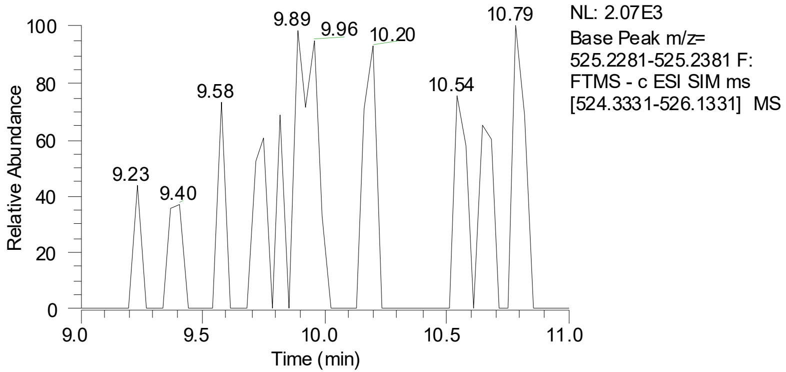

Extended Data Fig. 18

IM + UDP-GlcA + 15-keto PGE_2 + gut bacteria

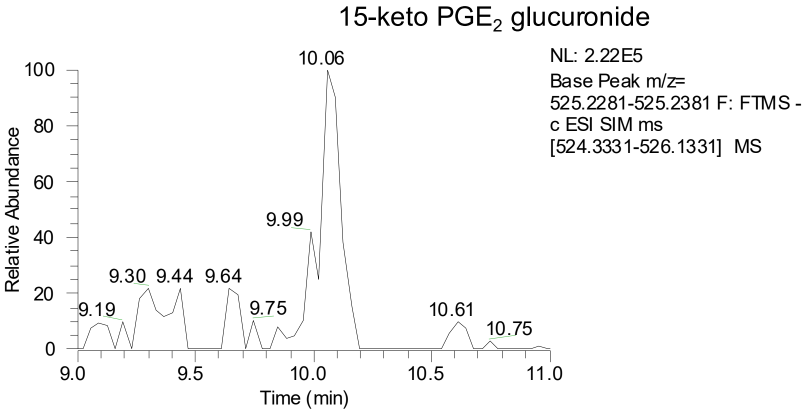

IM + UDP-GlcA + 15-keto PGE_2 + *E. coli* GUS

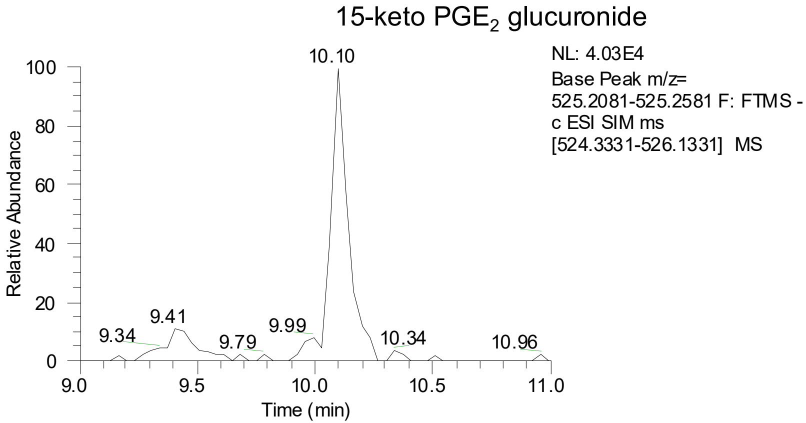

Extended Data Fig. 21
TSQ LC–MS/MS spectrometry of PGE_2-Acyl-GlcA in human UGT isoform's reaction

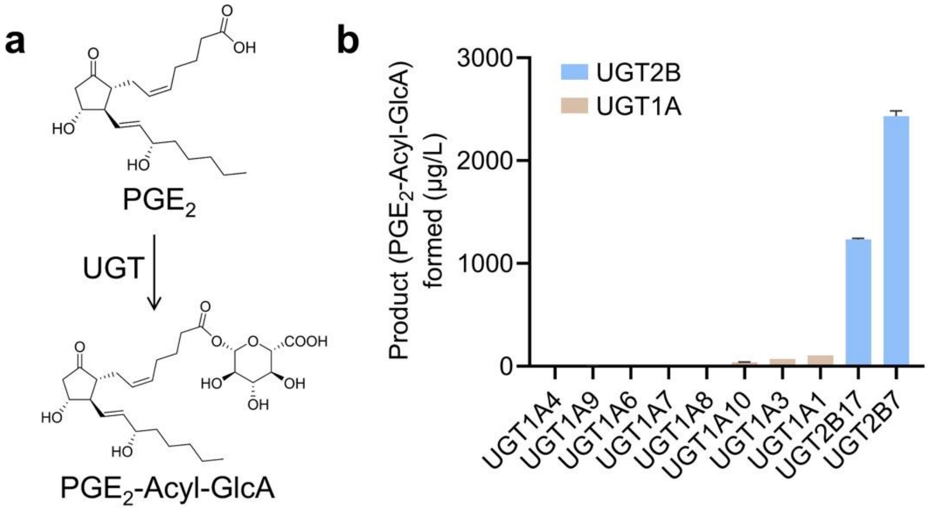

Extended Data Fig. 21

UGT1A4
Duplicate 1

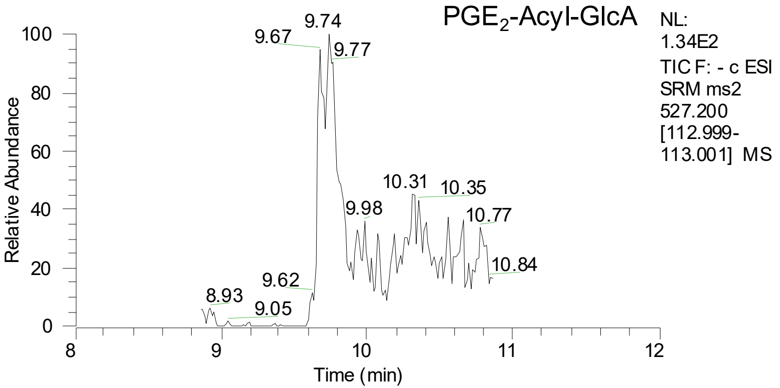

UGT1A4
Duplicate 2

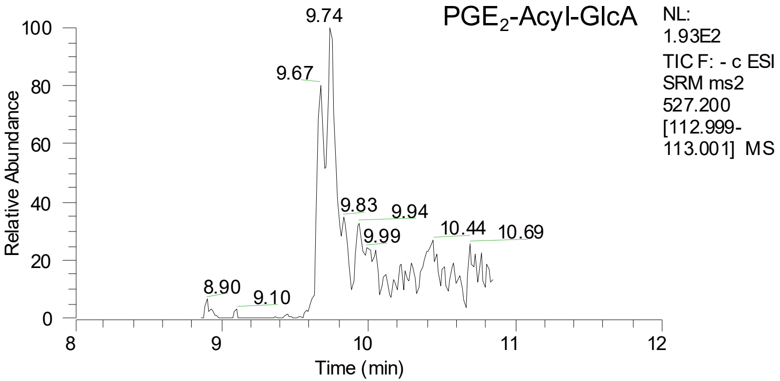

Extended Data Fig. 21

UGT1A9
Duplicate 1

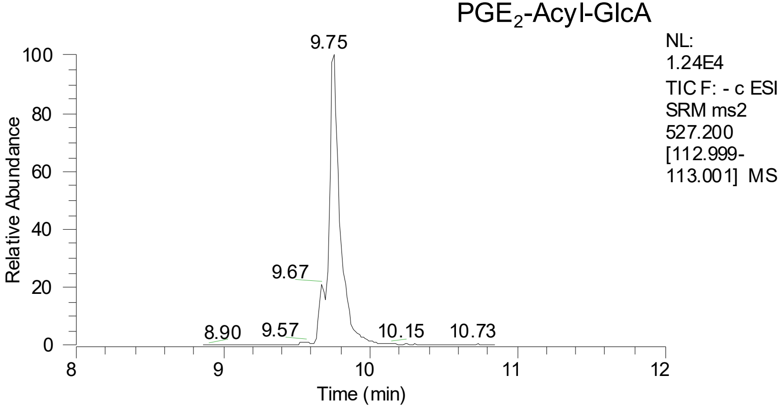

UGT1A9
Duplicate 2

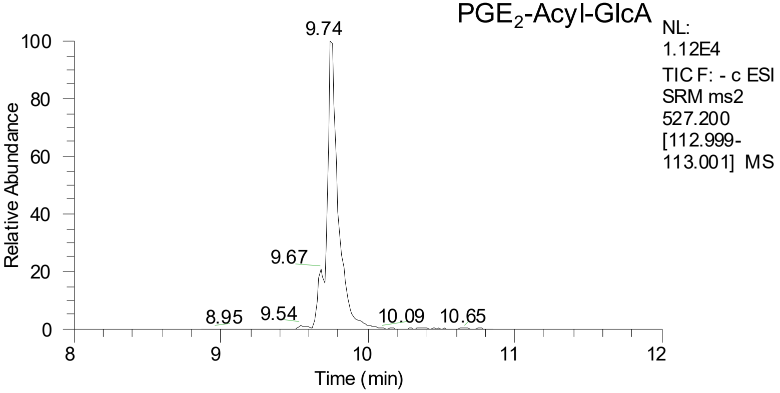

Extended Data Fig. 21

UGT1A6 Duplicate 1

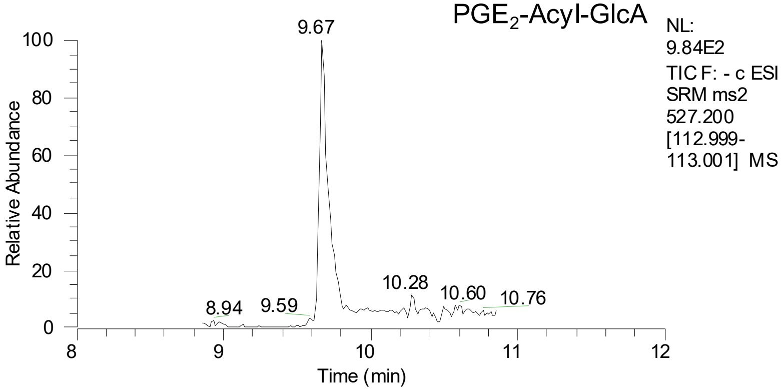

UGT1A6 Duplicate 2

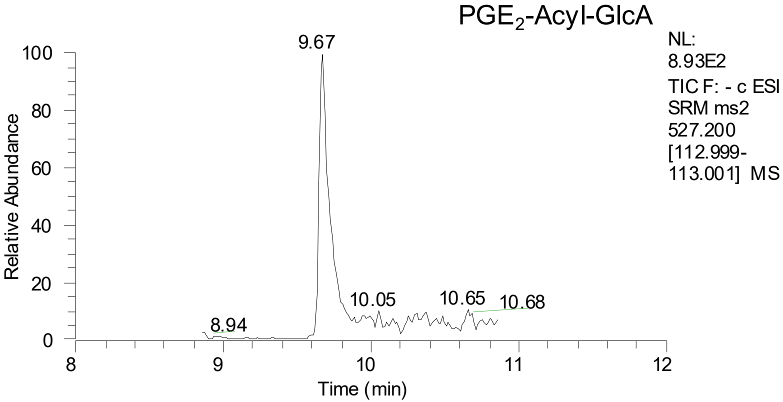

Extended Data Fig. 21

UGT1A7
Duplicate 1

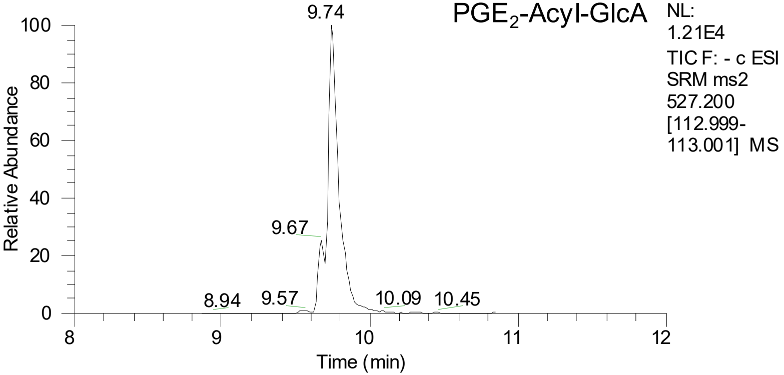

UGT1A7
Duplicate 2

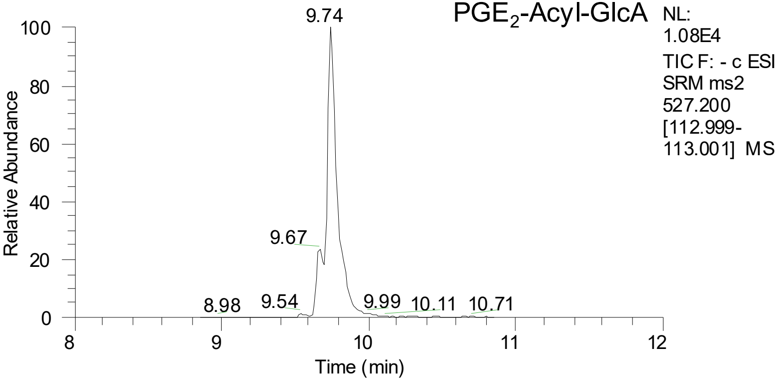

Extended Data Fig. 21

UGT1A8
Duplicate 1

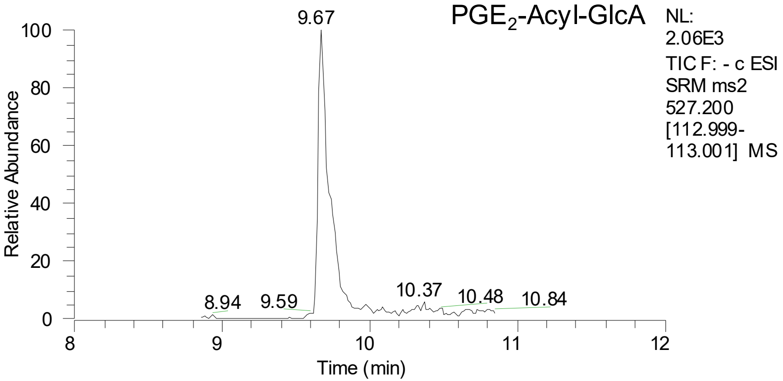

UGT1A8
Duplicate 2

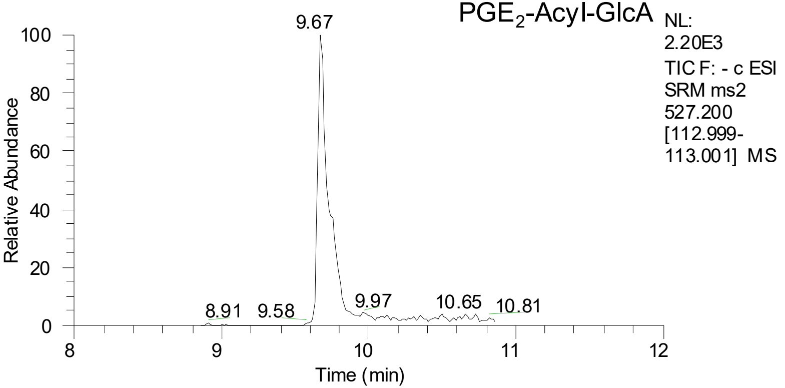

Extended Data Fig. 21

UGT1A10
Duplicate 1

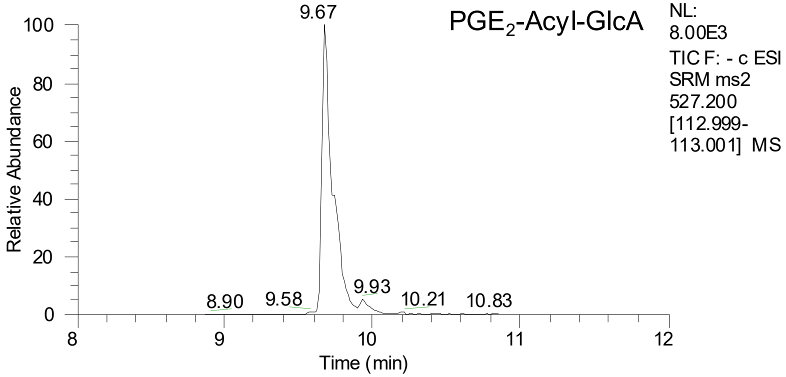

UGT1A10
Duplicate 2

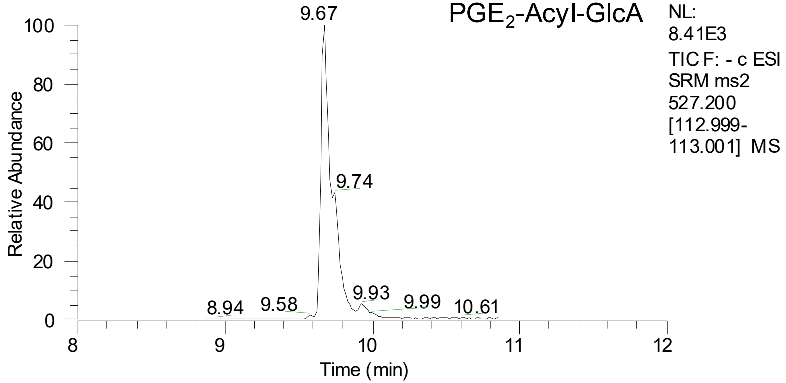

Extended Data Fig. 21

UGT1A3 Duplicate 1

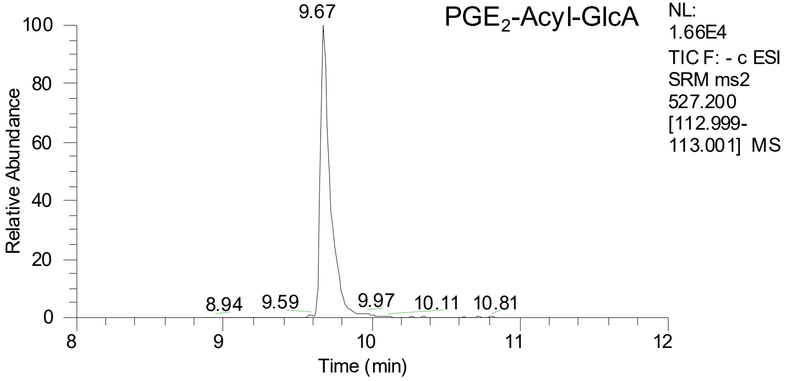

UGT1A3 Duplicate 2

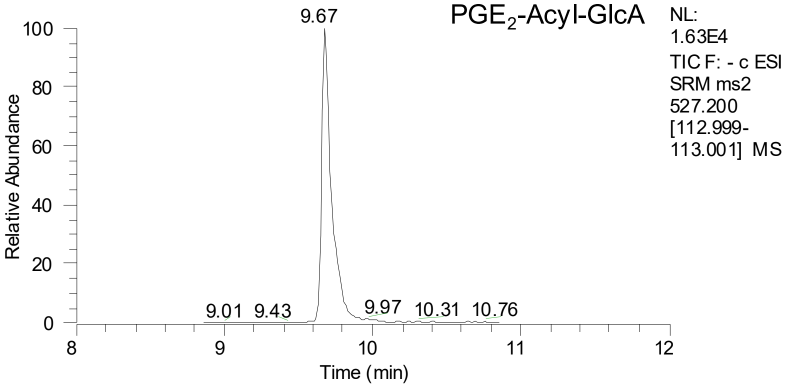

Extended Data Fig. 21

UGT1A1 Duplicate 1

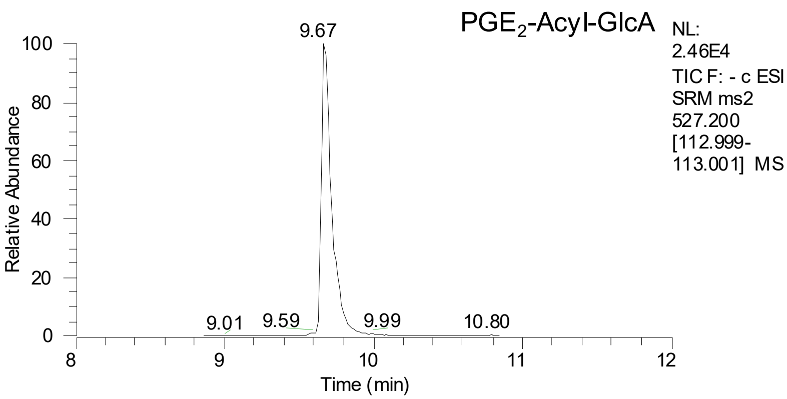

UGT1A1 Duplicate 2

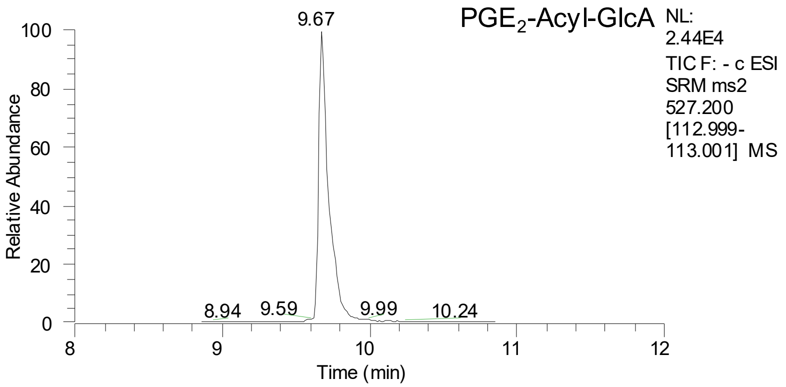

Extended Data Fig. 21

UGT2B17
Duplicate 1

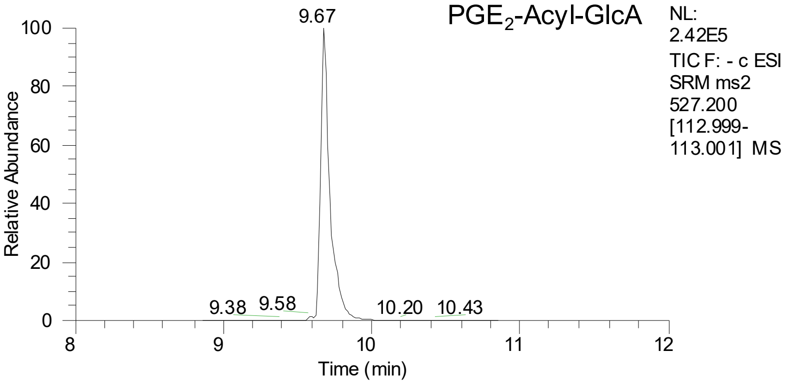

UGT2B17
Duplicate 2

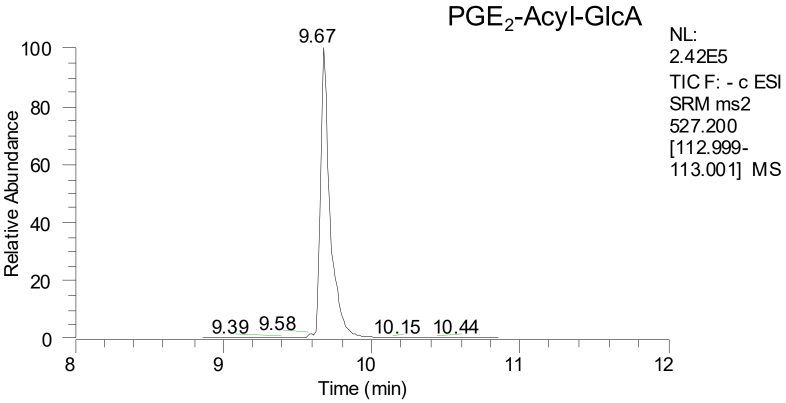

Extended Data Fig. 21

UGT2B7
Duplicate 1

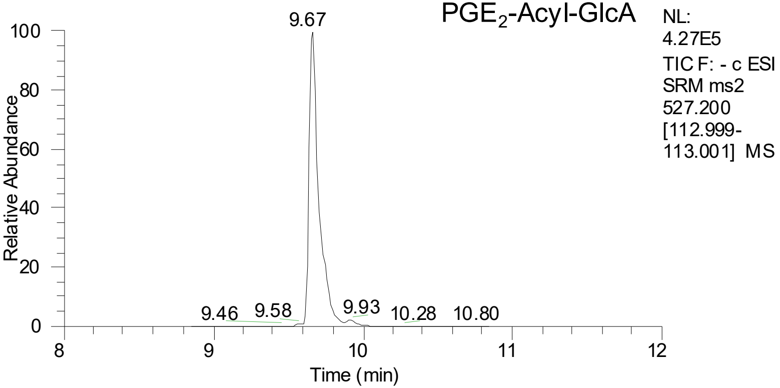

UGT2B7
Duplicate 2

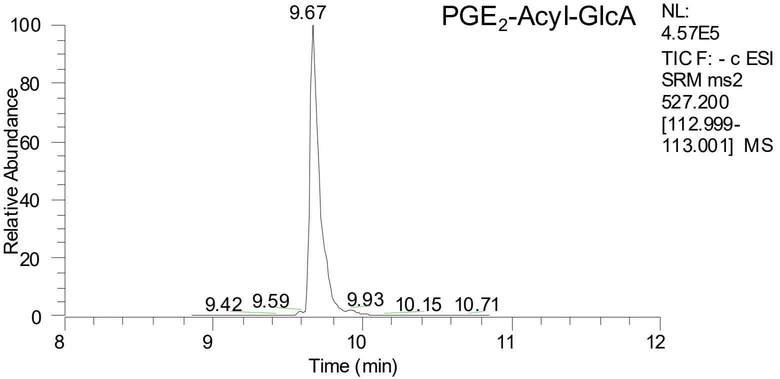

Supplement: 1 [file NIHPPrs8856024V1-supplement-1.pdf]
